# Supplementary material for: A ligand-directed divergent catalytic approach to establish structural and functional scaffold diversity
Source: Nat Commun. 2017 Feb 14;8:14043. doi: 10.1038/ncomms14043 (PMC5316858; doi:10.1038/ncomms14043)
Supplement: Supplementary Information — Supplementary Figures, Supplementary Tables, Supplementary Methods and Supplementary References. [file ncomms14043-s1.pdf]

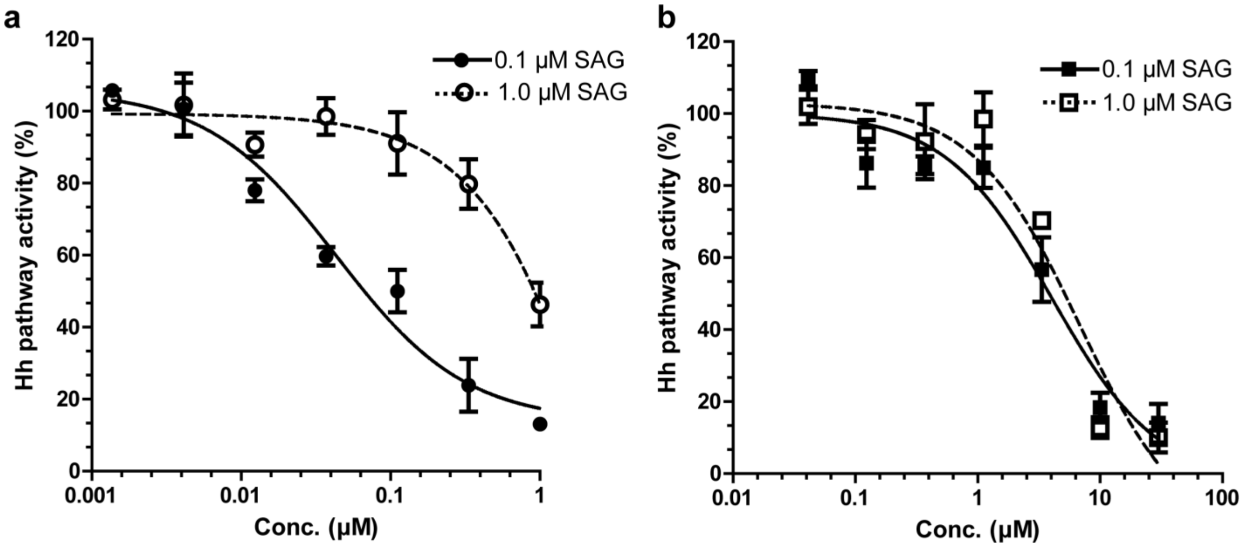

Supplementary Figure 1. SAG competition assay with vismodegib and GANT 61. Influence of vismodegib (**a**) and GANT-61 (**b**) on the Gli-mediated reporter gene expression upon Hh pathway activation in Shh Light II cells by SAG (0.1 µM and 1 µM). Nonlinear regression analysis was performed using a four parameter fit. Data are mean values of three independent experiments (n = 3) ± s.d. and were normalized to cells treated with the respective concentration of SAG (set to 100 %).

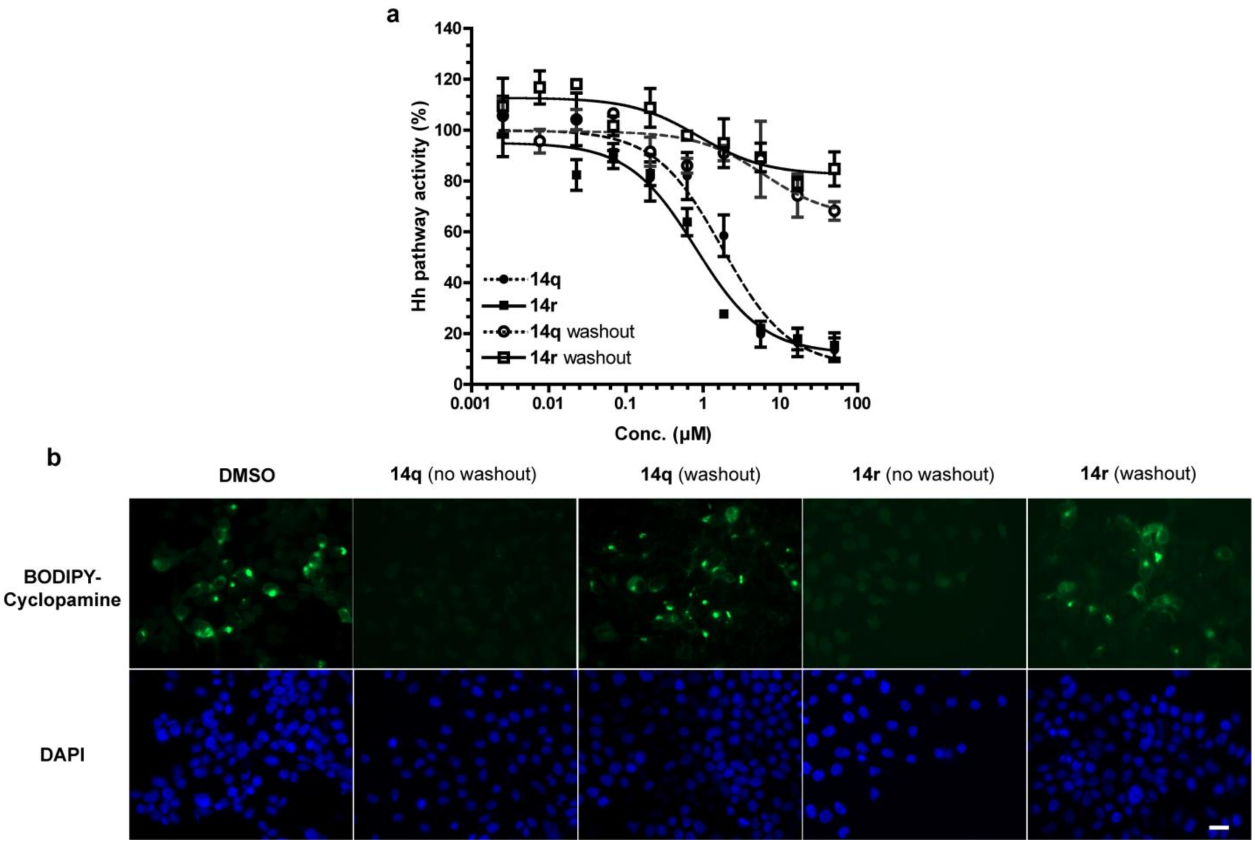

Supplementary Figure 2. Wash out experiment to access the mode of inhibition of **14q** and **14r**. (**a**) Hh signaling activity was determined using the in Gli-dependent reporter gene expression in Shh Light II cells. Cells were treated with 1.5 µM purmorphamine and different concentrations of compounds for 30 min prior to washout followed by addition of fresh media containing 1.5 µM purmorphamine or DMSO as a control and incubation for 48 h. Firefly and Renilla luciferase activities were then determined. Ratios of firefly luciferase/Renilla luciferase signals were calculated as a measure of Hedgehog pathway activity. Nonlinear regression analysis was performed using a four parameter fit. Data are mean values of three independent experiments (n = 3) ± s.d. (**b**) HEK293T cells were transiently transfected with Smo-expressing plasmid or empty vector. 48 h later cells were treated with 10 µM of **14q** or **14r** and DMSO as a control for one hour. Cells were then washed three times with media and supplemented with 5 nM BODIPY-cyclopamine (green) for 1 h prior to fixation and staining with DAPI to visualize the nuclei (blue). Scale bar: 20 µm.

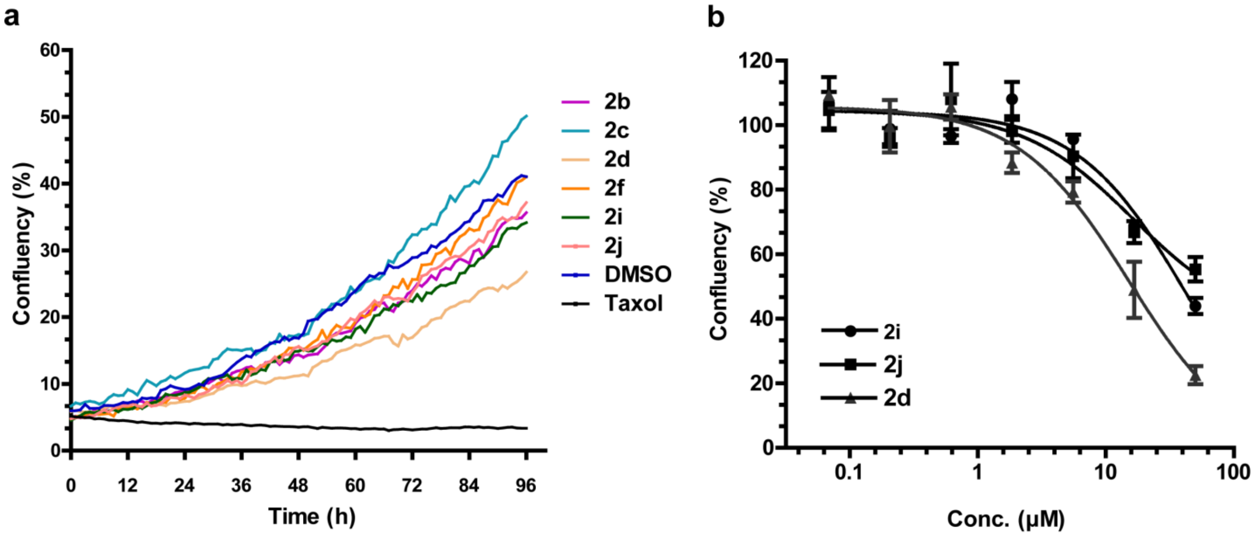

Supplementary Figure 3. Influence of selected compounds on HeLa cell proliferation. (**a**) HeLa cells were treated with the compounds and DMSO or taxol as controls for 96 h. Live-cell imaging using the IncuCyte Zoom® imaging system was performed to assess cell confluency. The data are representative results of three independent replicates. (**b**) Dose-dependent decrease in cell confluency by compound **2i, 2j** and **2d** after treatment of HeLa cells for 72 h. Nonlinear regression analysis was performed using a four parameter fit. Data are mean values of three independent experiments (n = 3) ± s.d.

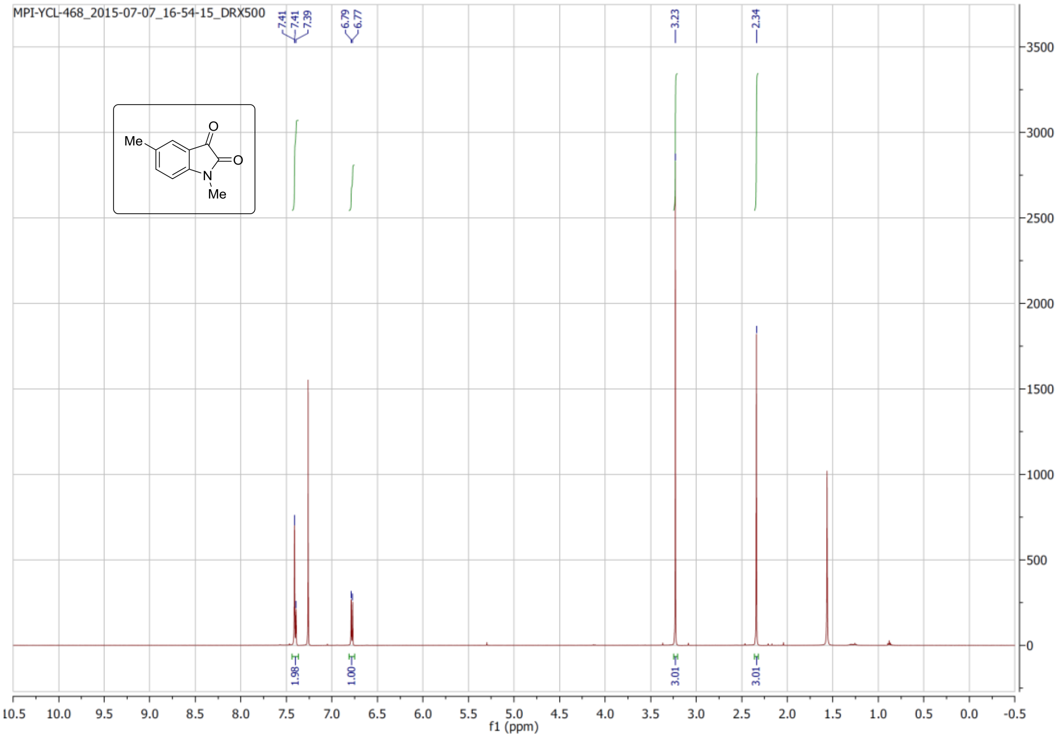

Supplementary Figure 4. ^{1}H NMR spectrum for **S8a**.

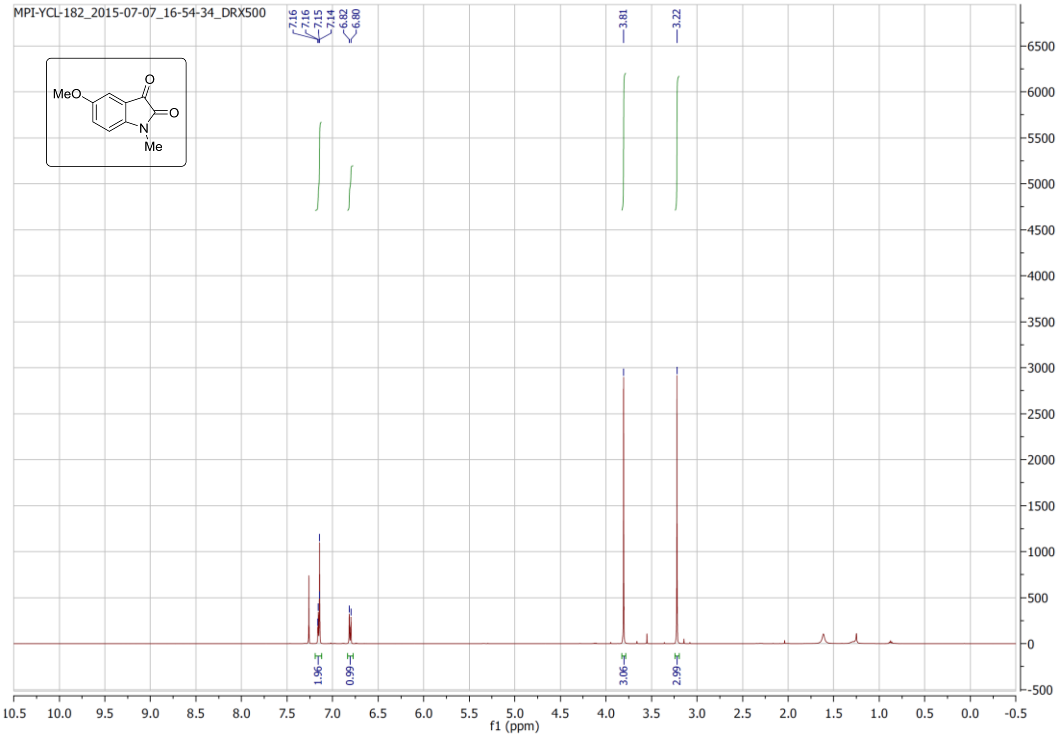

Supplementary Figure 5. ^{1}H NMR spectrum for **S8b**.

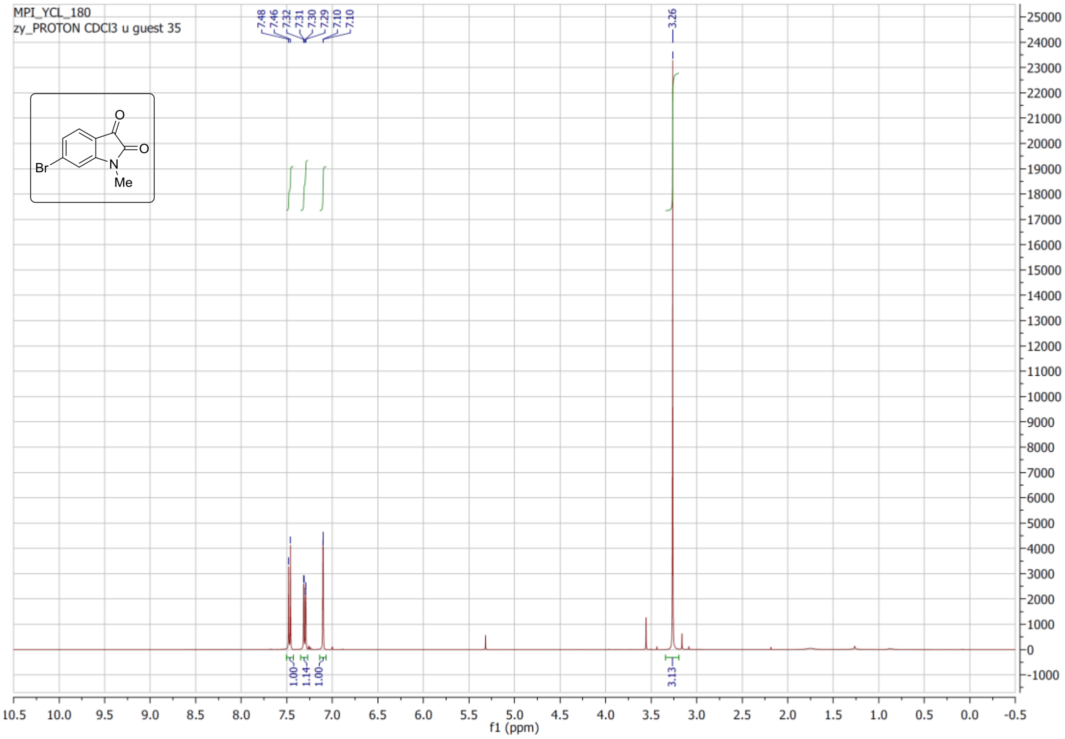

Supplementary Figure 6. ^{1}H NMR spectrum for **S8c**.

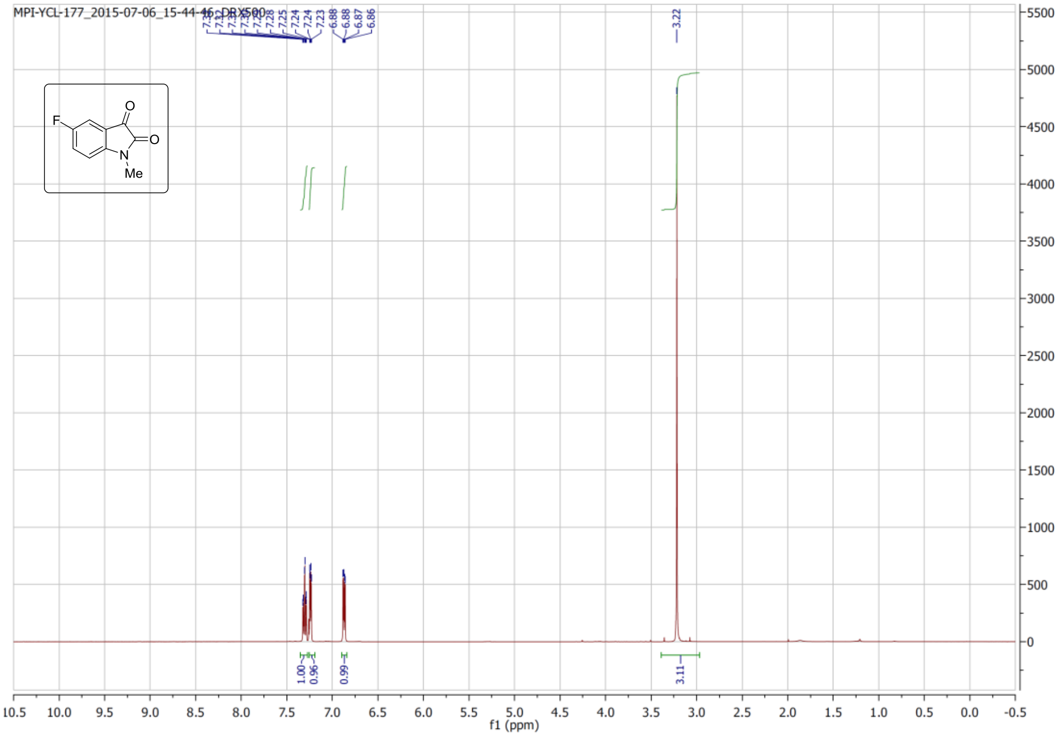

Supplementary Figure 7. ^{1}H NMR spectrum for **S8d**.

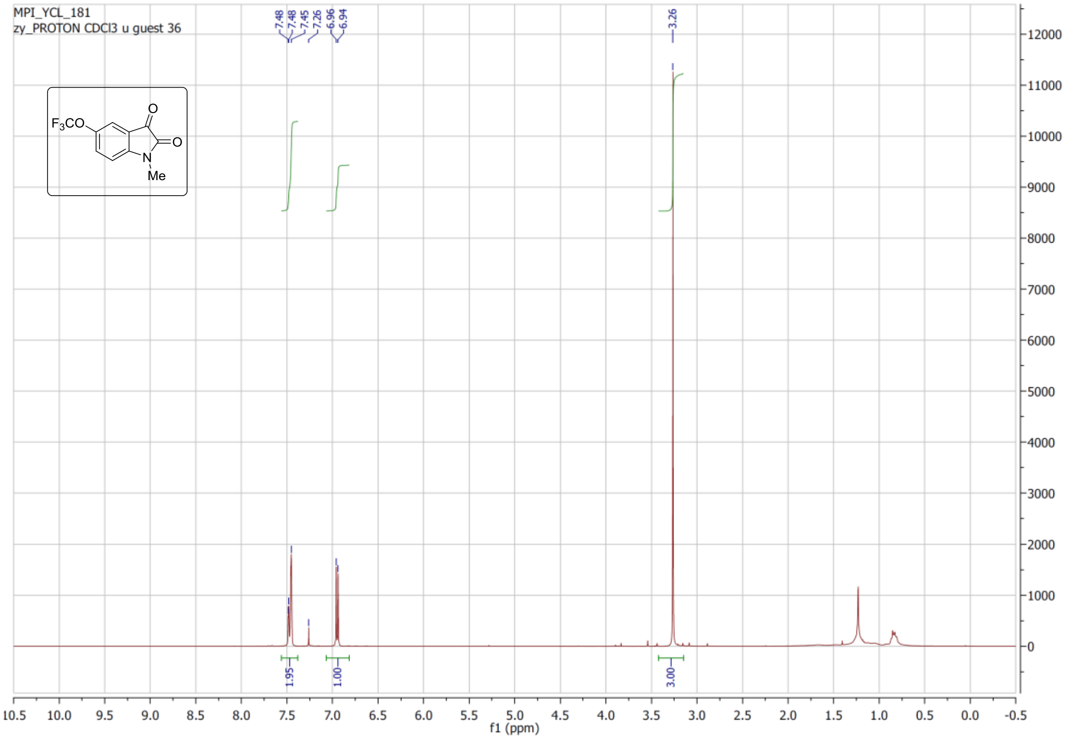

Supplementary Figure 8. ^{1}H NMR spectrum for **S8e**.

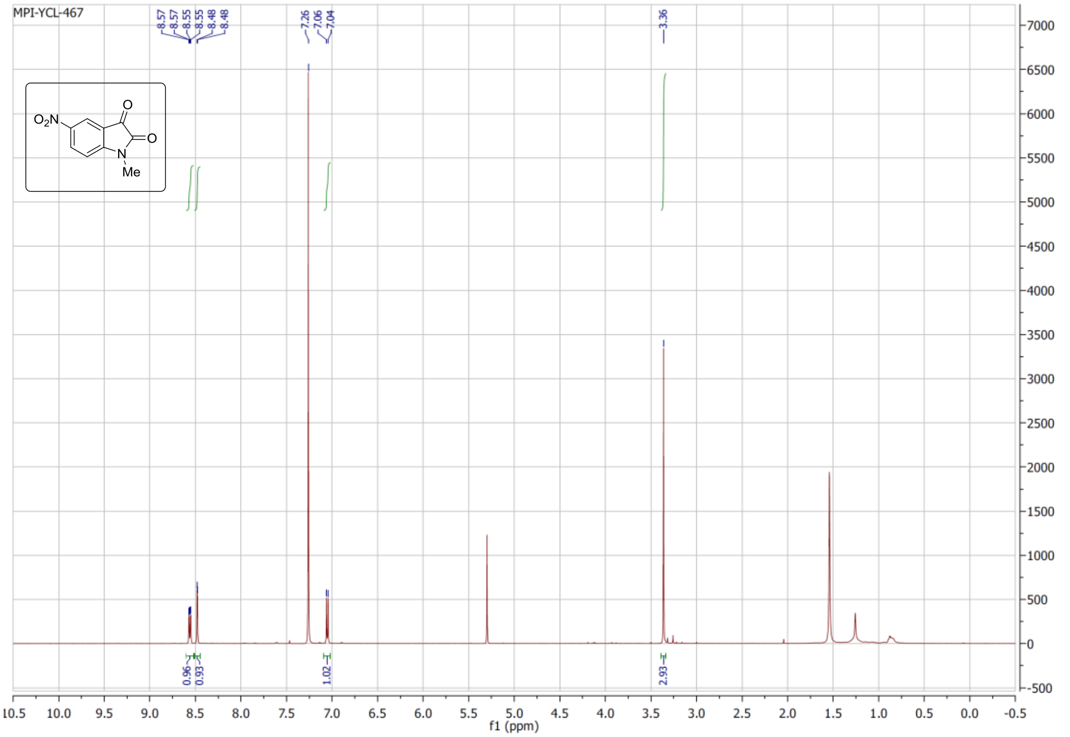

Supplementary Figure 9. ^{1}H NMR spectrum for **S8f**.

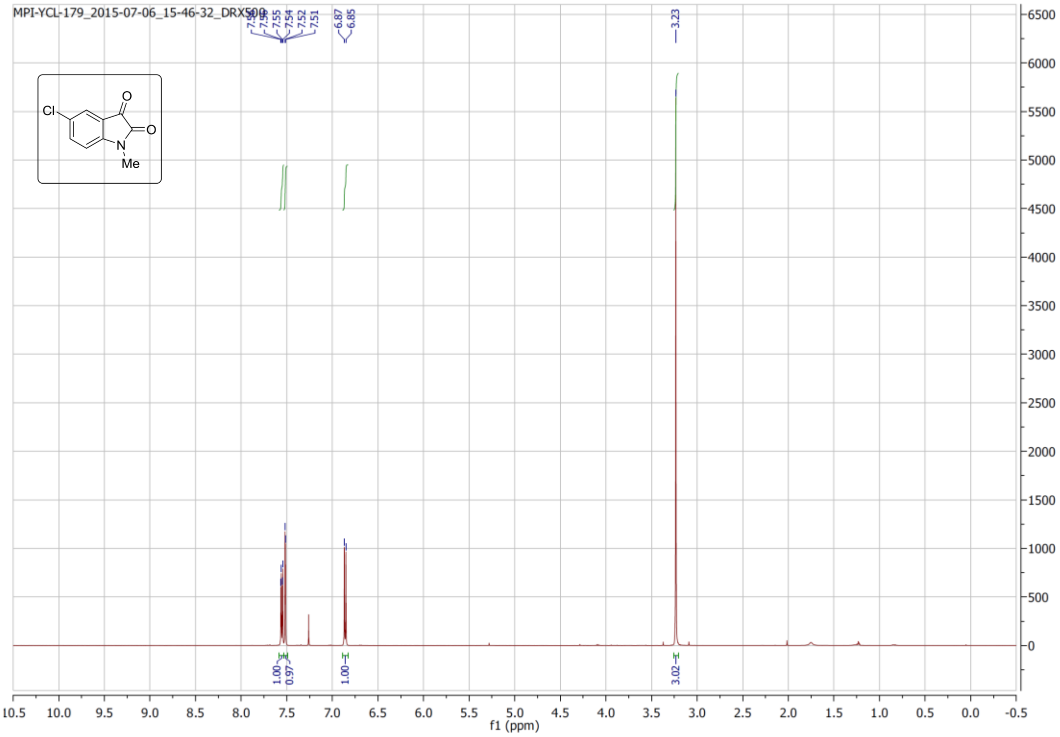

Supplementary Figure 10. ^{1}H NMR spectrum for **S8g**.

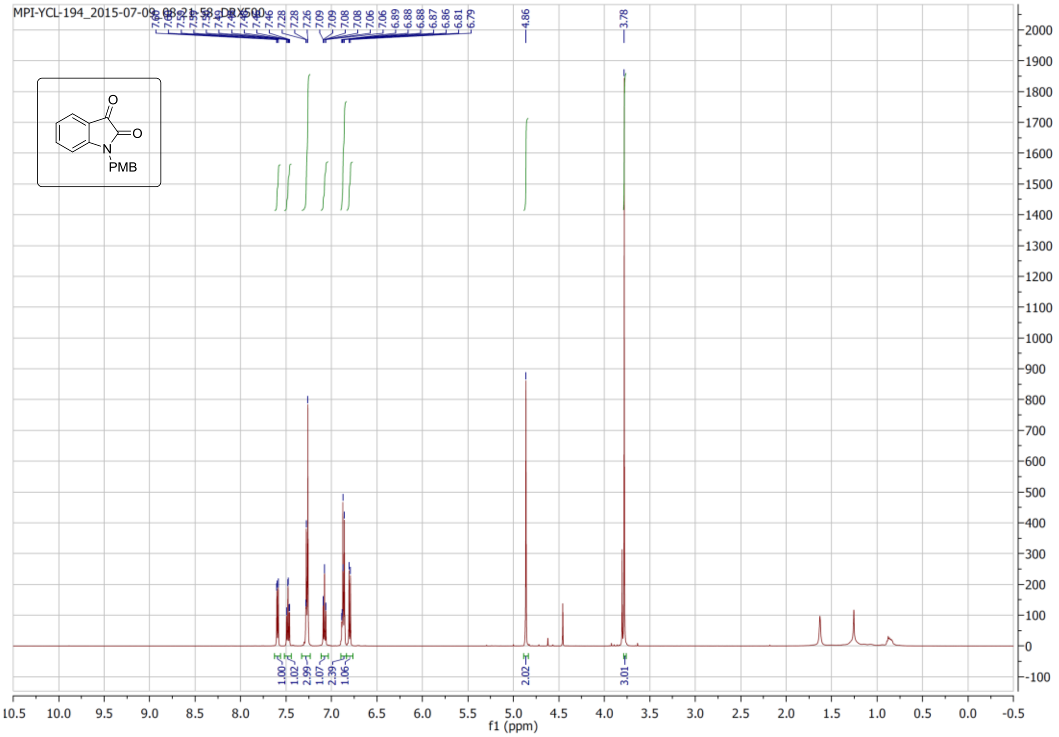

Supplementary Figure 11. ^{1}H NMR spectrum for **S8h**.

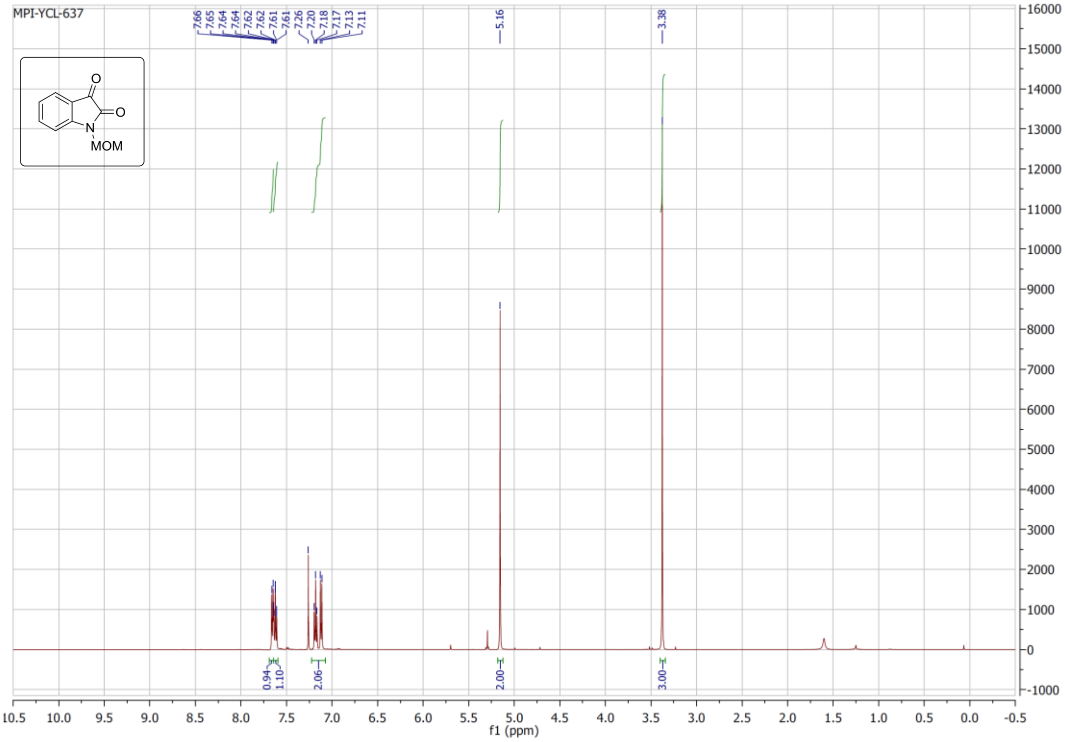

Supplementary Figure 12. ^{1}H NMR spectrum for **S8i**.

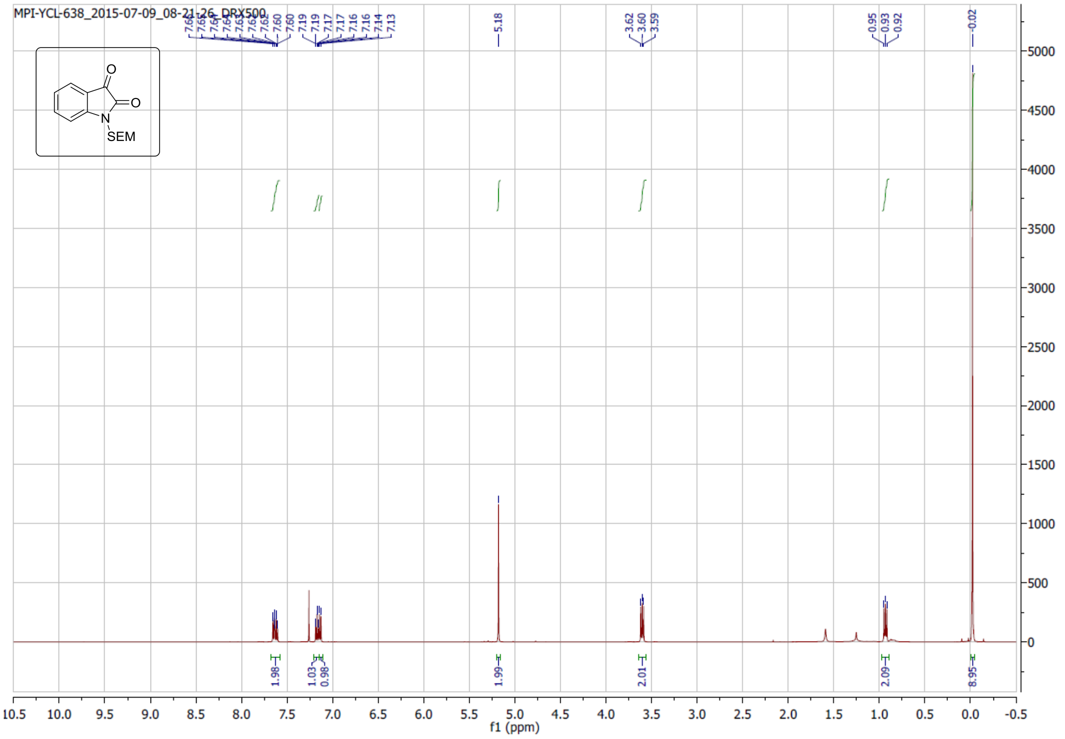

Supplementary Figure 13. ^{1}H NMR spectrum for **S8j**.

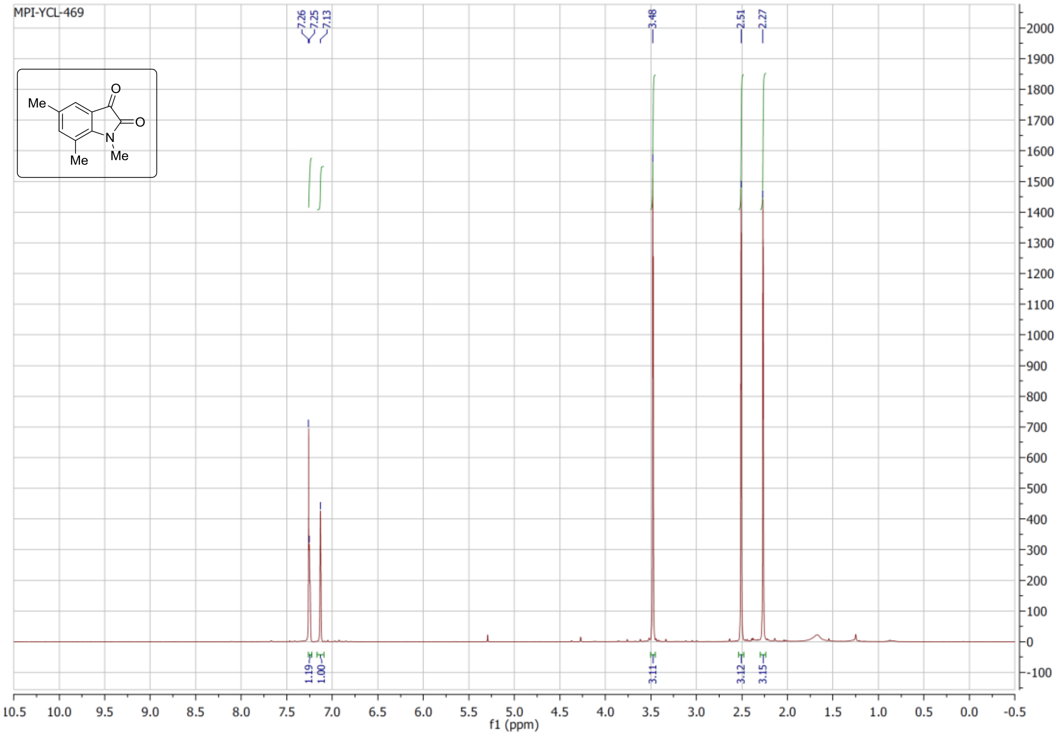

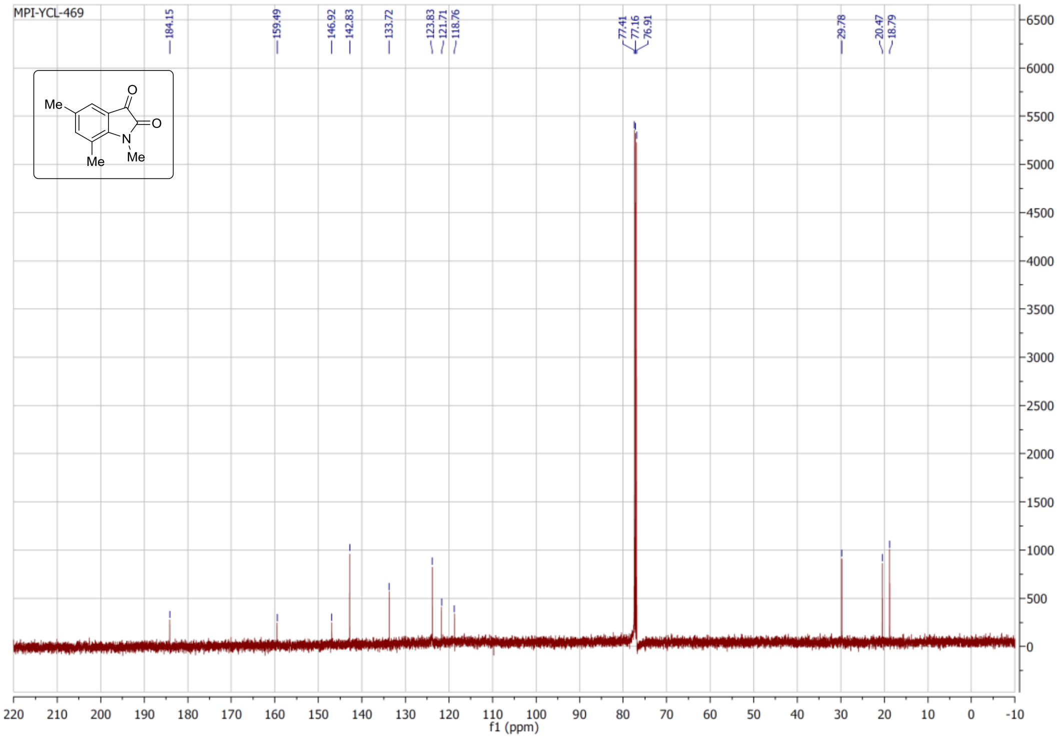

Supplementary Figure 14. ^{1}H and ^{13}C NMR spectra for **S8k**.

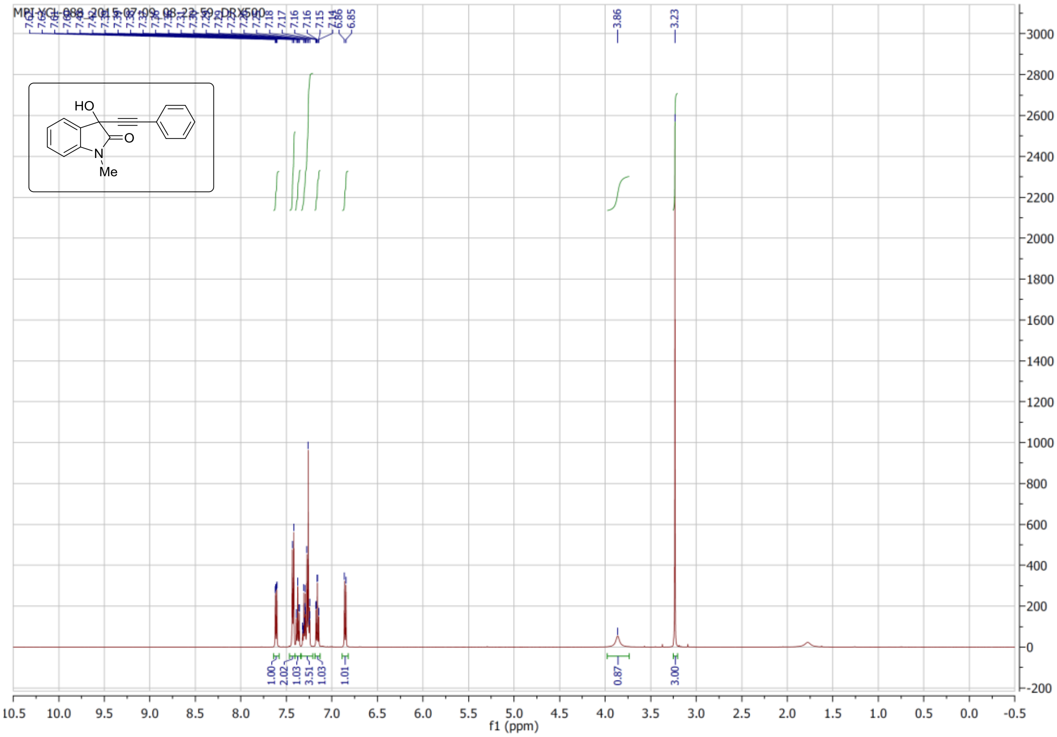

Supplementary Figure 15. ^{1}H NMR spectrum for **S9a.**

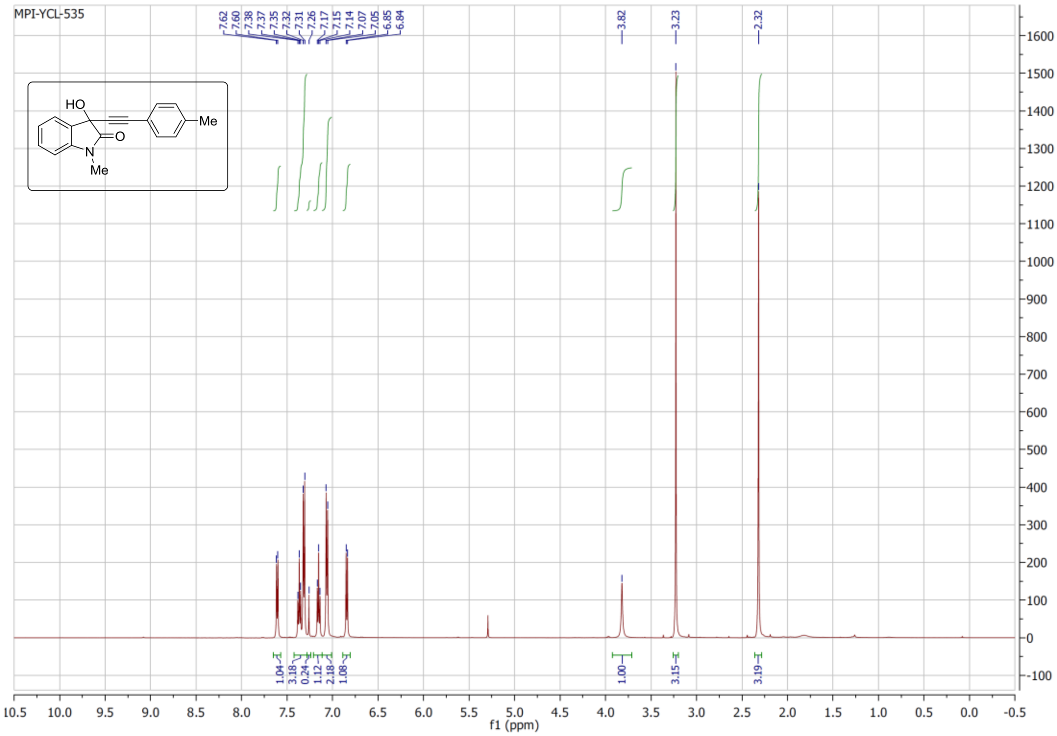

Supplementary Figure 16. ^{1}H NMR spectrum for **S9b.**

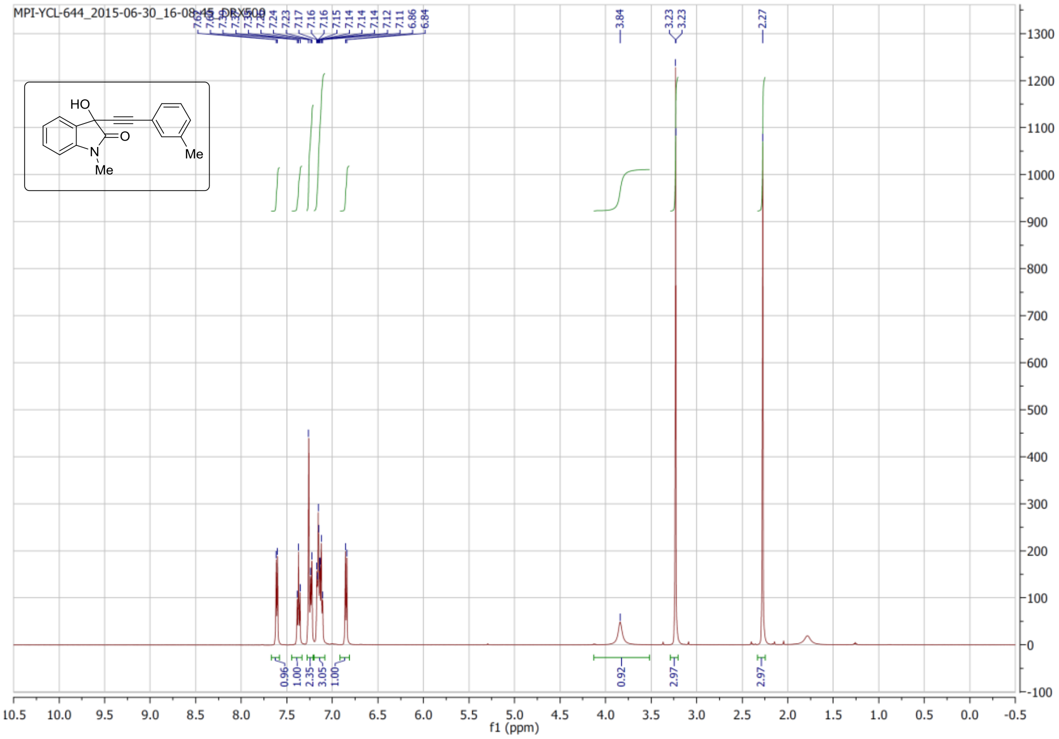

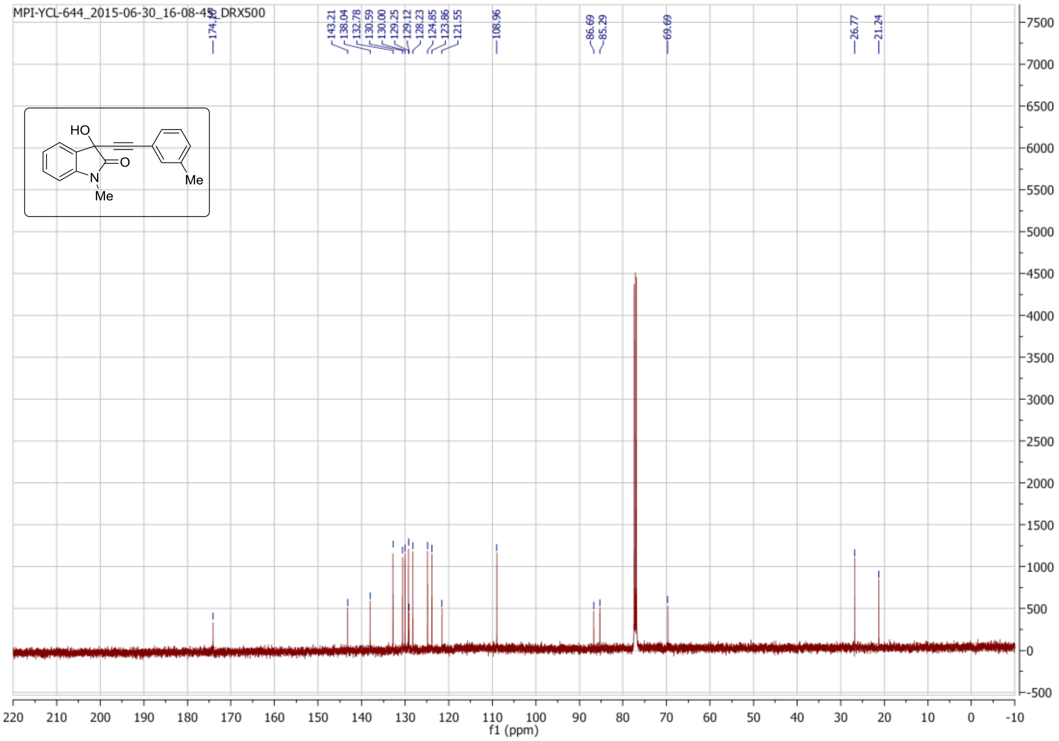

Supplementary Figure 17. ^{1}H and ^{13}C NMR spectra for **S9c.**

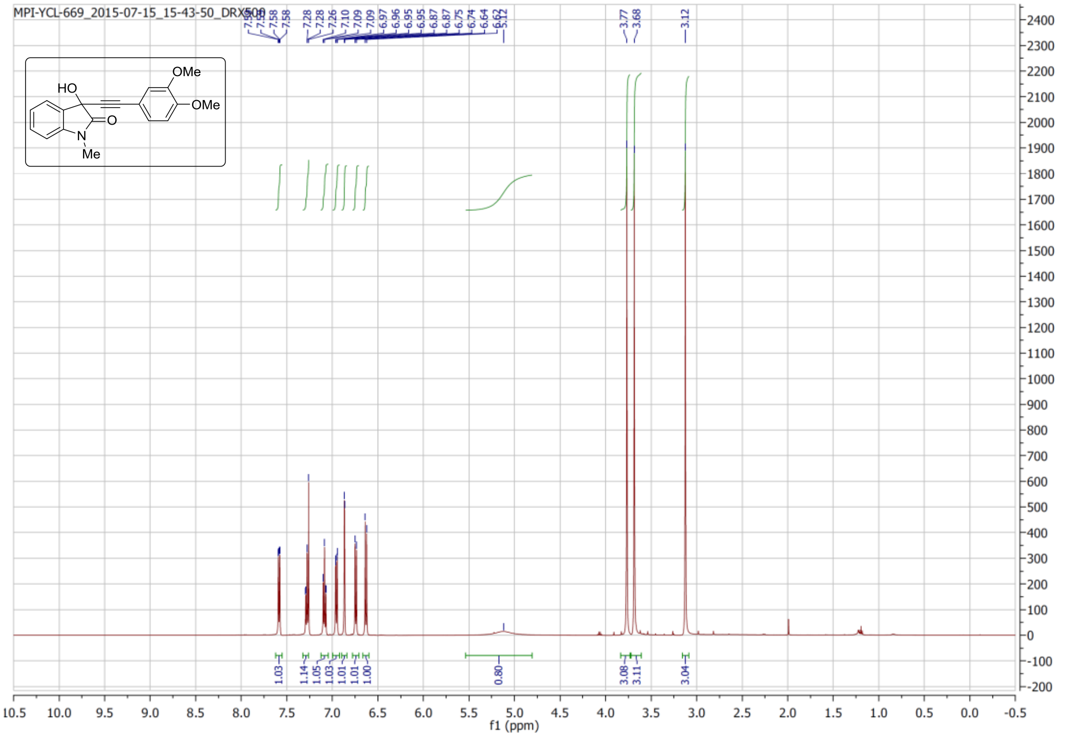

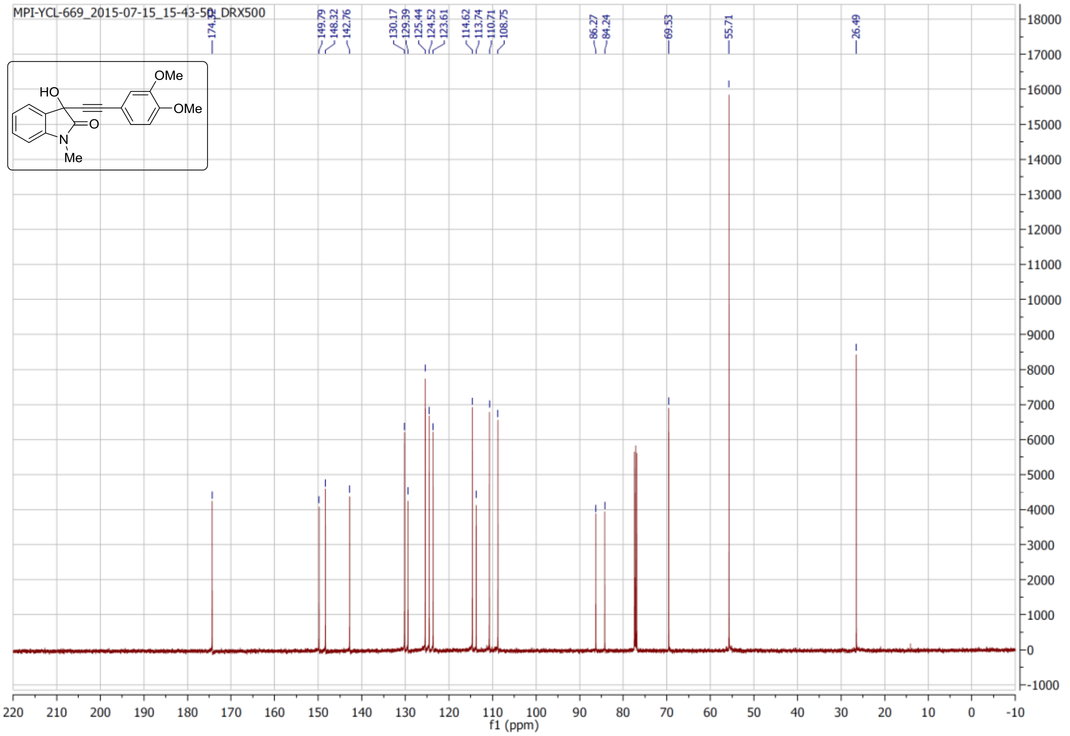

Supplementary Figure 18. ^{1}H and ^{13}C NMR spectra for **S9d.**

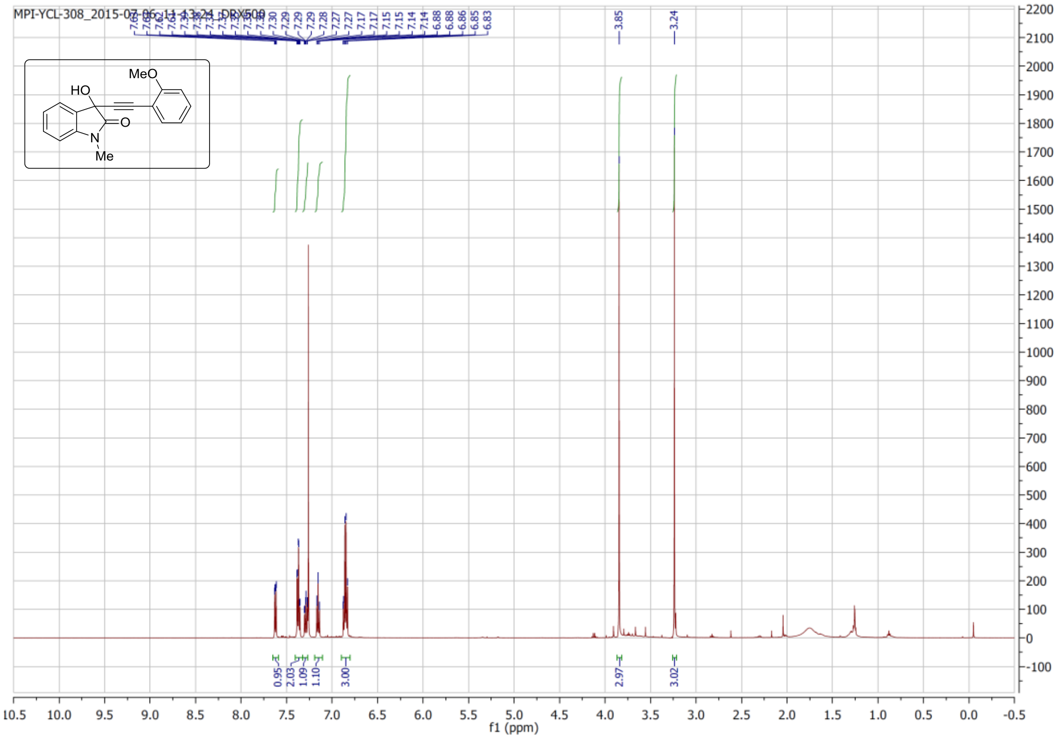

Supplementary Figure 19. ^{1}H NMR spectrum for **S9e.**

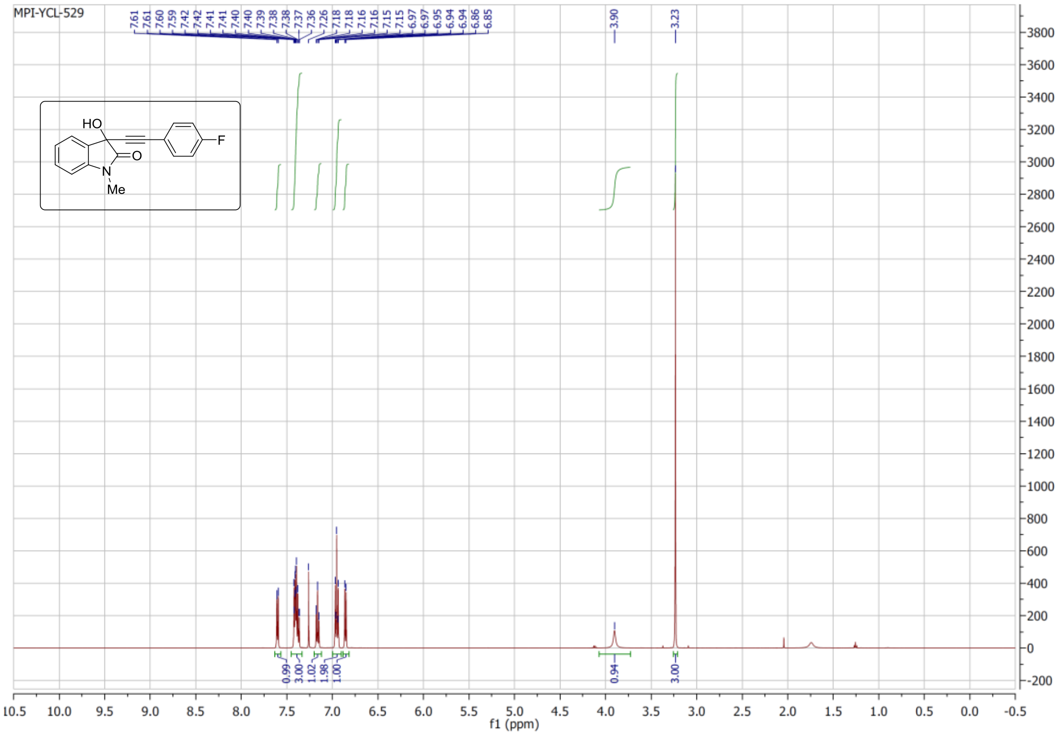

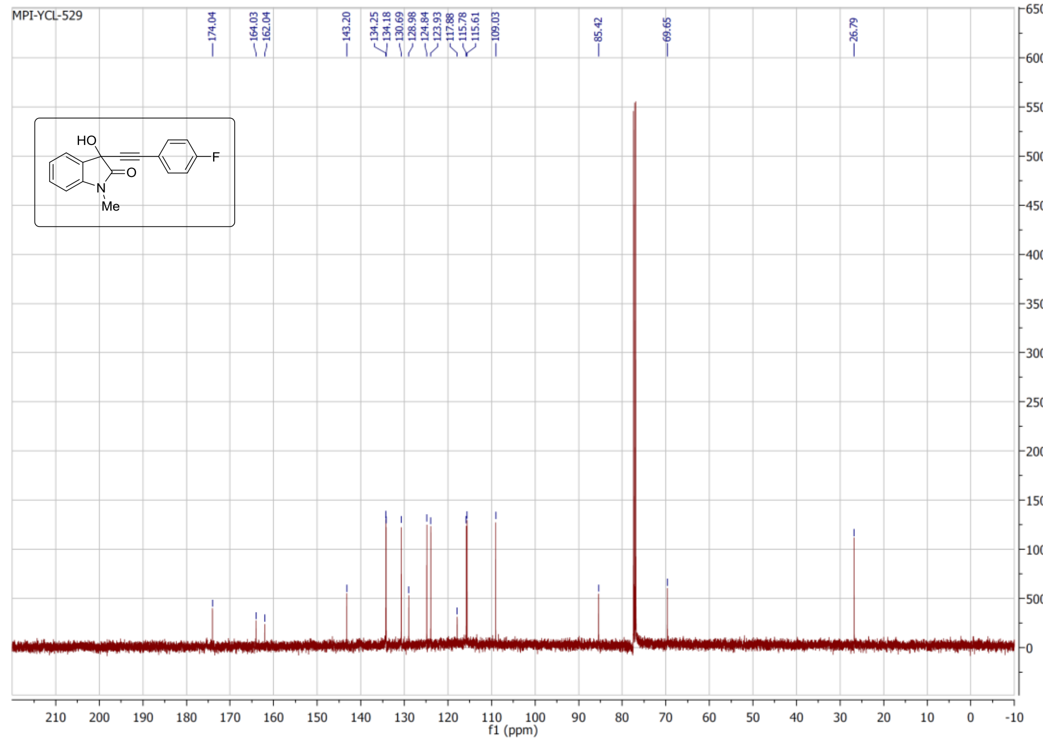

Supplementary Figure 20. ^{1}H and ^{13}C NMR spectra for **S9f.**

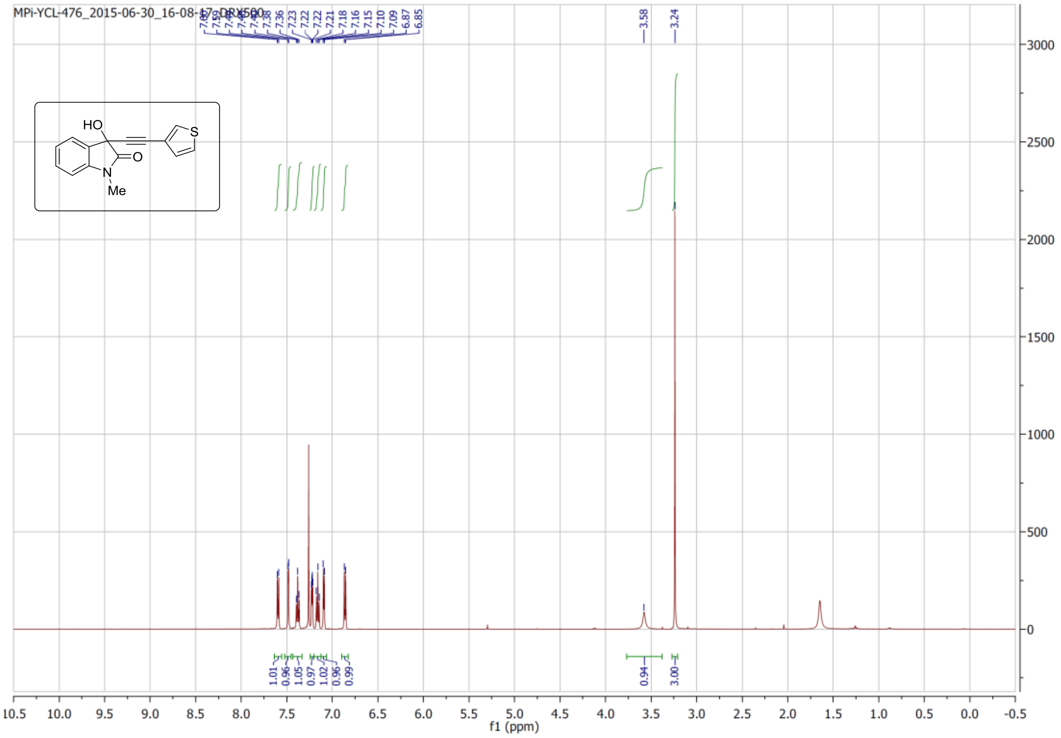

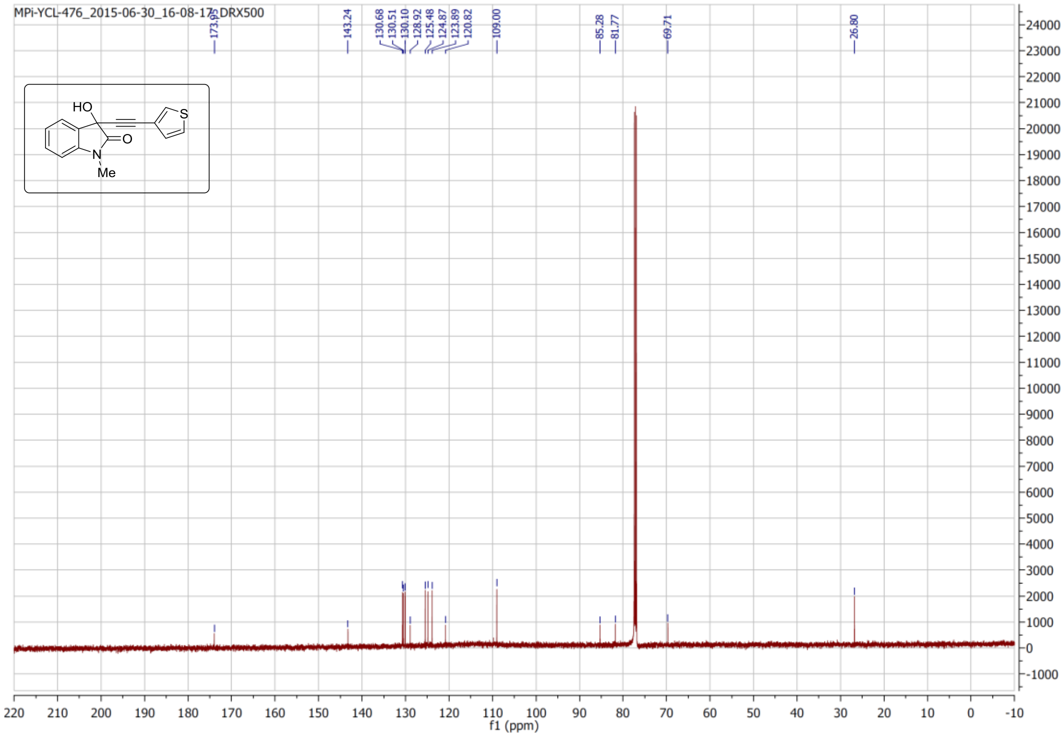

Supplementary Figure 21. ^{1}H and ^{13}C NMR spectra for **S9g.**

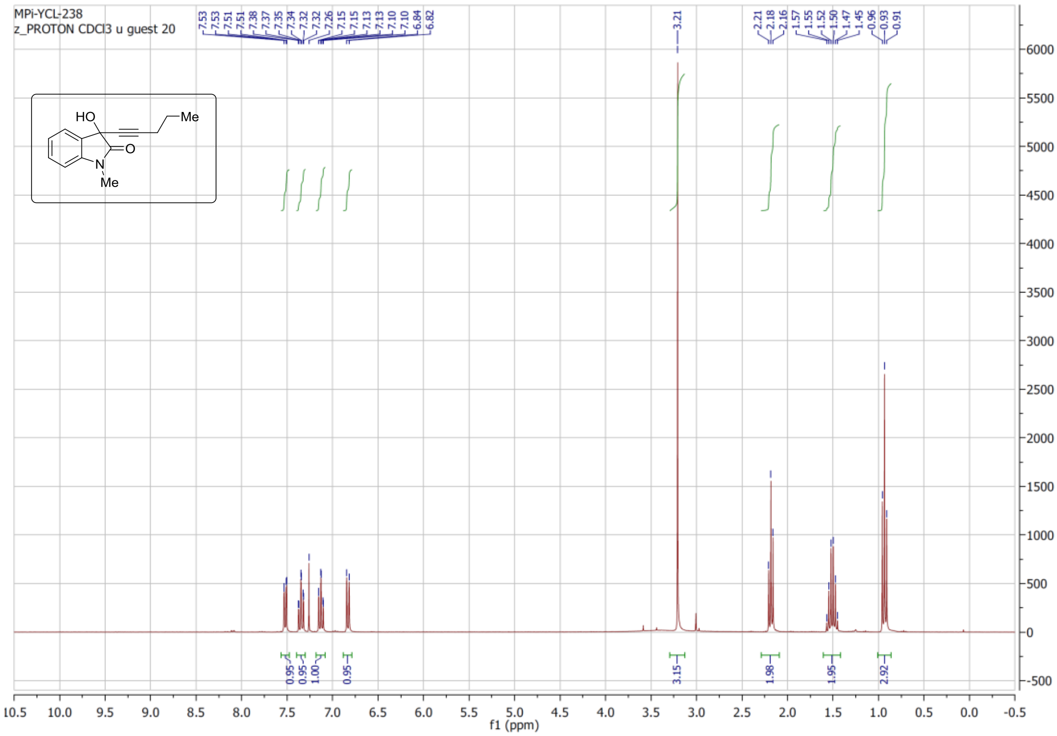

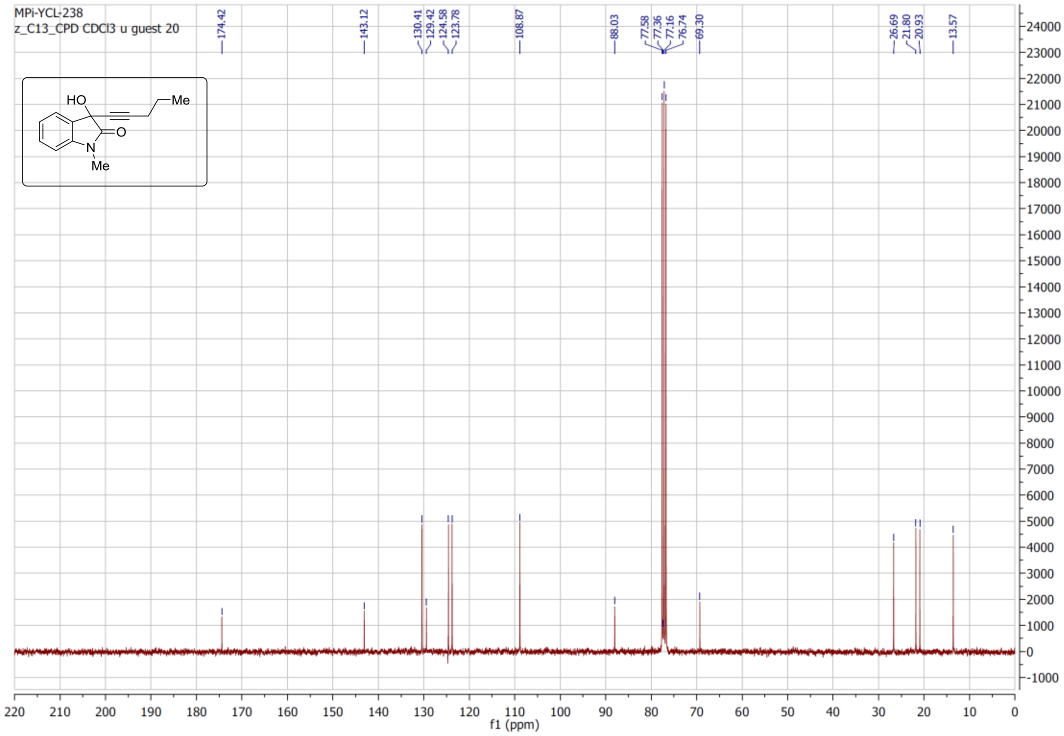

Supplementary Figure 22. ^{1}H and ^{13}C NMR spectra for **S9h.**

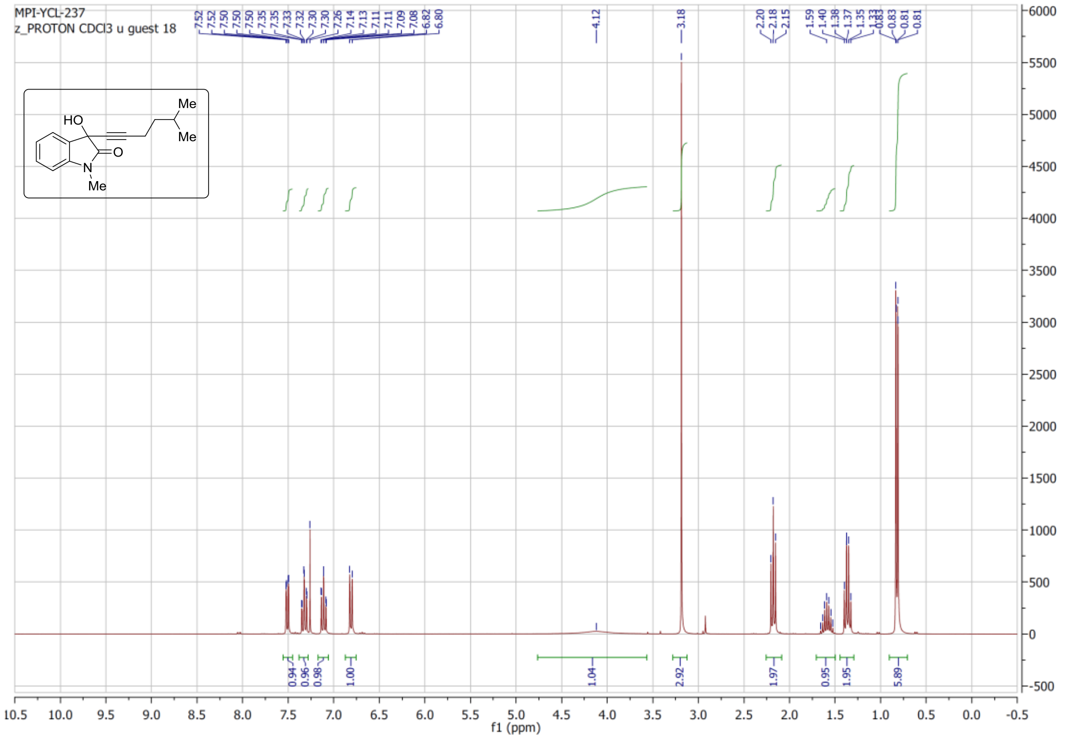

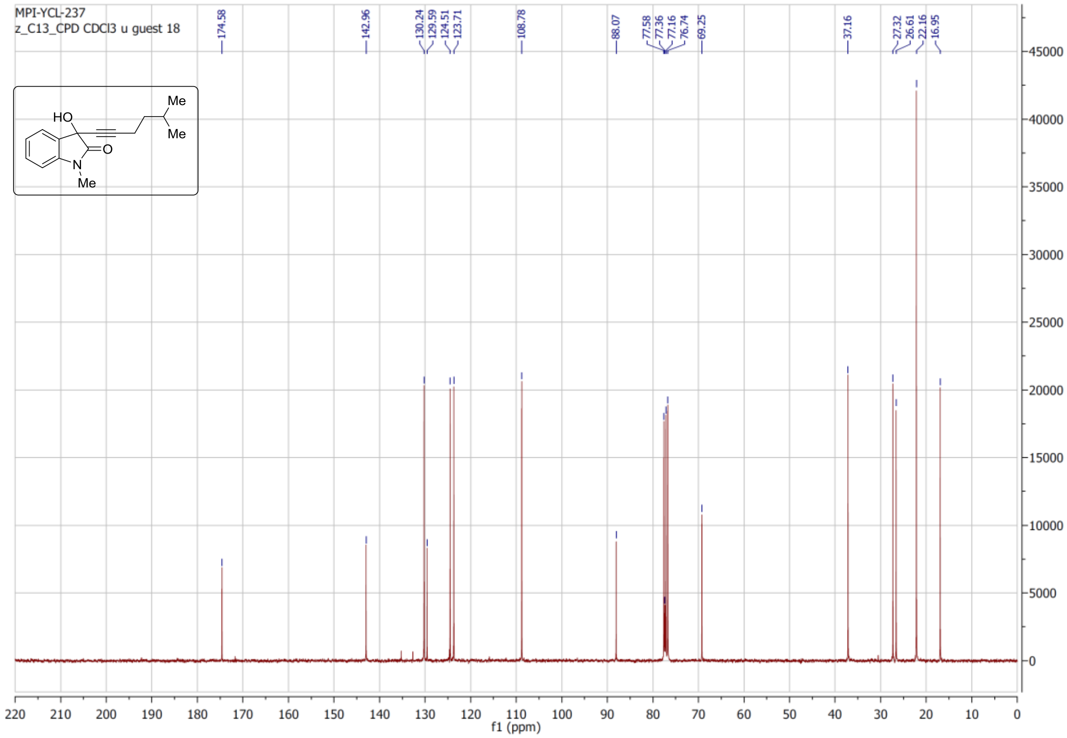

Supplementary Figure 23. ^{1}H and ^{13}C NMR spectra for **S9i.**

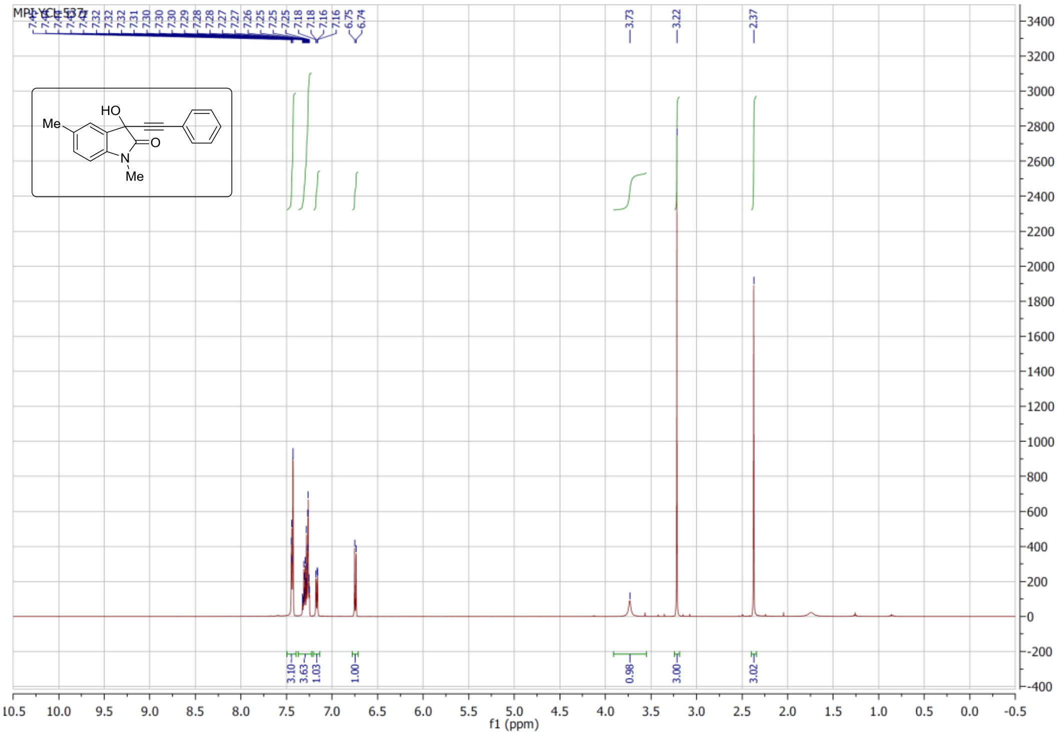

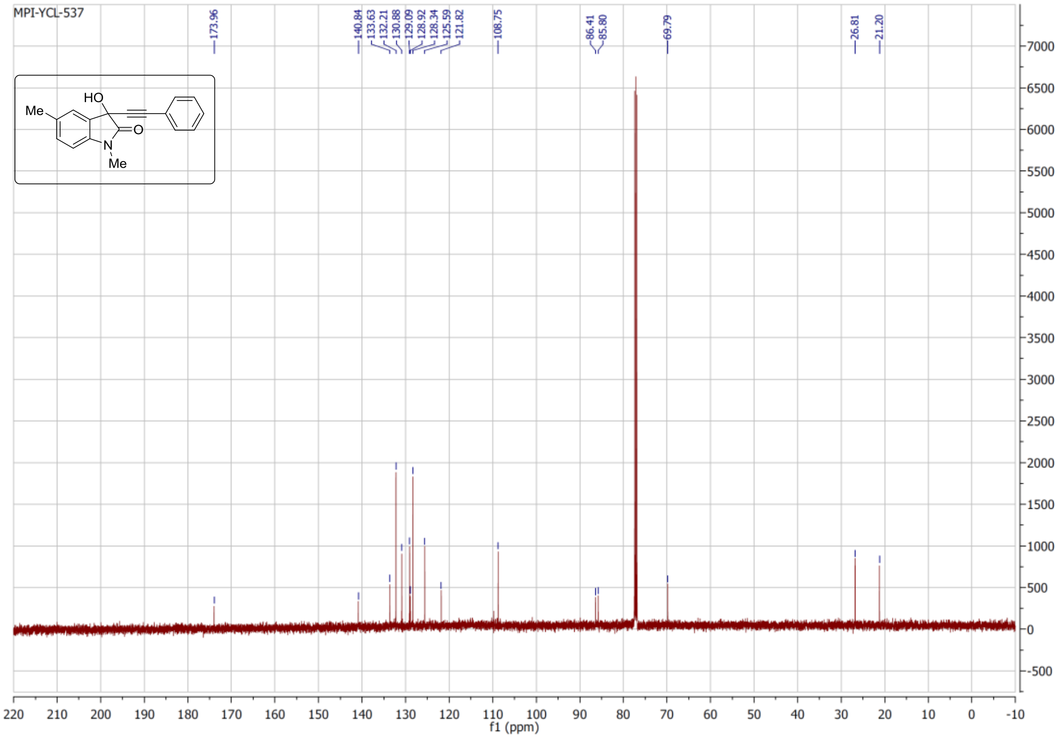

Supplementary Figure 24. ^{1}H and ^{13}C NMR spectra for **S9j.**

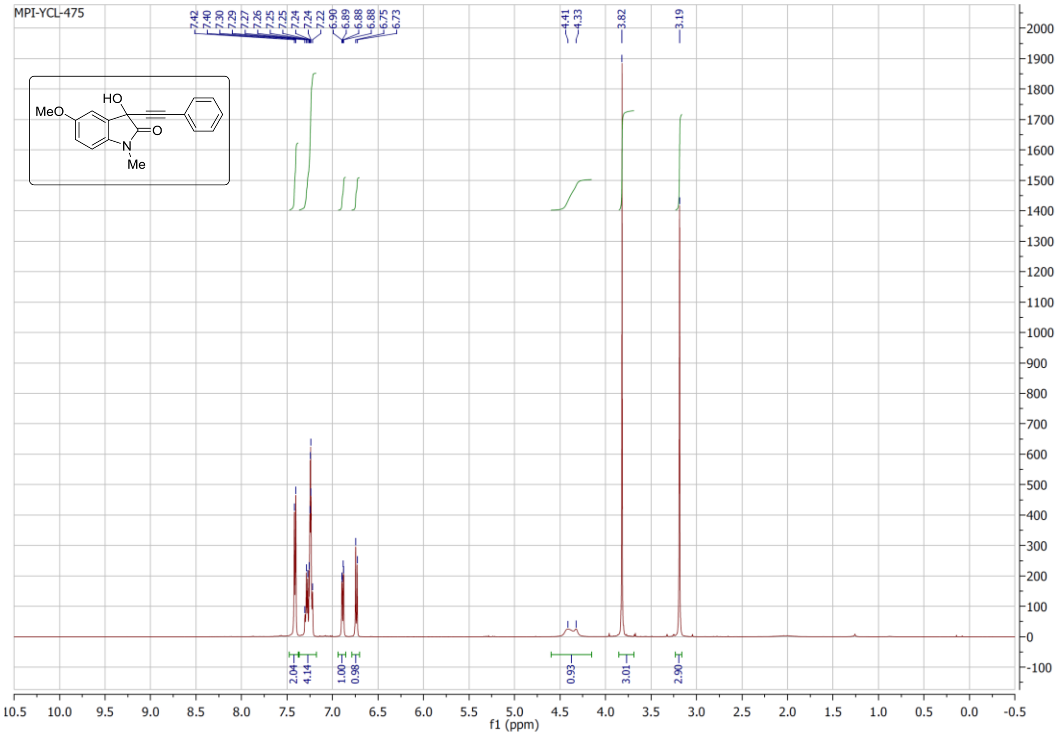

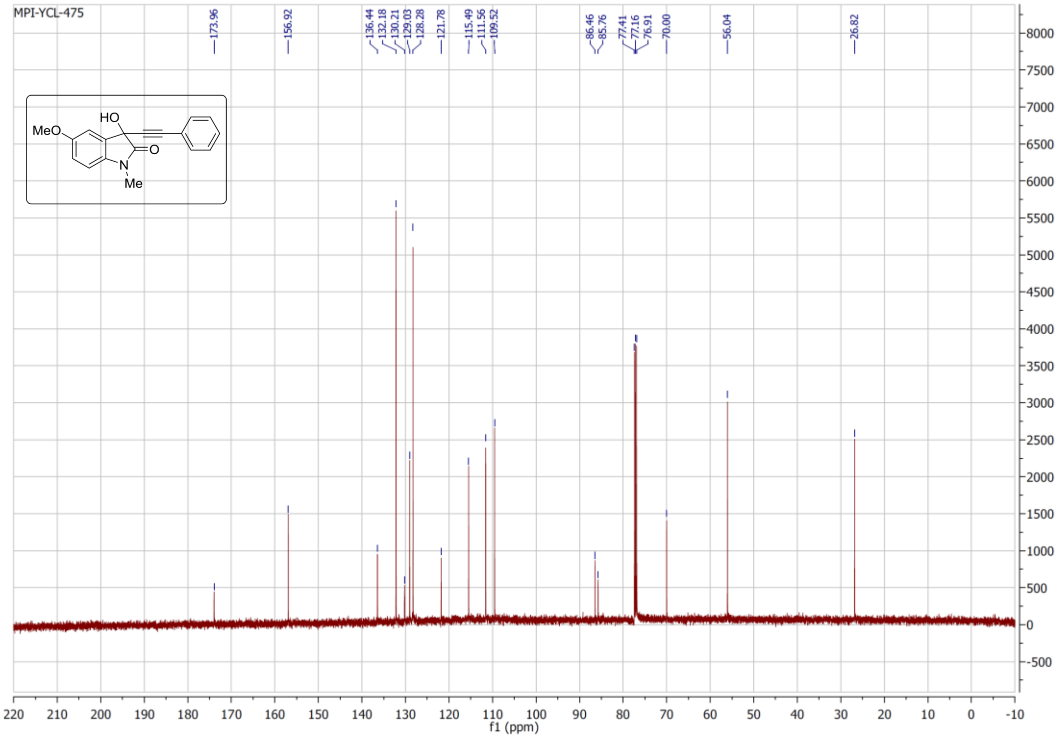

Supplementary Figure 25. ^{1}H and ^{13}C NMR spectra for **S9k.**

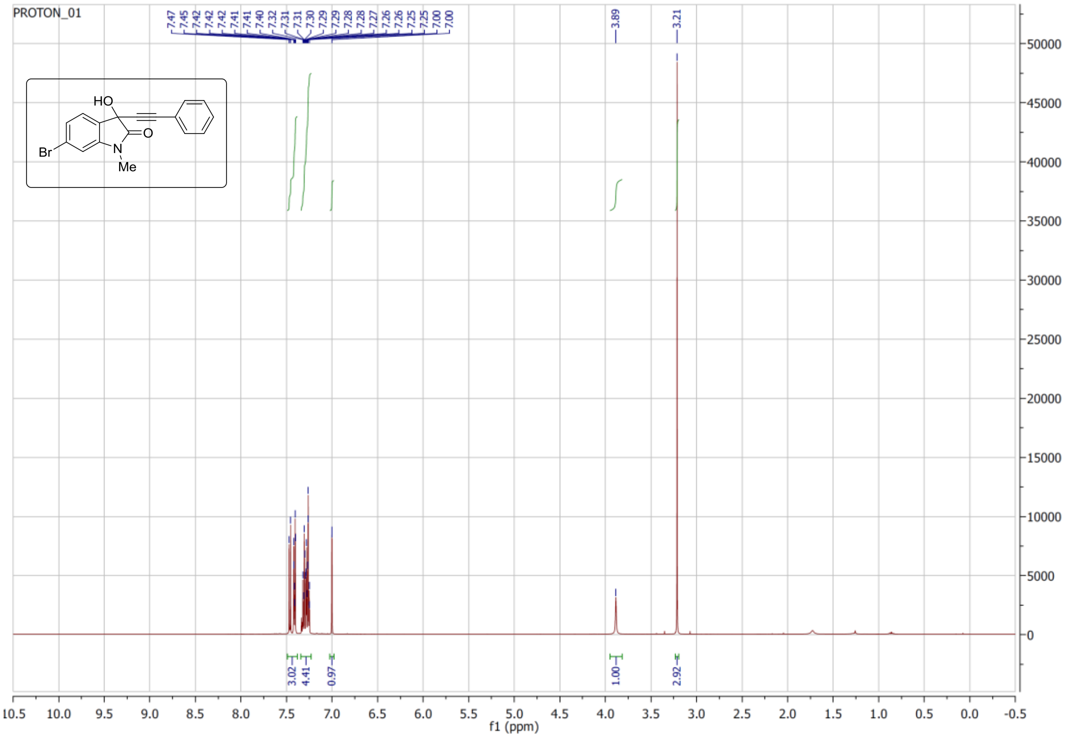

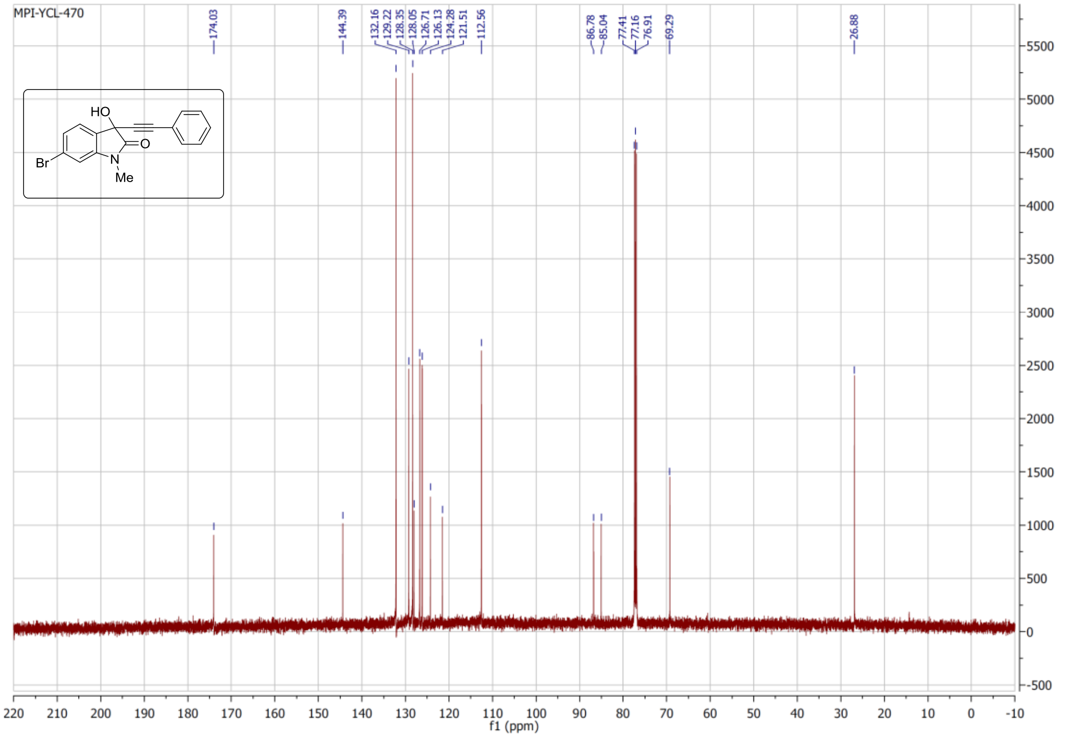

Supplementary Figure 26. ^{1}H and ^{13}C NMR spectra for **S9l.**

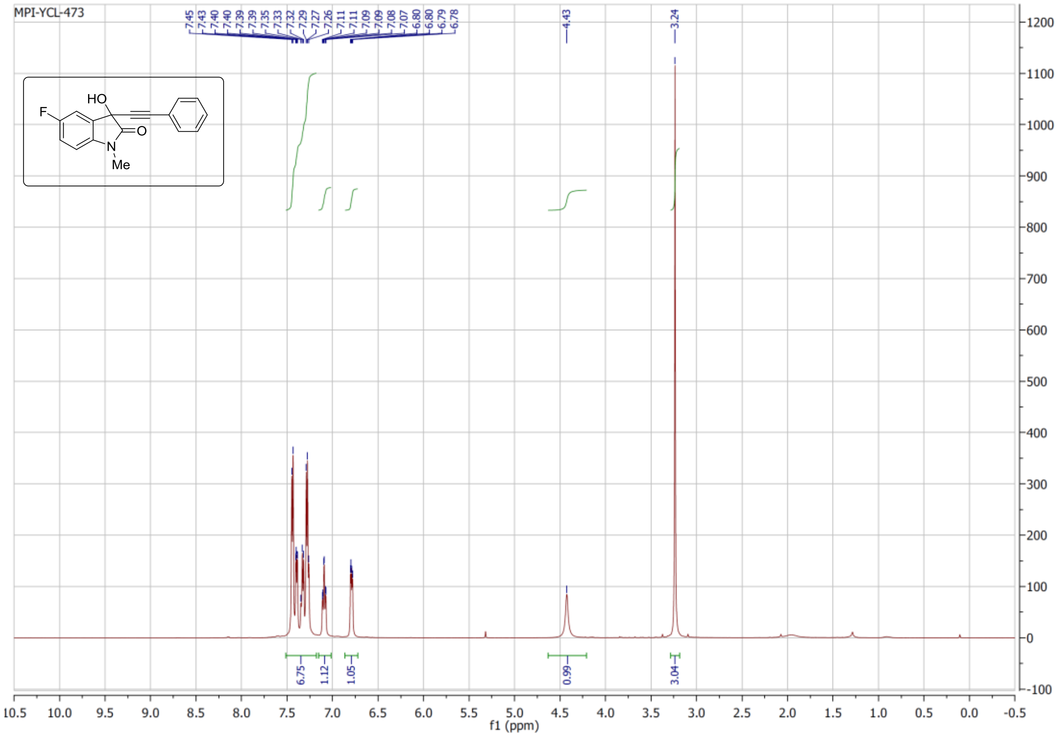

Supplementary Figure 27. ^{1}H NMR spectrum for **S9m.**

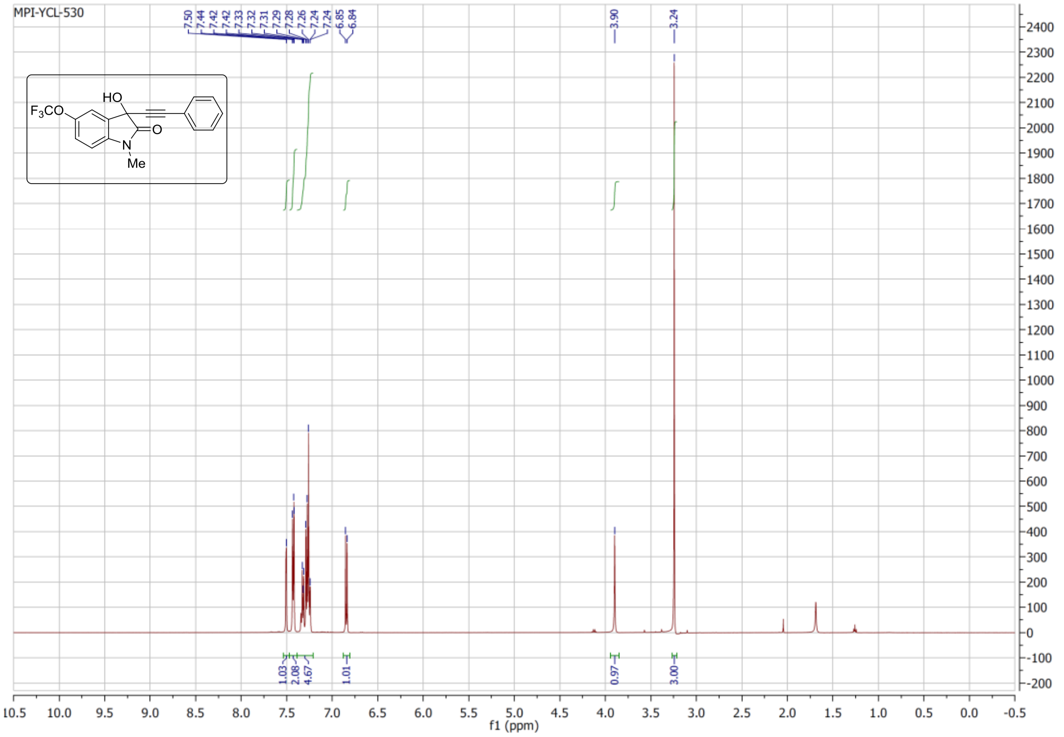

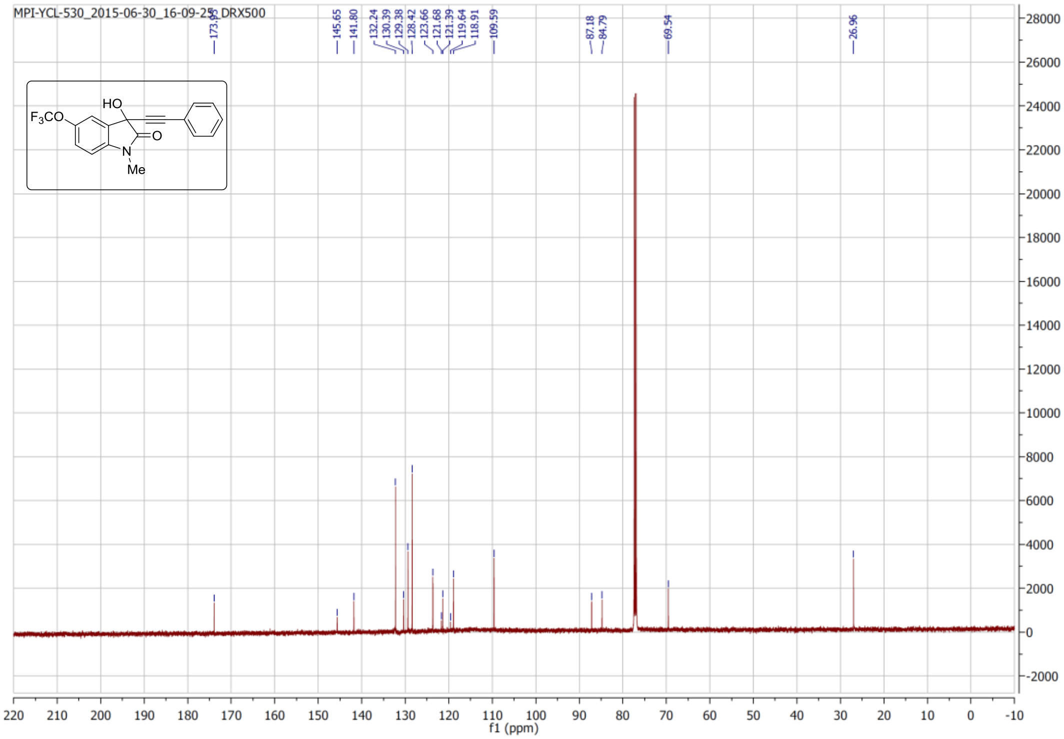

Supplementary Figure 28. ^{1}H and ^{13}C NMR spectra for **S9n.**

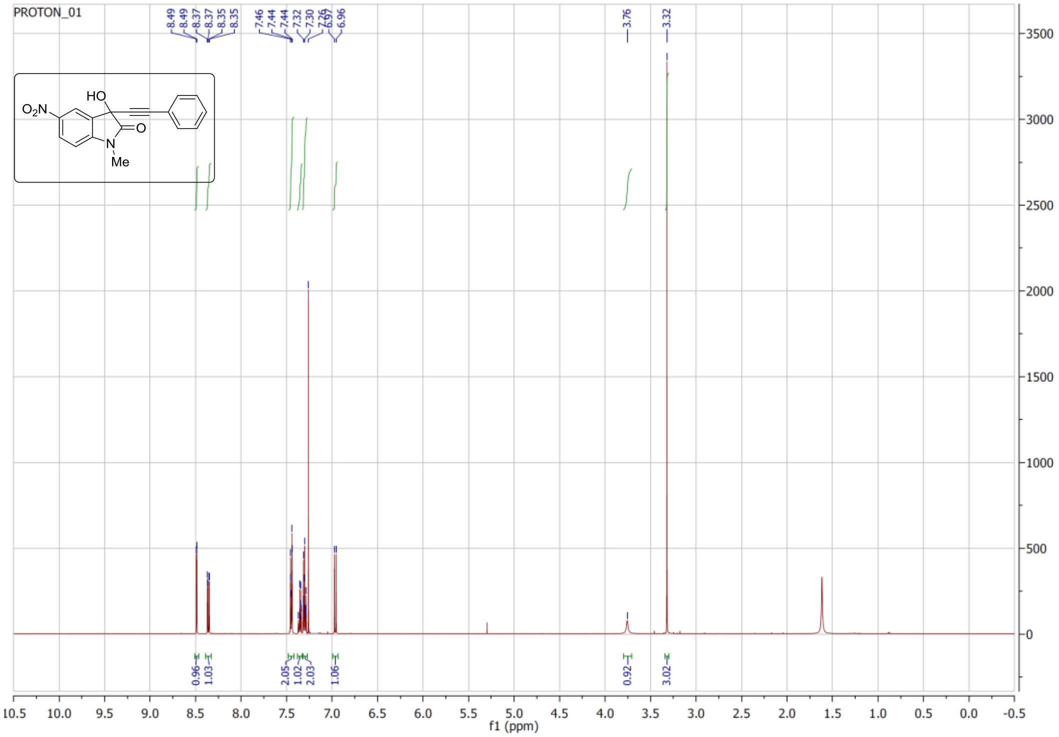

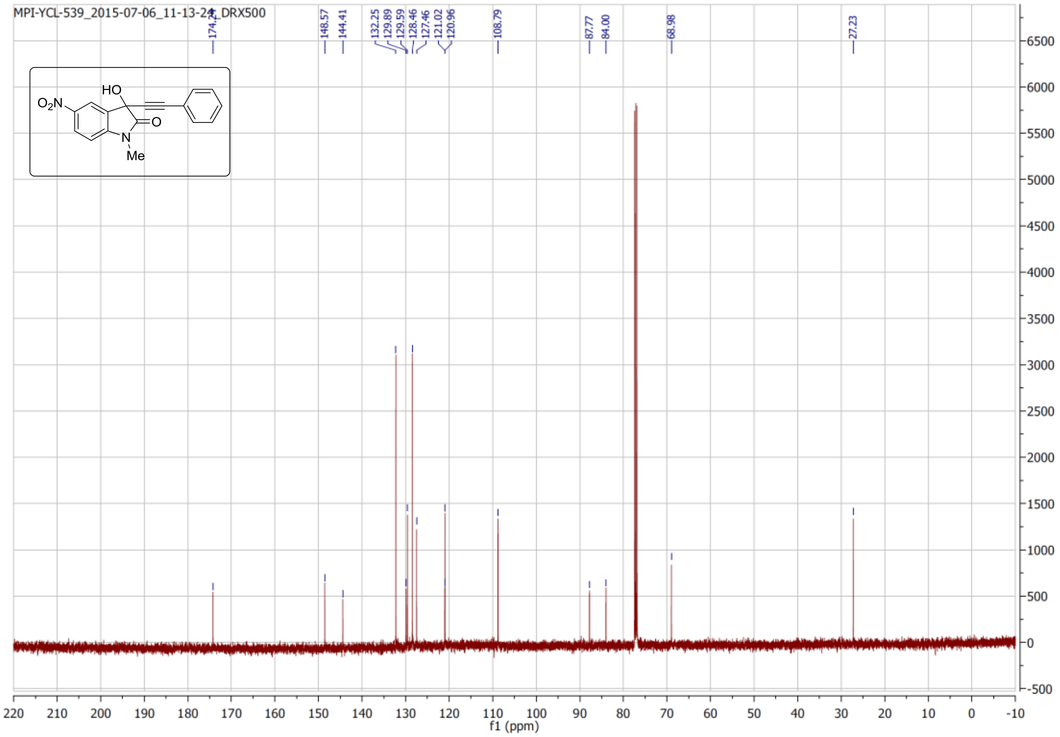

Supplementary Figure 29. ^{1}H and ^{13}C NMR spectra for **S9o.**

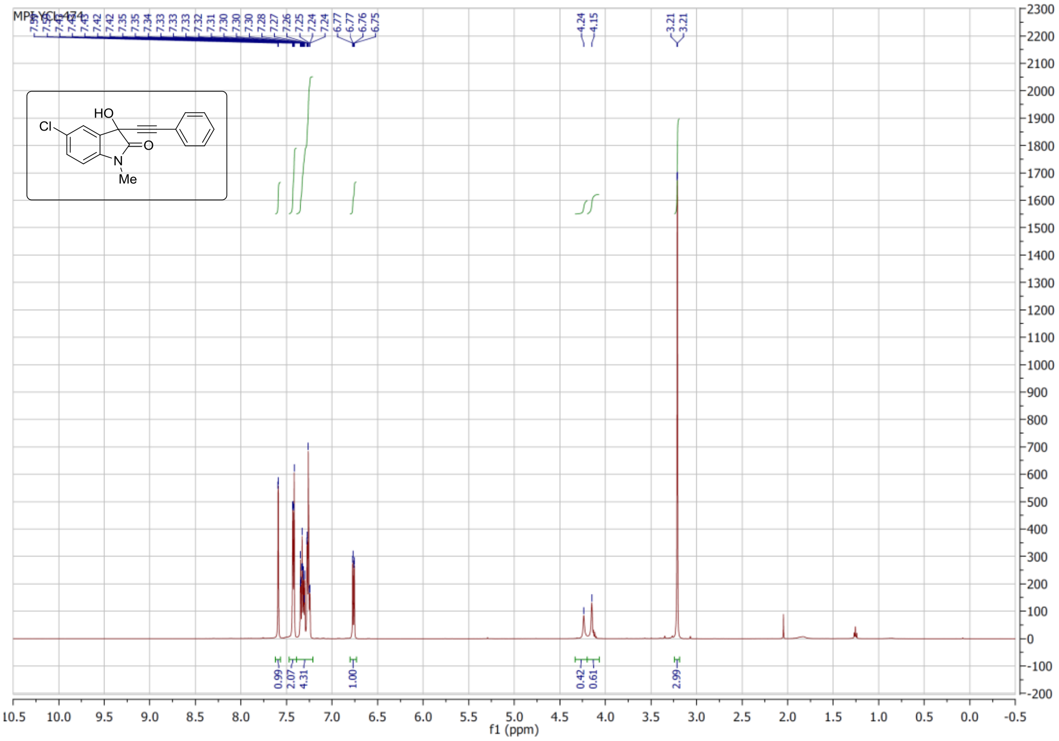

Supplementary Figure 30. ^{1}H NMR spectrum for **S9p.**

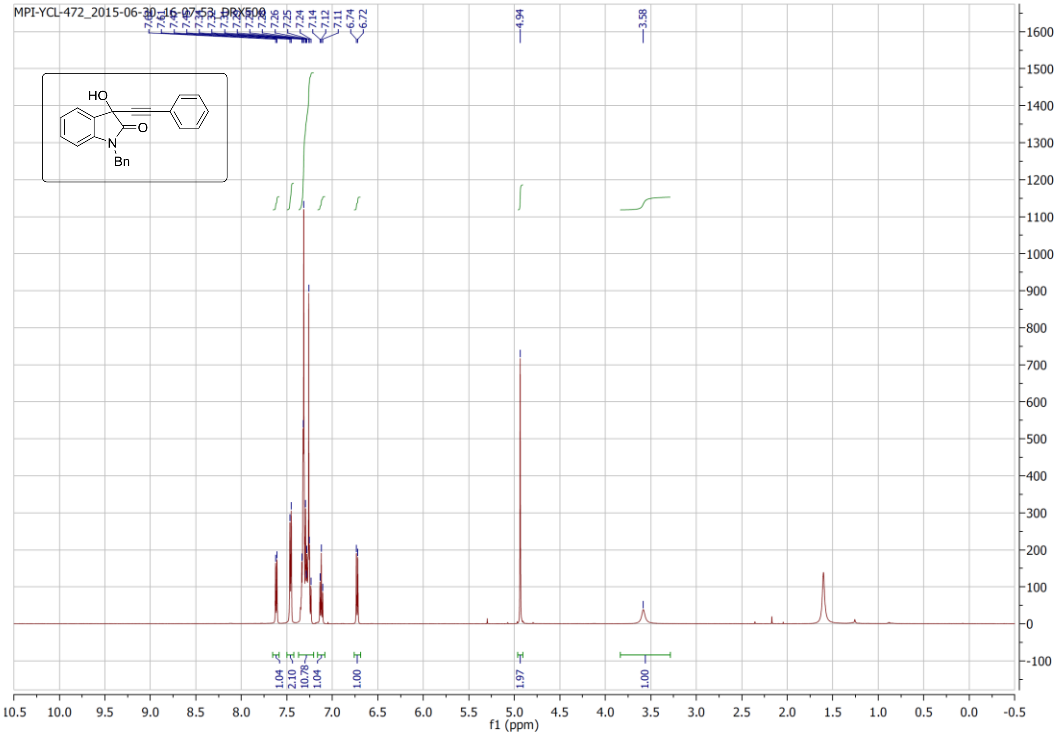

Supplementary Figure 31. ^{1}H NMR spectrum for **S9q.**

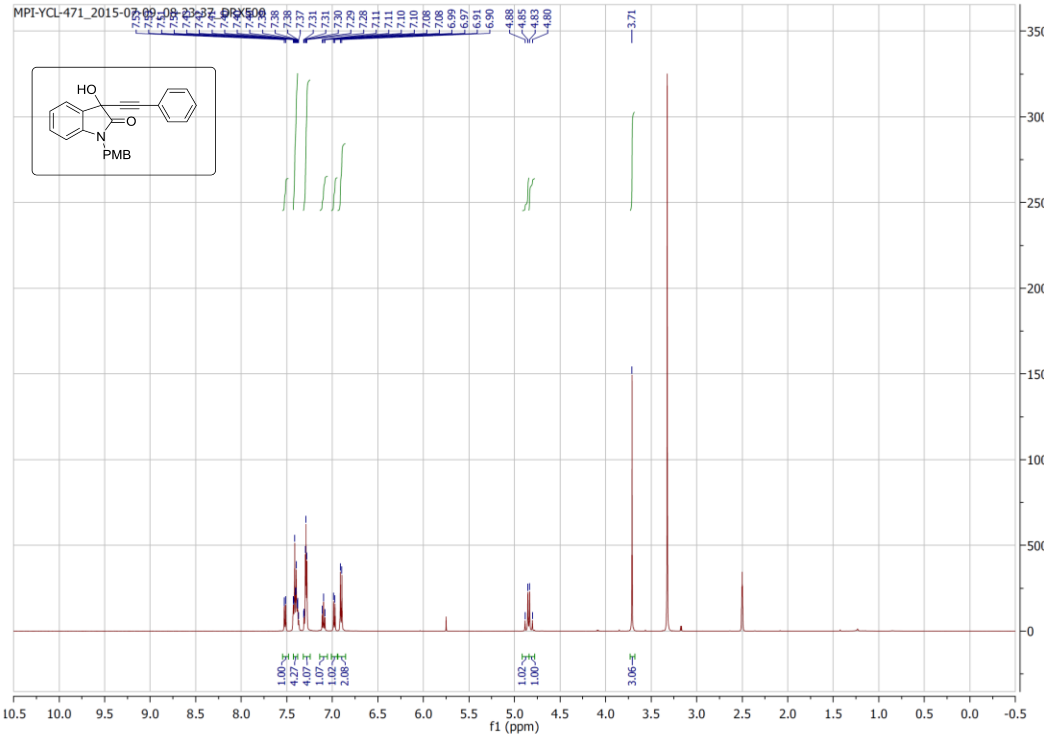

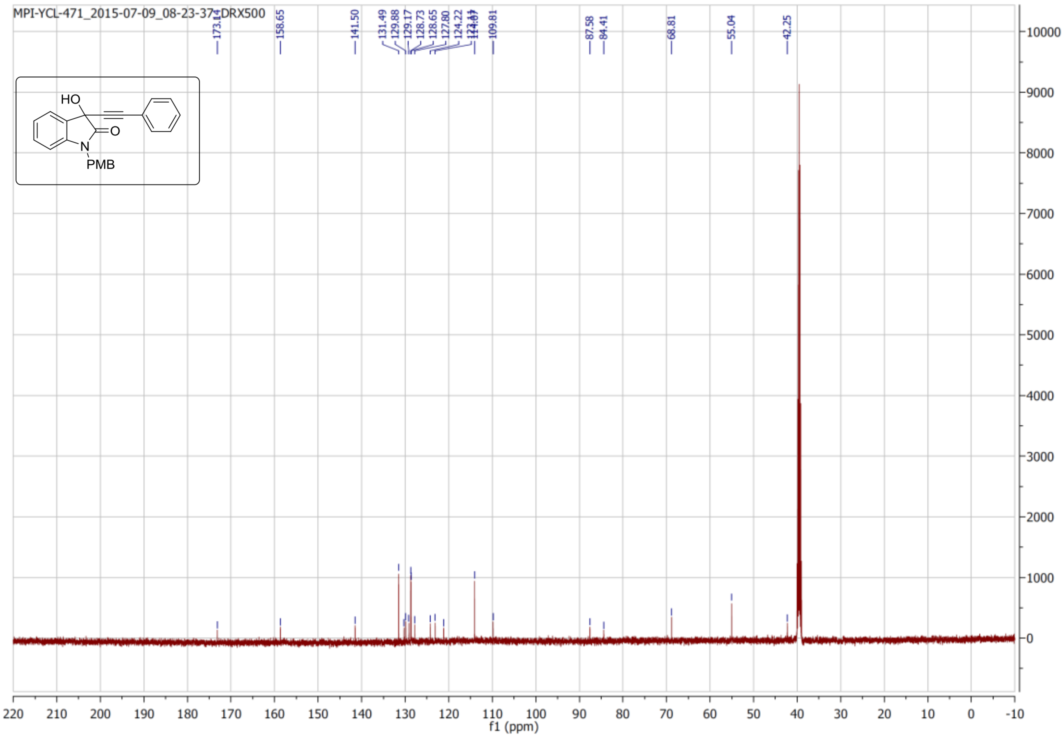

Supplementary Figure 32. ^{1}H and ^{13}C NMR spectra for **S9r.**

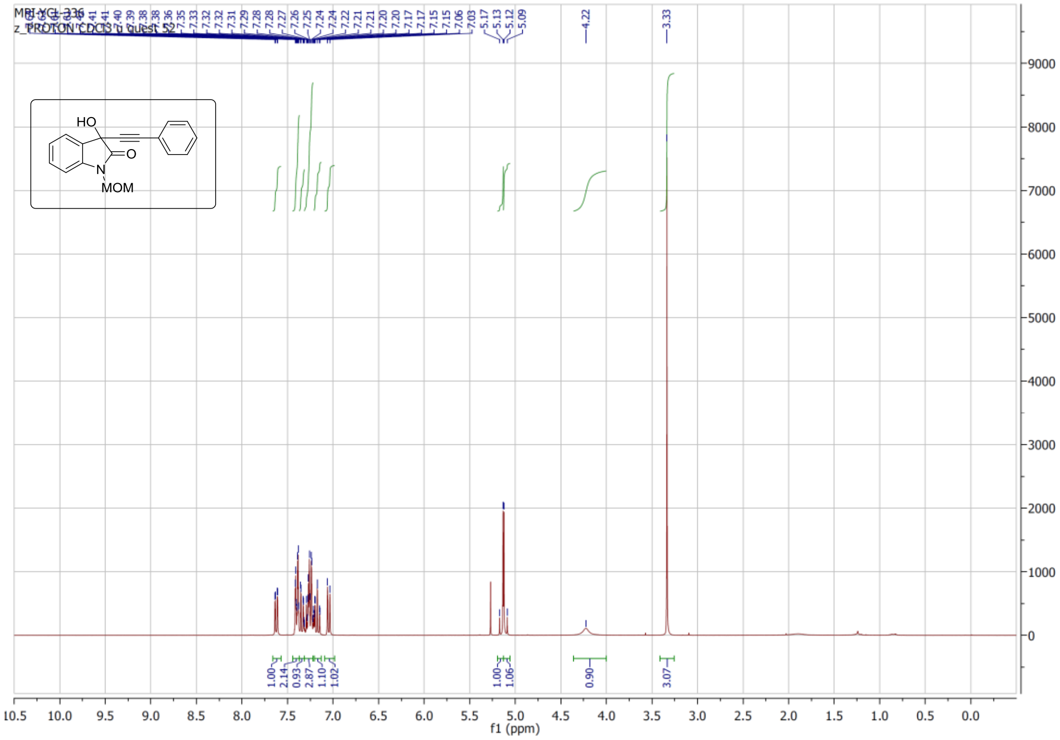

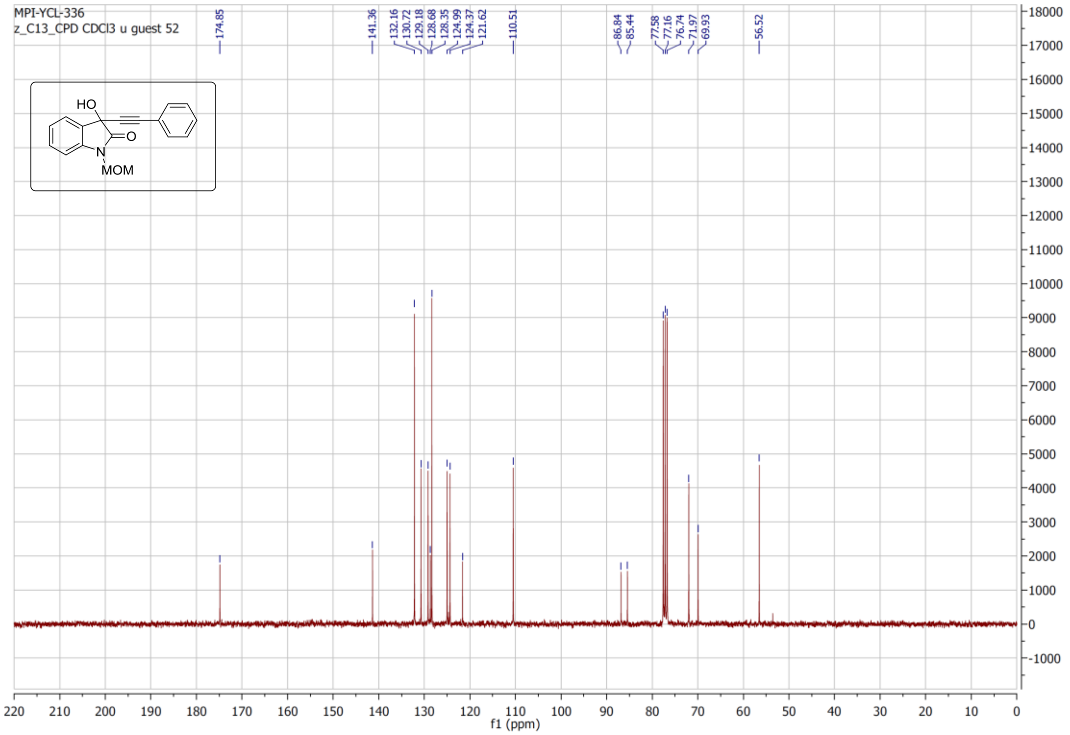

Supplementary Figure 33. ^{1}H and ^{13}C NMR spectra for **S9s.**

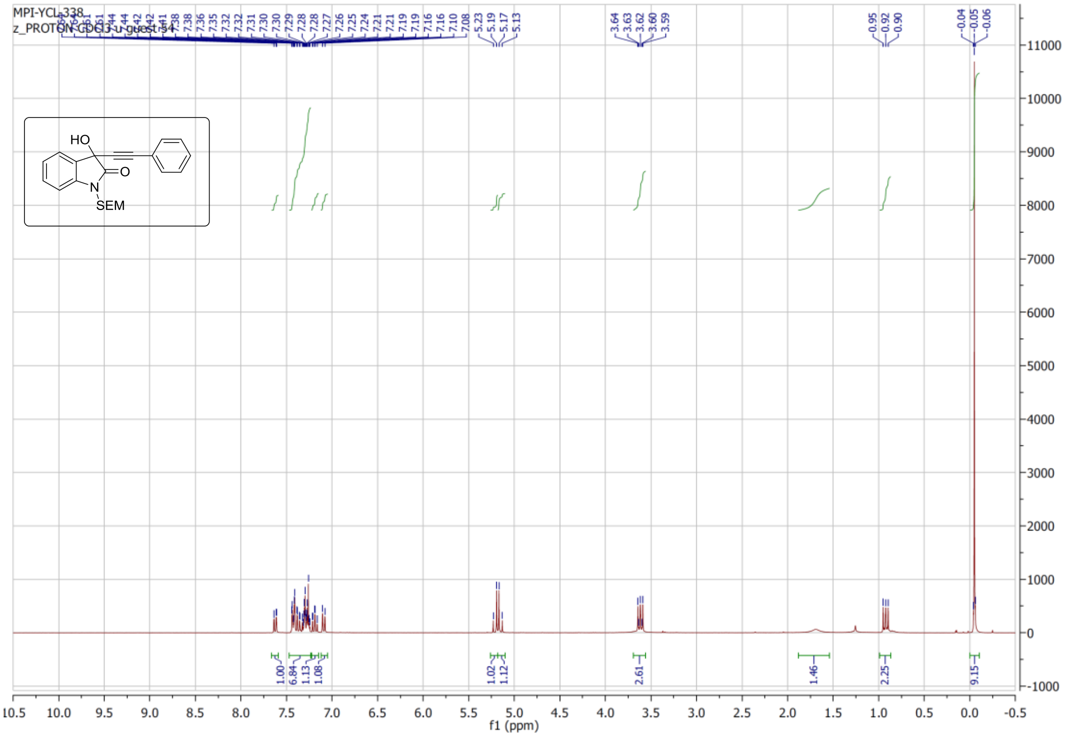

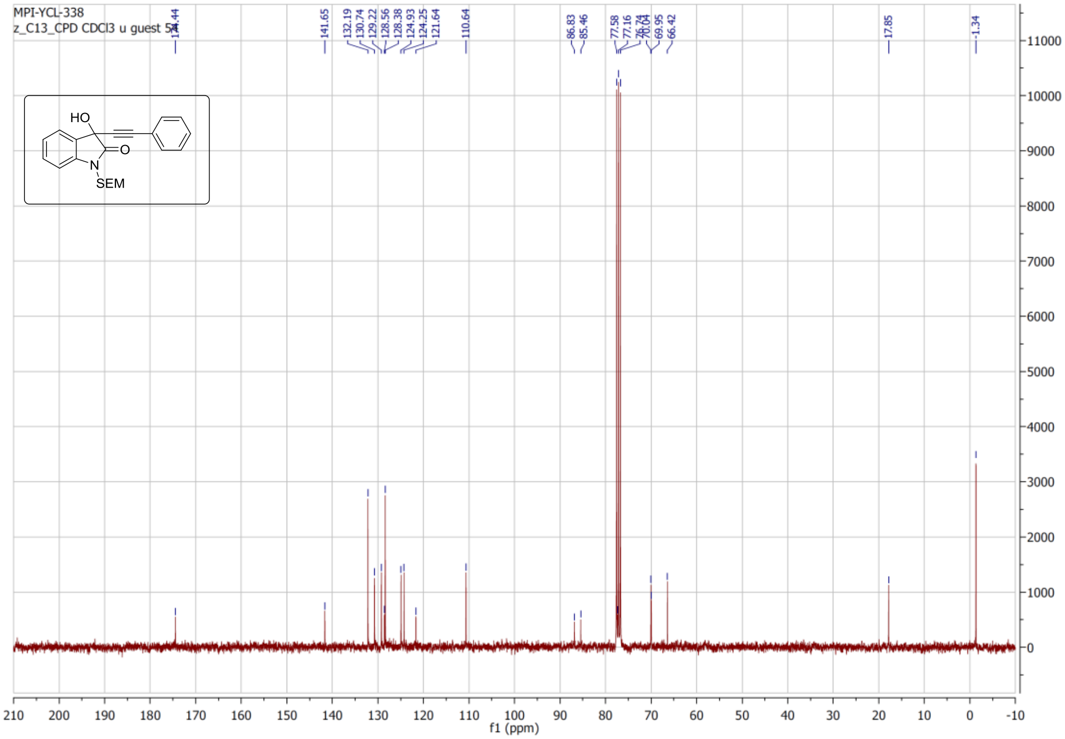

Supplementary Figure 34. ^{1}H and ^{13}C NMR spectra for **S9t.**

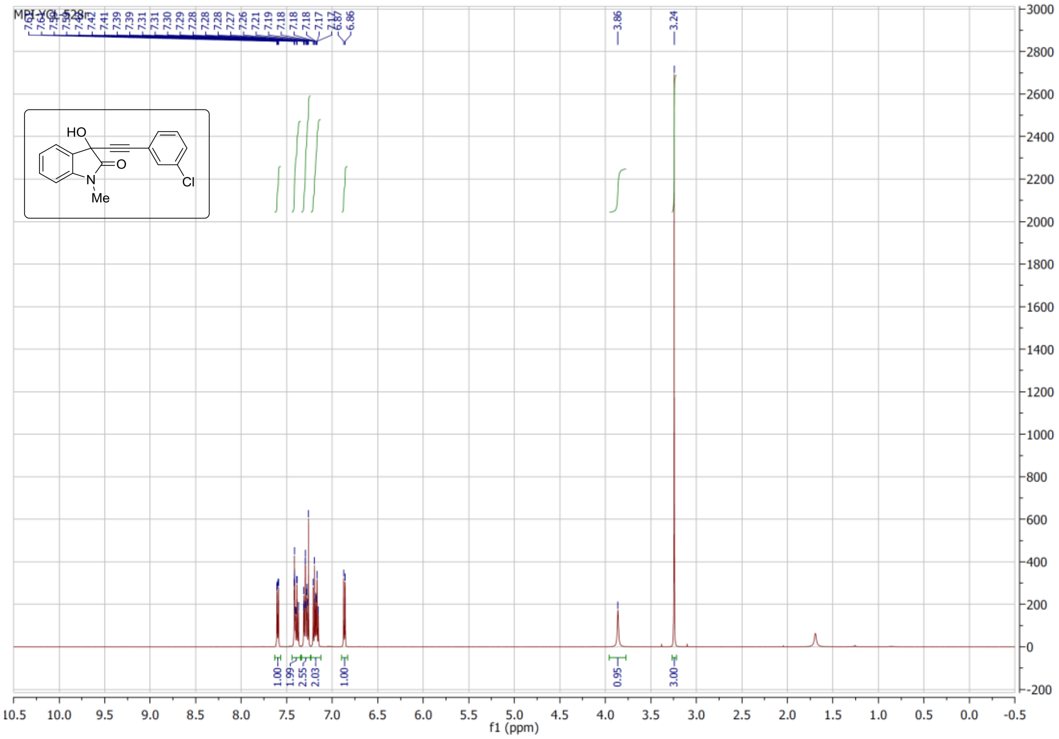

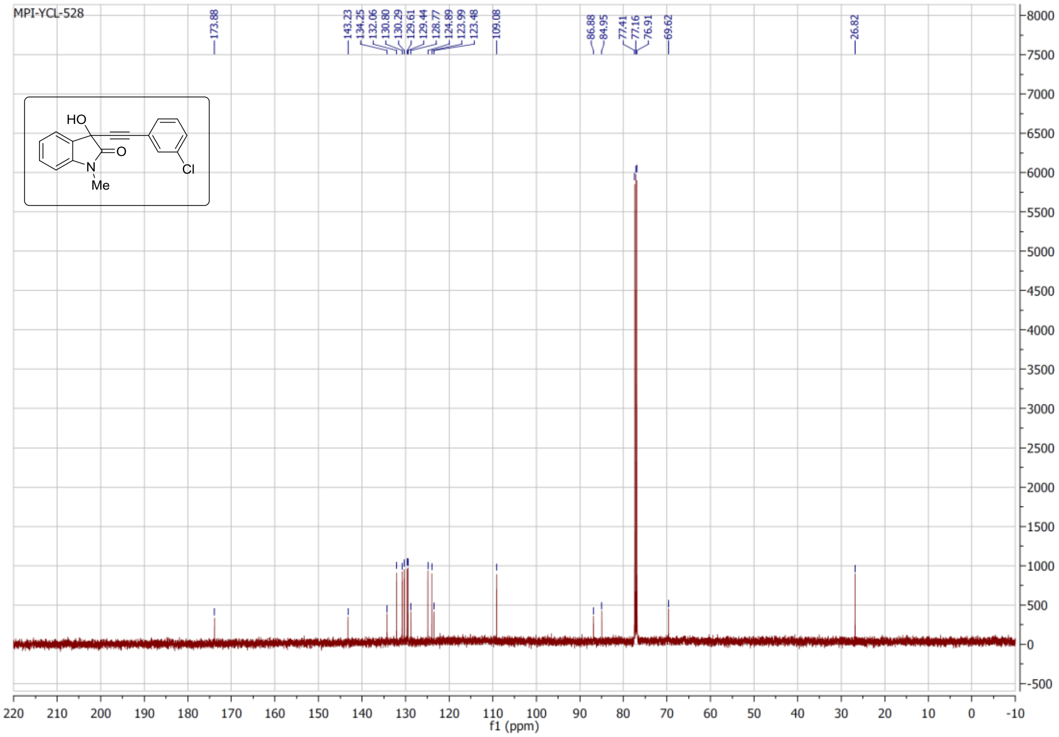

Supplementary Figure 35. ^{1}H and ^{13}C NMR spectra for **S9u.**

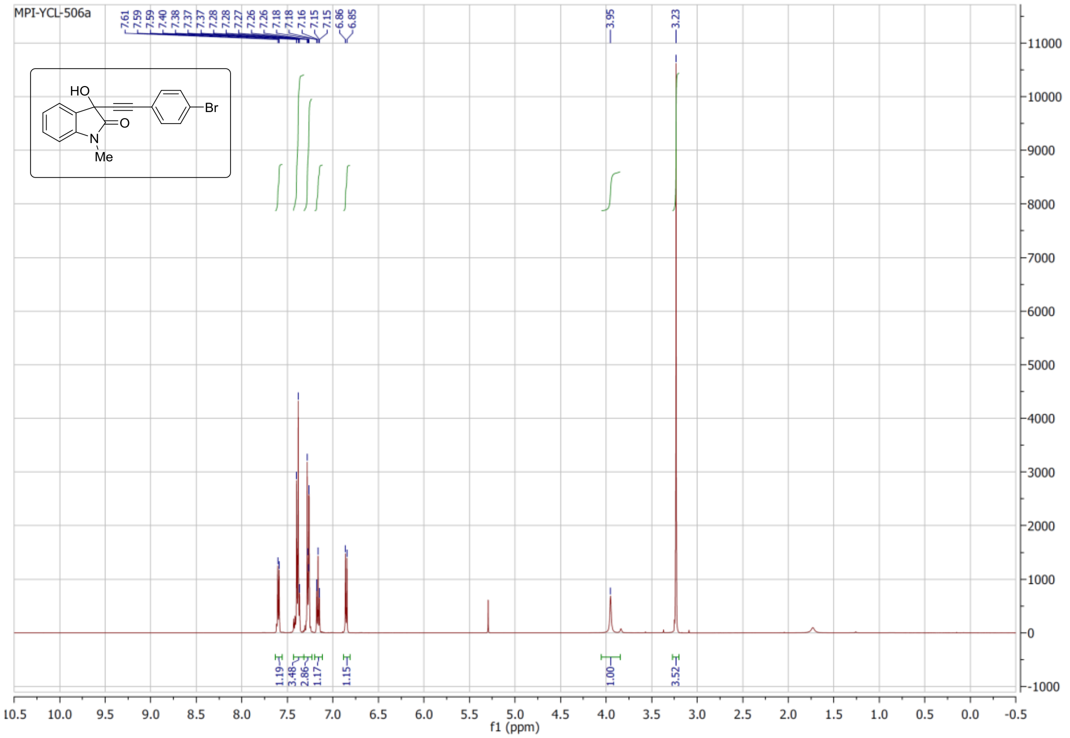

Supplementary Figure 36. ^{1}H NMR spectrum for **S9v.**

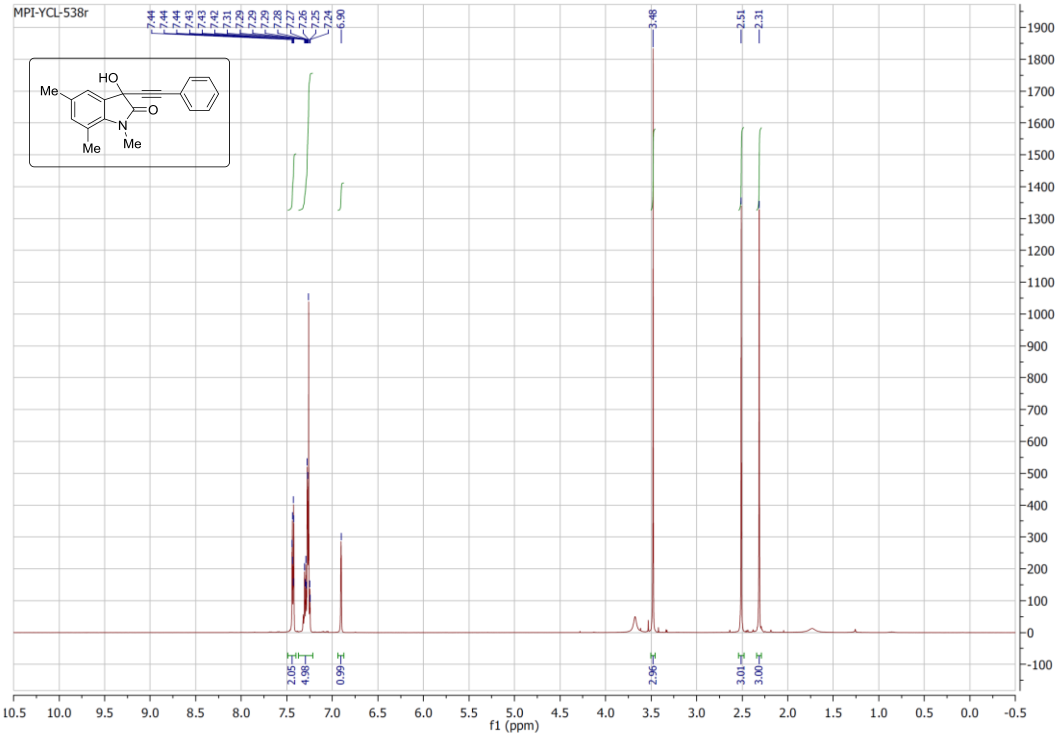

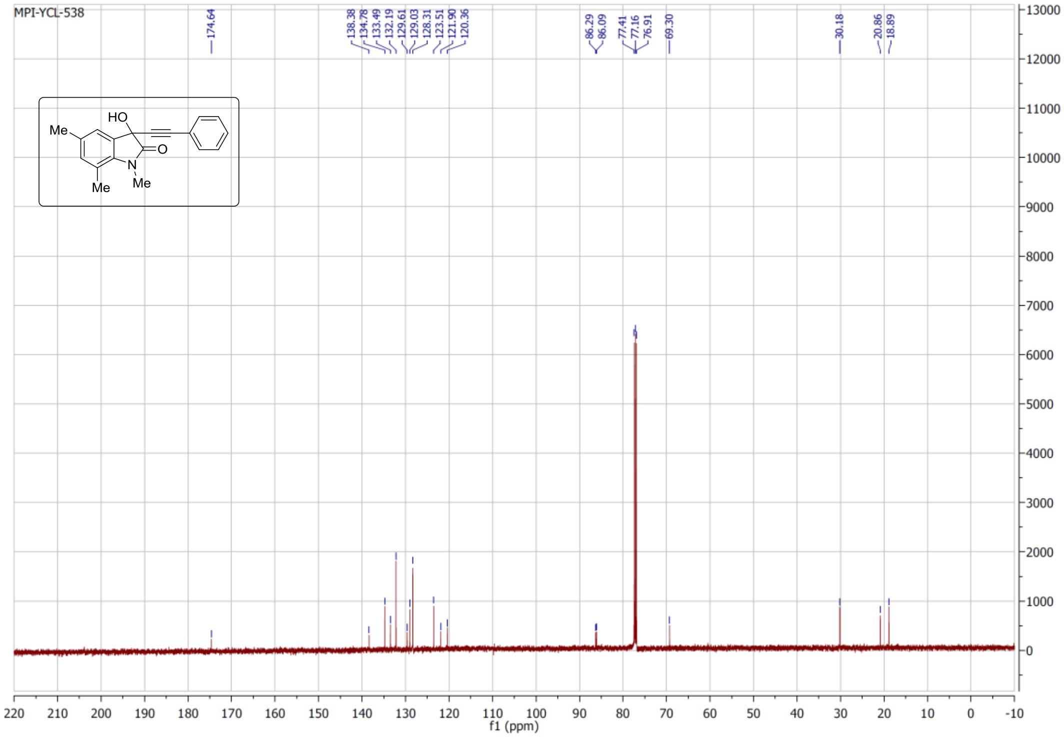

Supplementary Figure 37. ^{1}H and ^{13}C NMR spectra for **S9w.**

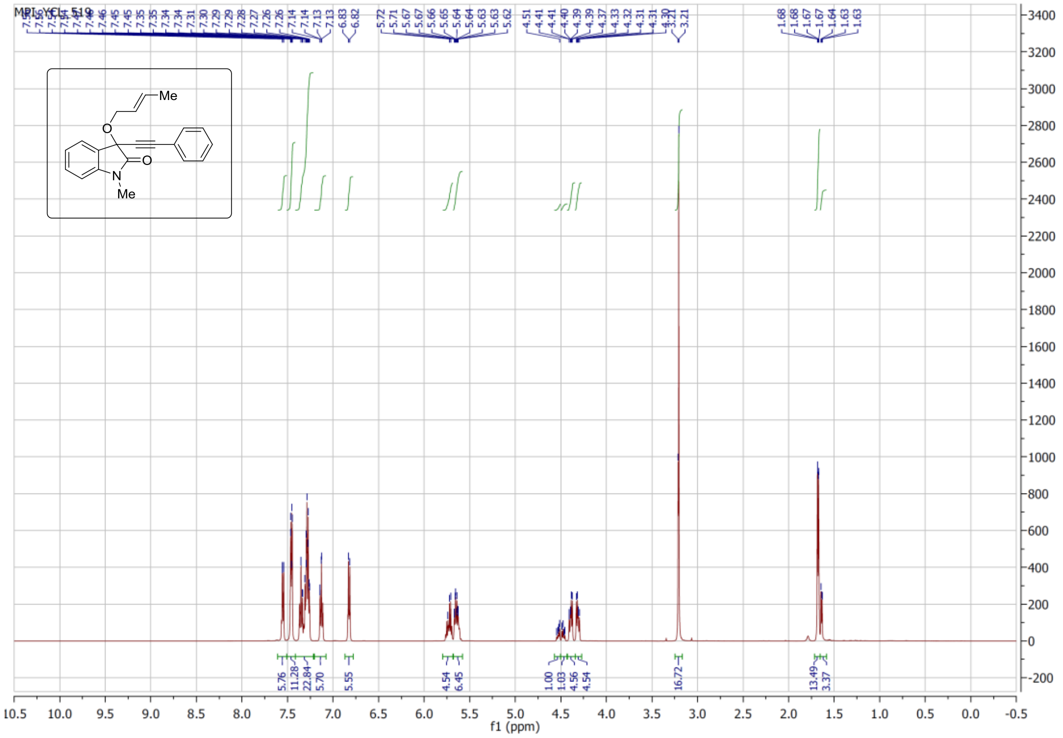

Supplementary Figure 38. ^{1}H NMR spectrum for **1a.**

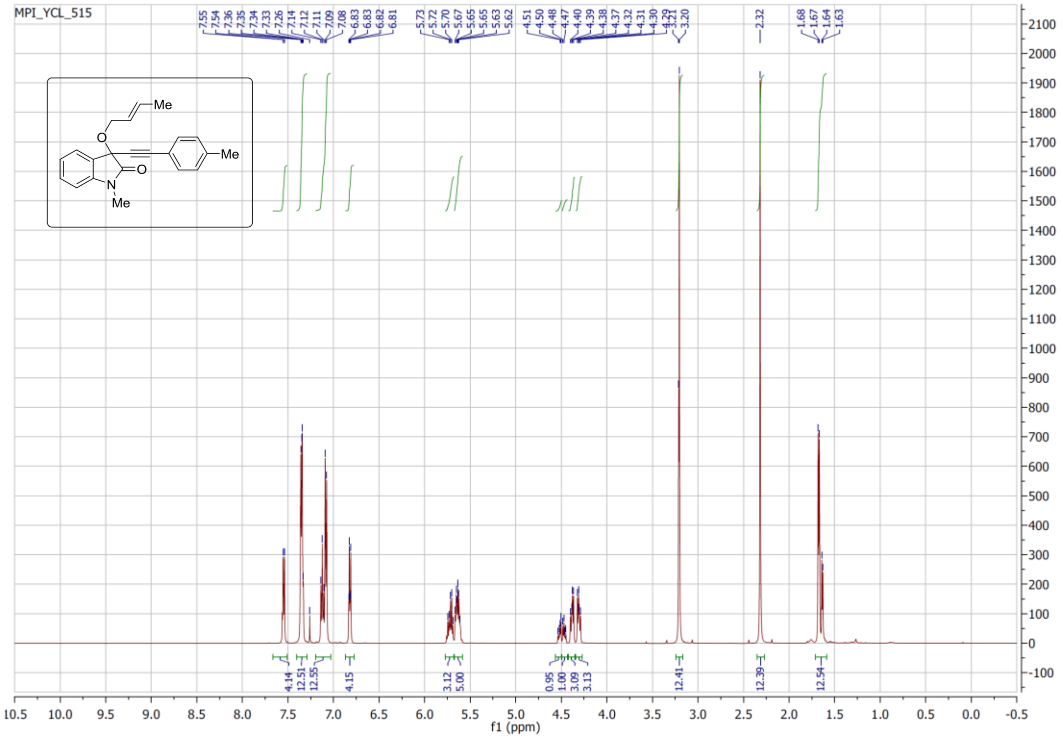

Supplementary Figure 39. ^{1}H NMR spectrum for **1b**.

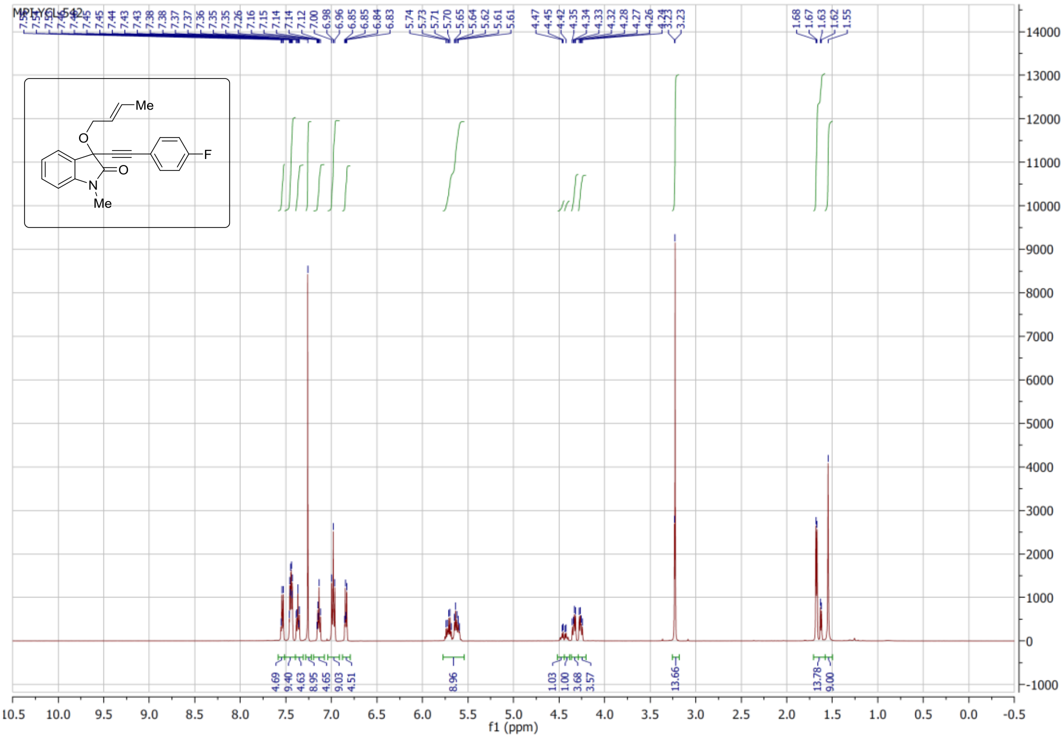

Supplementary Figure 40. ^{1}H NMR spectrum for **1c**.

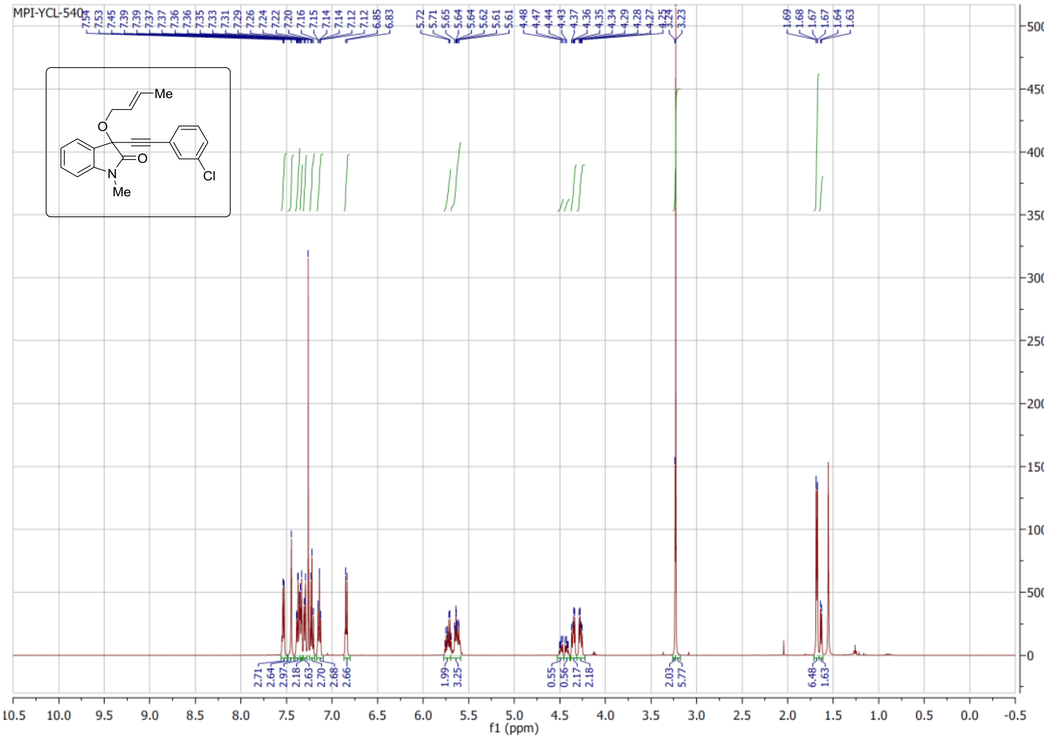

Supplementary Figure 41. ^{1}H NMR spectrum for **1d**.

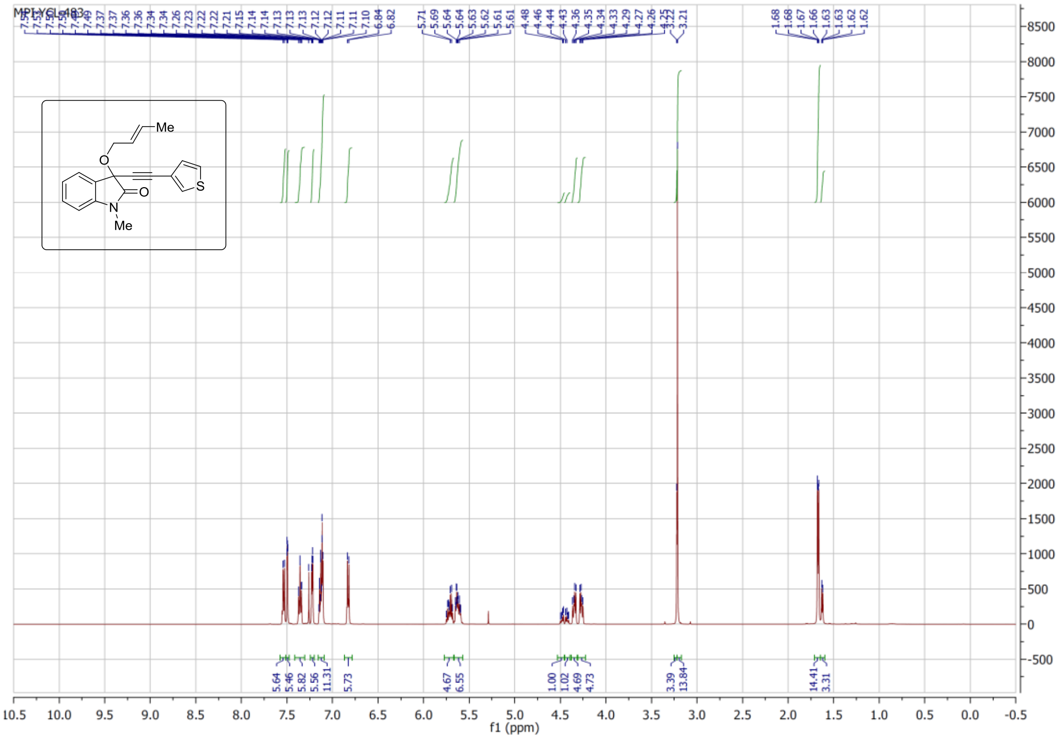

Supplementary Figure 42. ^{1}H NMR spectrum for **1e**.

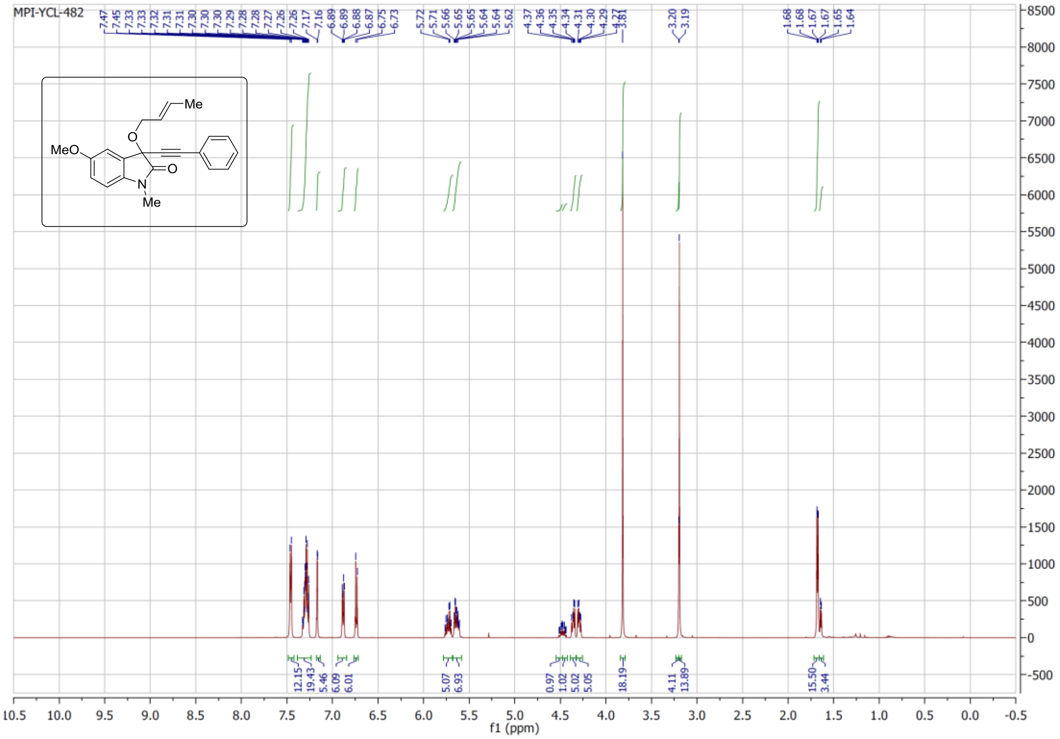

Supplementary Figure 43. ^{1}H NMR spectrum for **1f**.

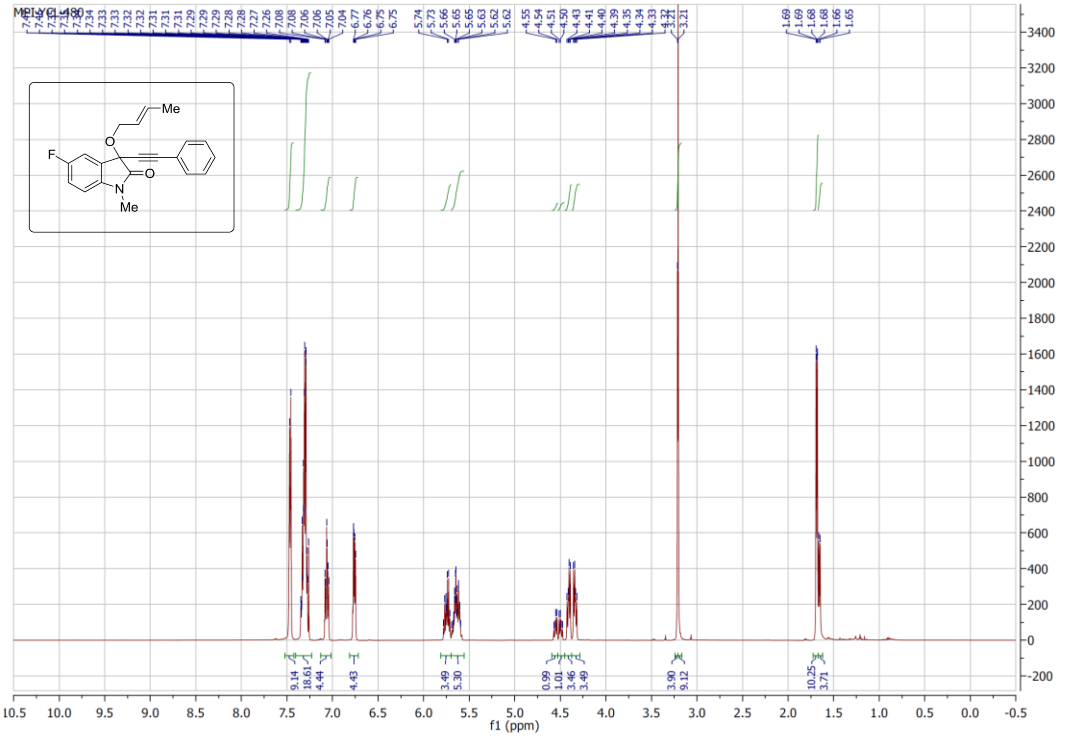

Supplementary Figure 44. ^{1}H NMR spectrum for **1g**.

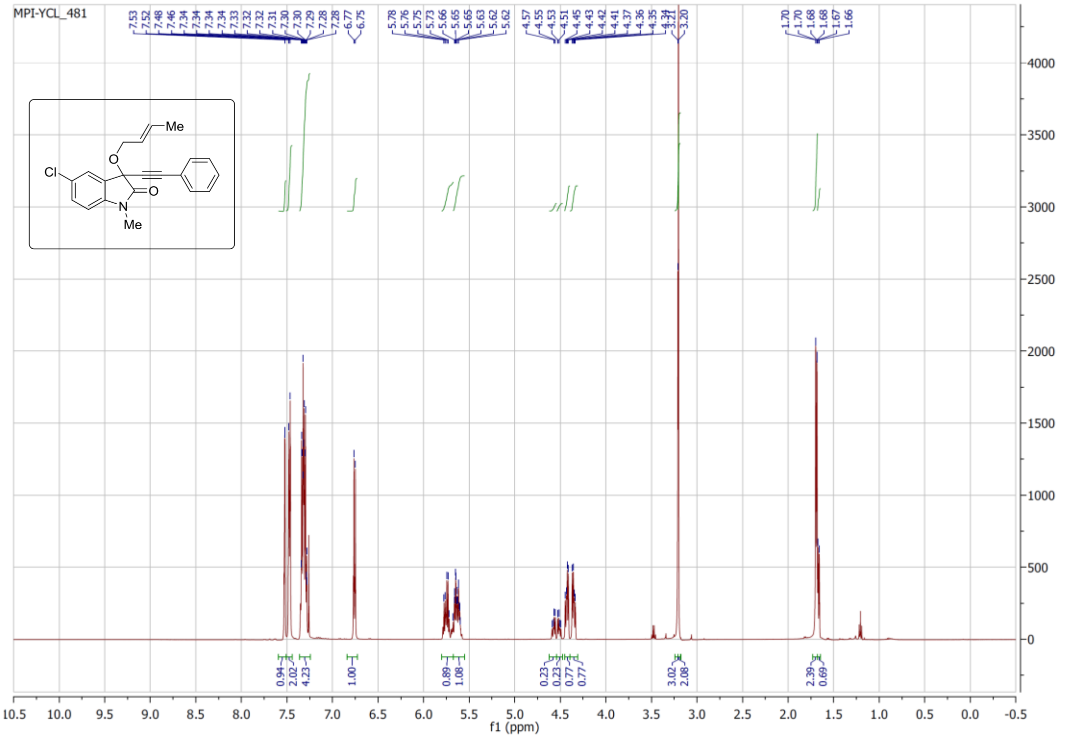

Supplementary Figure 45. ^{1}H NMR spectrum for **1h**.

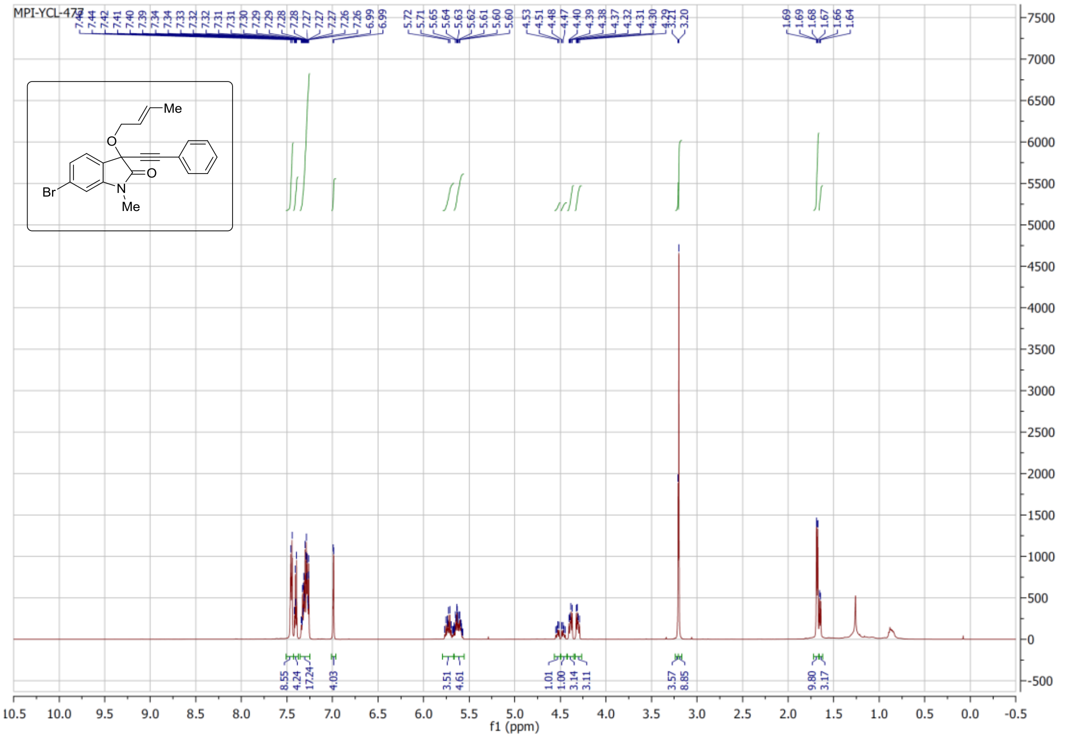

Supplementary Figure 46. ^{1}H NMR spectrum for **1i**.

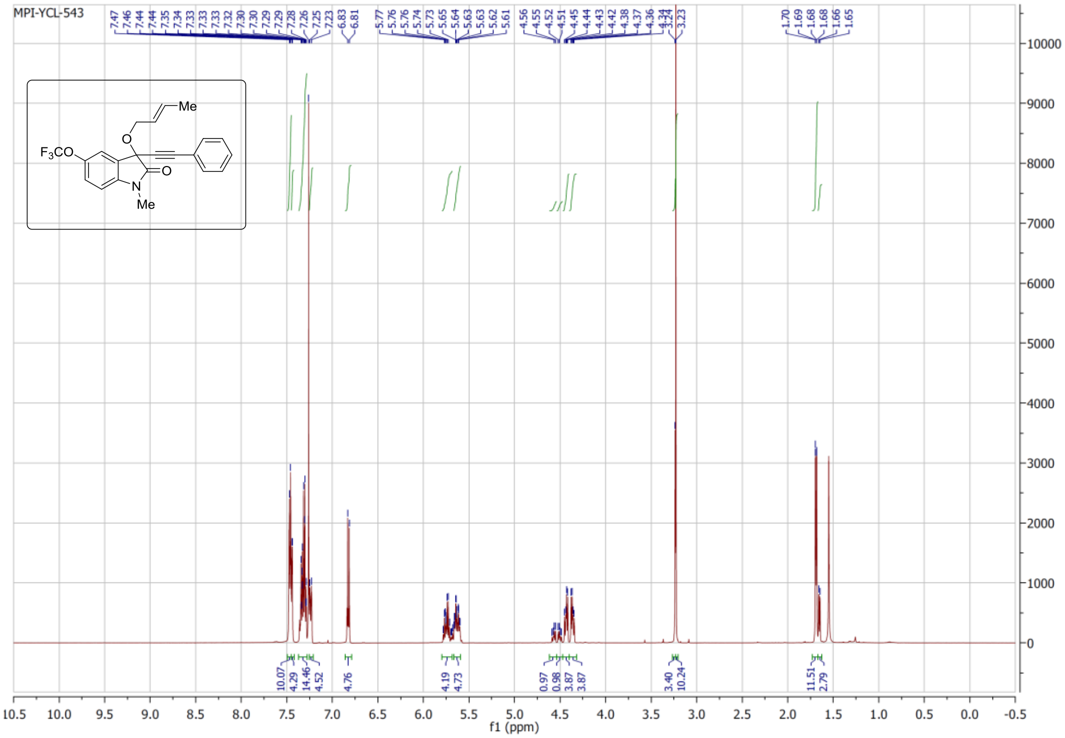

Supplementary Figure 47. ^{1}H NMR spectrum for **1j**.

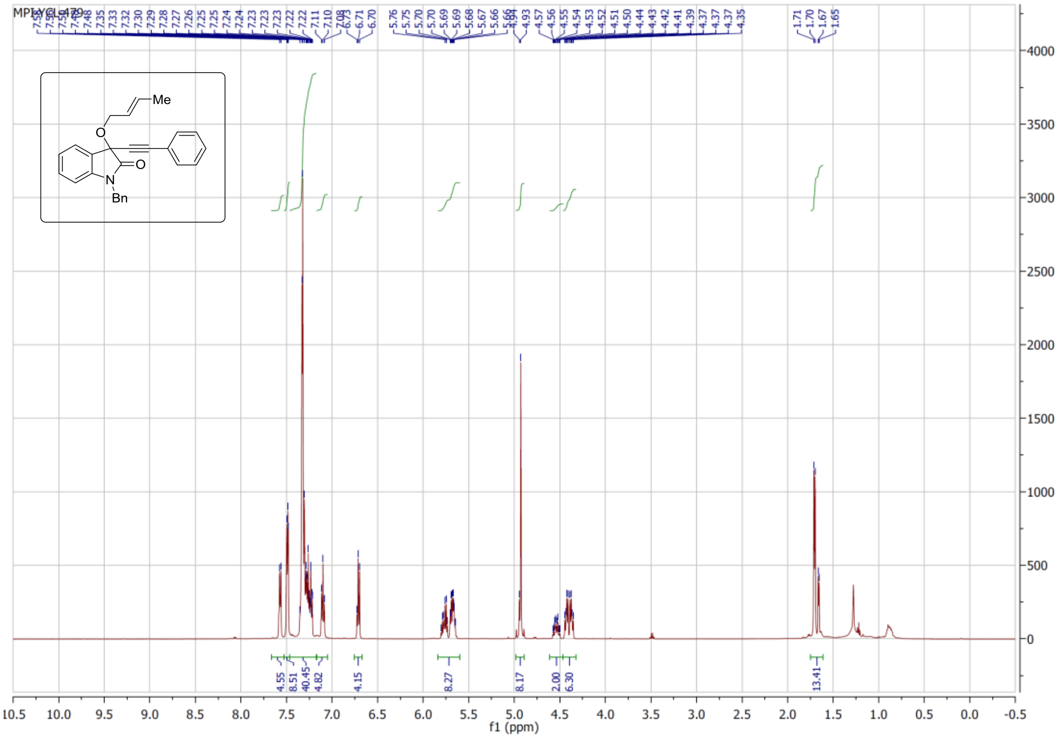

Supplementary Figure 48. ^{1}H NMR spectrum for **1k**.

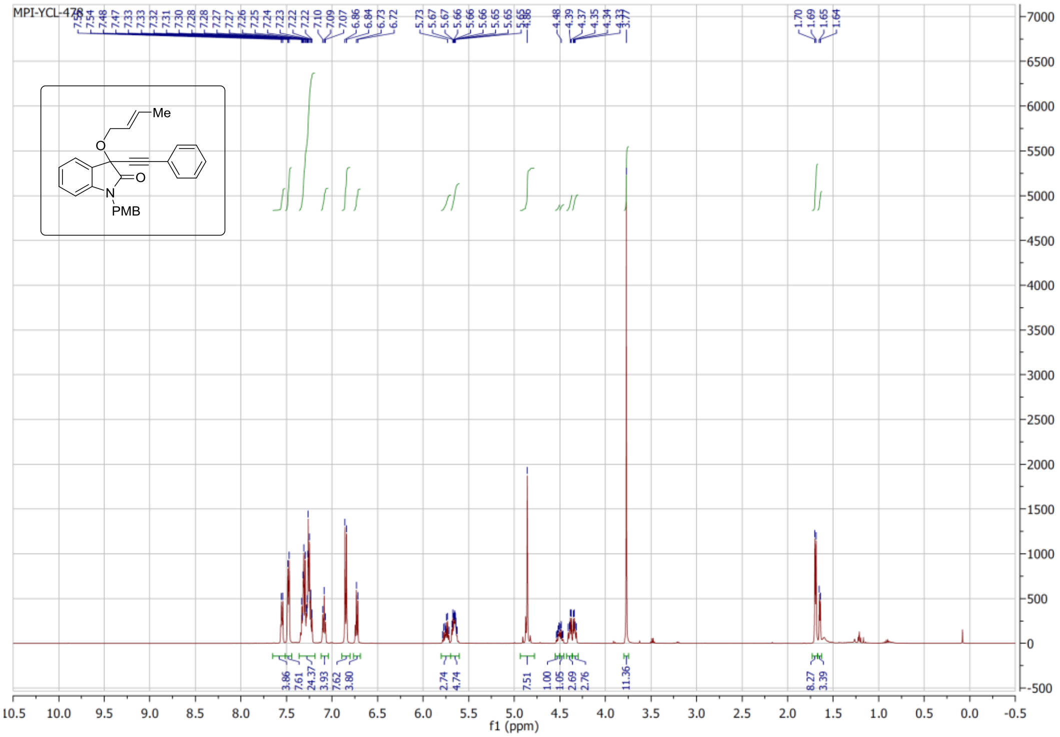

Supplementary Figure 49. ^{1}H NMR spectrum for **1l**.

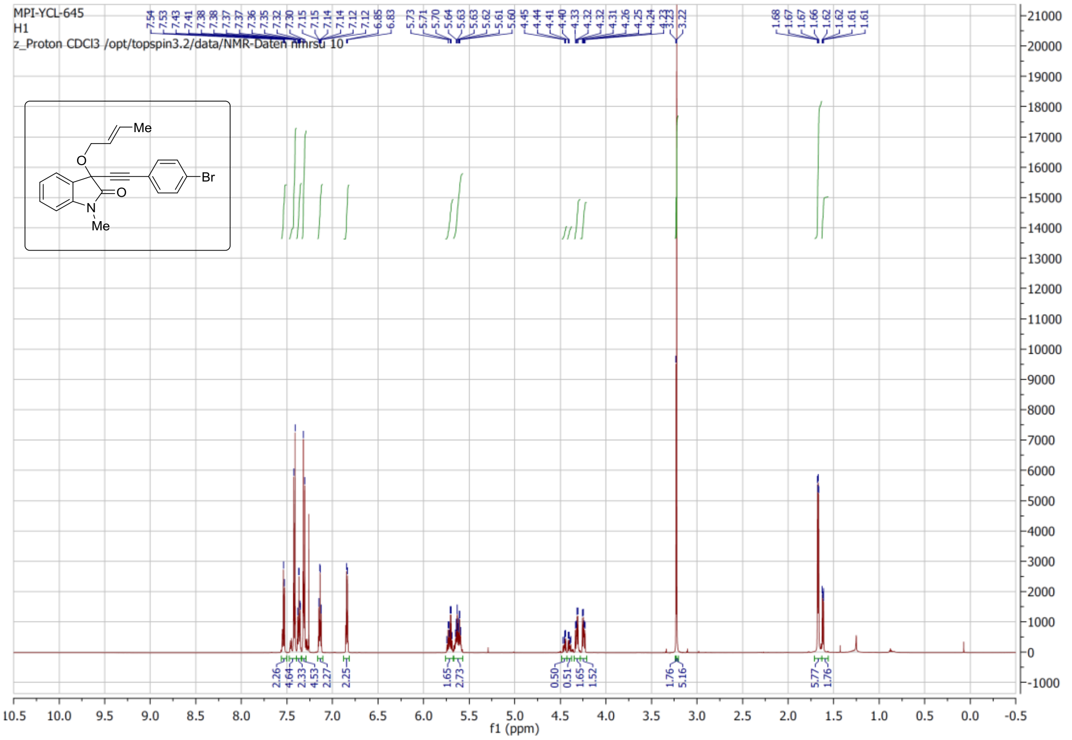

Supplementary Figure 50. ^{1}H NMR spectrum for **1m**.

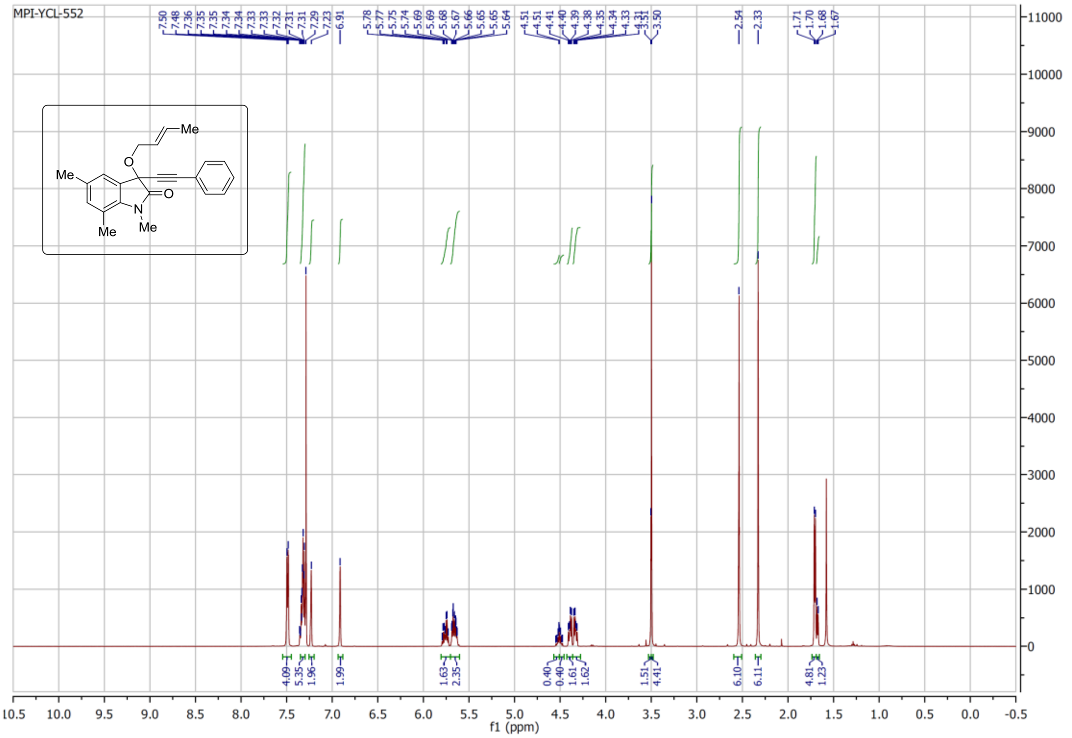

Supplementary Figure 51. ^{1}H NMR spectrum for **1n**.

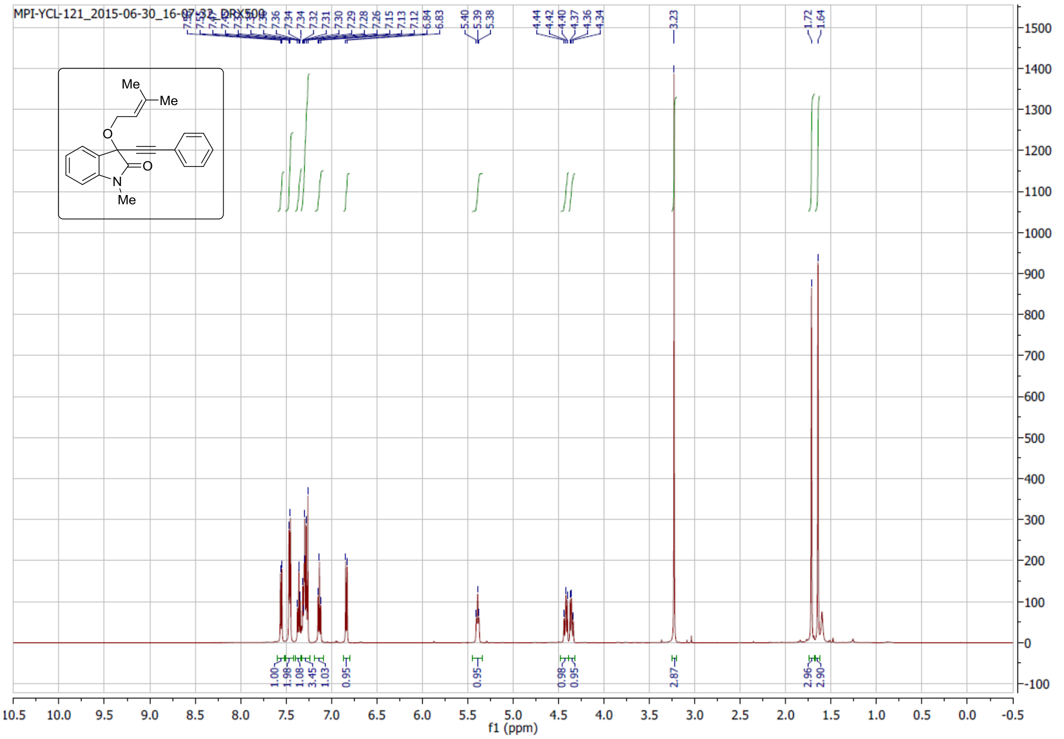

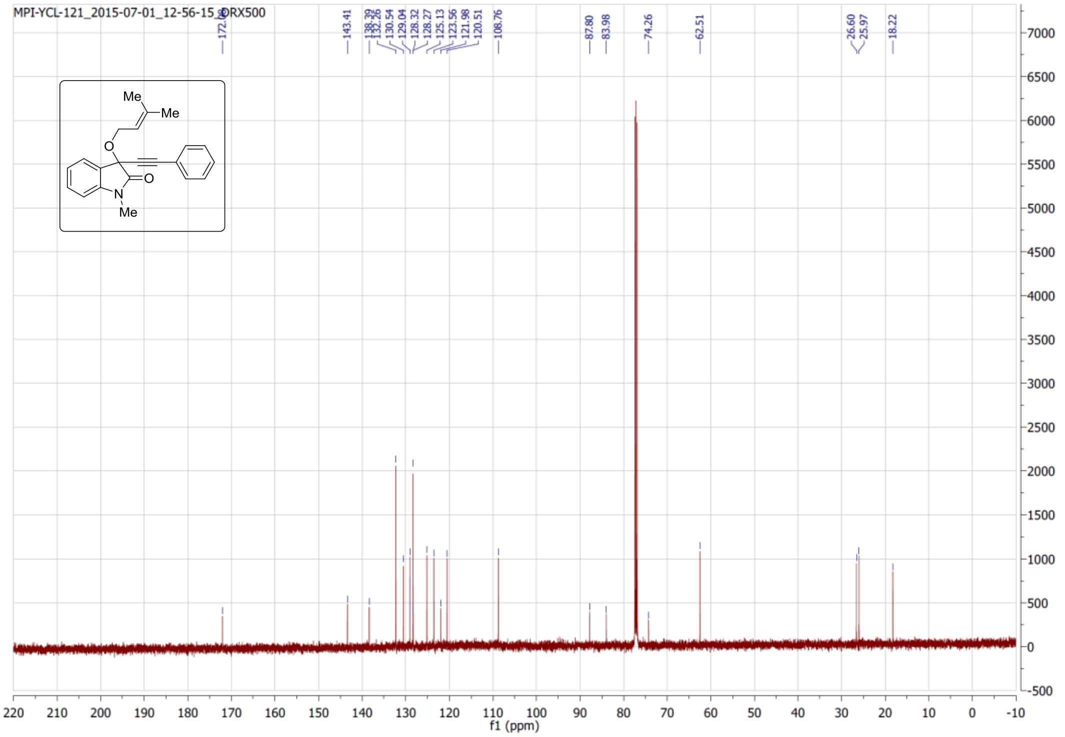

Supplementary Figure 52. ^{1}H and ^{13}C NMR spectra for **13a**.

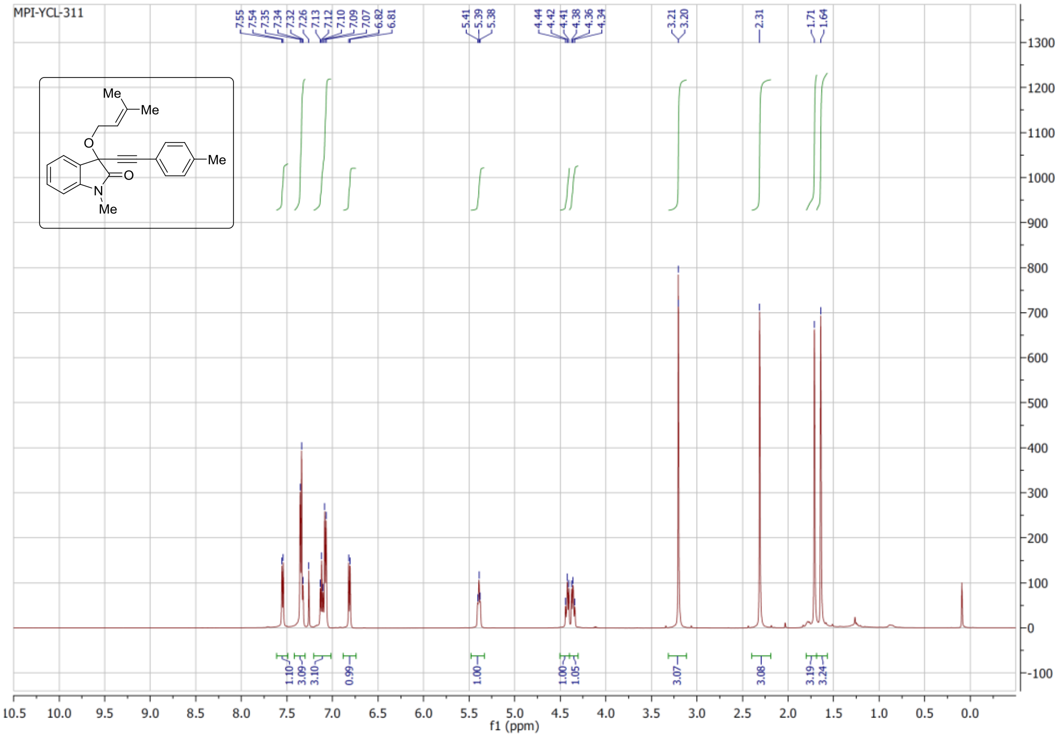

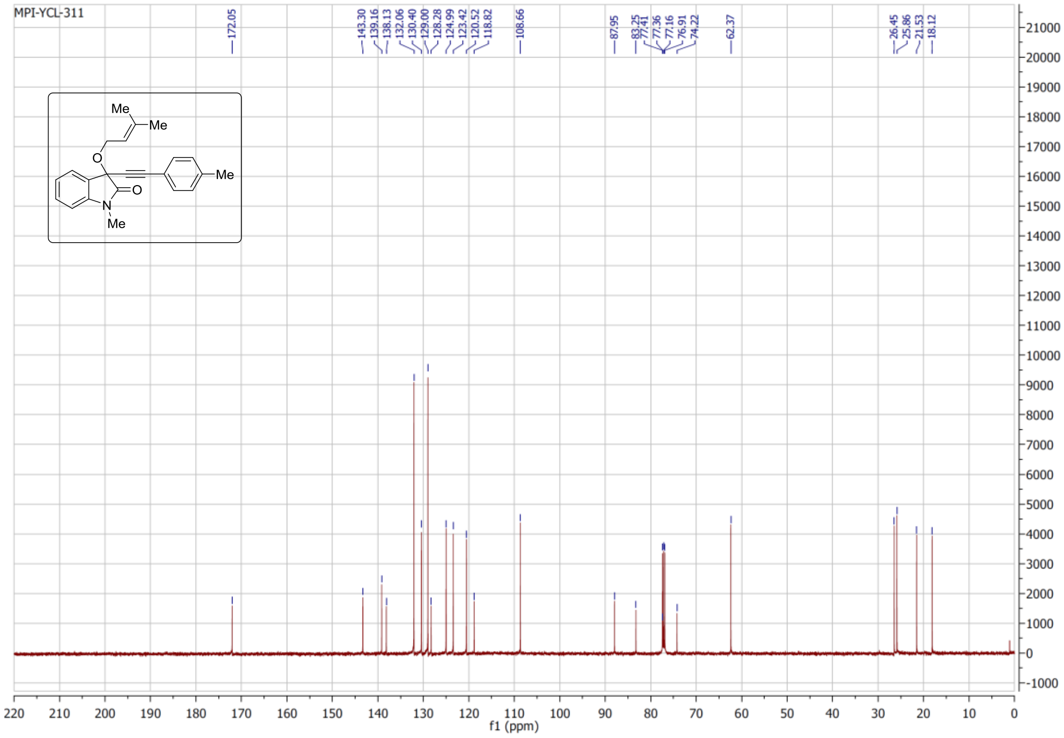

Supplementary Figure 53. ^{1}H and ^{13}C NMR spectra for **13b**.

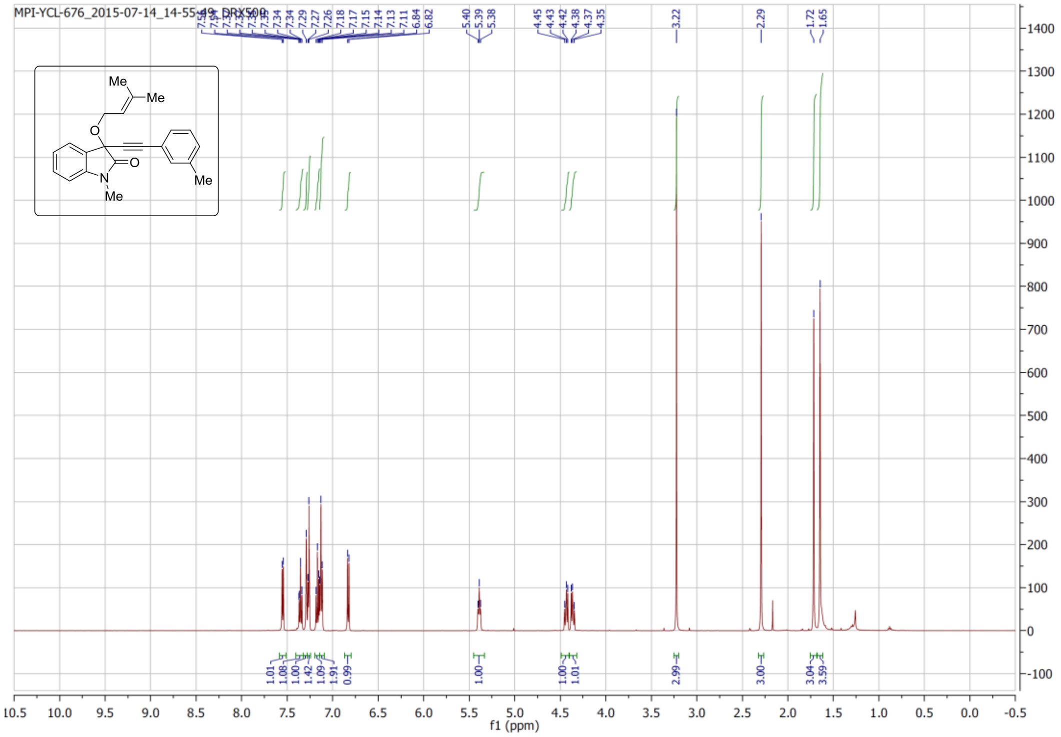

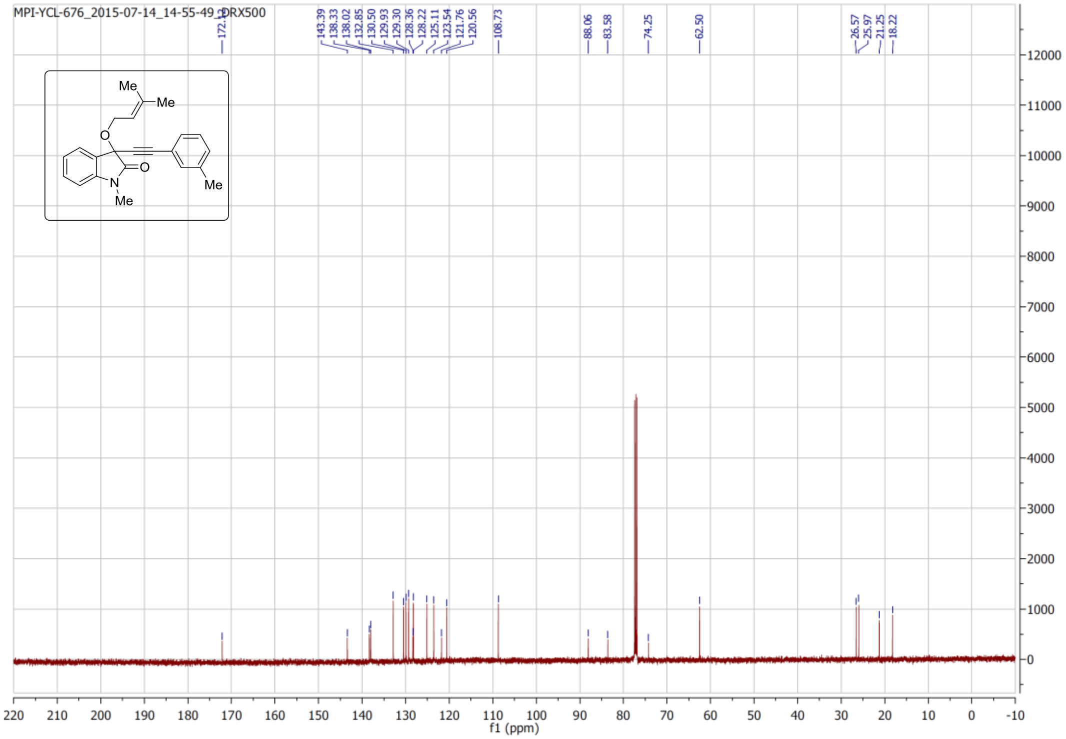

Supplementary Figure 54. ^{1}H and ^{13}C NMR spectra for **13c**.

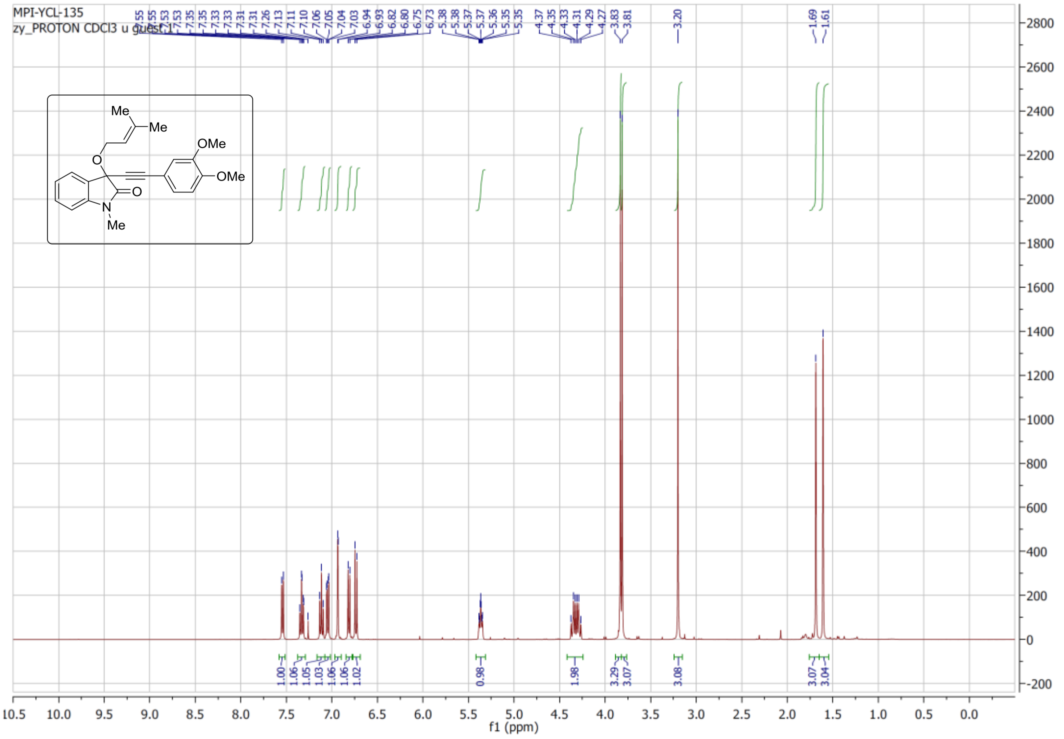

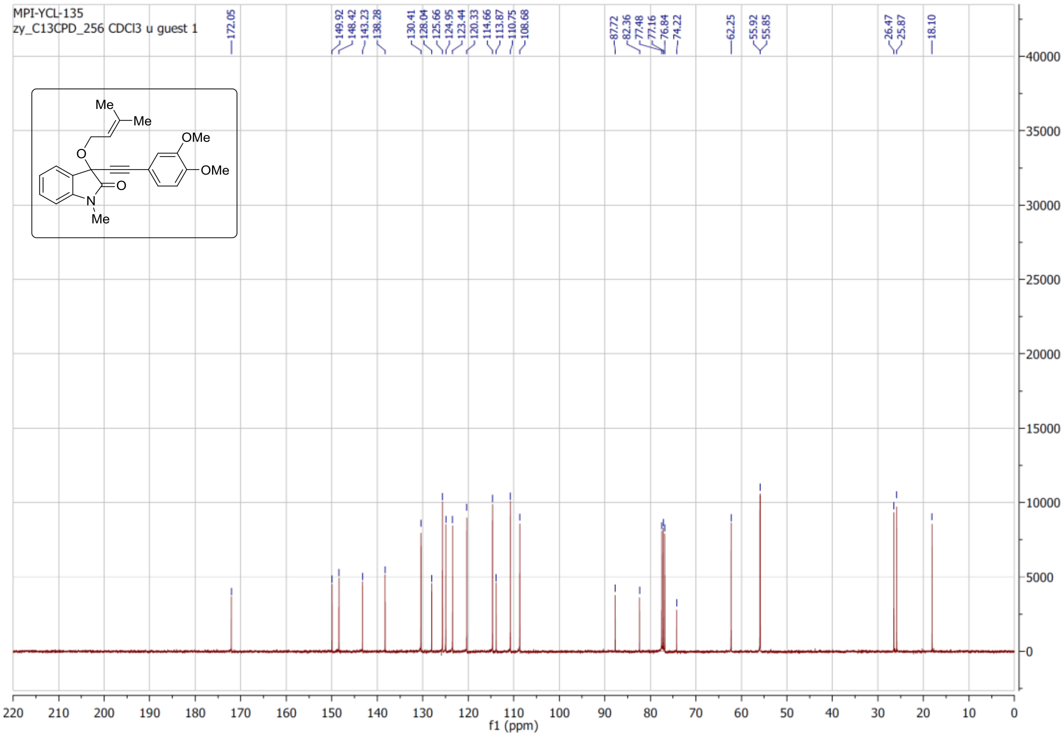

Supplementary Figure 55. ^{1}H and ^{13}C NMR spectra for **13d**.

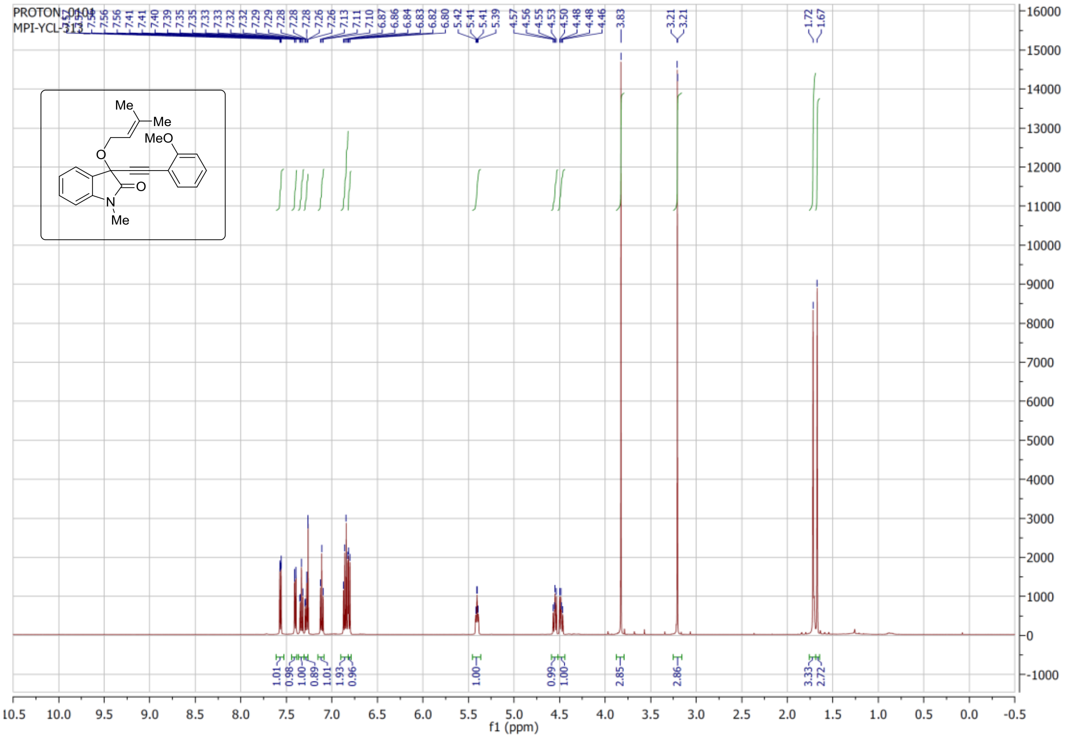

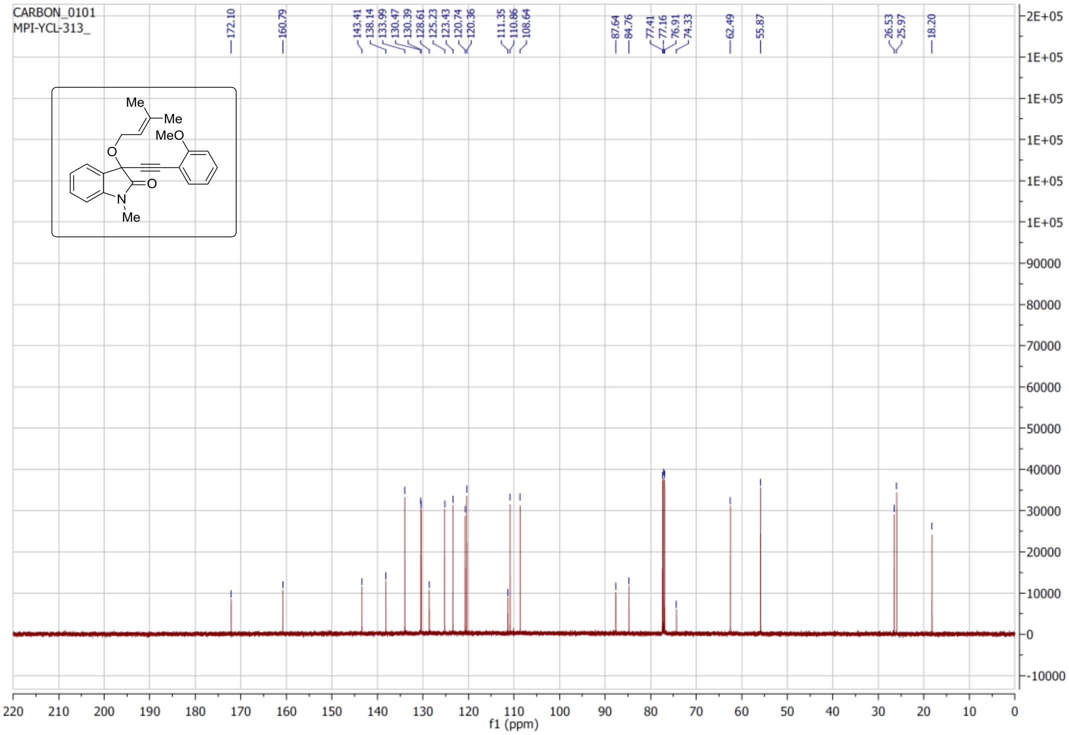

Supplementary Figure 56. ^{1}H and ^{13}C NMR spectra for **13e**.

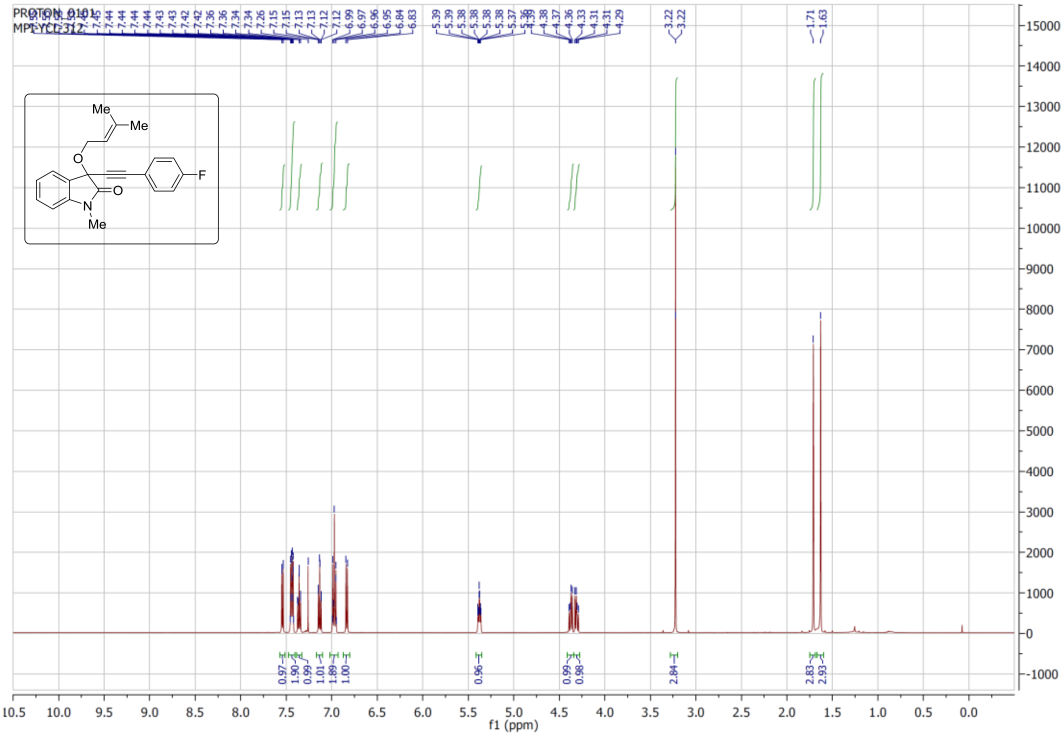

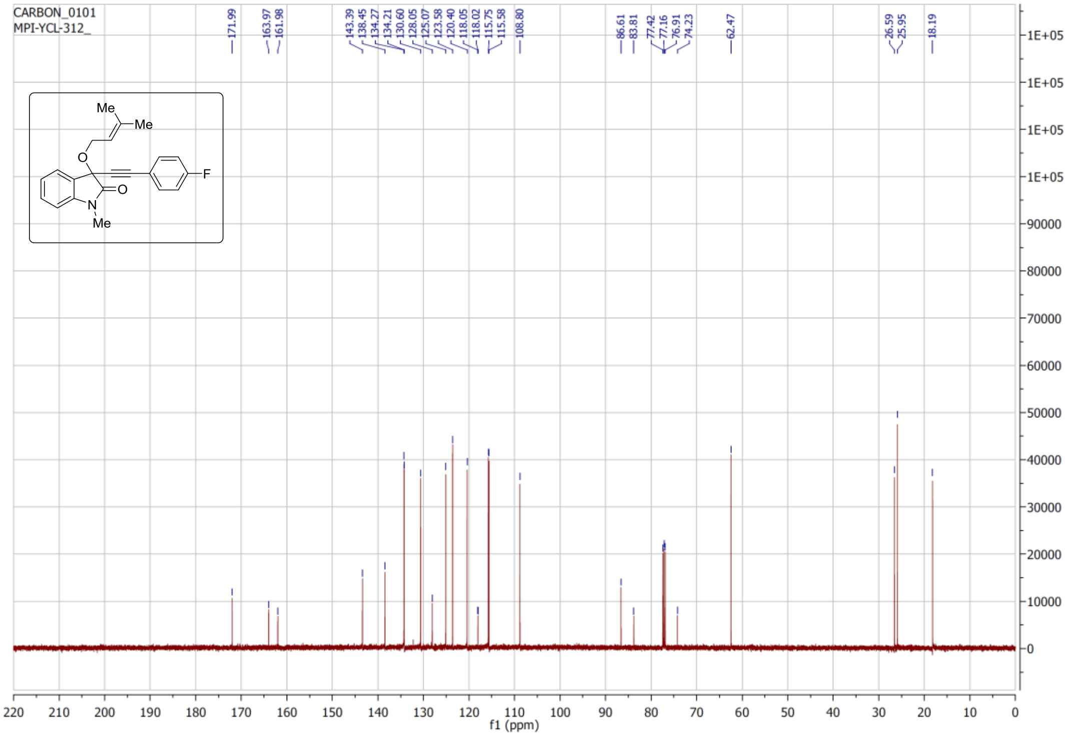

Supplementary Figure 57. ^{1}H and ^{13}C NMR spectra for **13f**.

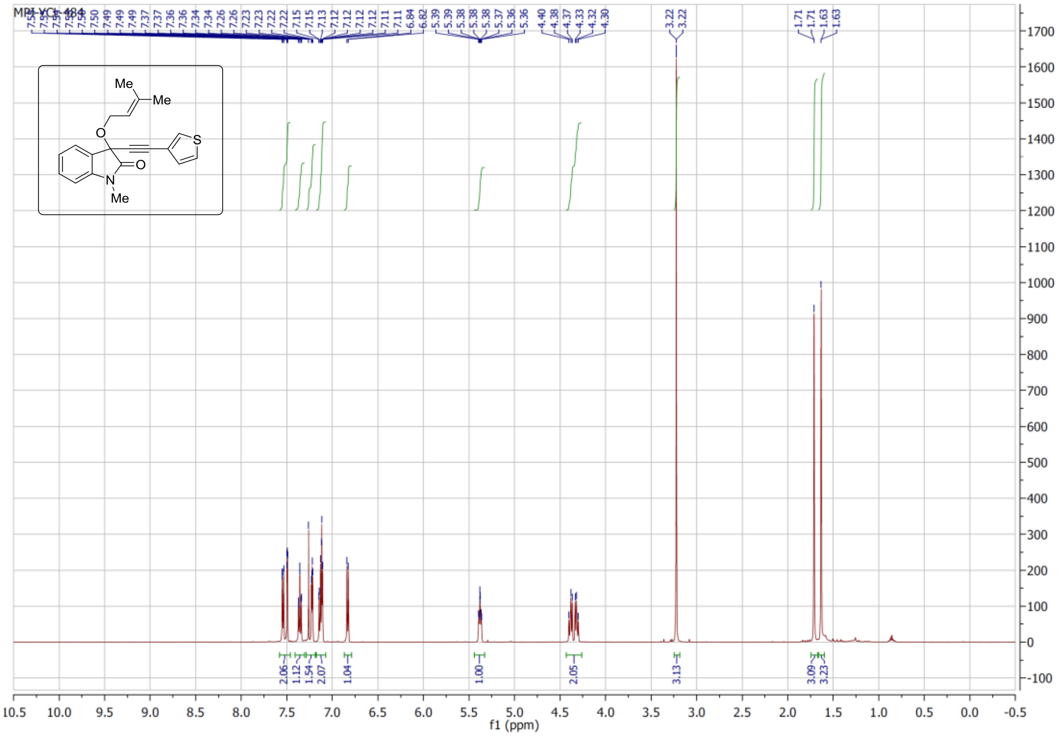

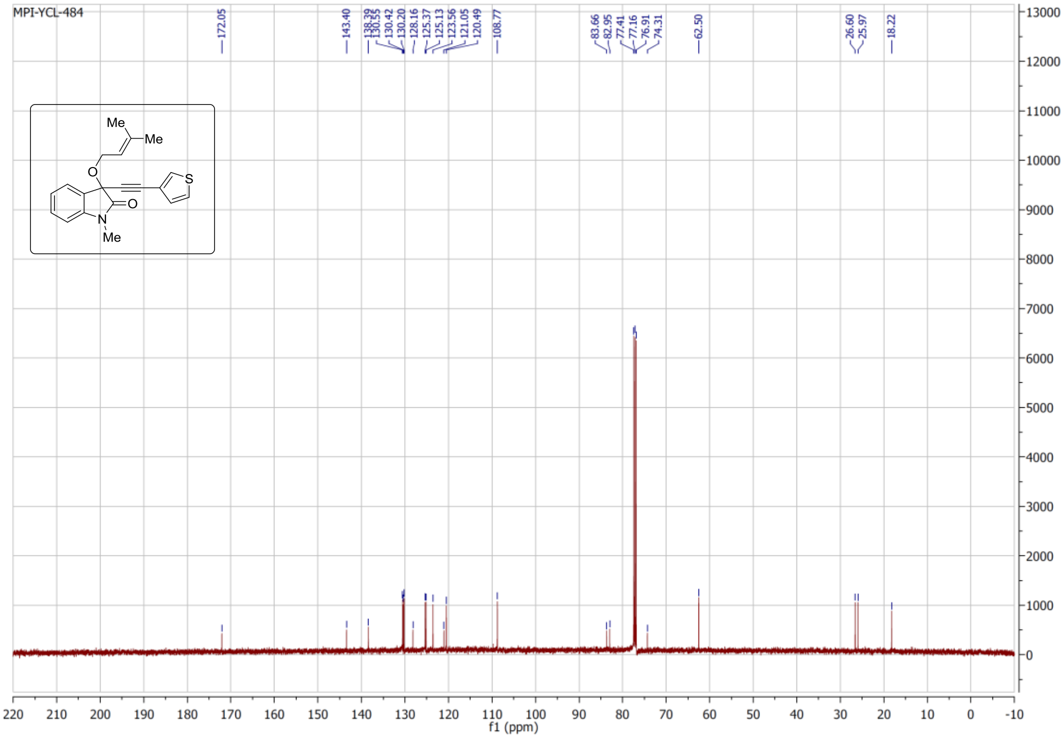

Supplementary Figure 58. ^{1}H and ^{13}C NMR spectra for **13g**.

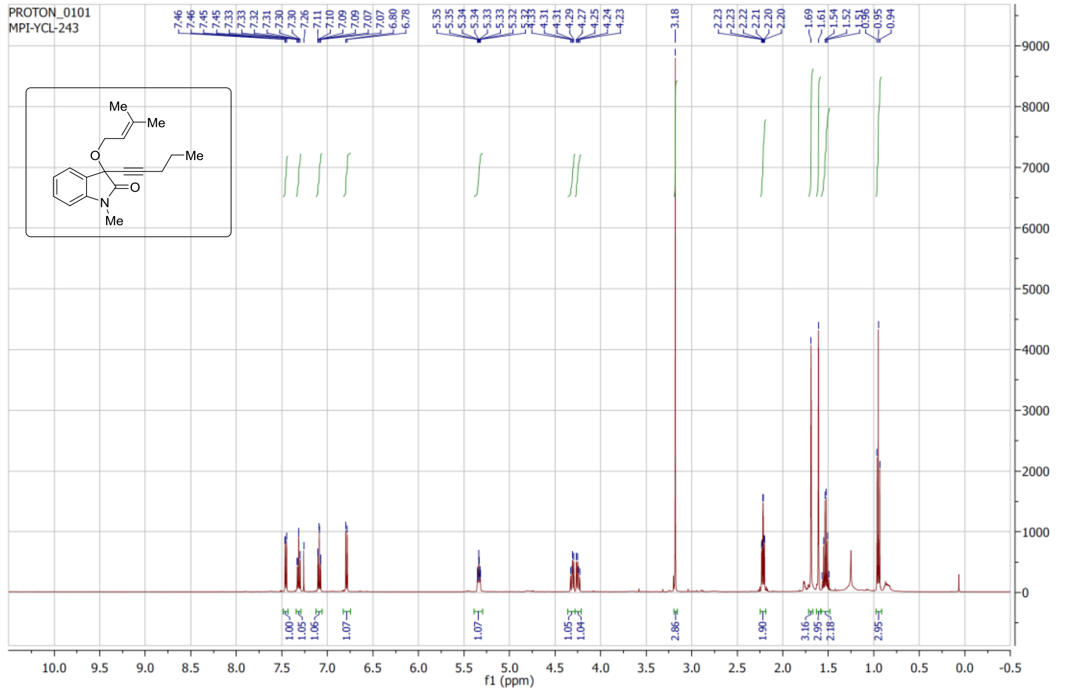

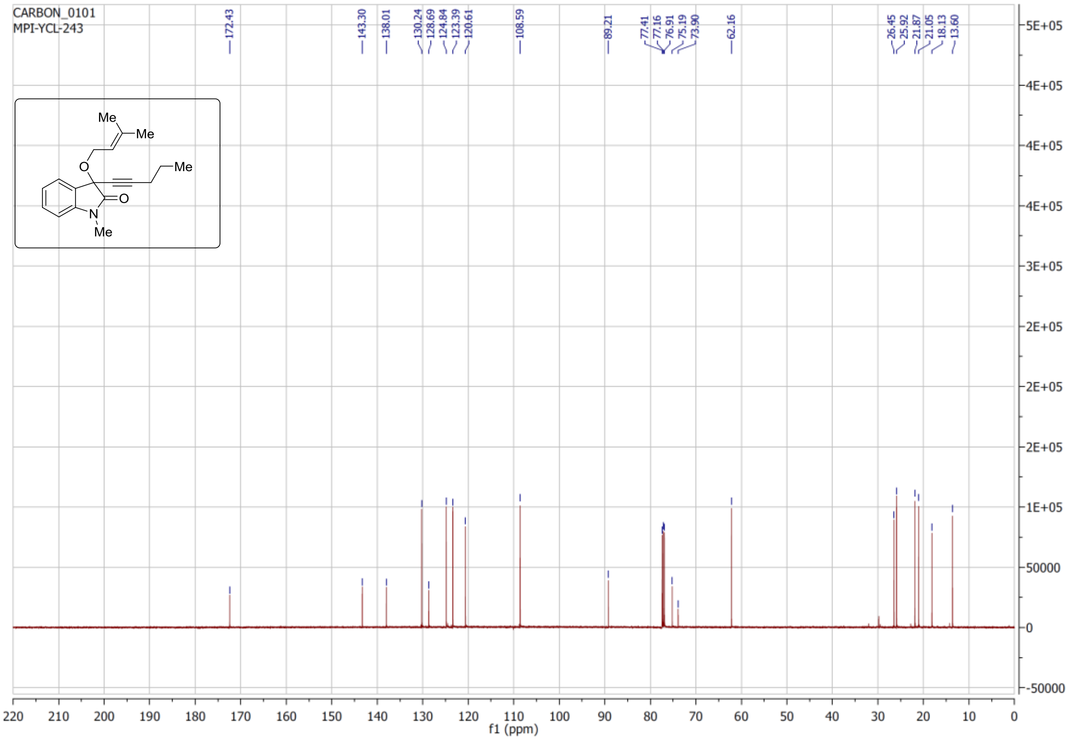

Supplementary Figure 59. ^{1}H and ^{13}C NMR spectra for **13h**.

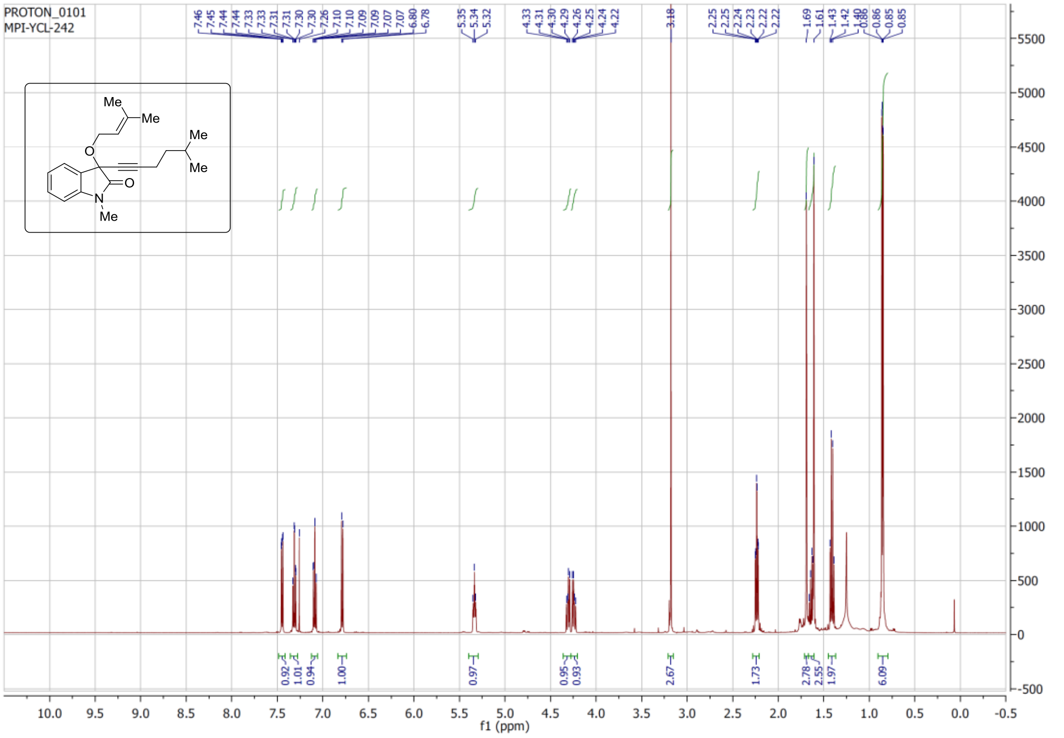

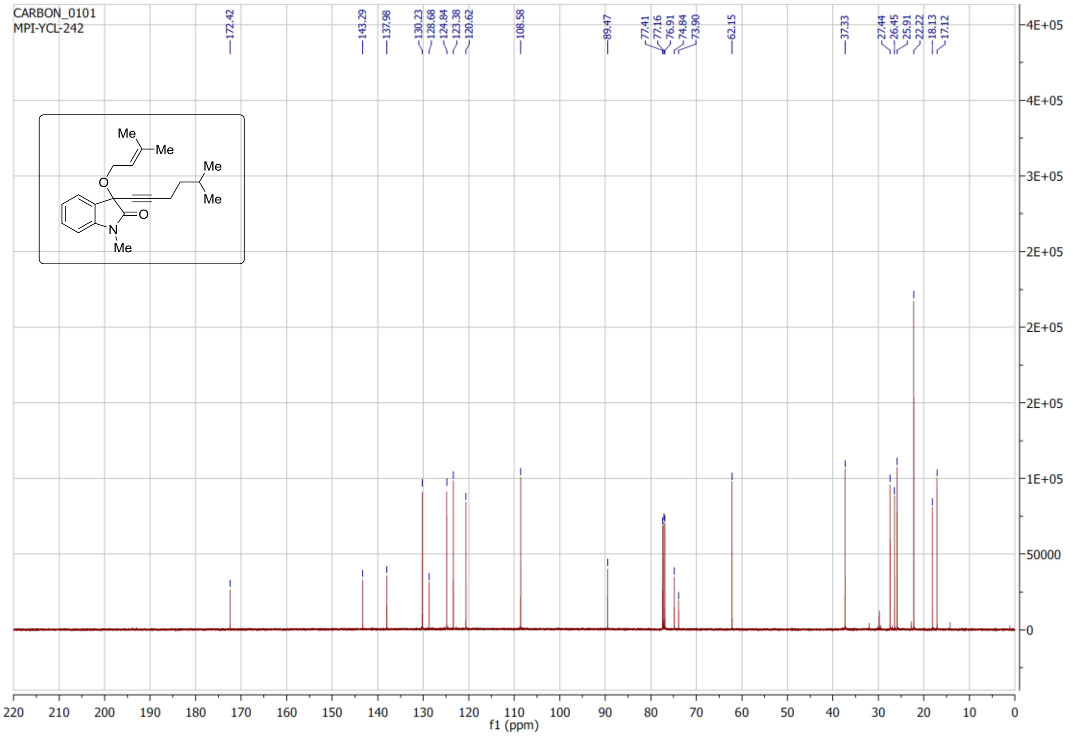

Supplementary Figure 60. ^{1}H and ^{13}C NMR spectra for **13i**.

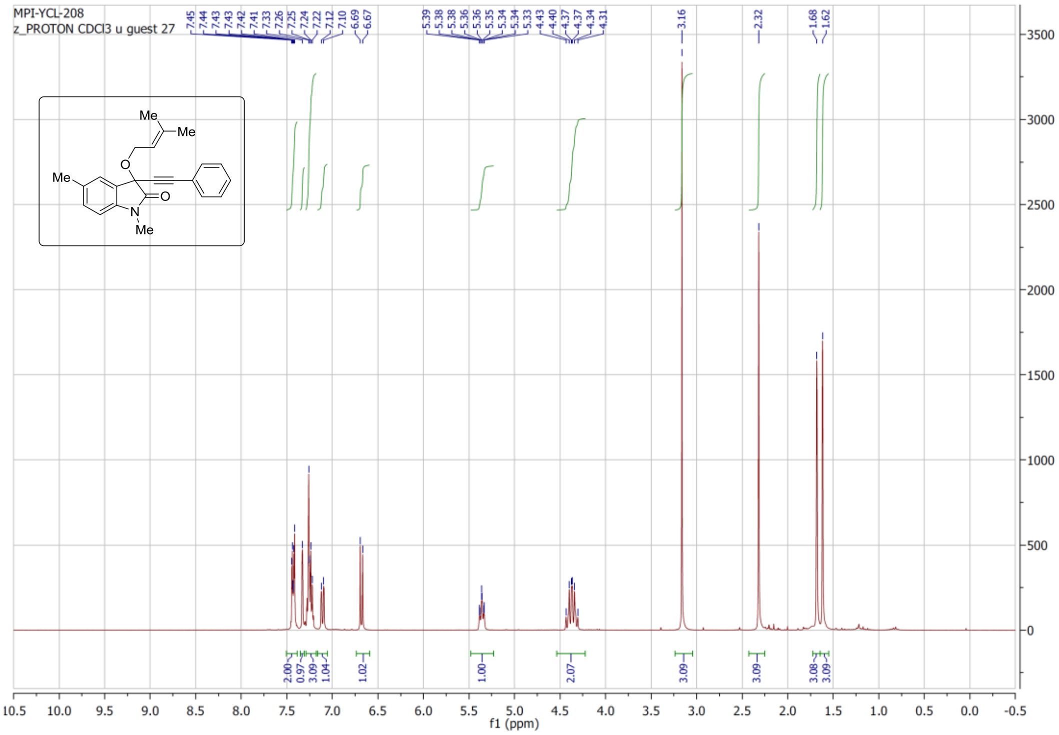

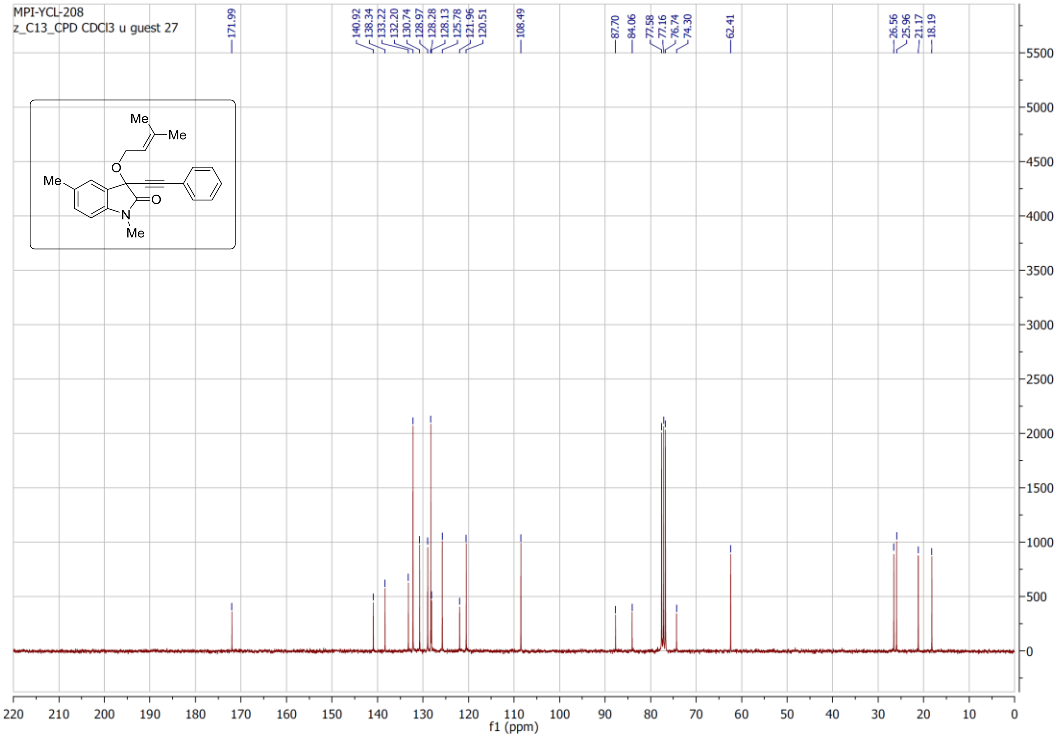

Supplementary Figure 61. ^{1}H and ^{13}C NMR spectra for **13j**.

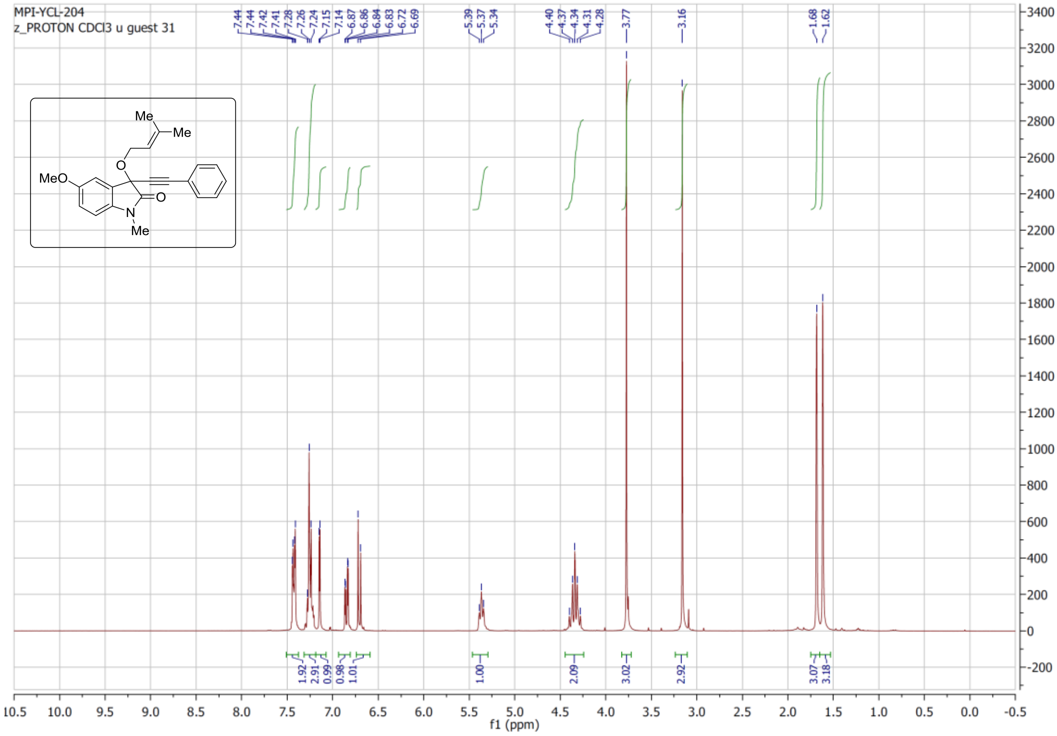

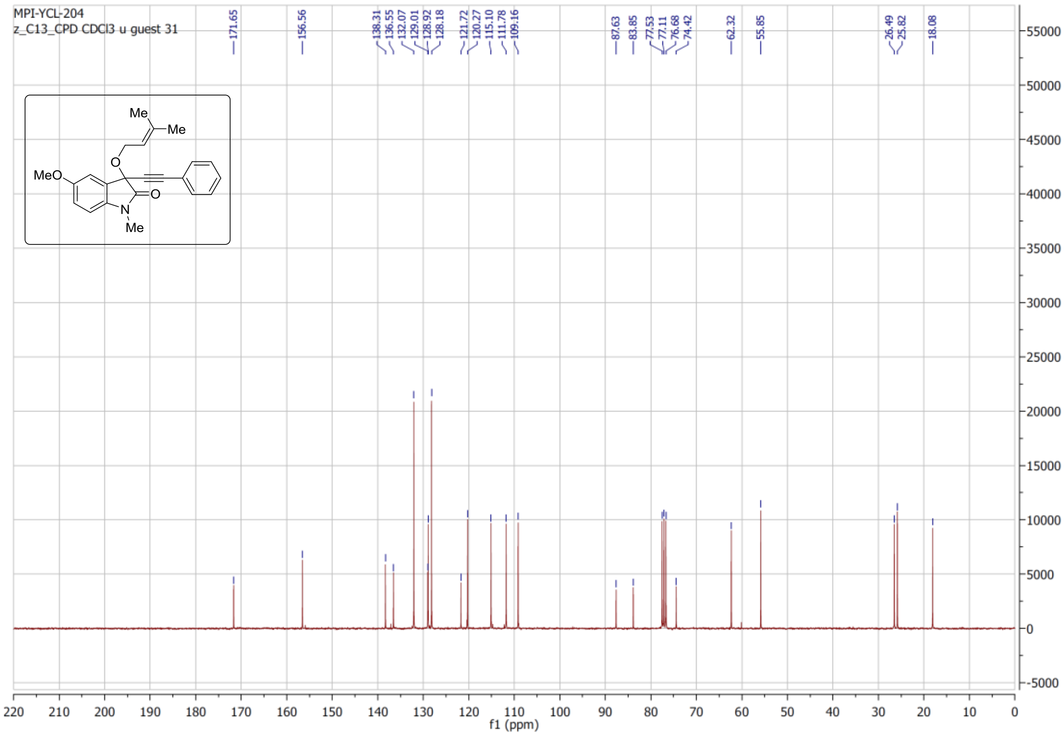

Supplementary Figure 62. ^{1}H and ^{13}C NMR spectra for **13k**.

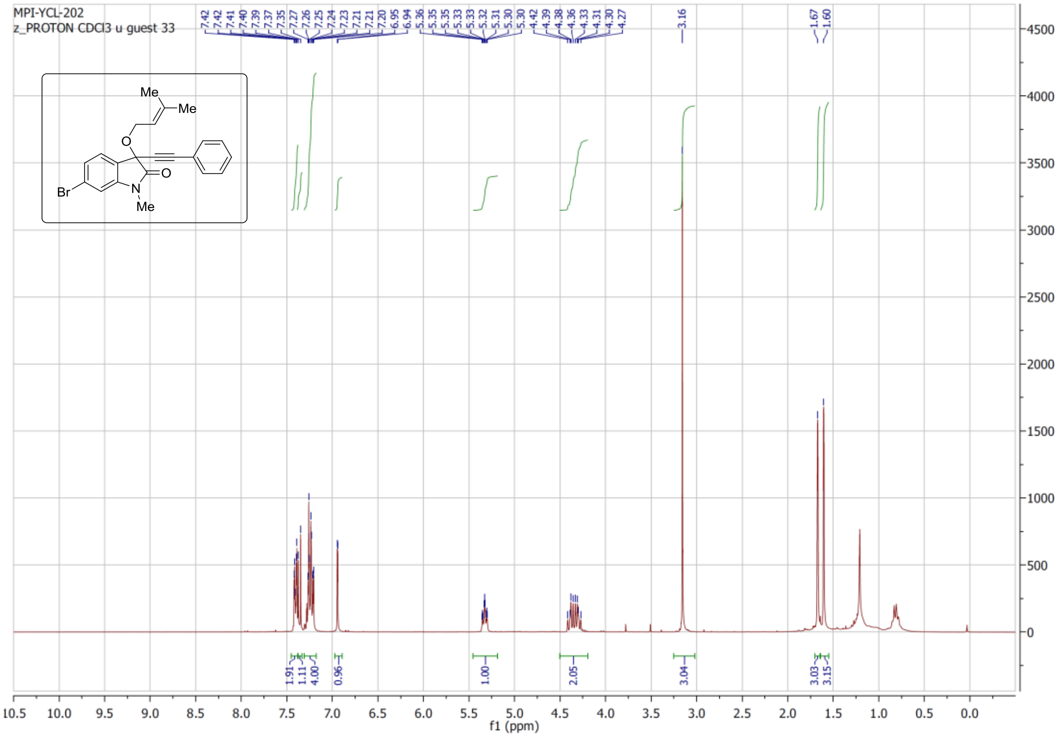

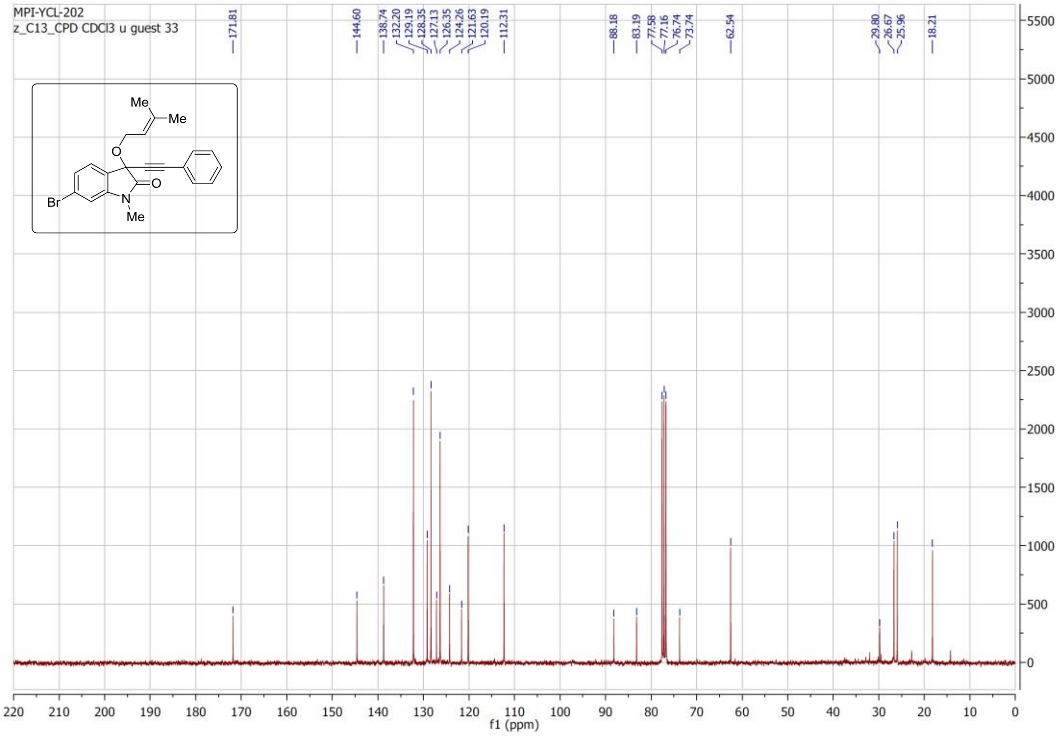

Supplementary Figure 63. ^{1}H and ^{13}C NMR spectra for **13l**.

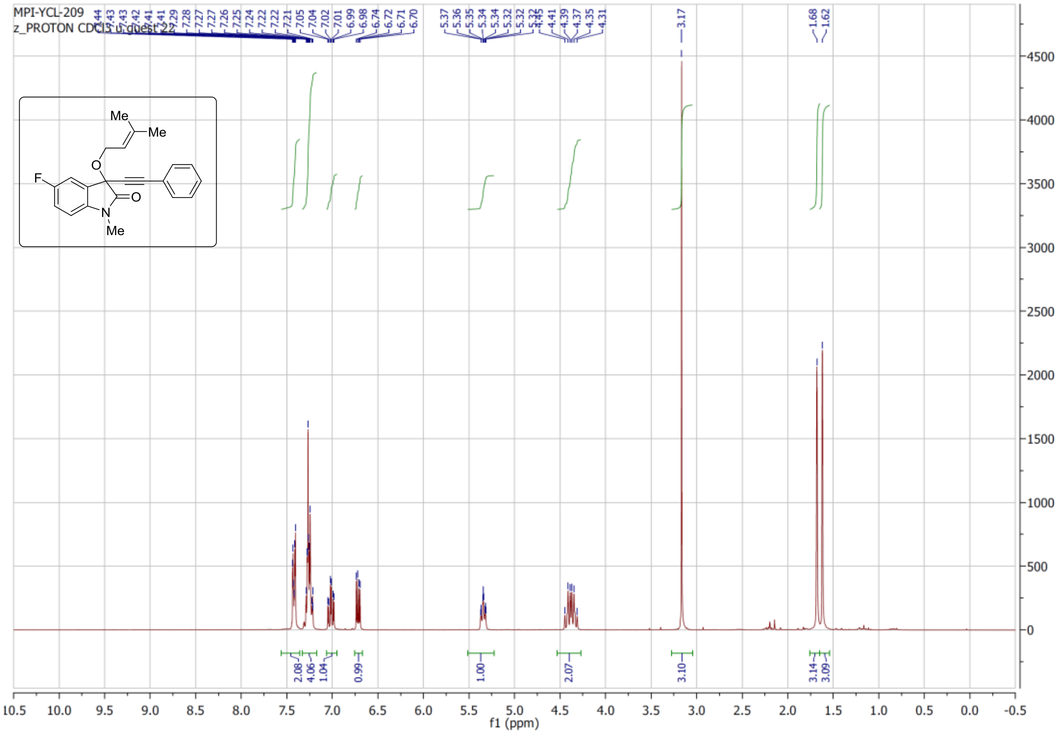

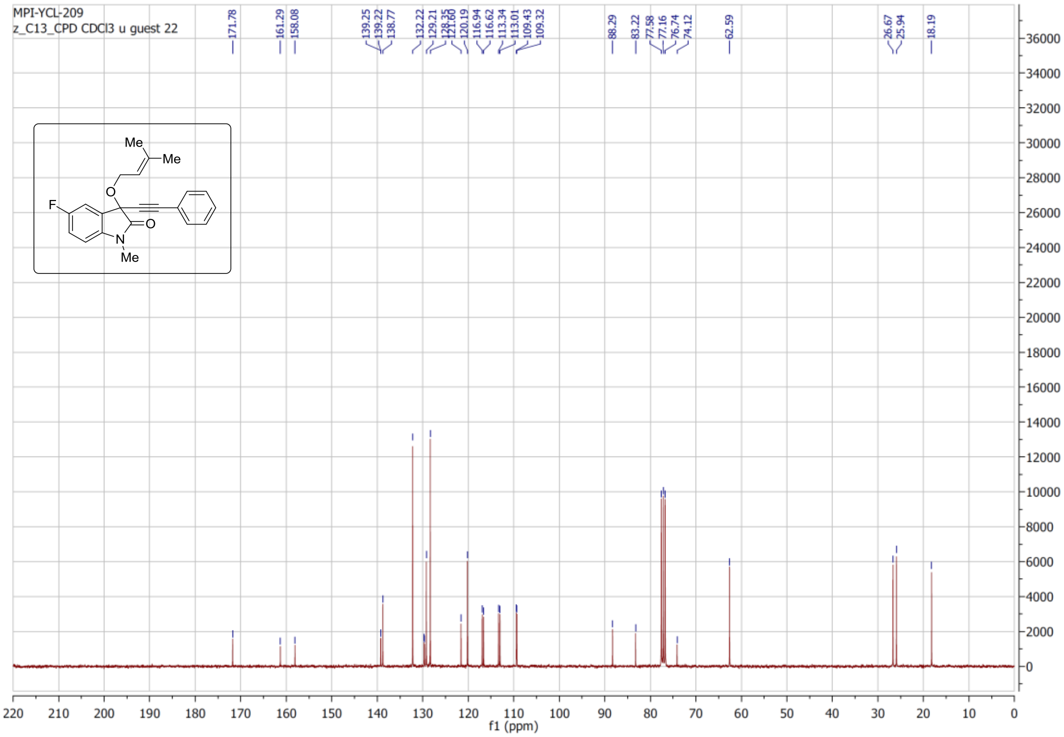

Supplementary Figure 64. ^{1}H and ^{13}C NMR spectra for **13m**.

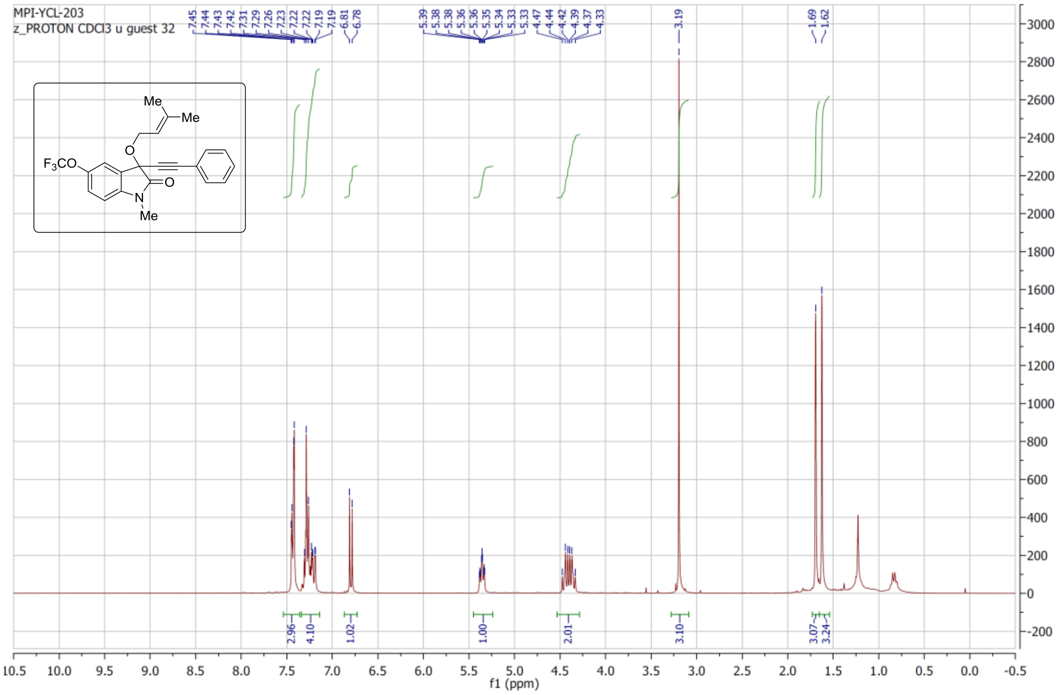

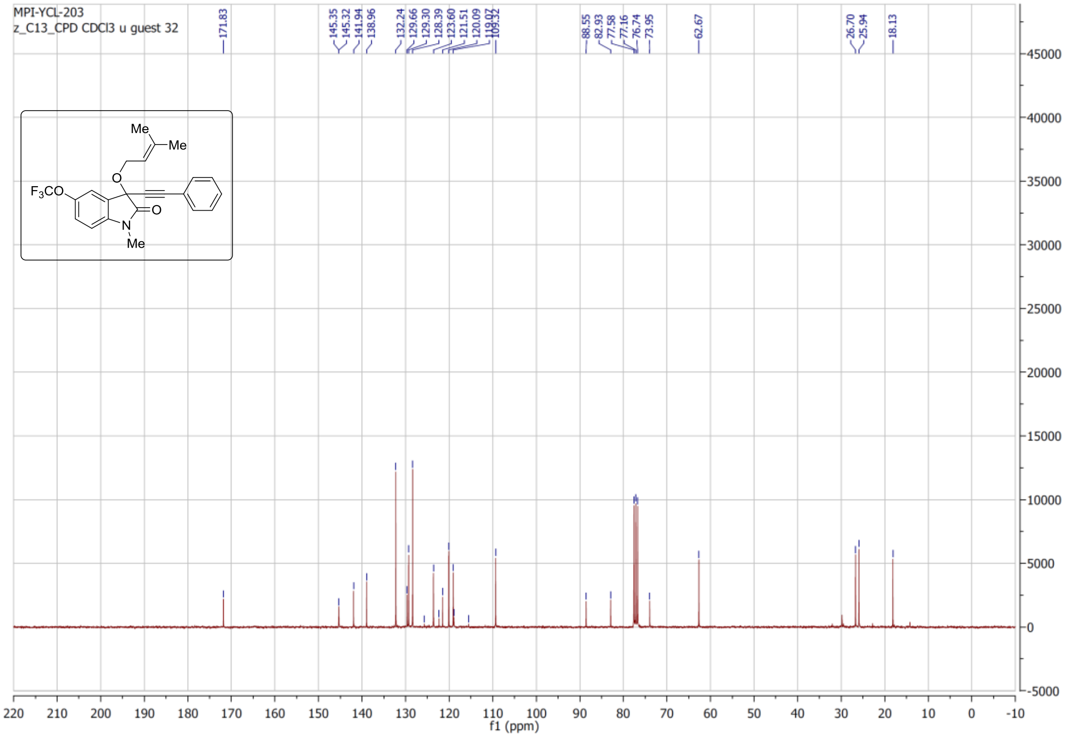

Supplementary Figure 65. ^{1}H and ^{13}C NMR spectra for **13n**.

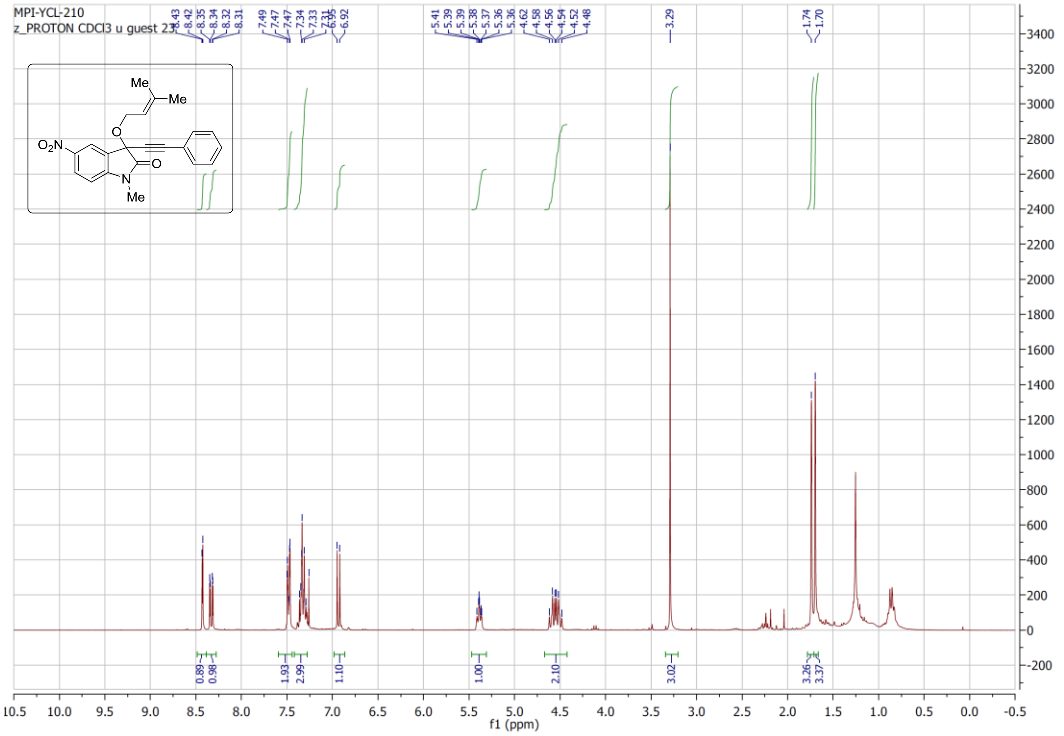

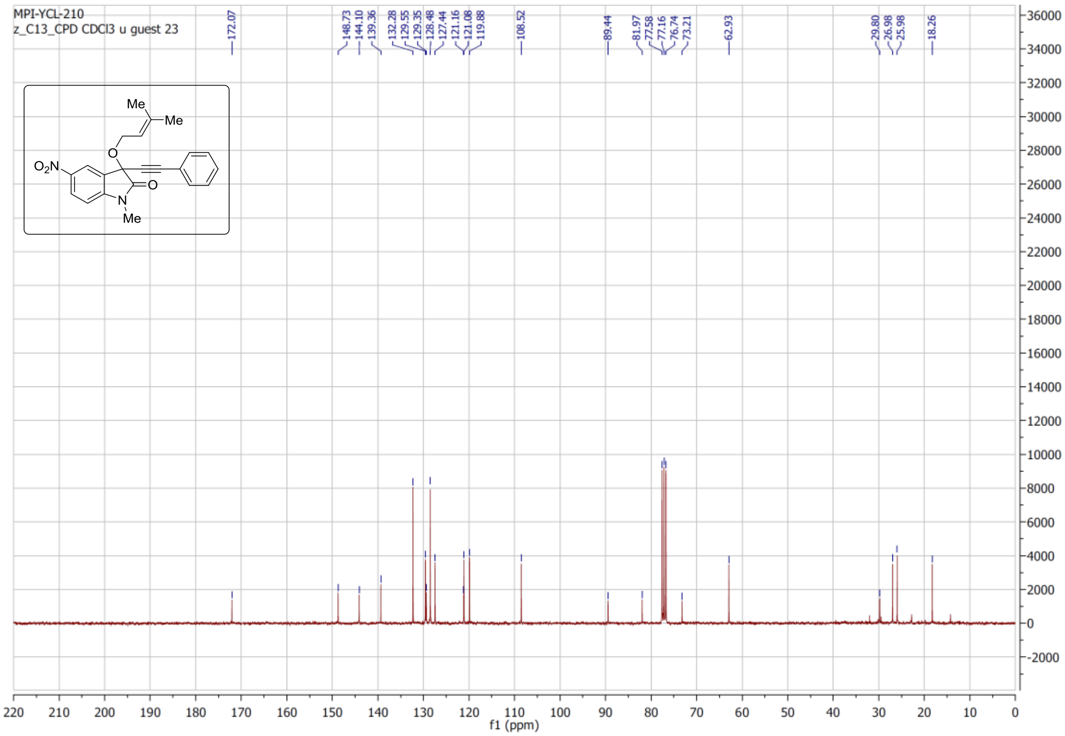

Supplementary Figure 66. ^{1}H and ^{13}C NMR spectra for **13o**.

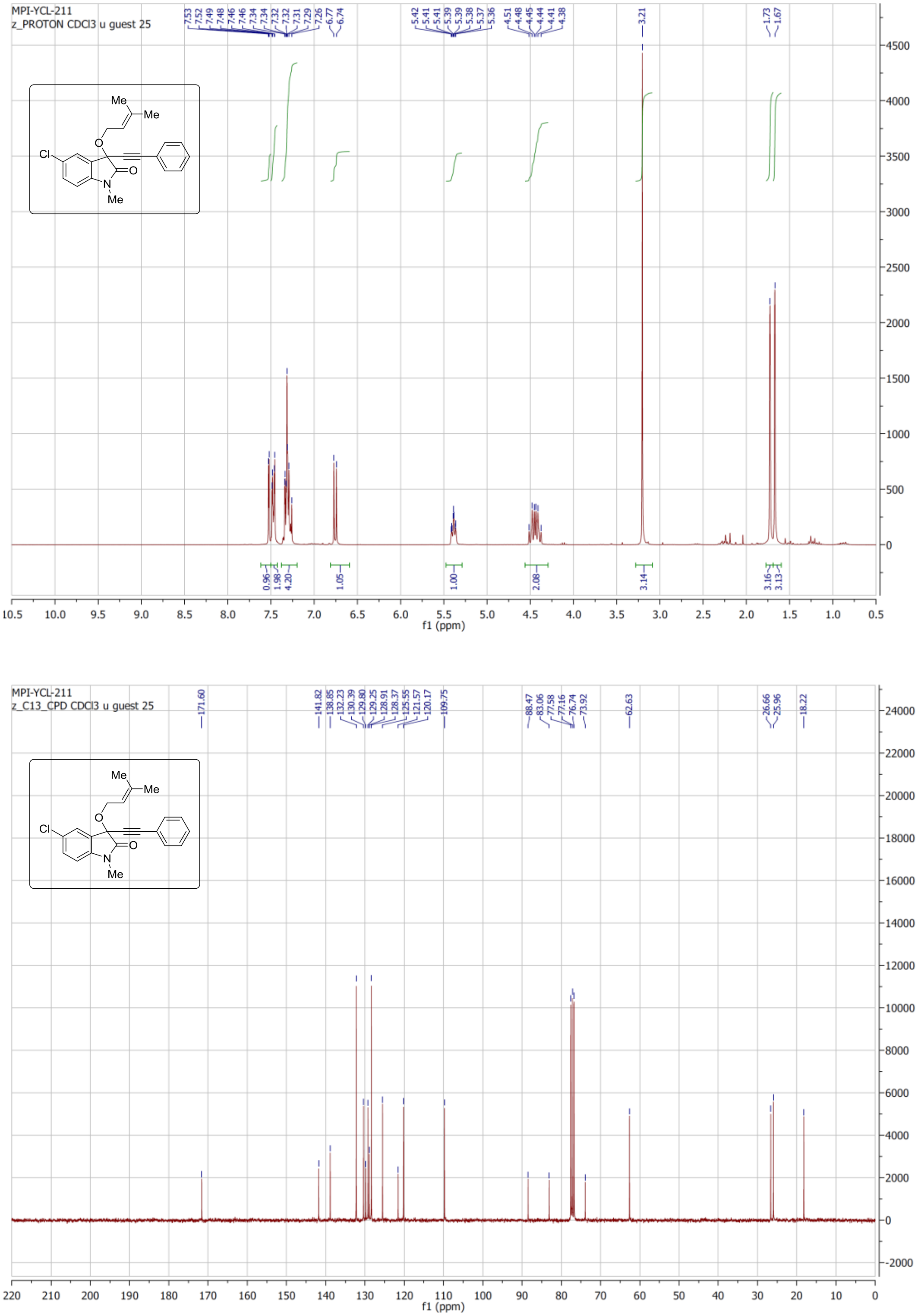

Supplementary Figure 67. ^{1}H and ^{13}C NMR spectra for **13p**.

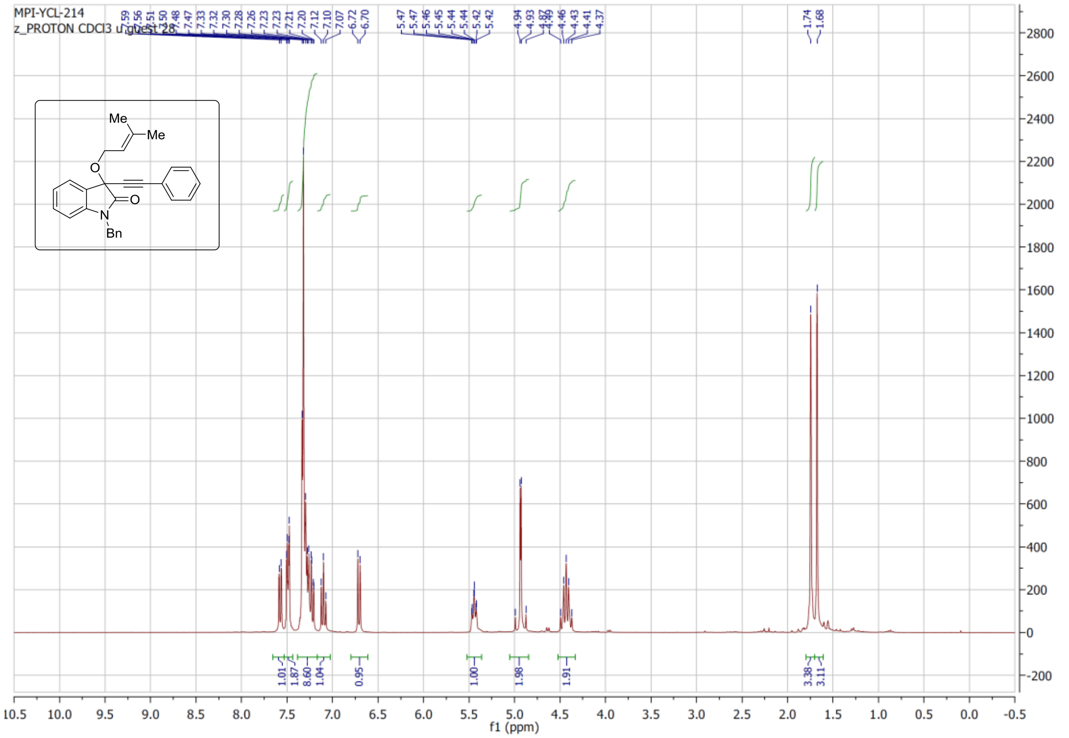

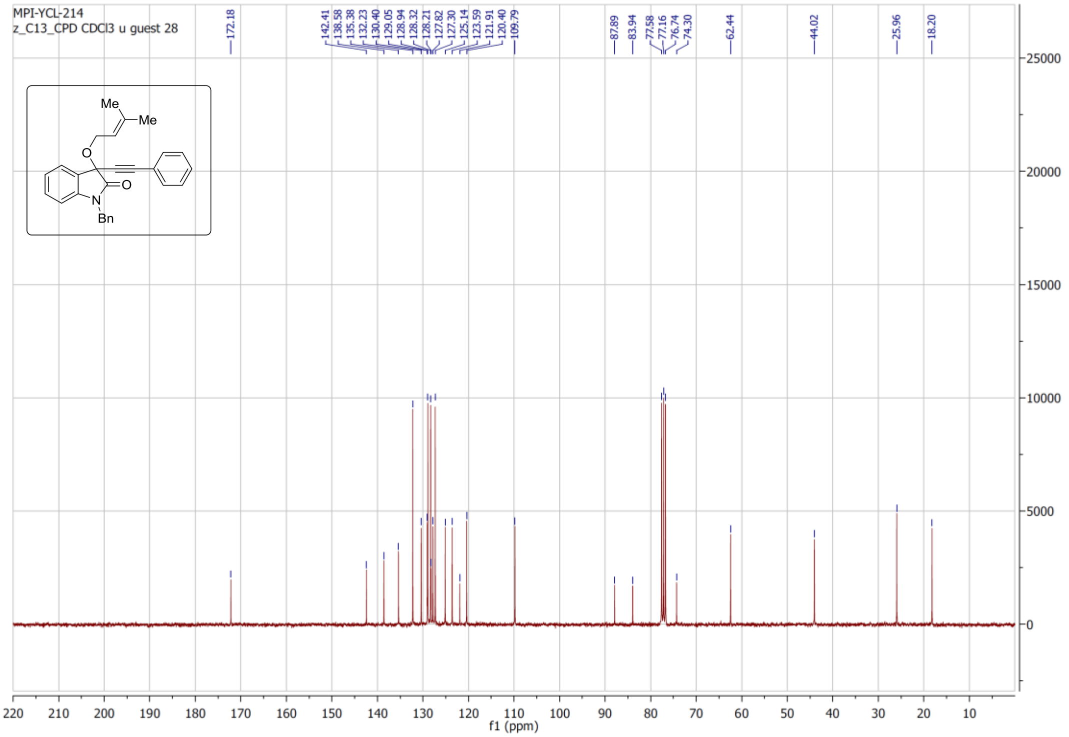

Supplementary Figure 68. ^{1}H and ^{13}C NMR spectra for **13q**.

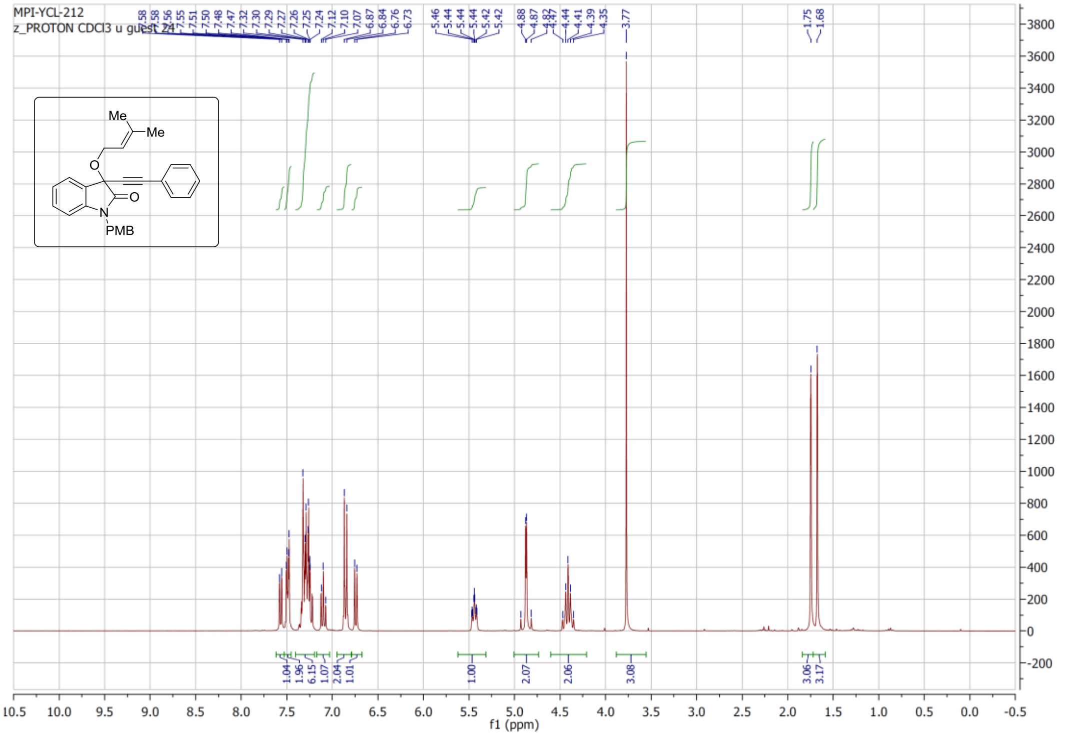

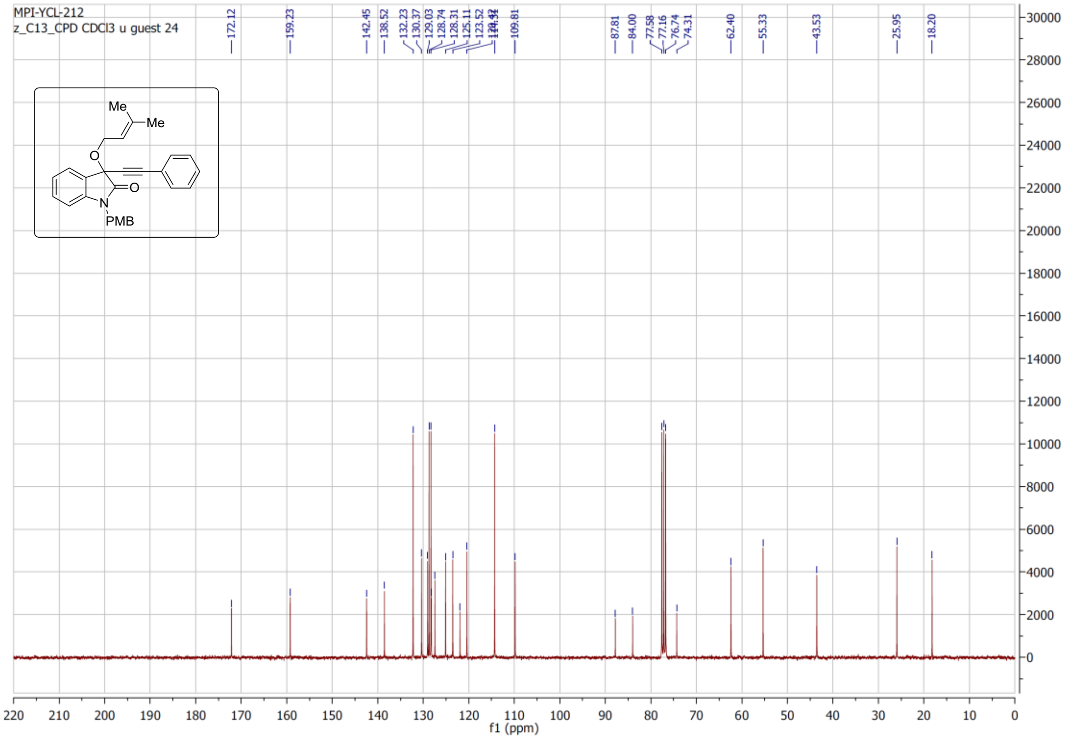

Supplementary Figure 69. ^{1}H and ^{13}C NMR spectra for **13r**.

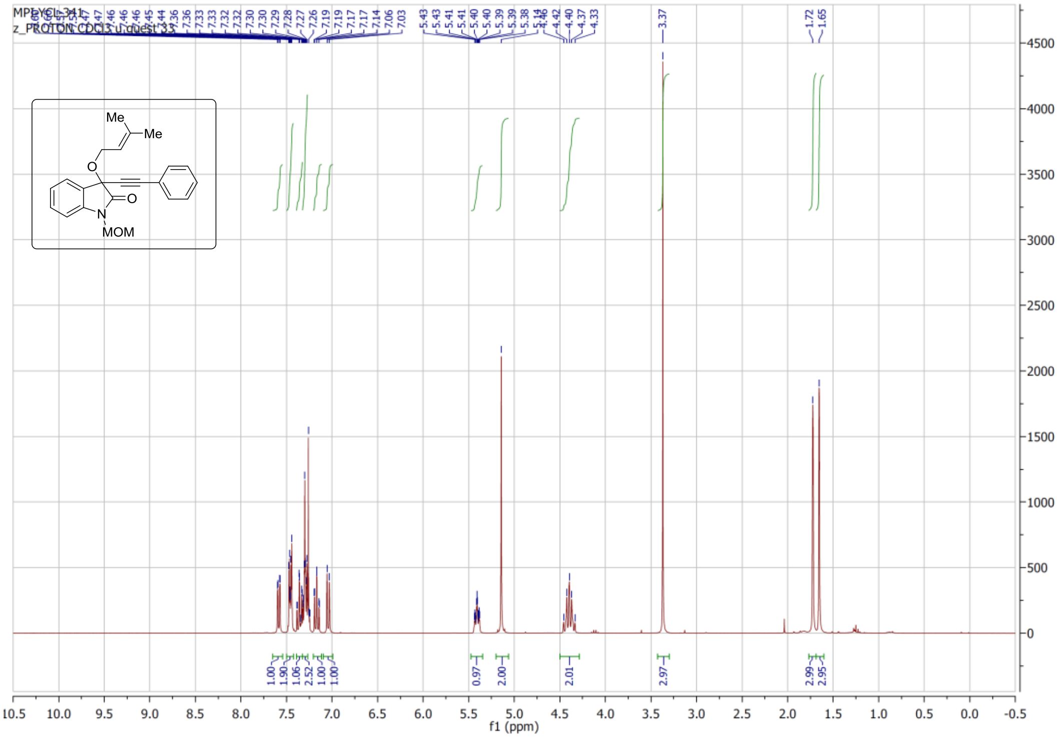

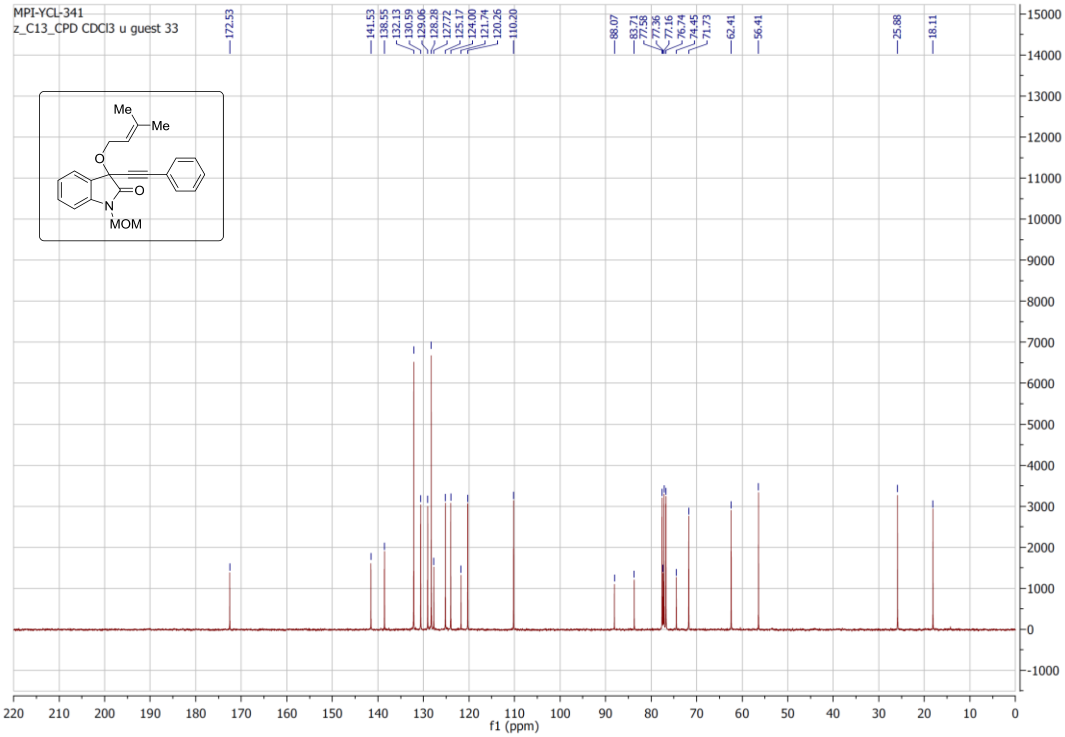

Supplementary Figure 70. ^{1}H and ^{13}C NMR spectra for **13s**.

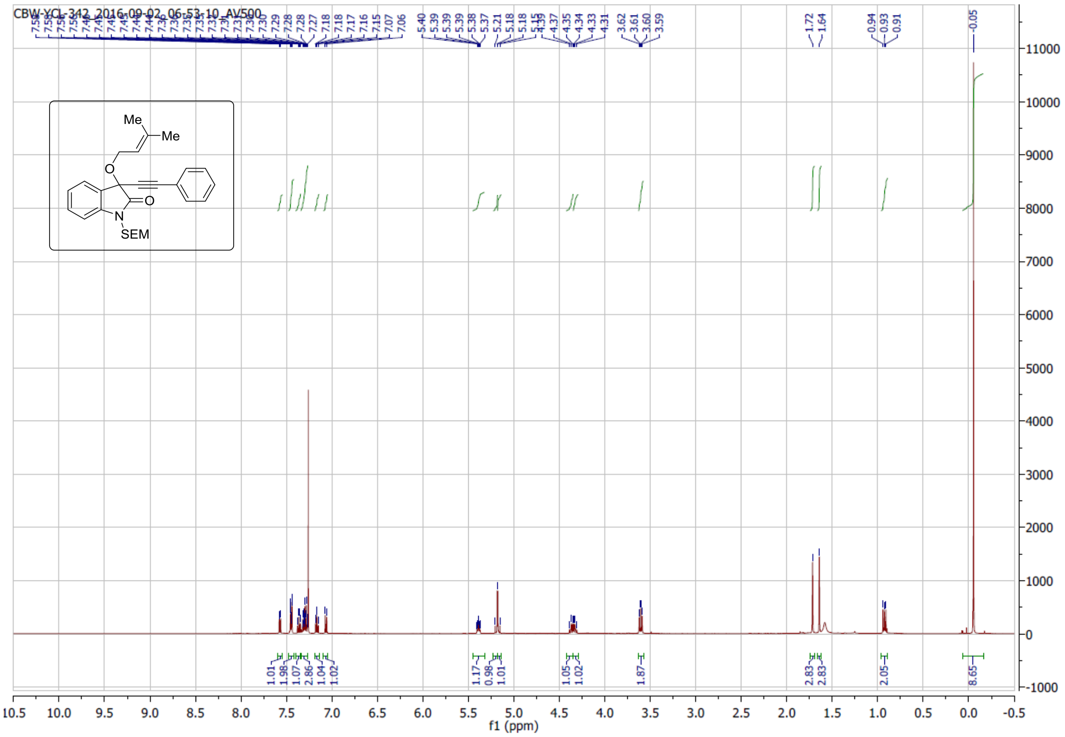

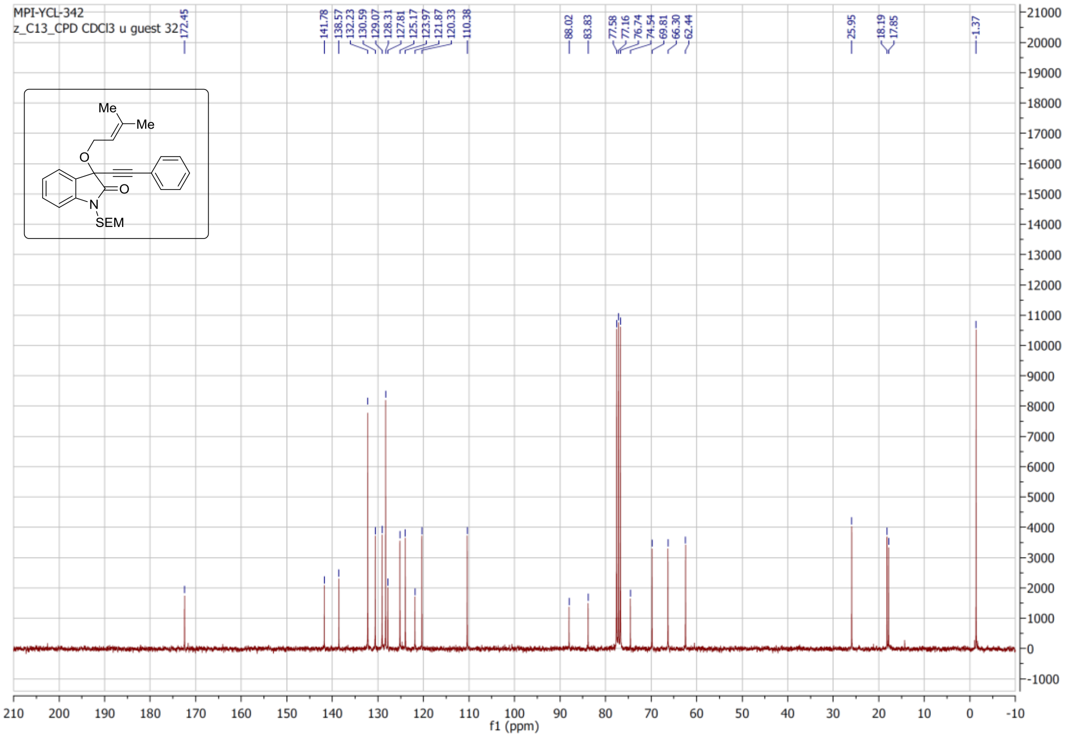

Supplementary Figure 71. ^{1}H and ^{13}C NMR spectra for **13t**.

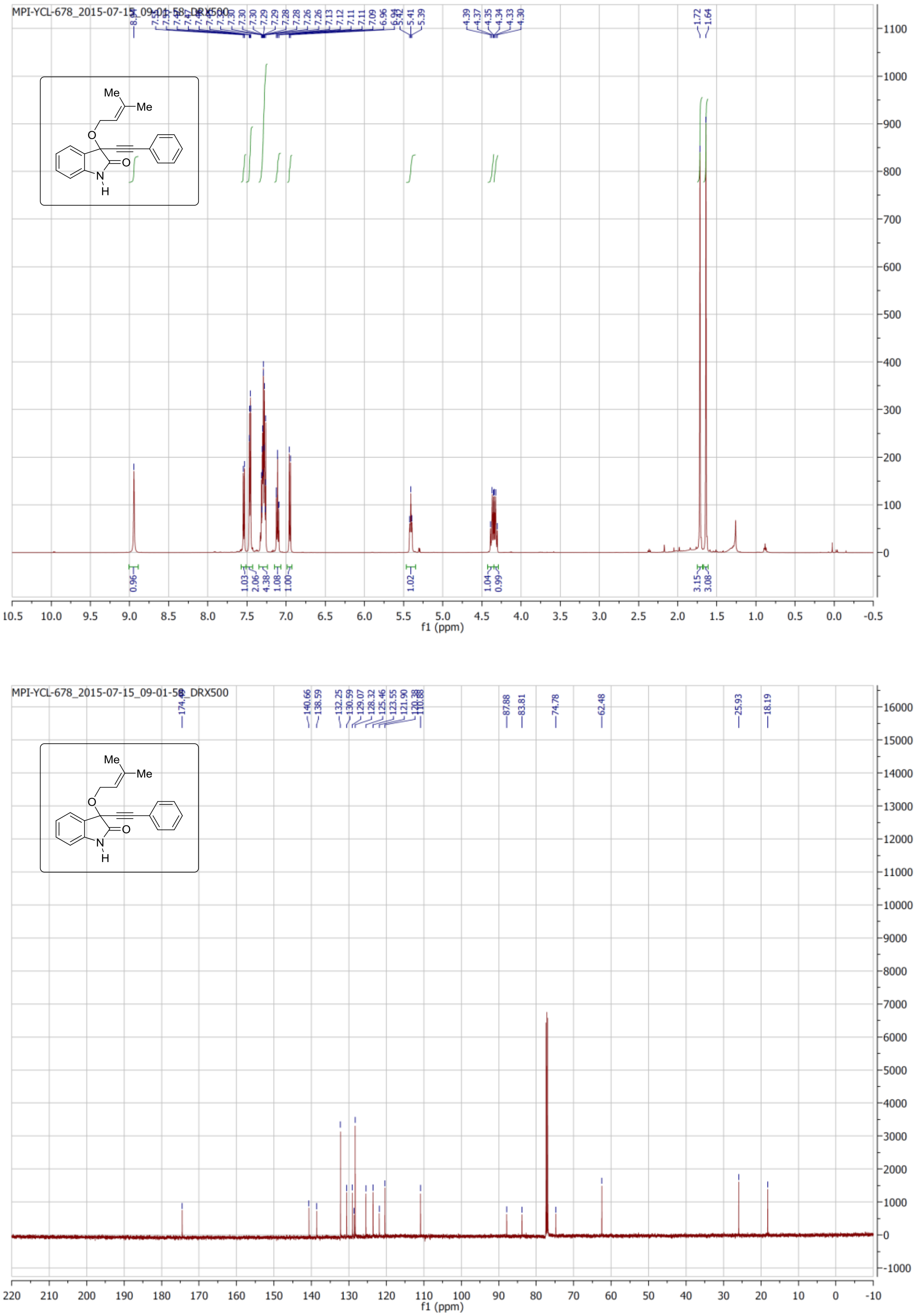

Supplementary Figure 72. ^{1}H and ^{13}C NMR spectra for **13u**.

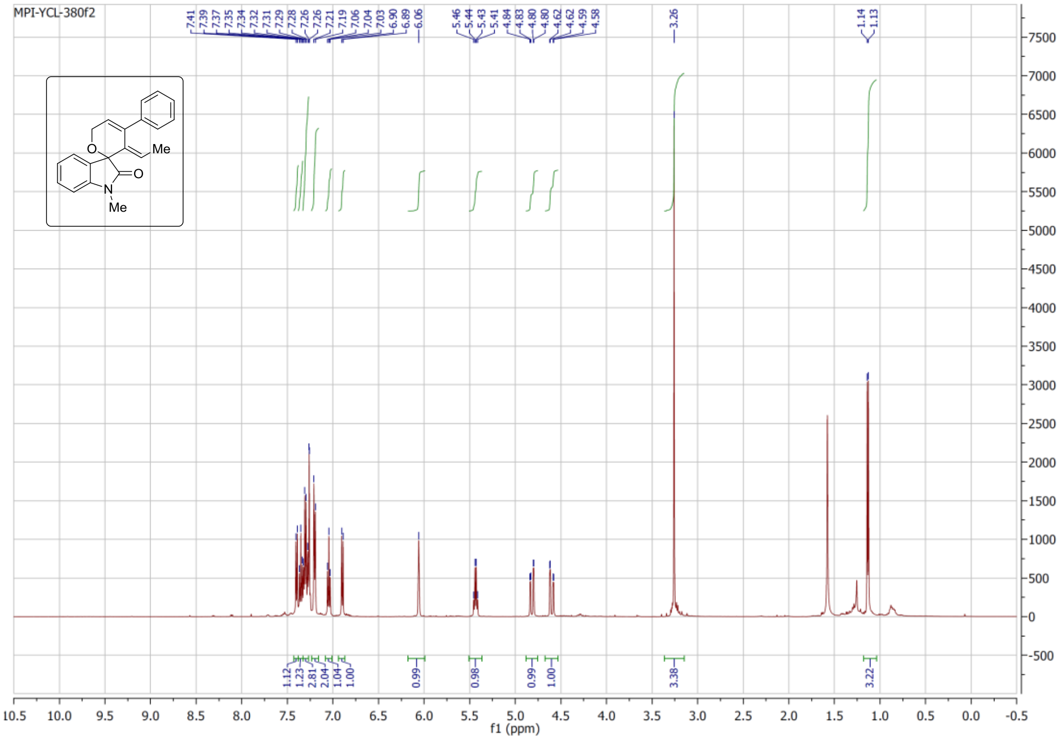

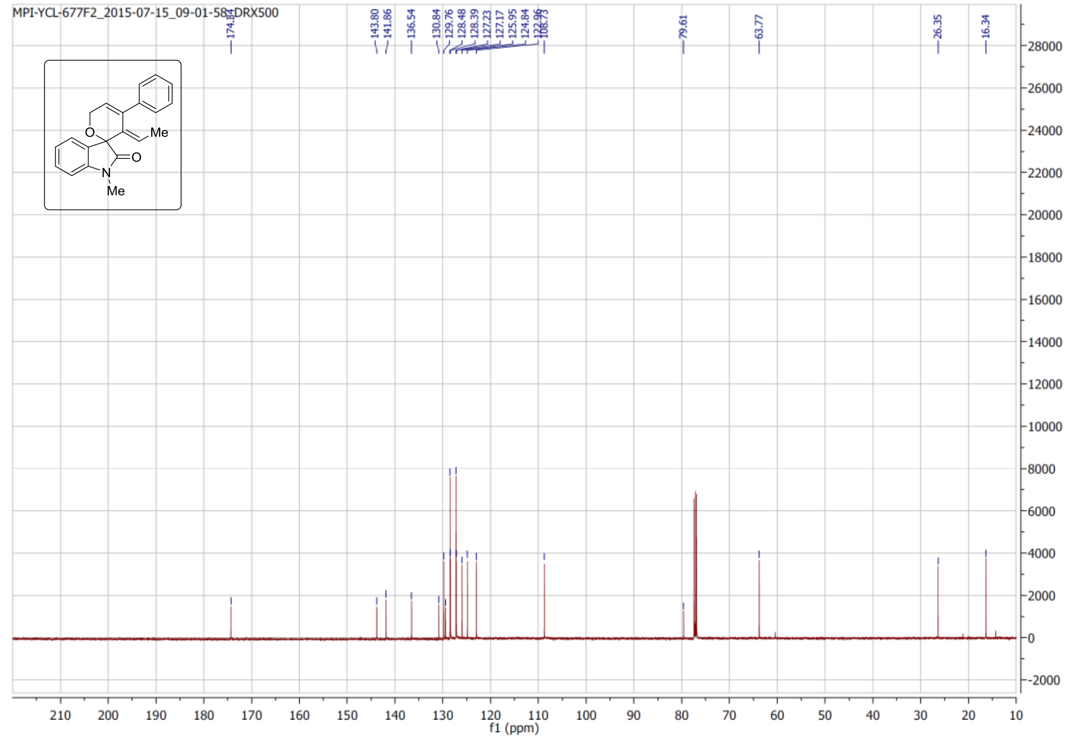

Supplementary Figure 73. ^{1}H and ^{13}C NMR spectra for **2a**.

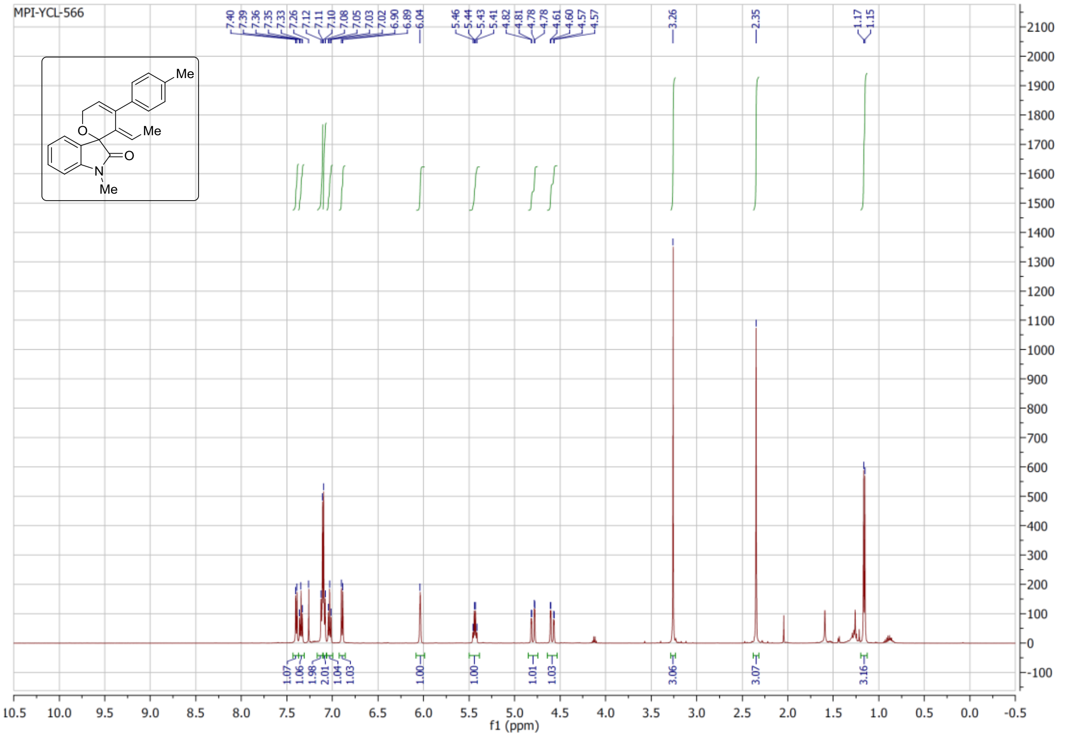

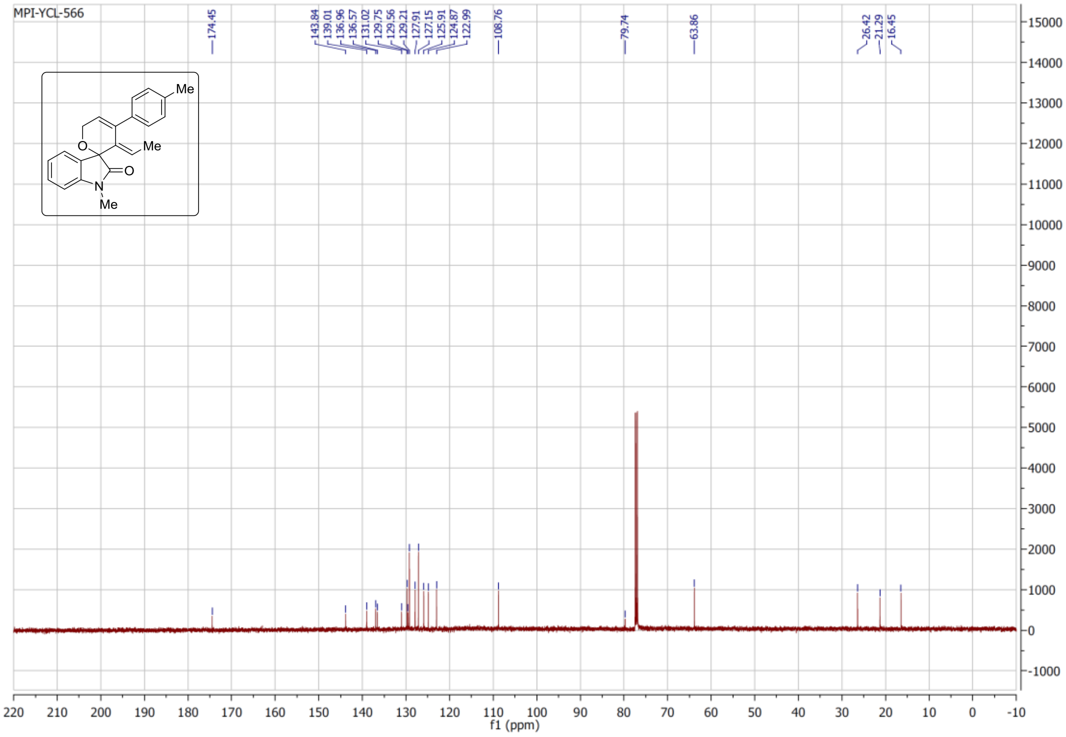

Supplementary Figure 74. ^{1}H and ^{13}C NMR spectra for **2b**.

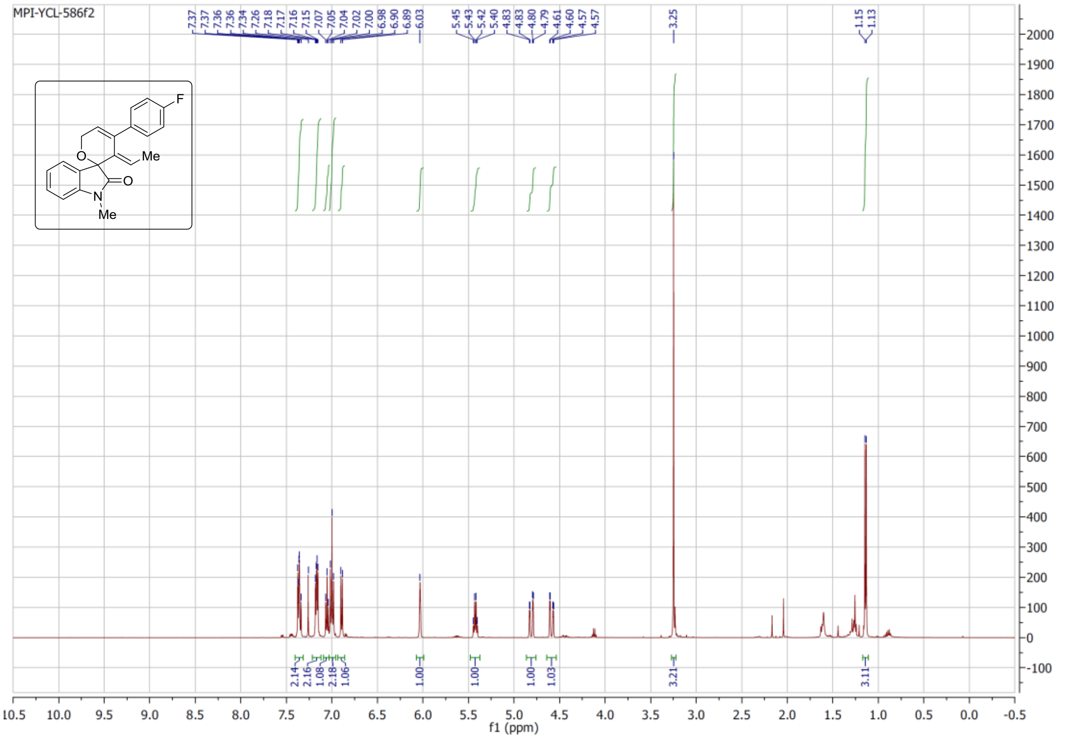

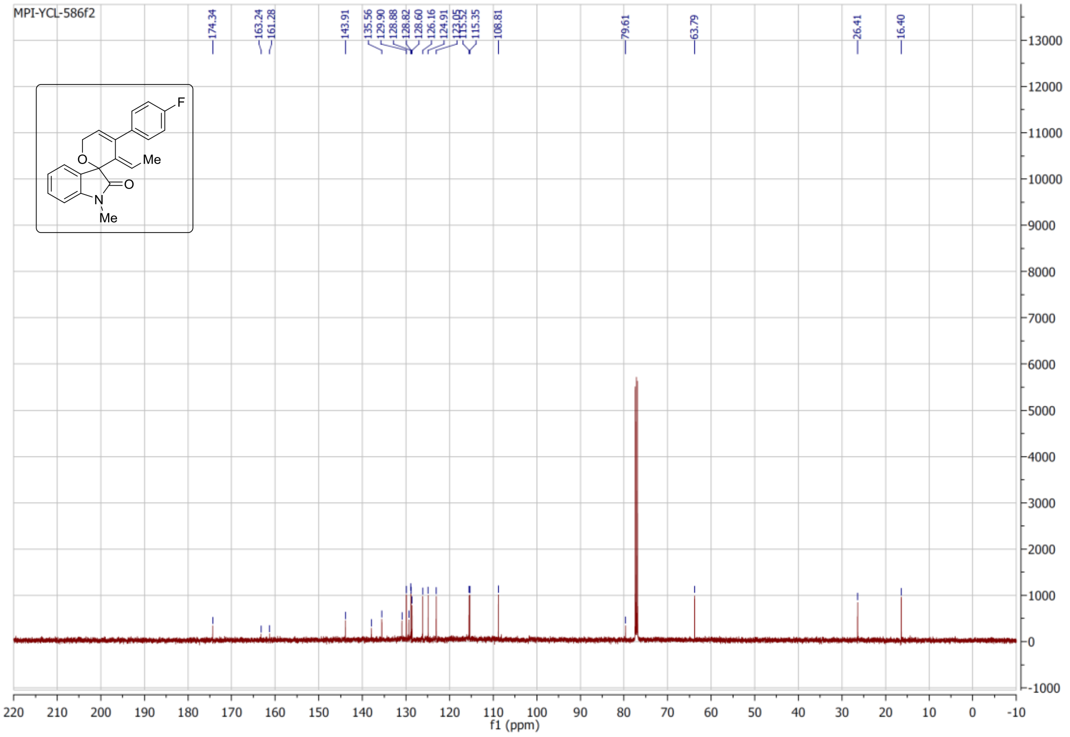

Supplementary Figure 75. ^{1}H and ^{13}C NMR spectra for **2c**.

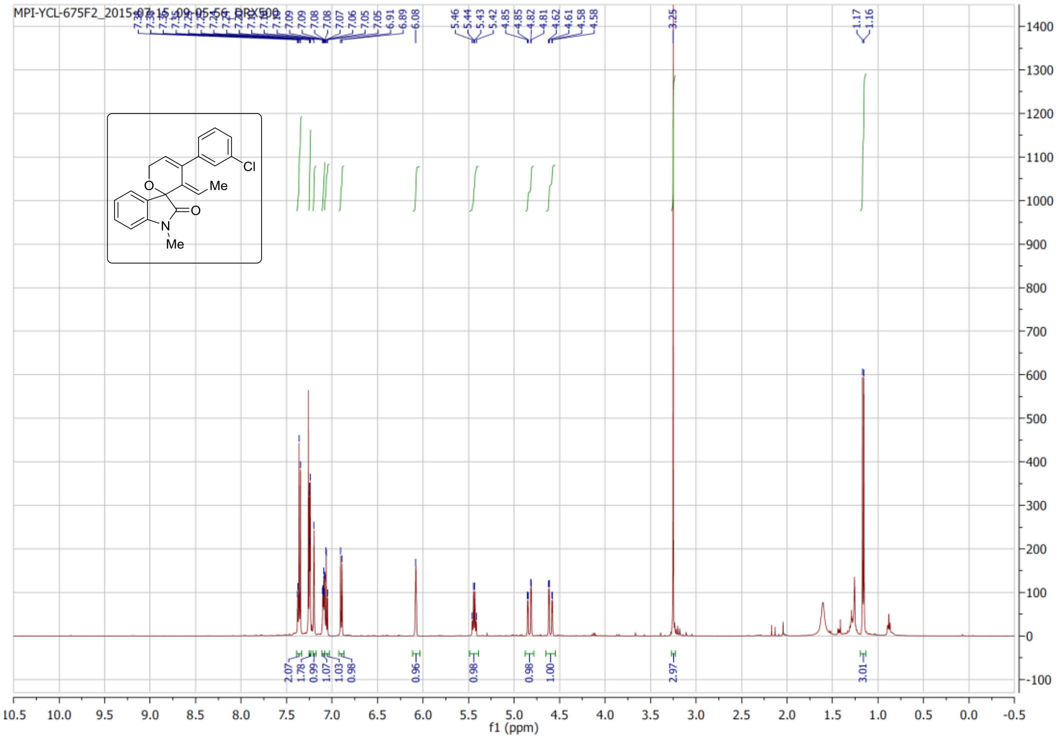

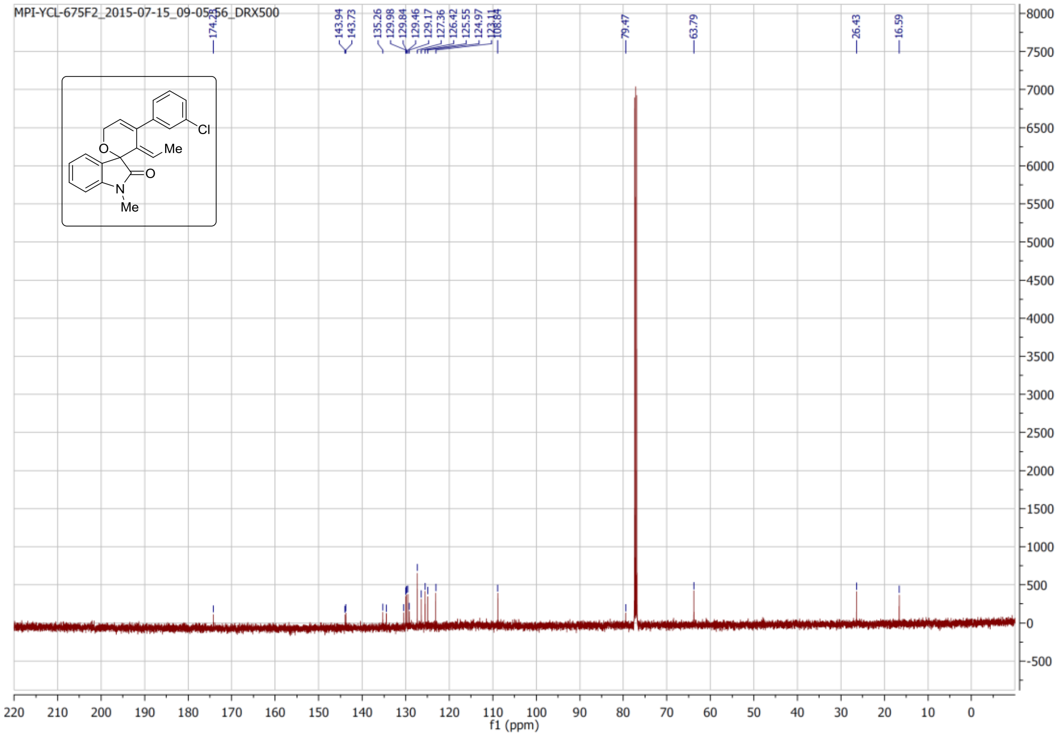

Supplementary Figure 76. ^{1}H and ^{13}C NMR spectra for **2d**.

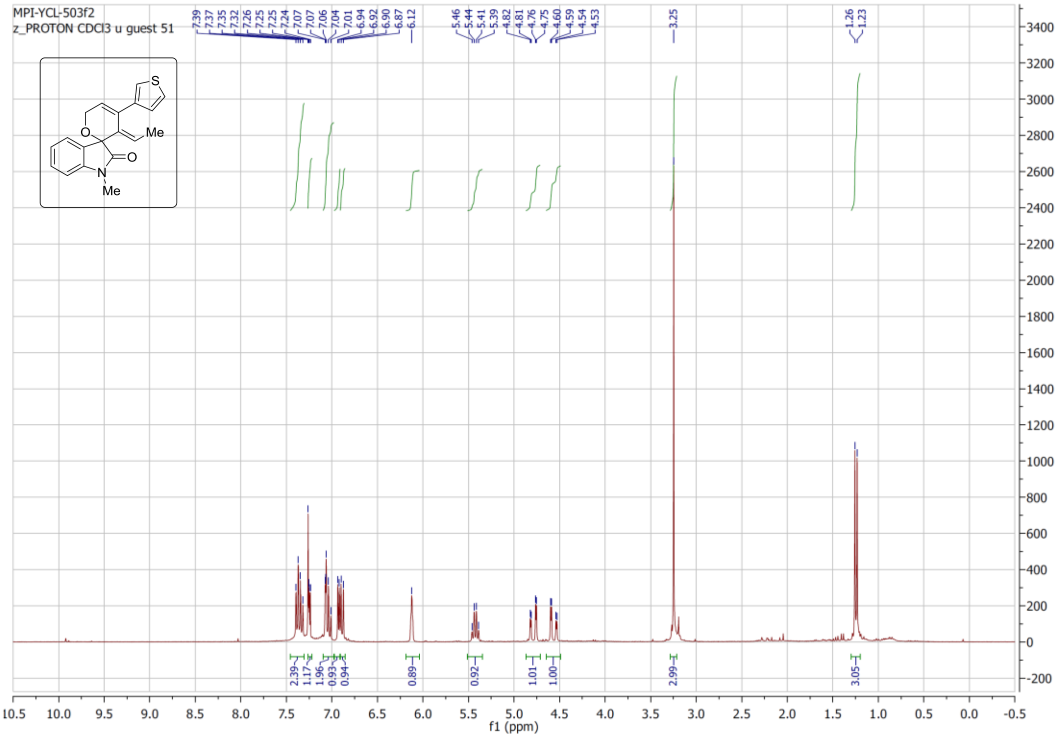

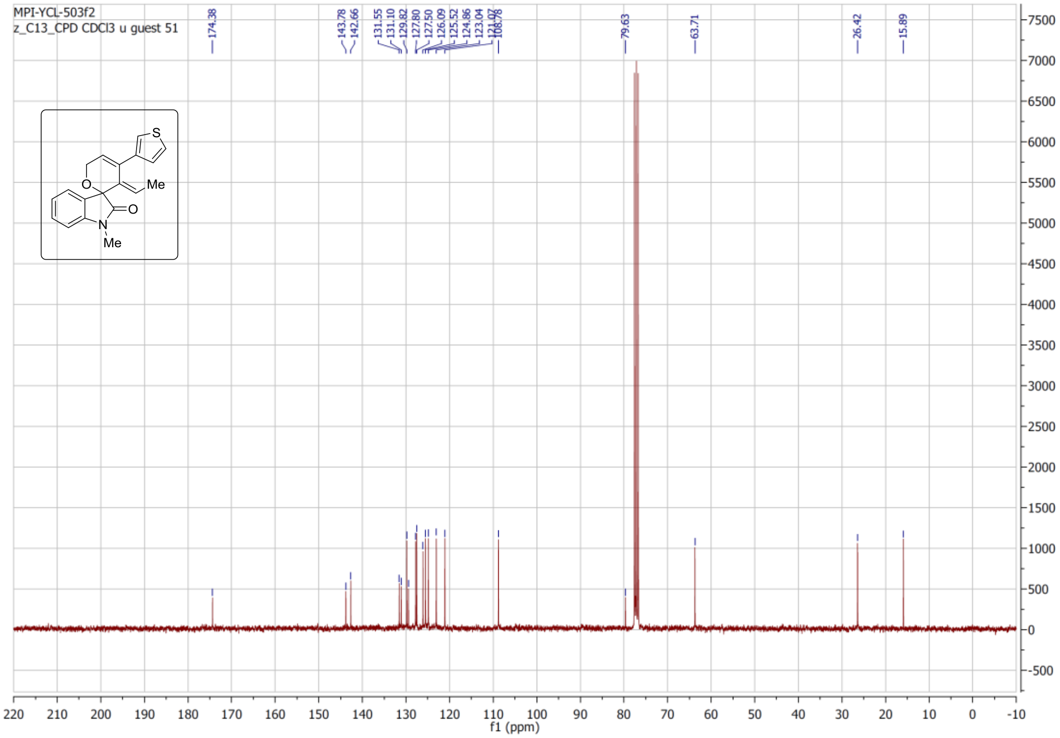

Supplementary Figure 77. ^{1}H and ^{13}C NMR spectra for **2e**.

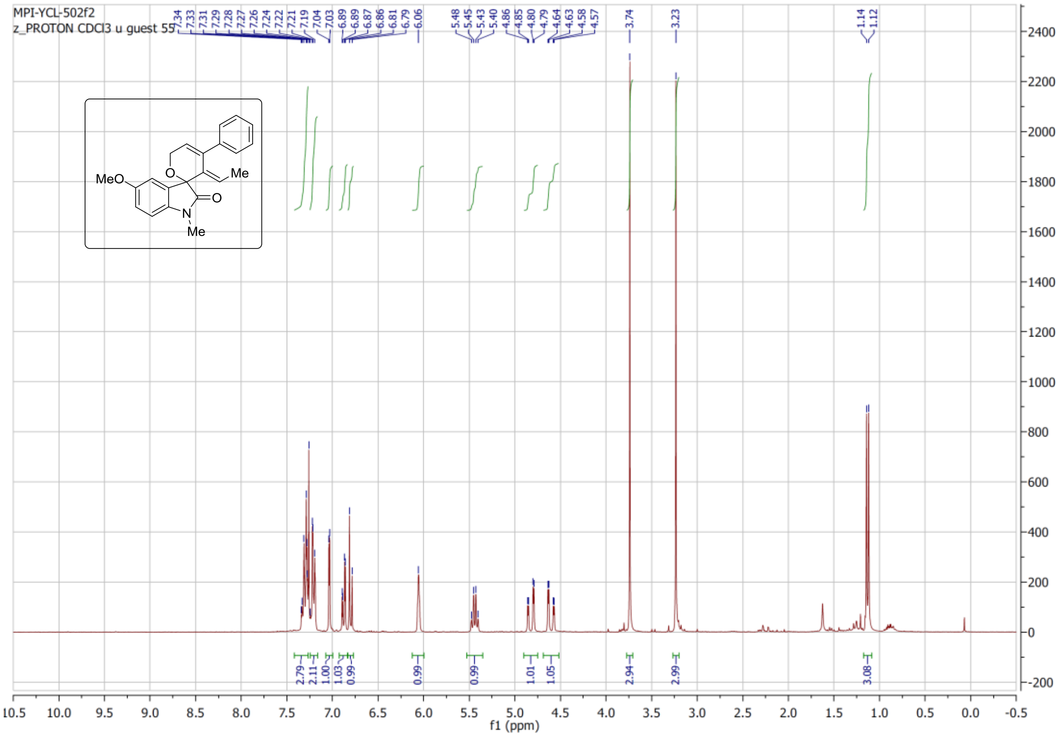

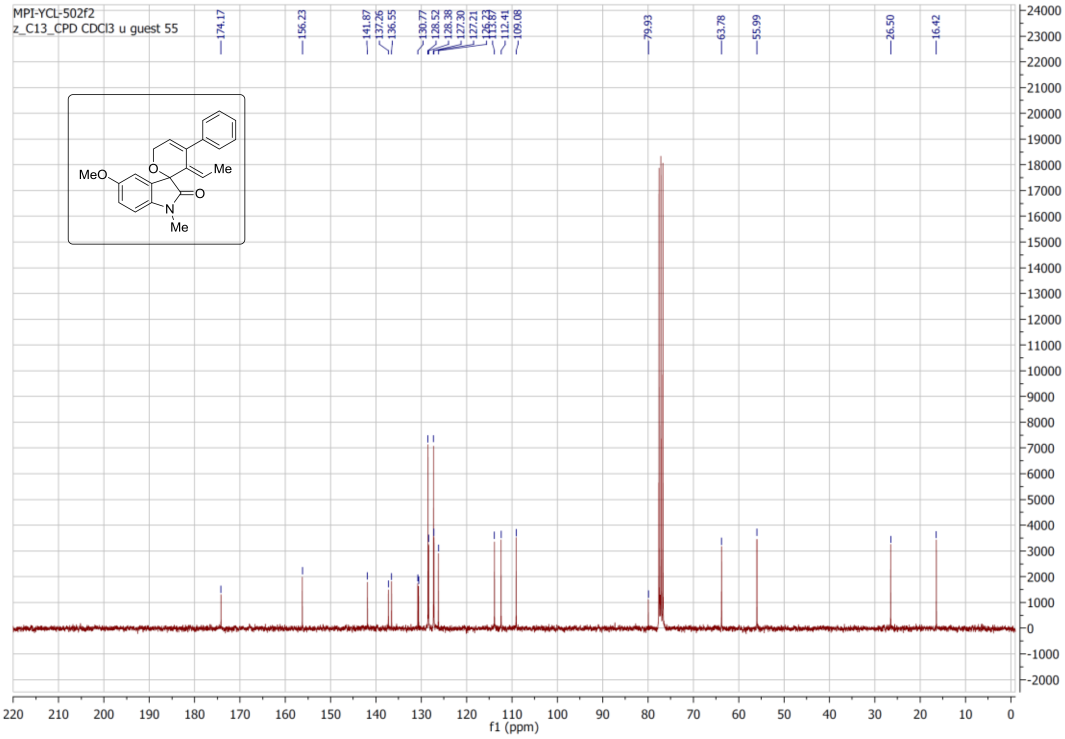

Supplementary Figure 78. ^{1}H and ^{13}C NMR spectra for **2f**.

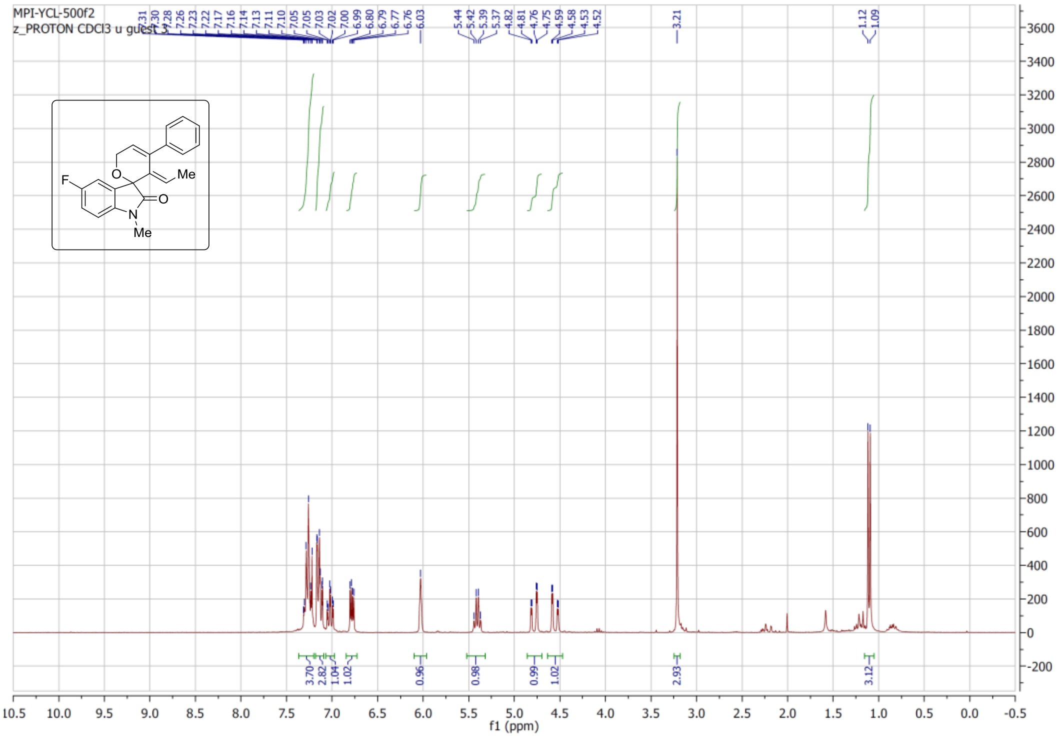

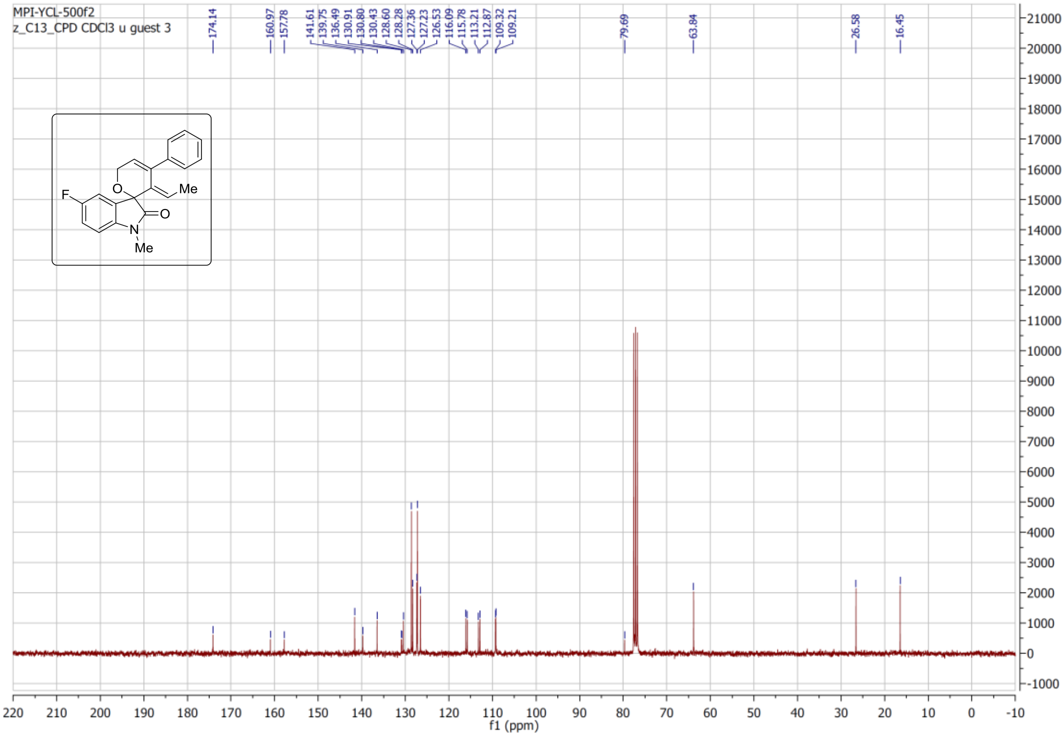

Supplementary Figure 79. ^{1}H and ^{13}C NMR spectra for **2g**.

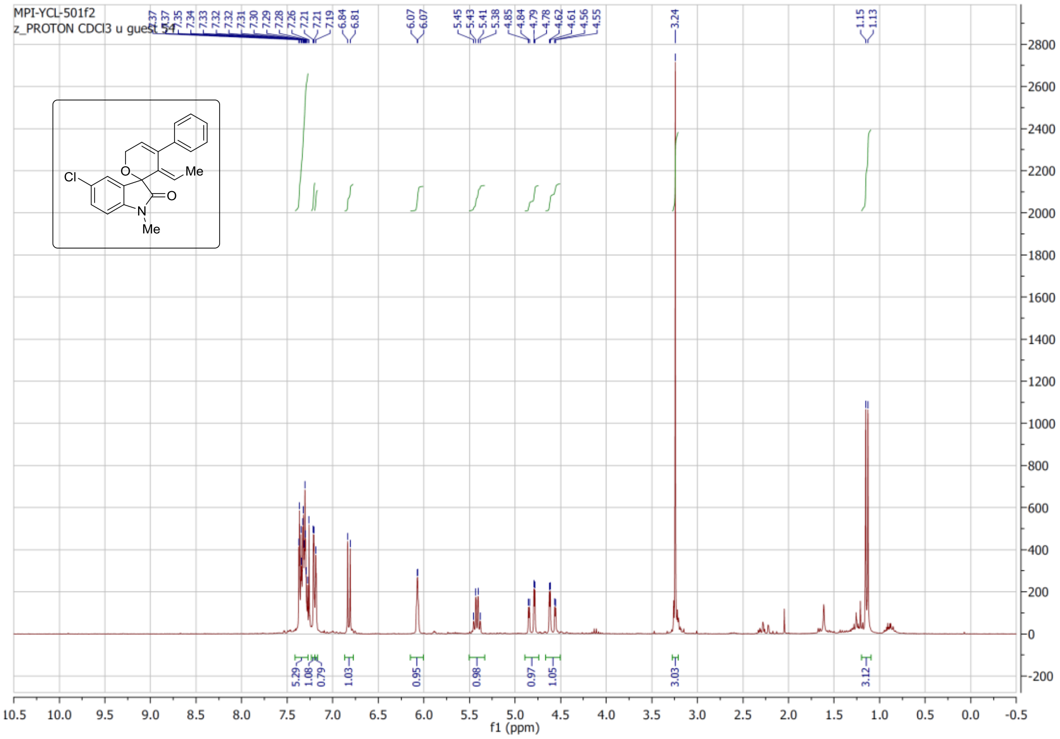

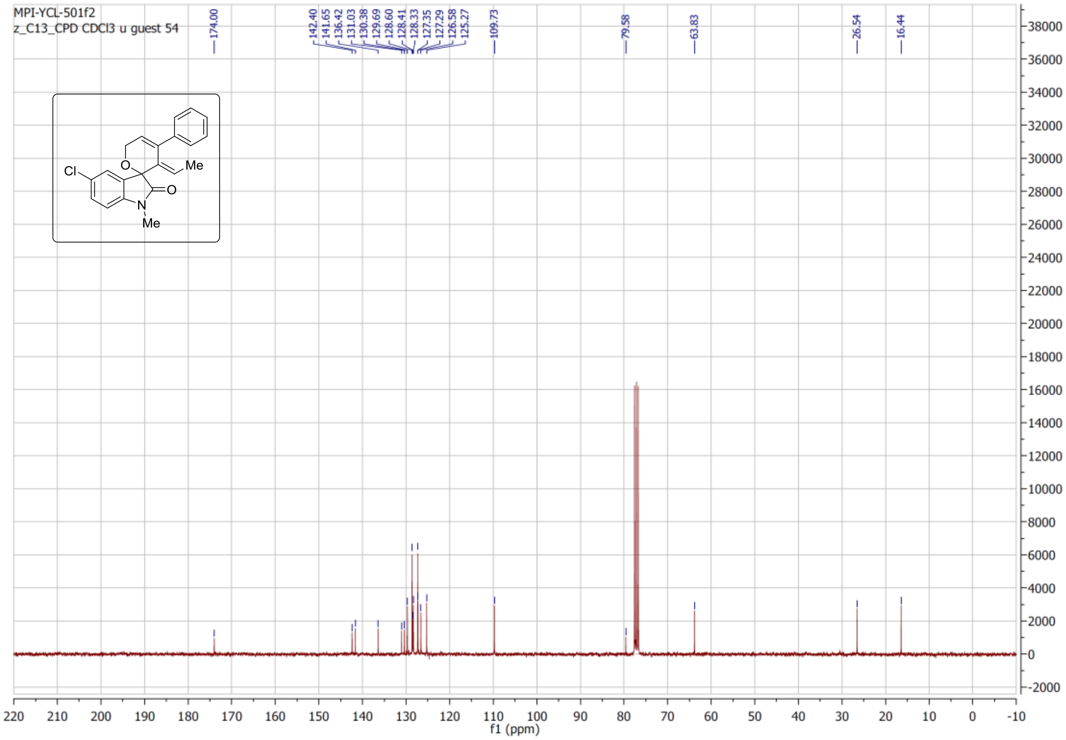

Supplementary Figure 80. ^{1}H and ^{13}C NMR spectra for **2h**.

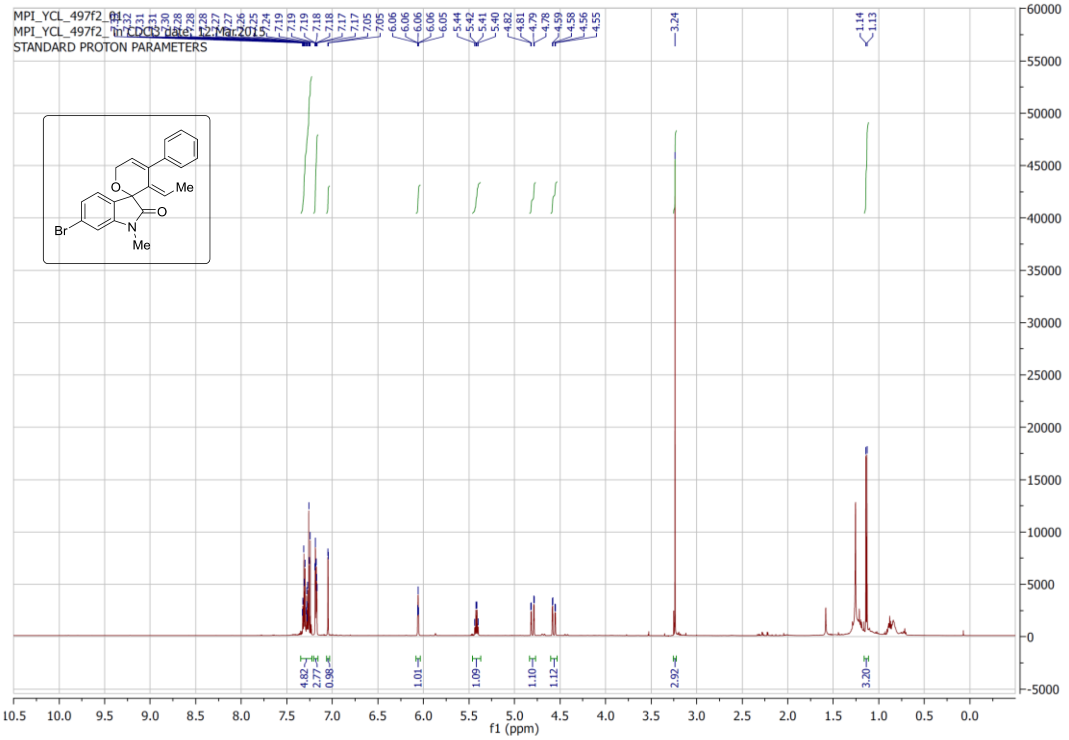

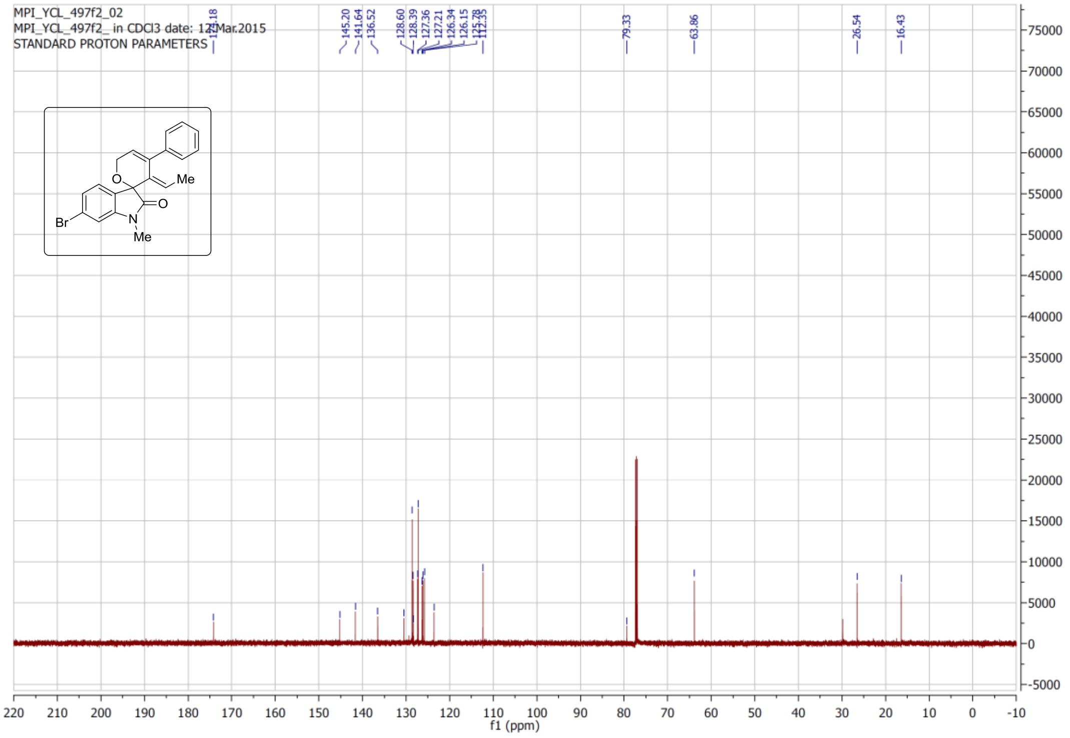

Supplementary Figure 81. ^{1}H and ^{13}C NMR spectra for **2i**.

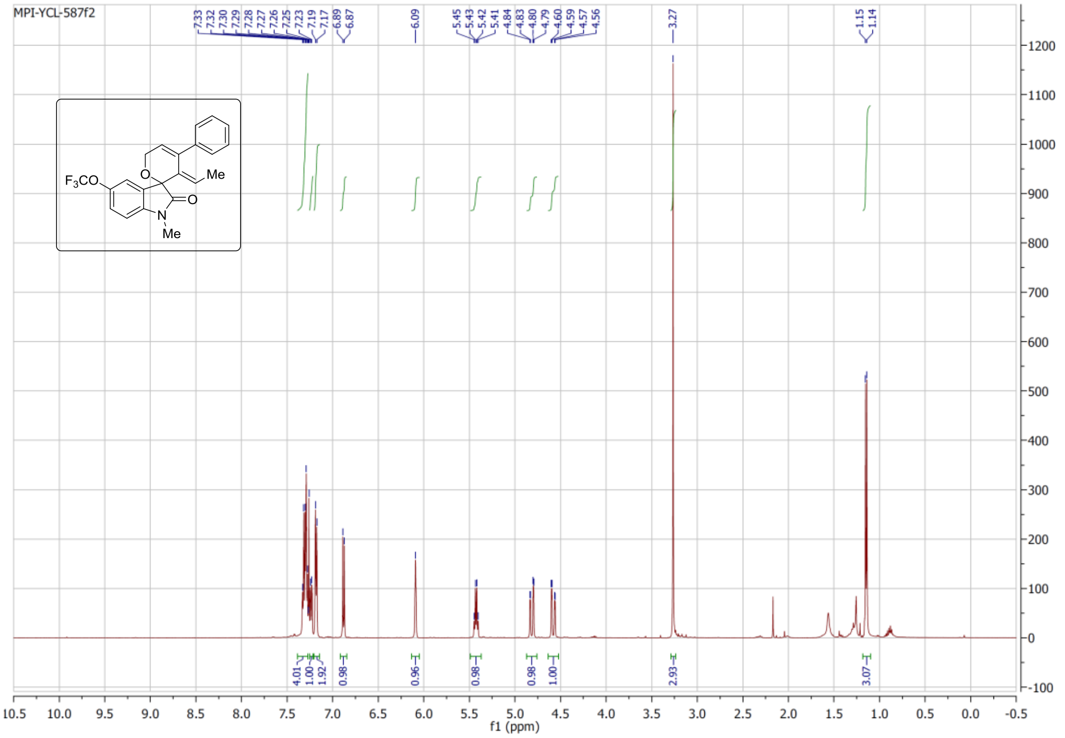

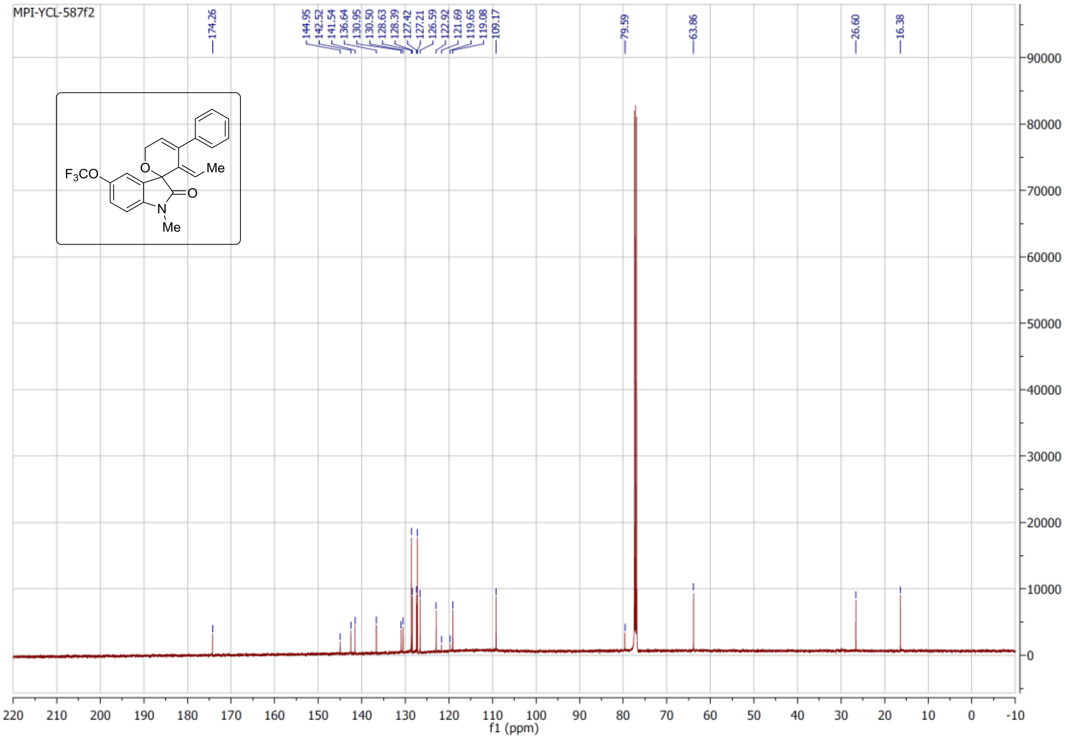

Supplementary Figure 82. ^{1}H and ^{13}C NMR spectra for **2j**.

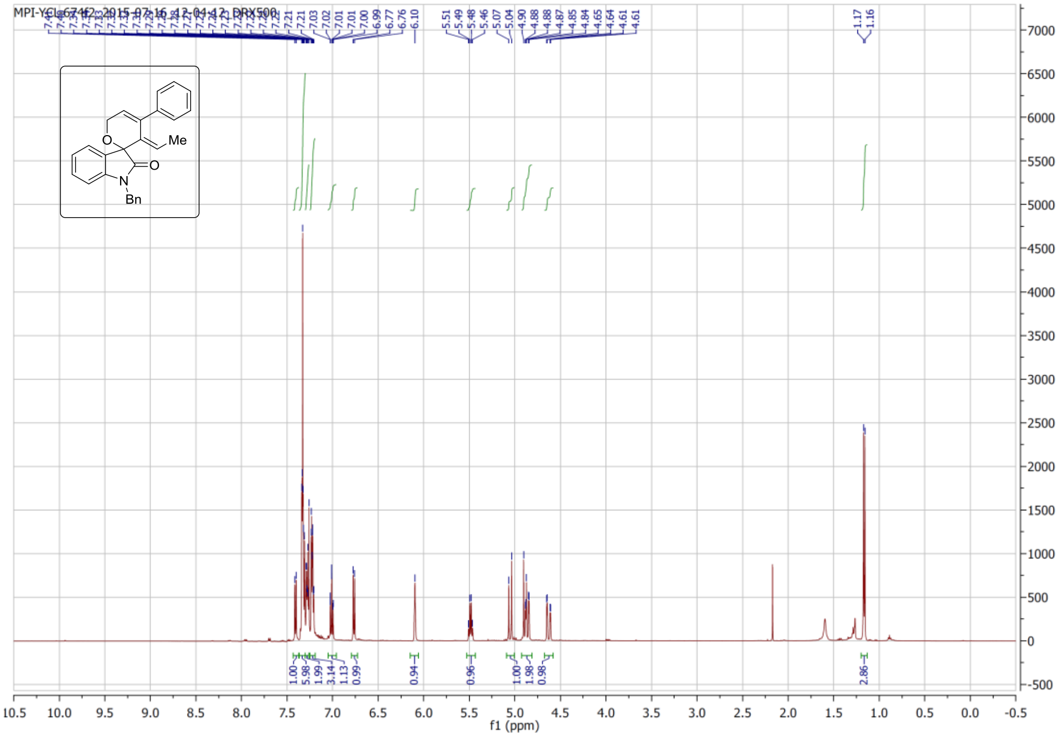

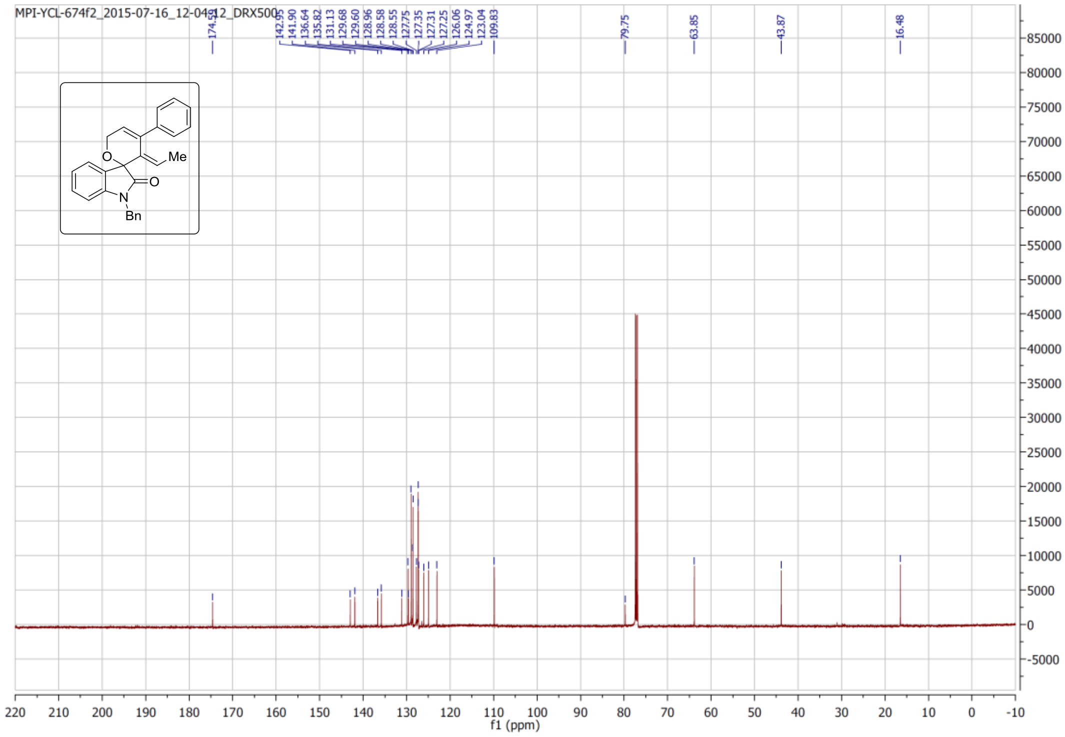

Supplementary Figure 83. ^{1}H and ^{13}C NMR spectra for **2k**.

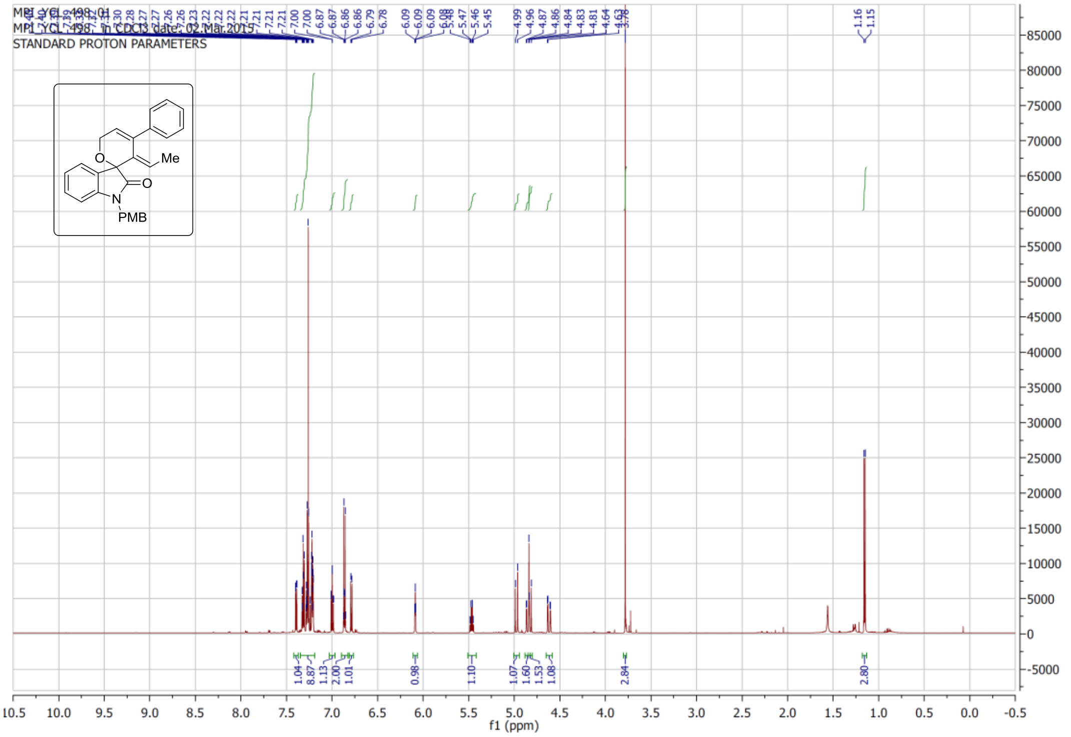

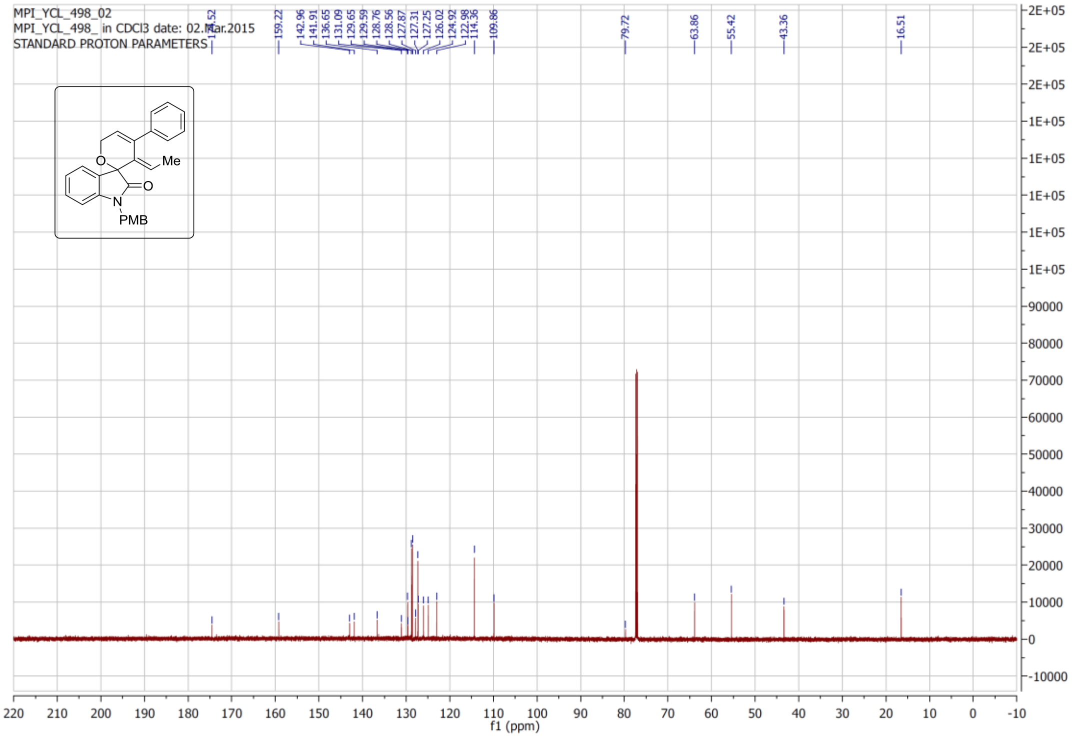

Supplementary Figure 84. ^{1}H and ^{13}C NMR spectra for **2l**.

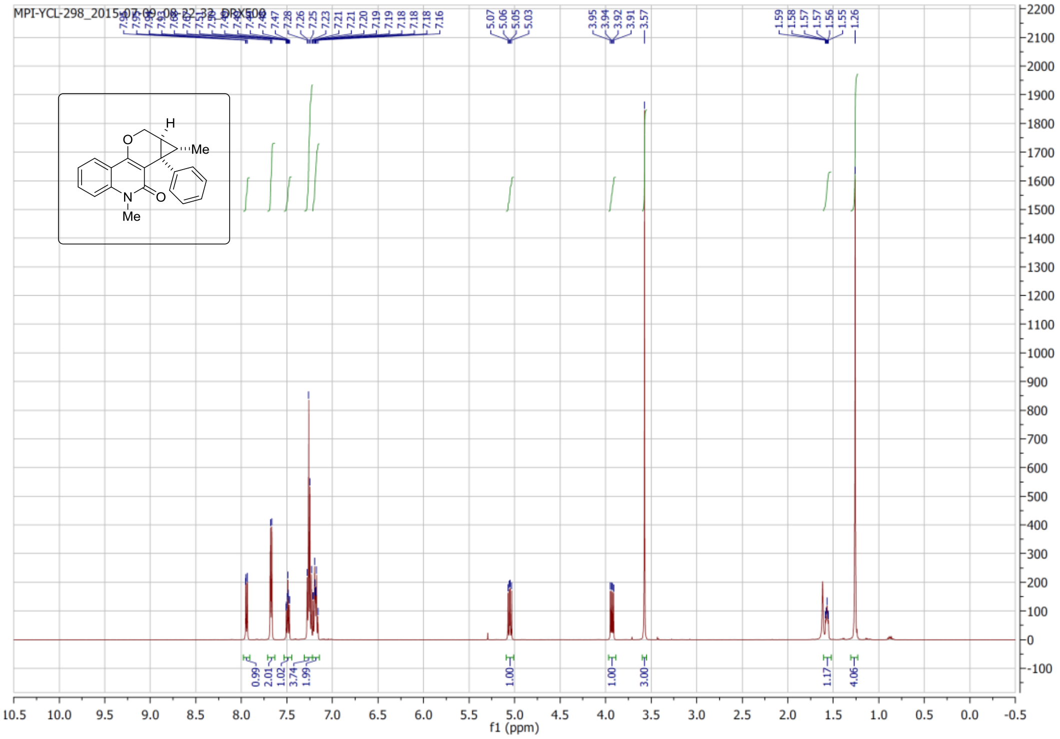

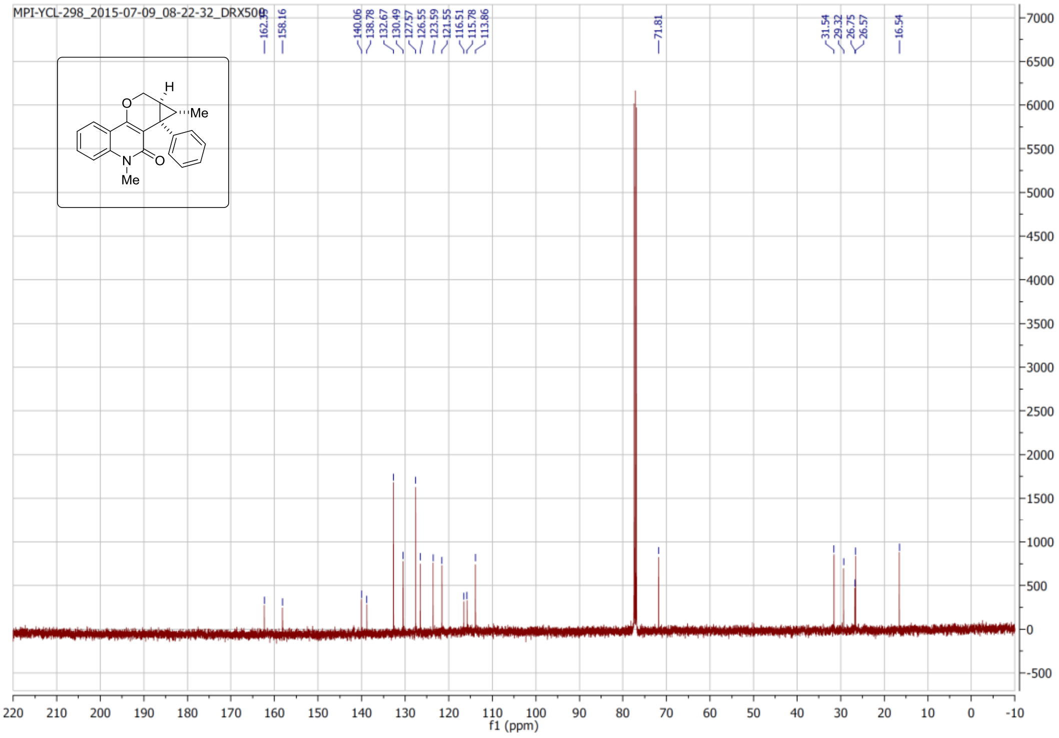

Supplementary Figure 85. ^{1}H and ^{13}C NMR spectra for **3a**.

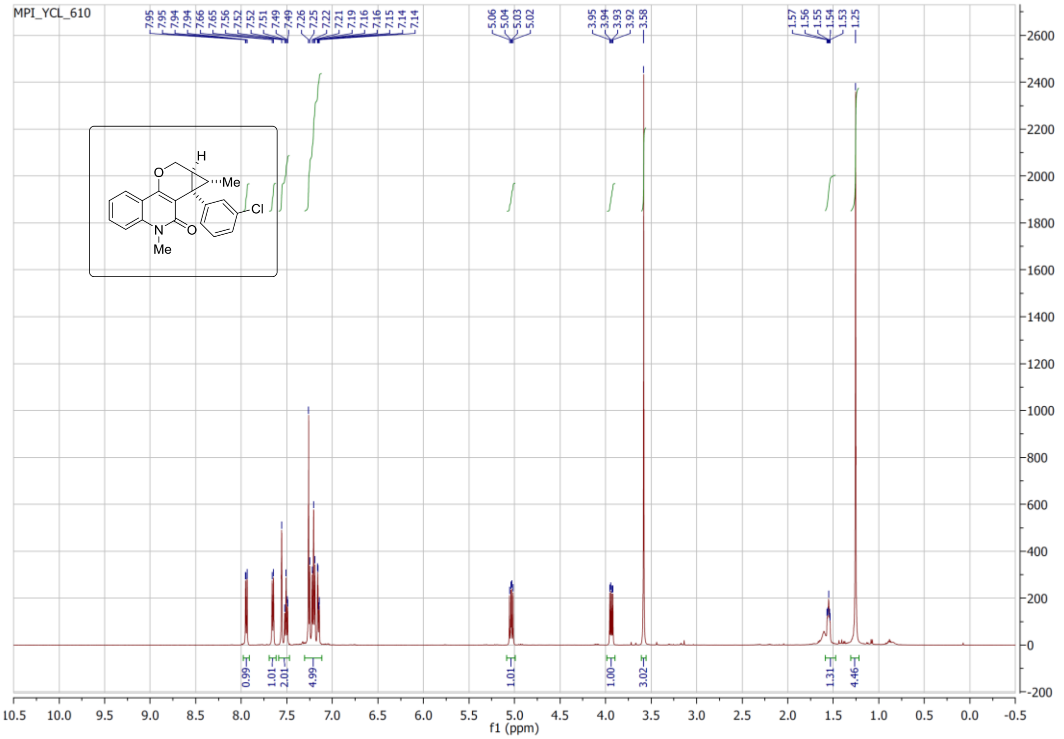

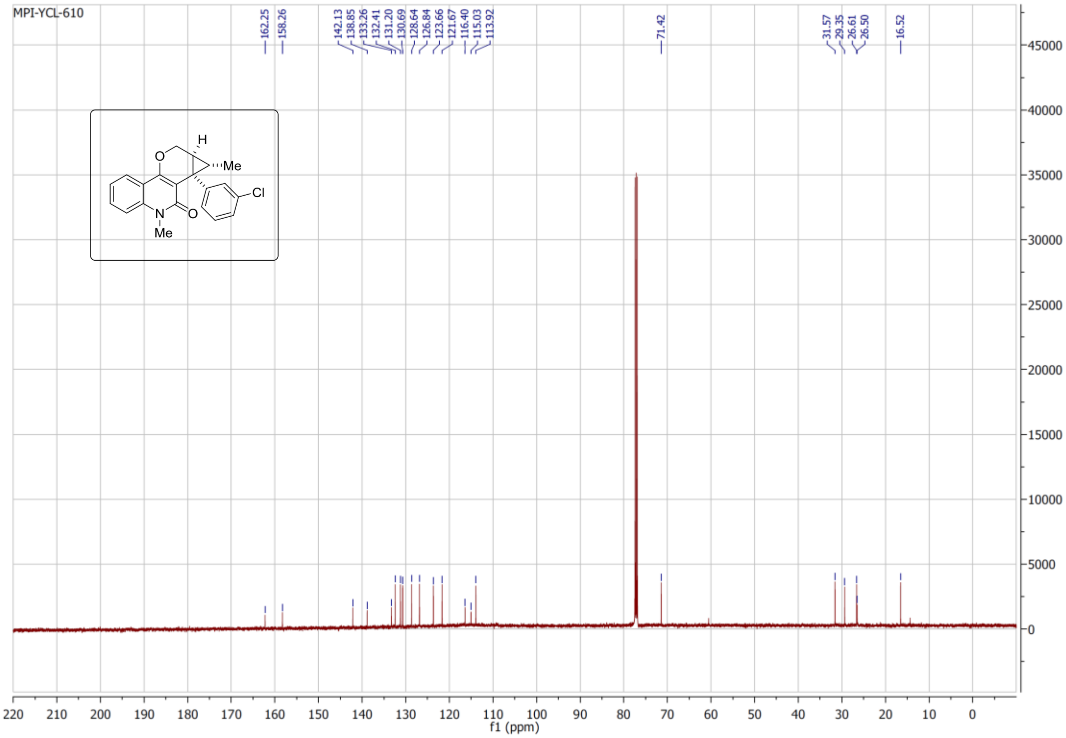

Supplementary Figure 86. ^{1}H and ^{13}C NMR spectra for **3b**.

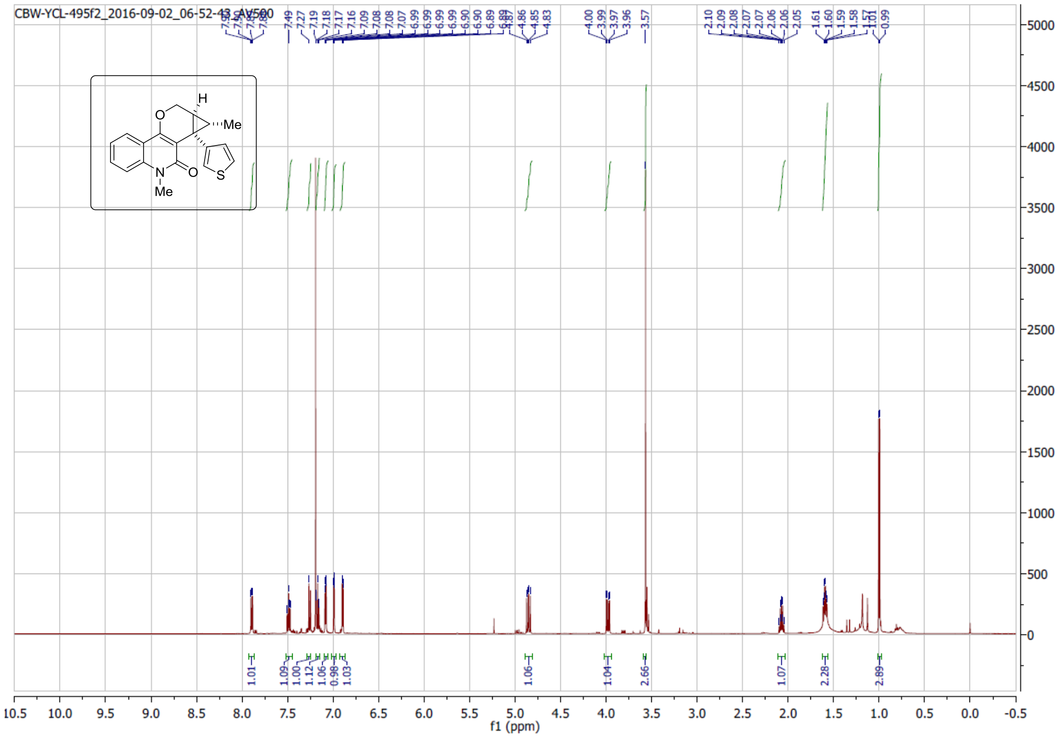

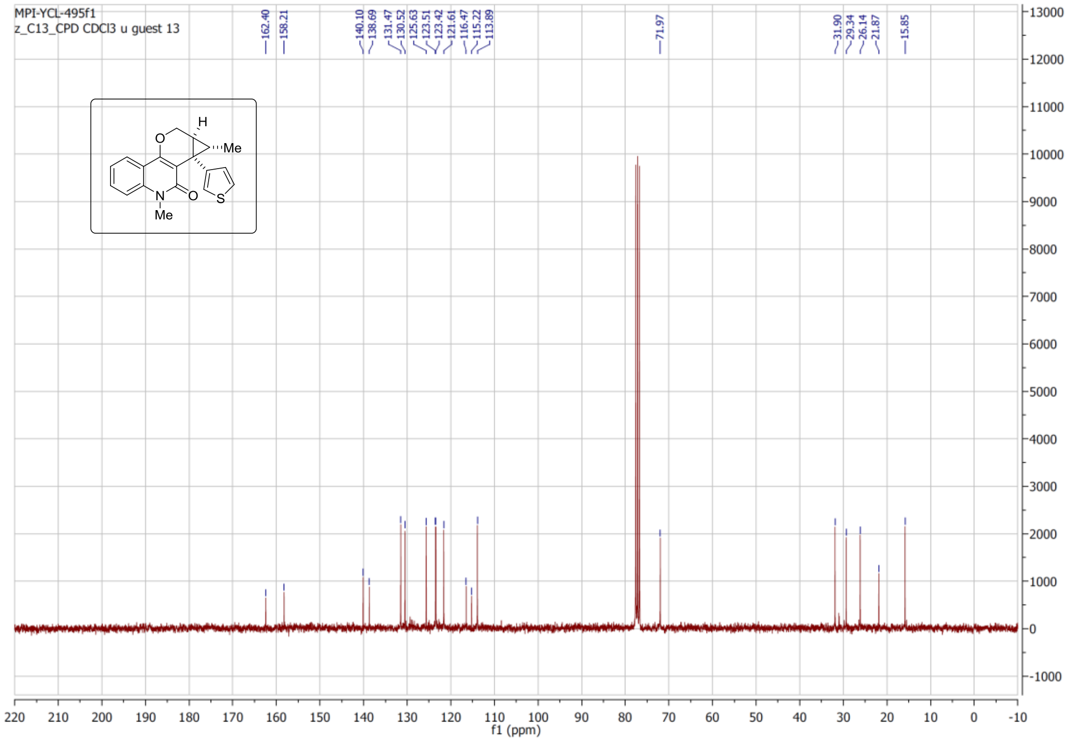

Supplementary Figure 87. ^{1}H and ^{13}C NMR spectra for **3c**.

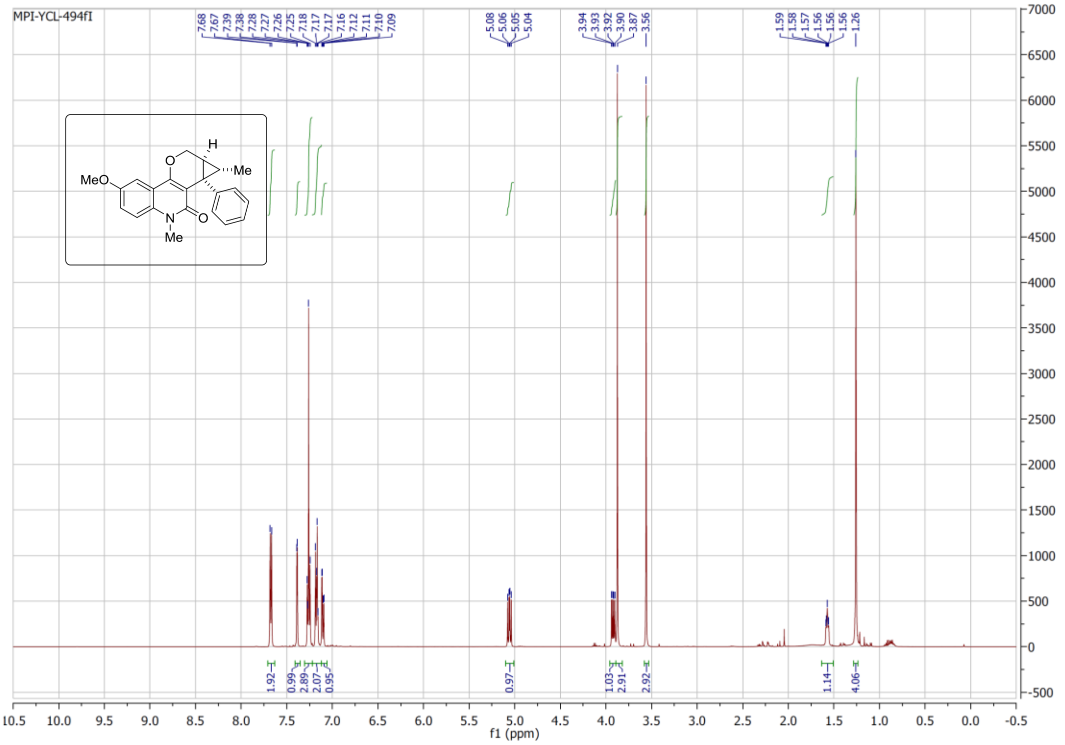

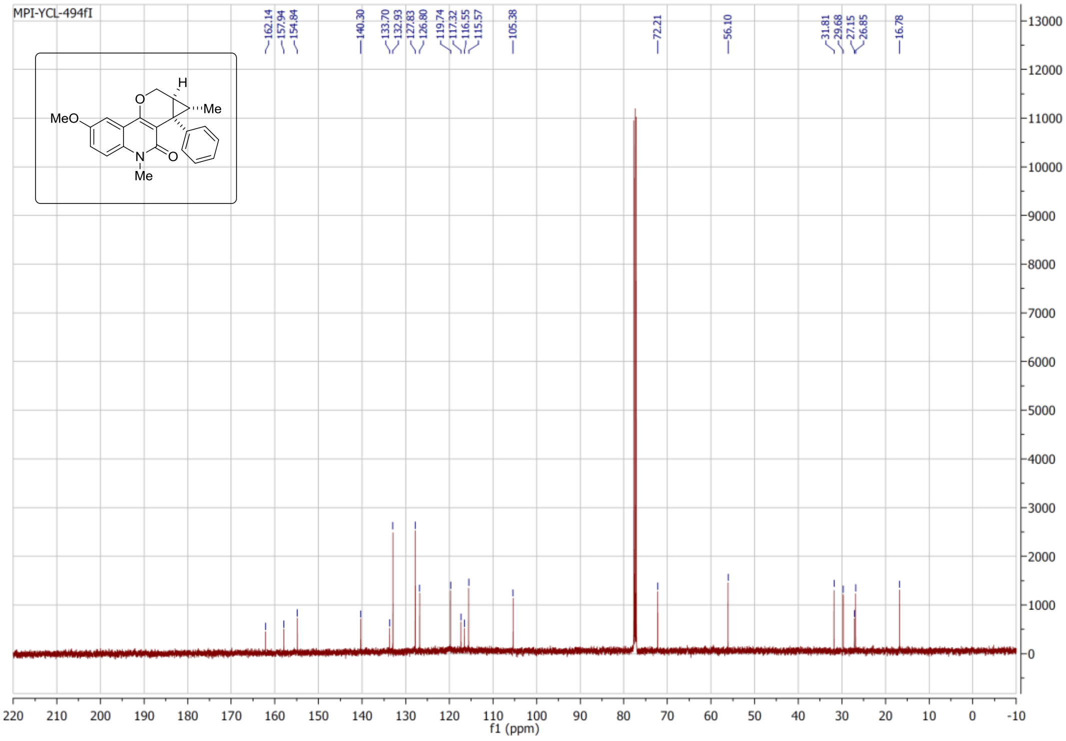

Supplementary Figure 88. ^{1}H and ^{13}C NMR spectra for **3d**.

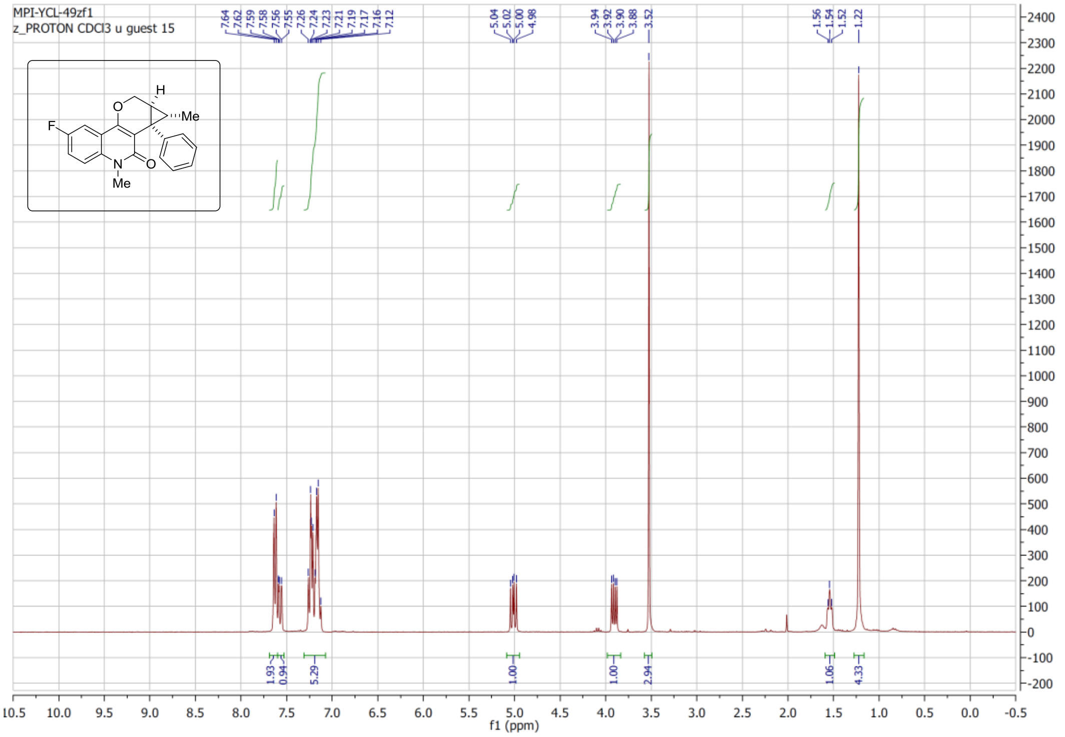

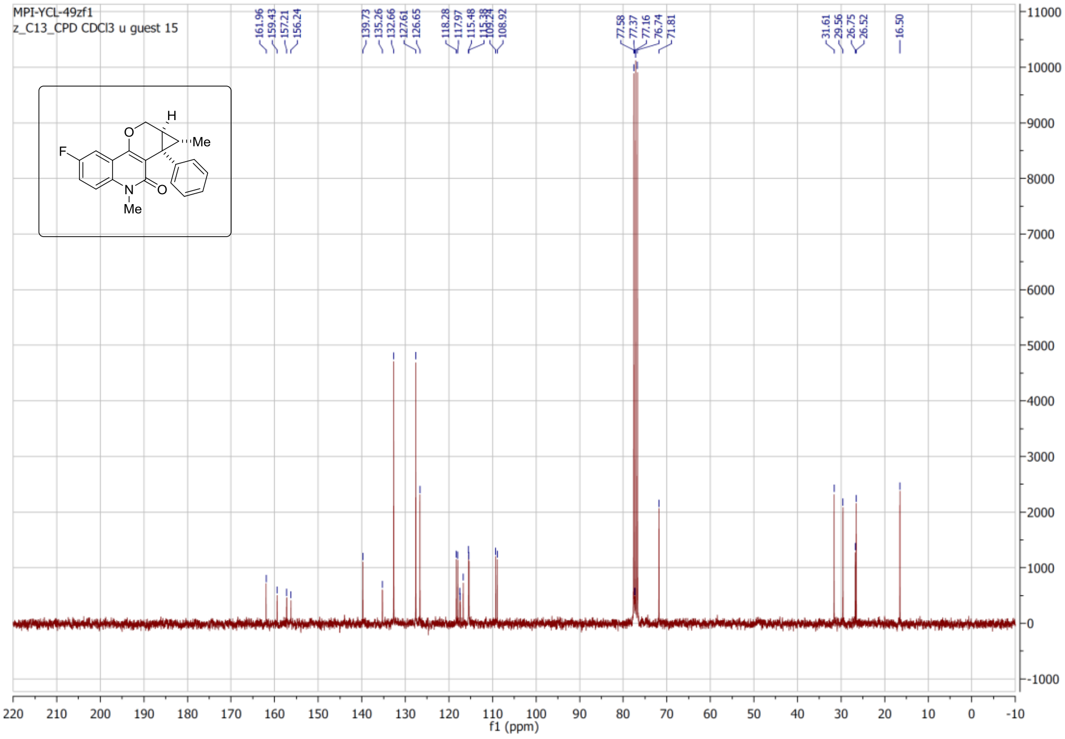

Supplementary Figure 89. ^{1}H and ^{13}C NMR spectra for **3e**.

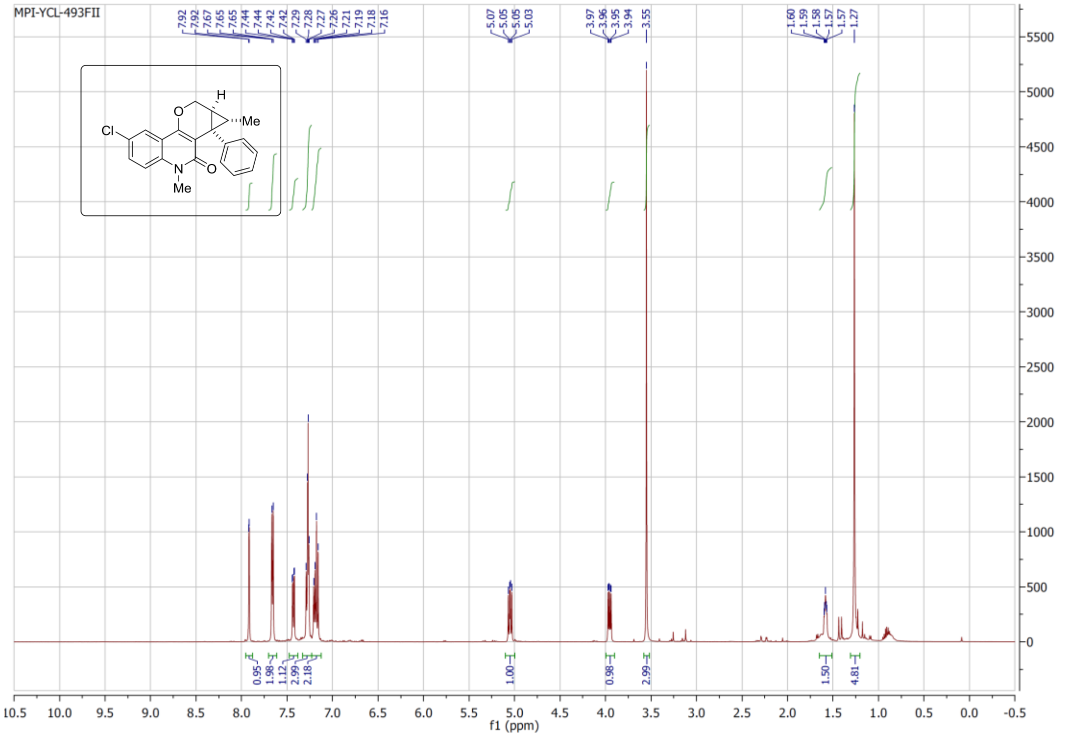

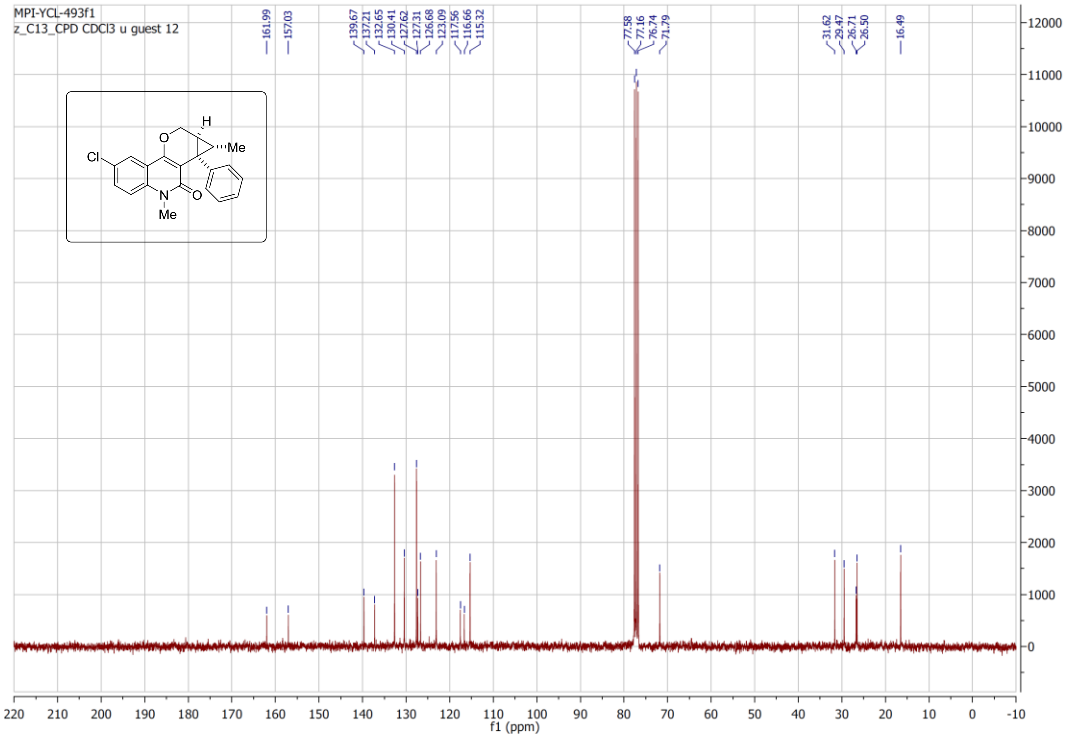

Supplementary Figure 90. ^{1}H and ^{13}C NMR spectra for **3f**.

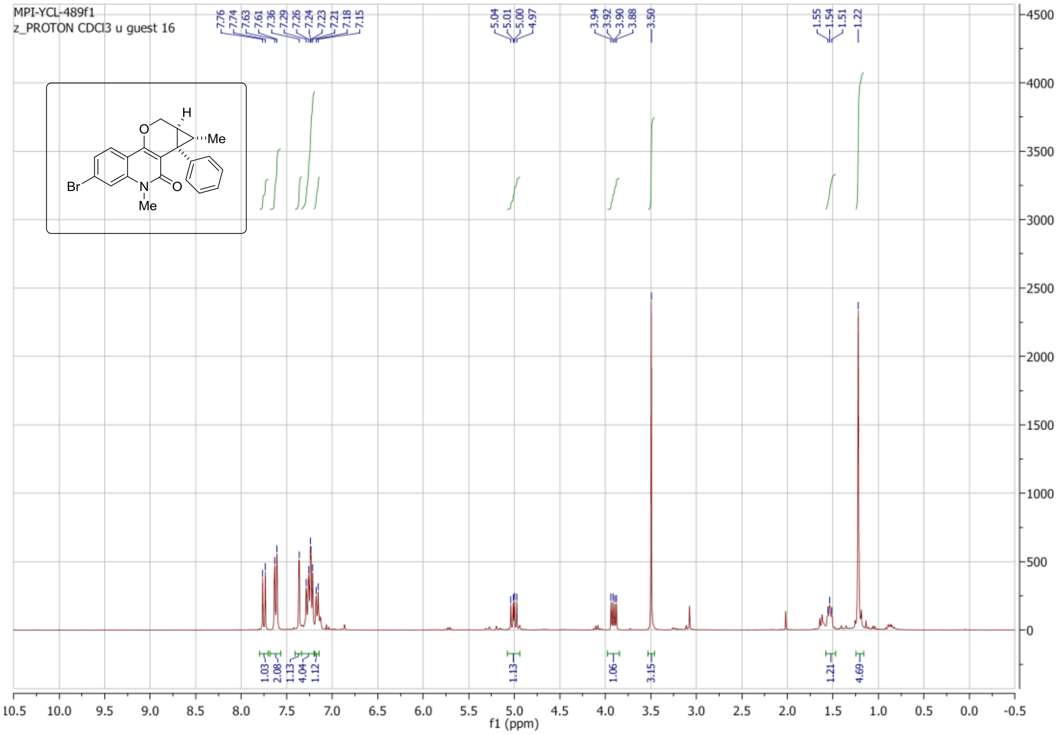

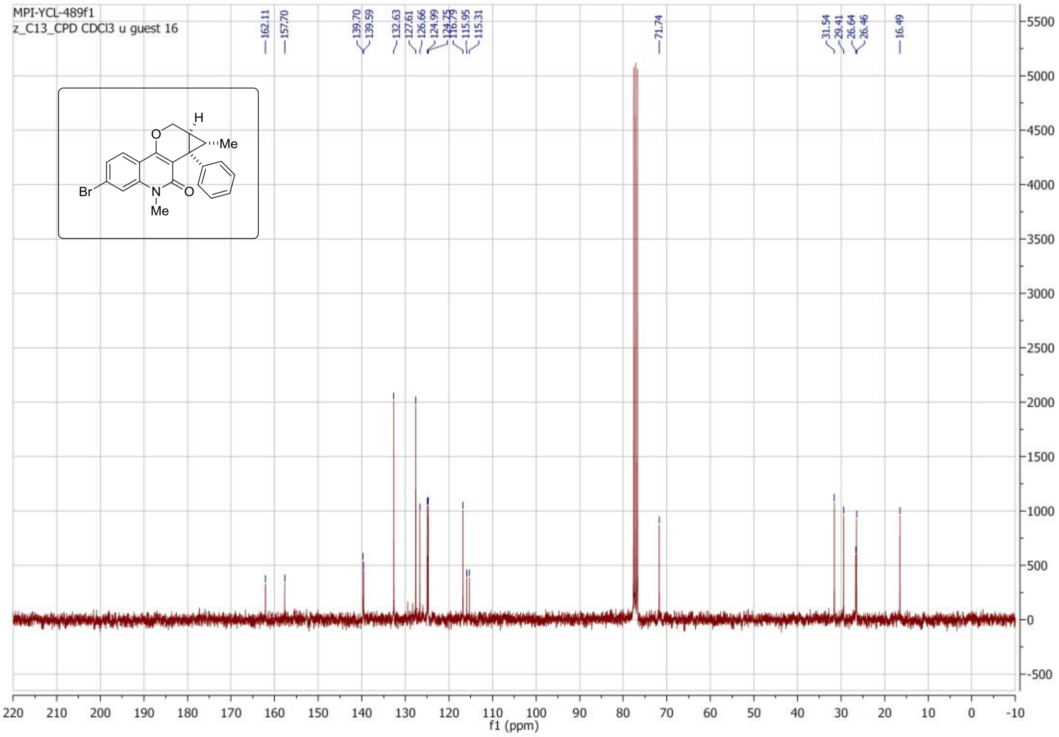

Supplementary Figure 91. ^{1}H and ^{13}C NMR spectra for **3g**.

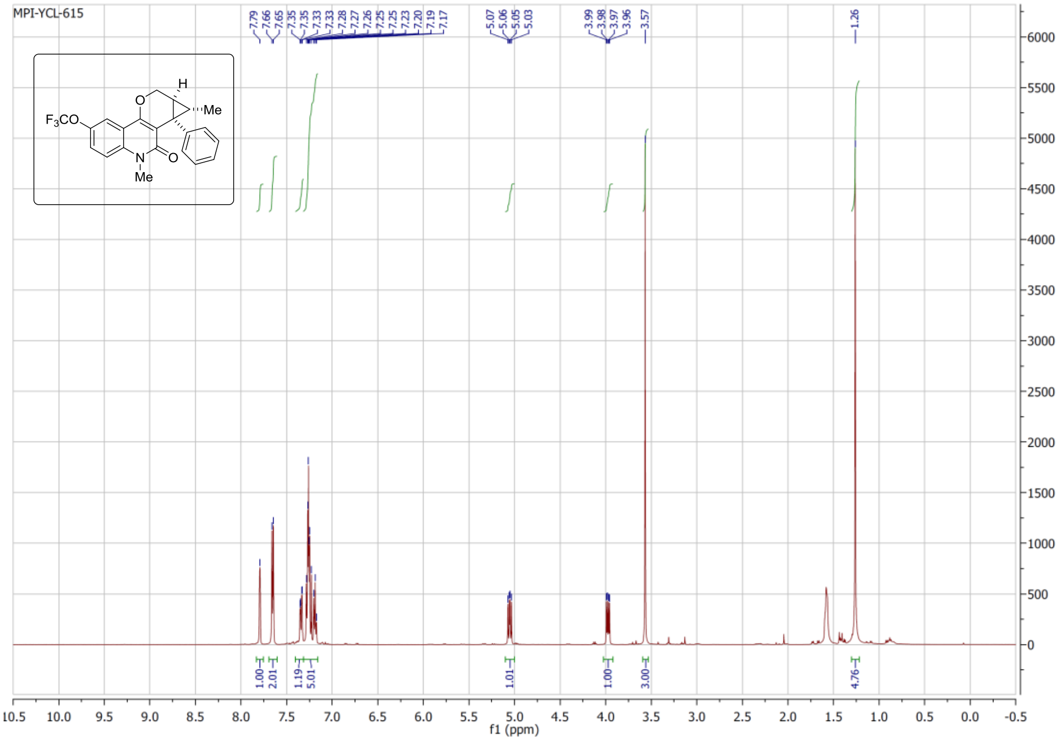

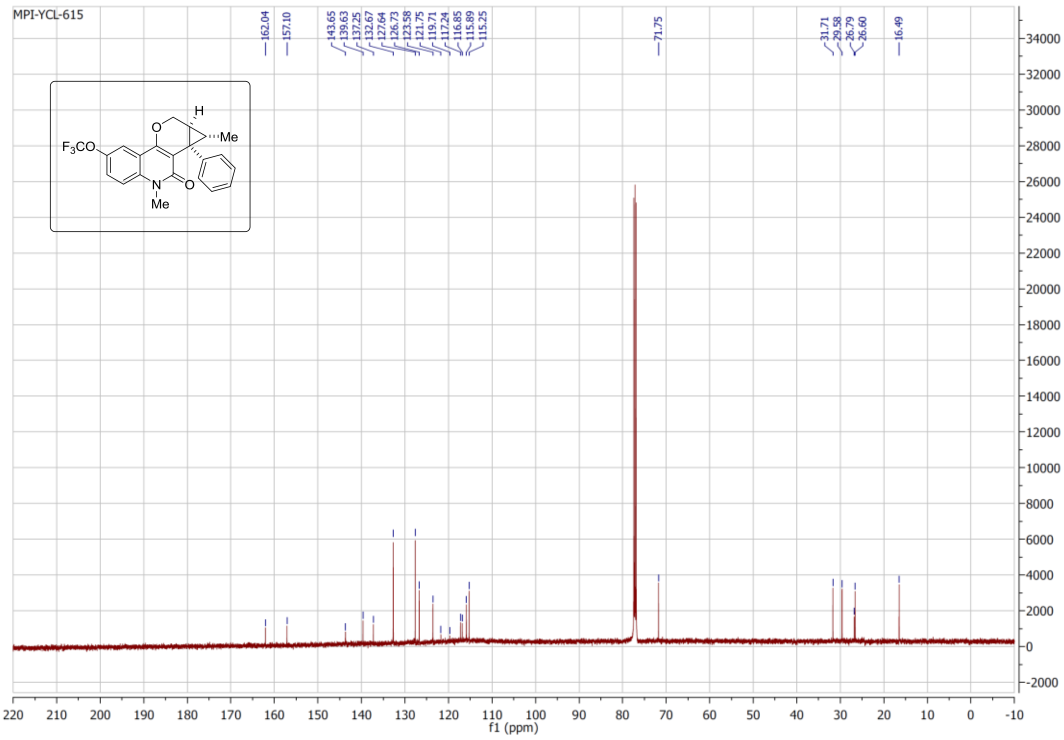

Supplementary Figure 92. ^{1}H and ^{13}C NMR spectra for **3h**.

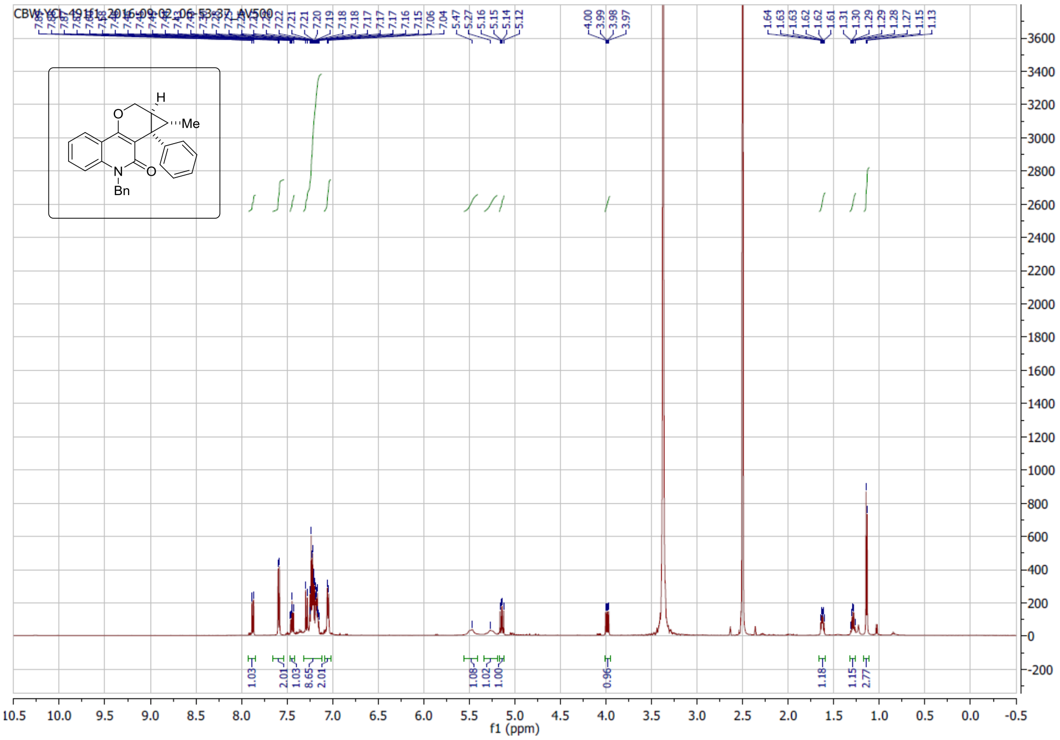

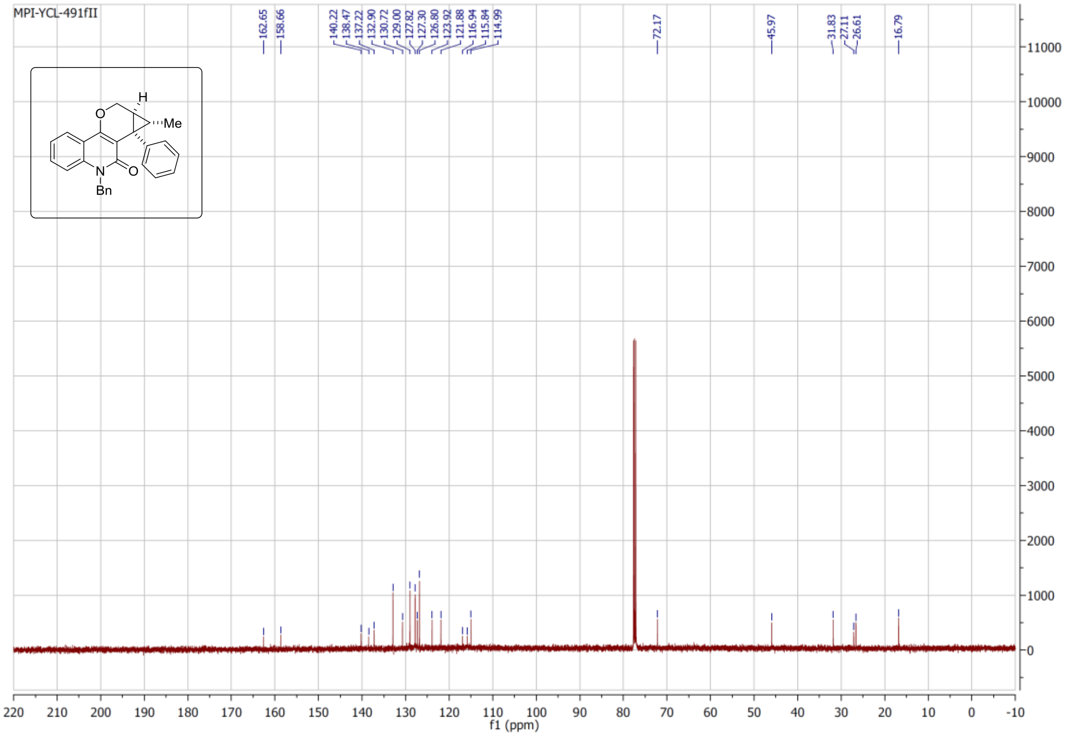

Supplementary Figure 93. ^{1}H and ^{13}C NMR spectra for **3i**.

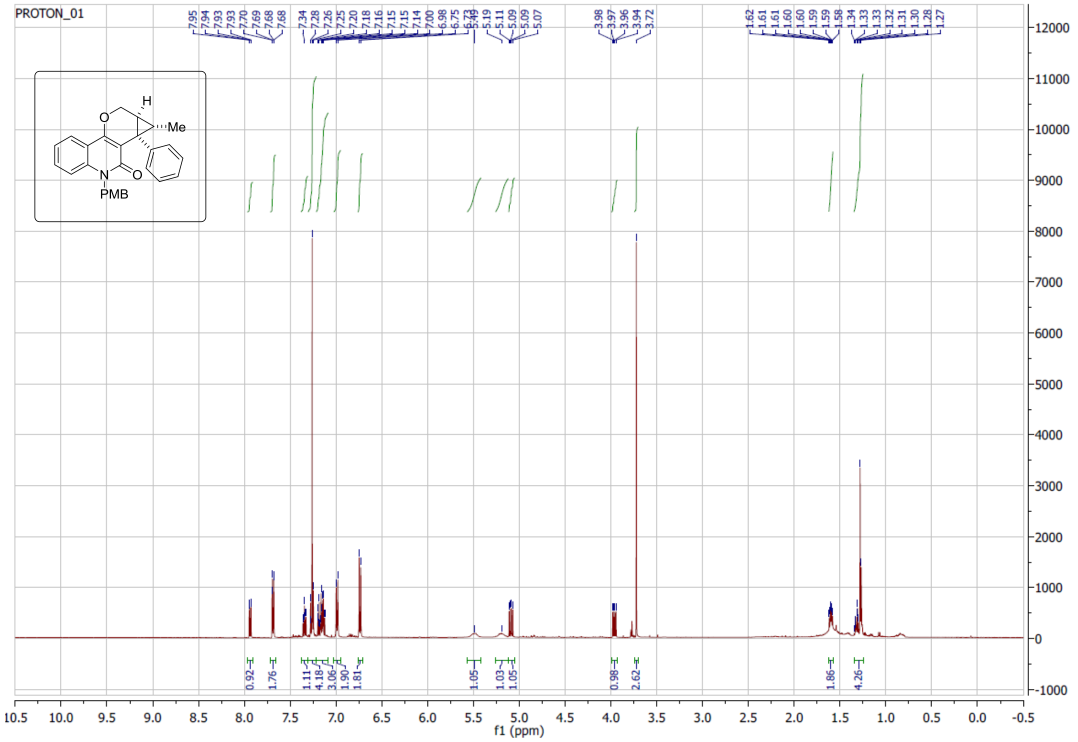

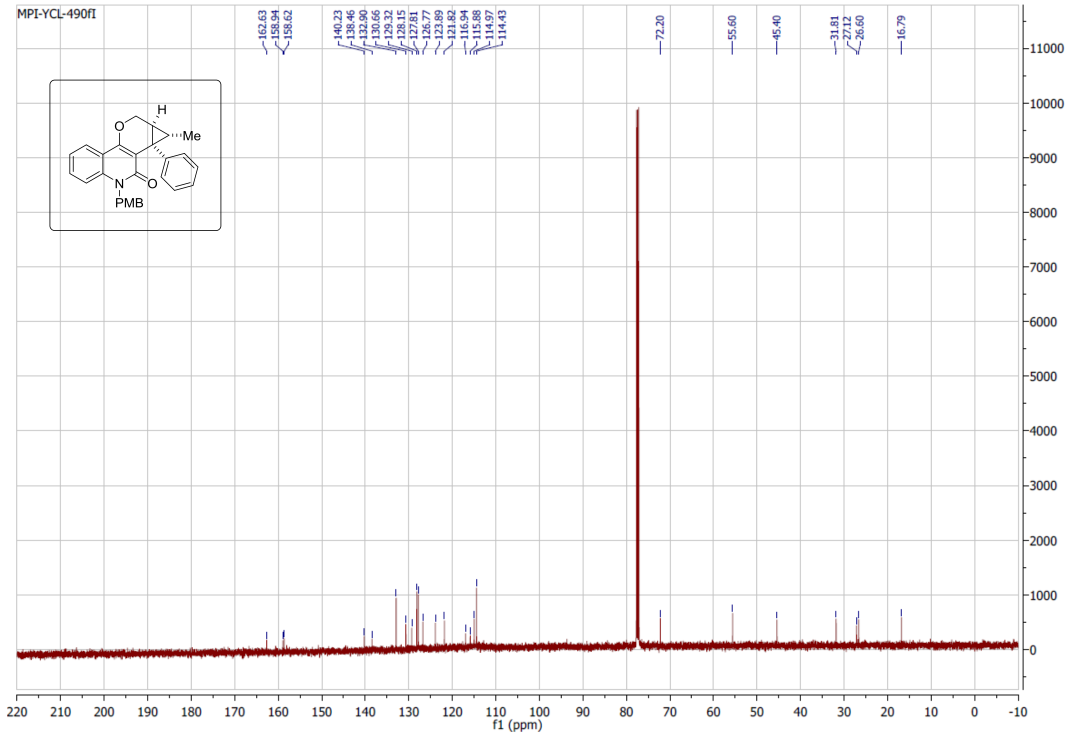

Supplementary Figure 94. ^{1}H and ^{13}C NMR spectra for **3j**.

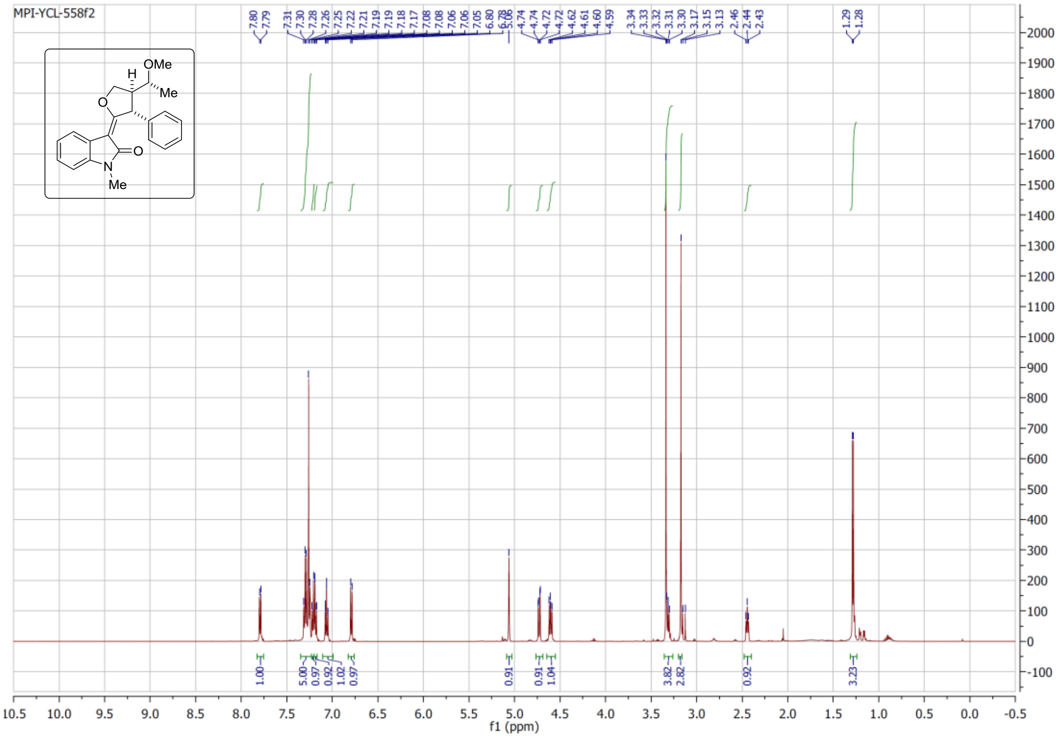

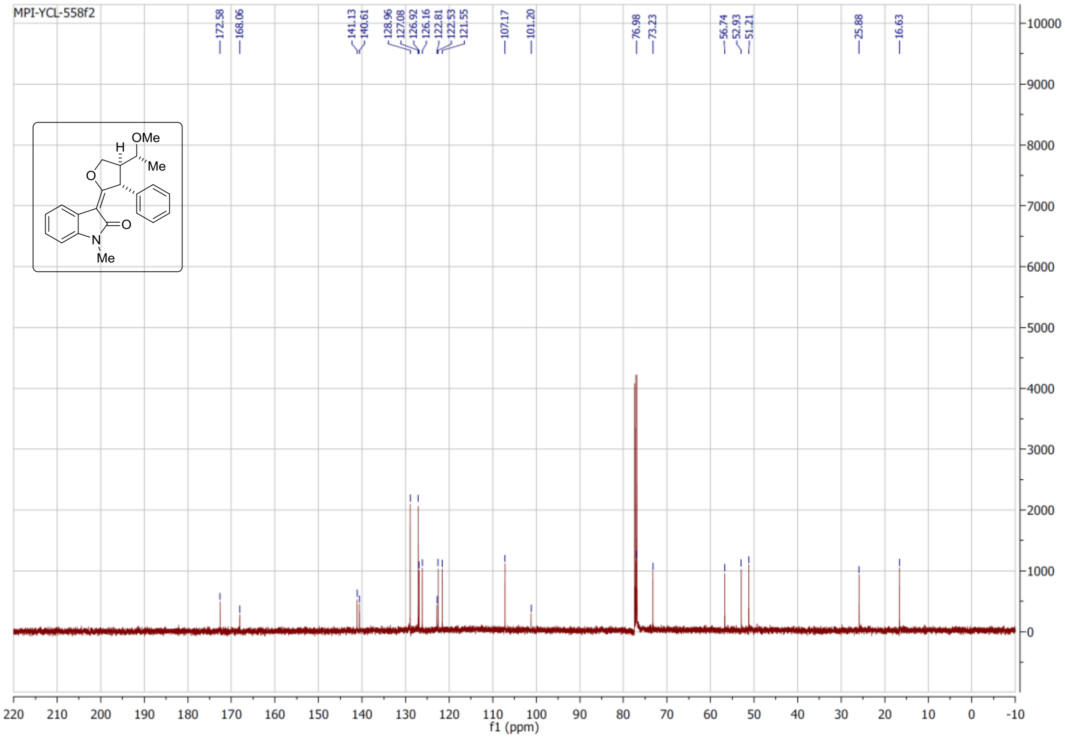

Supplementary Figure 95. ^{1}H and ^{13}C NMR spectra for **4a**.

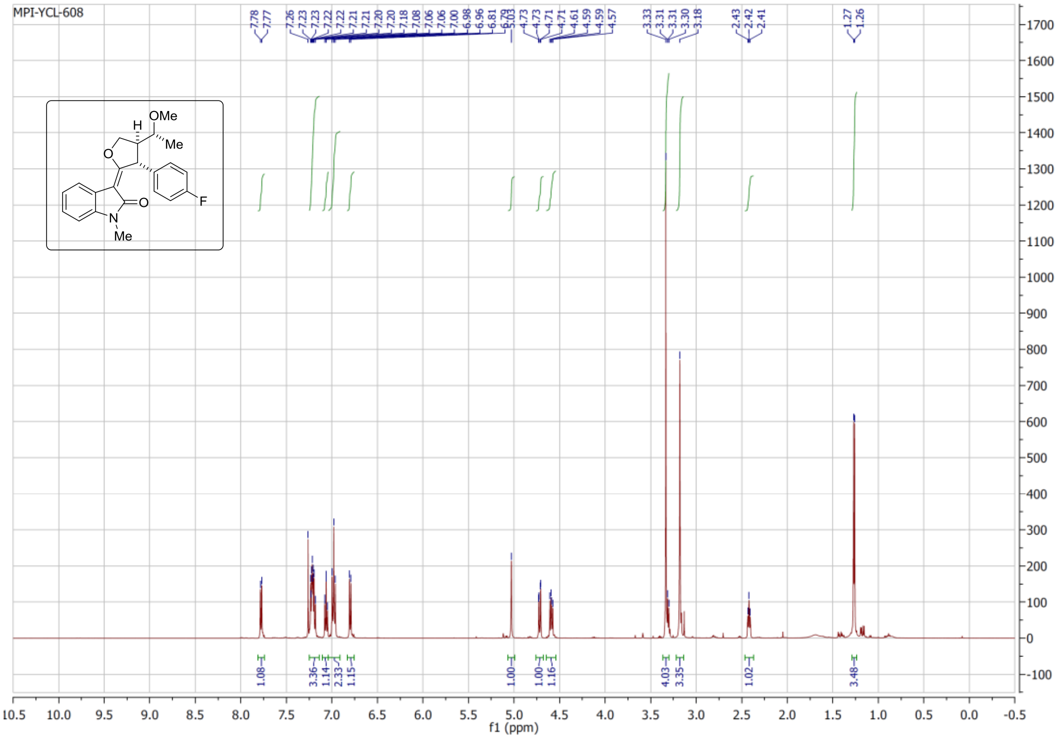

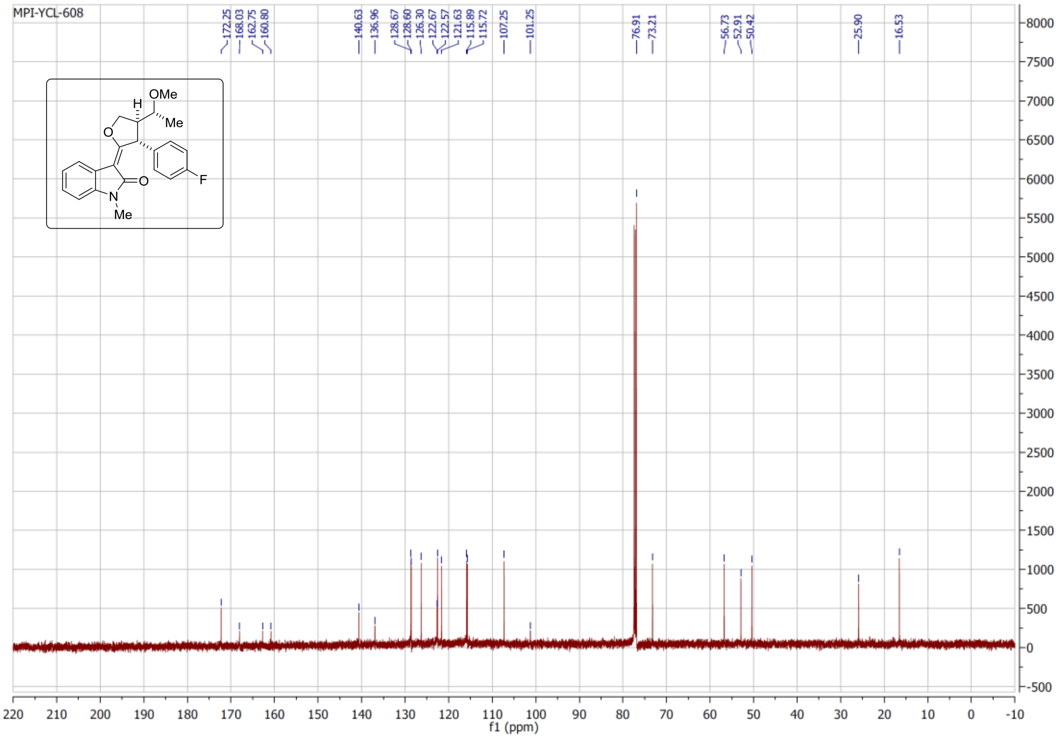

Supplementary Figure 96. ^{1}H and ^{13}C NMR spectra for **4b**.

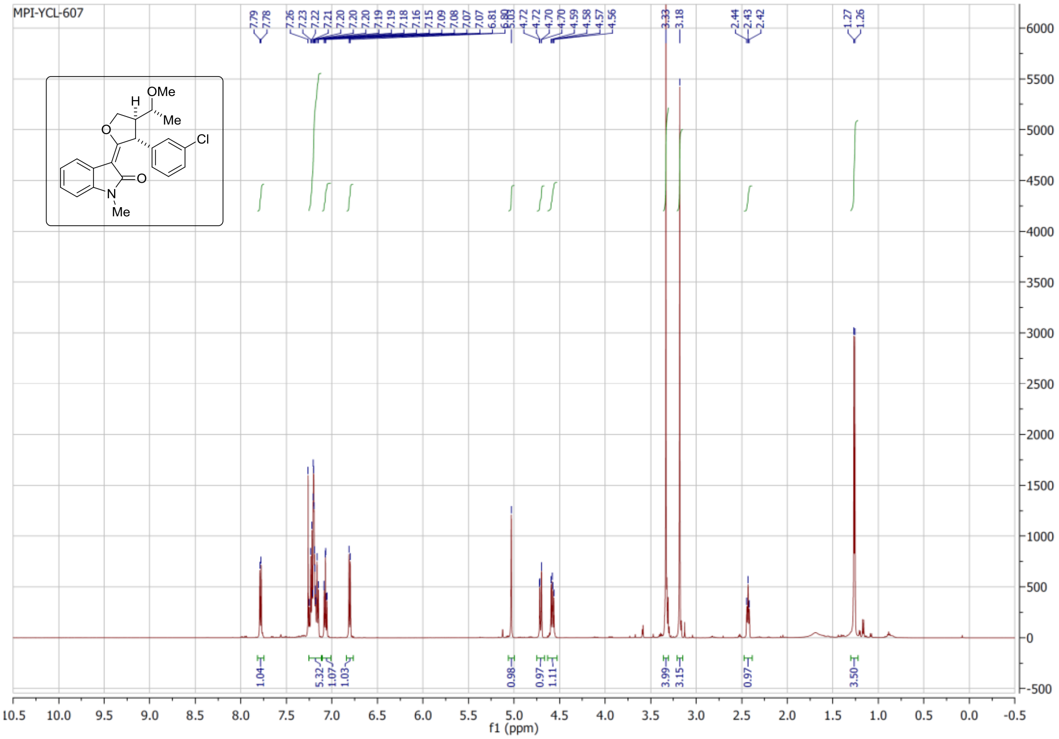

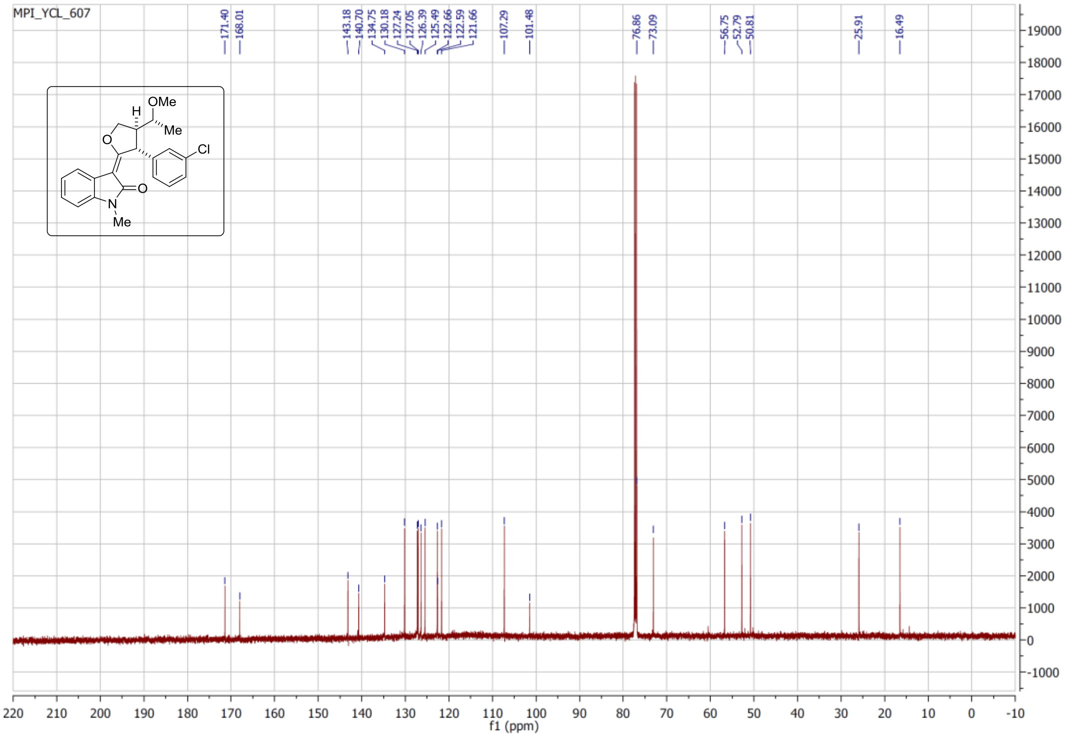

Supplementary Figure 97. ^{1}H and ^{13}C NMR spectra for **4c**.

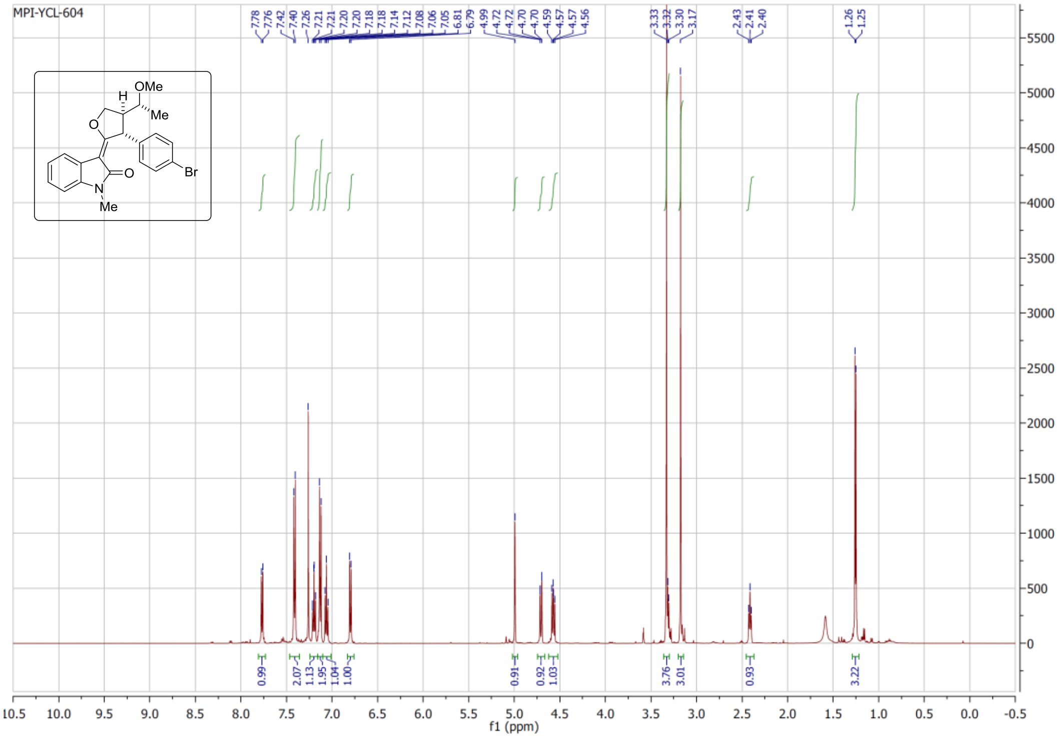

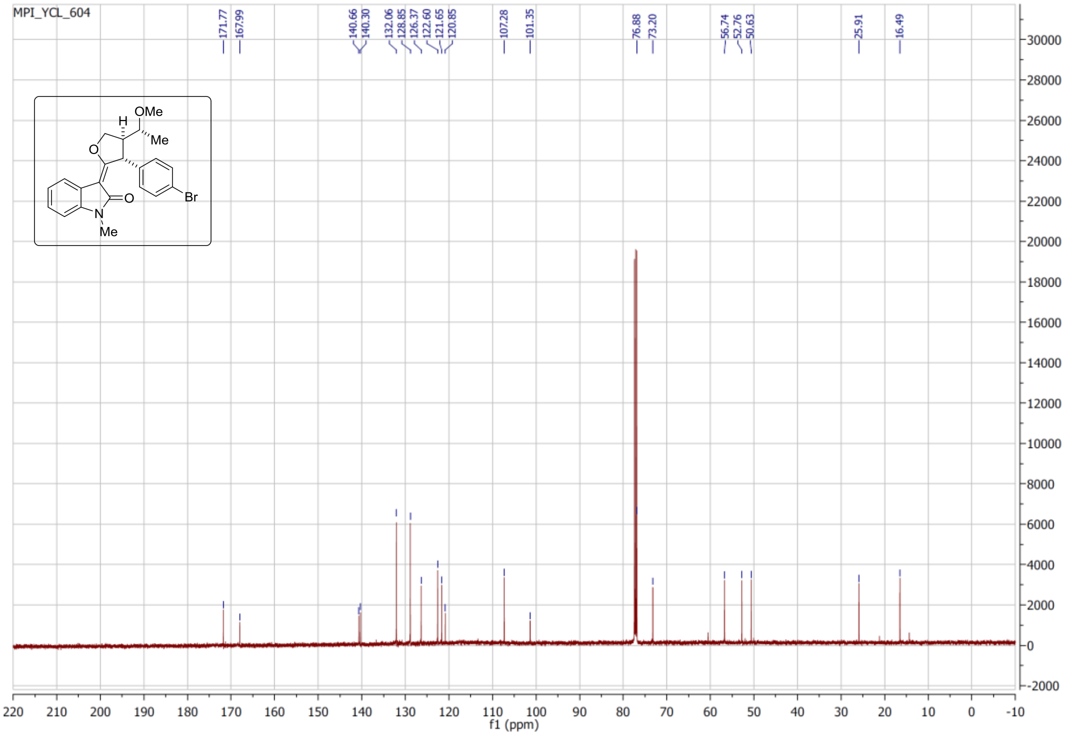

Supplementary Figure 98. ^{1}H and ^{13}C NMR spectra for **4d**.

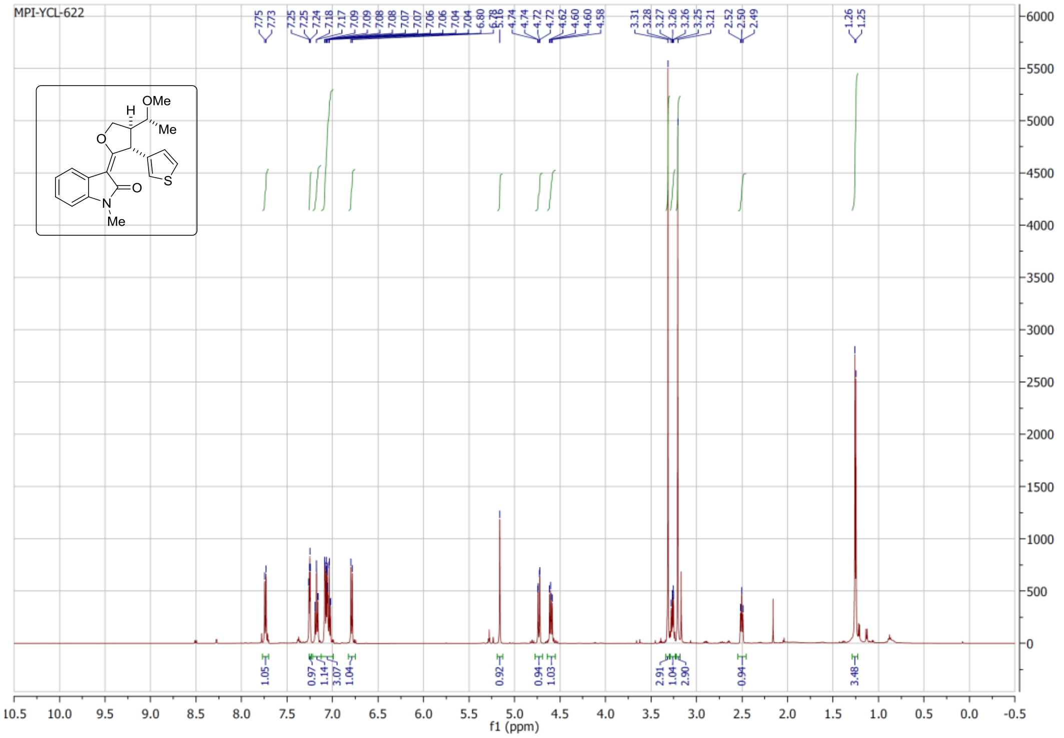

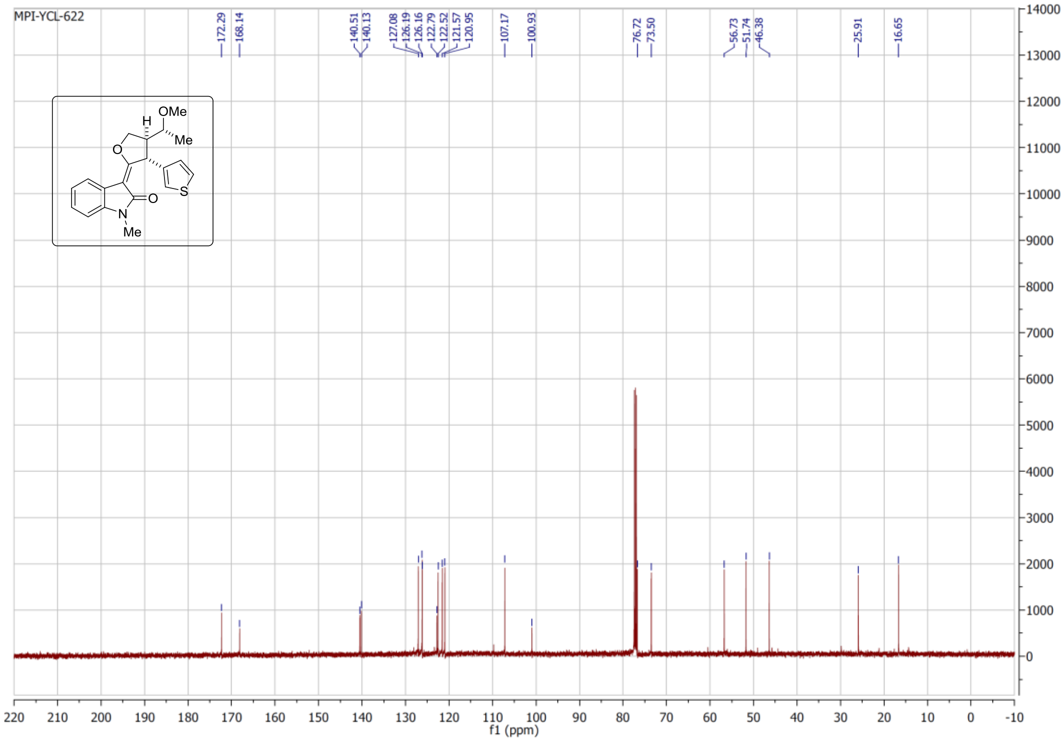

Supplementary Figure 99. ^{1}H and ^{13}C NMR spectra for **4e**.

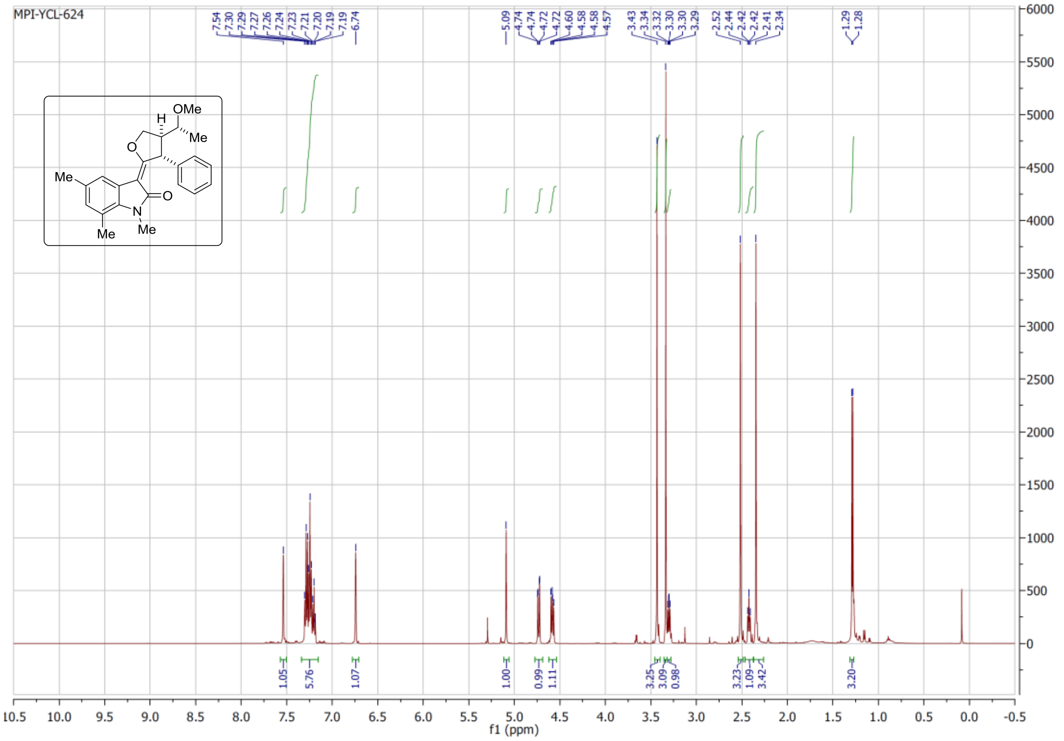

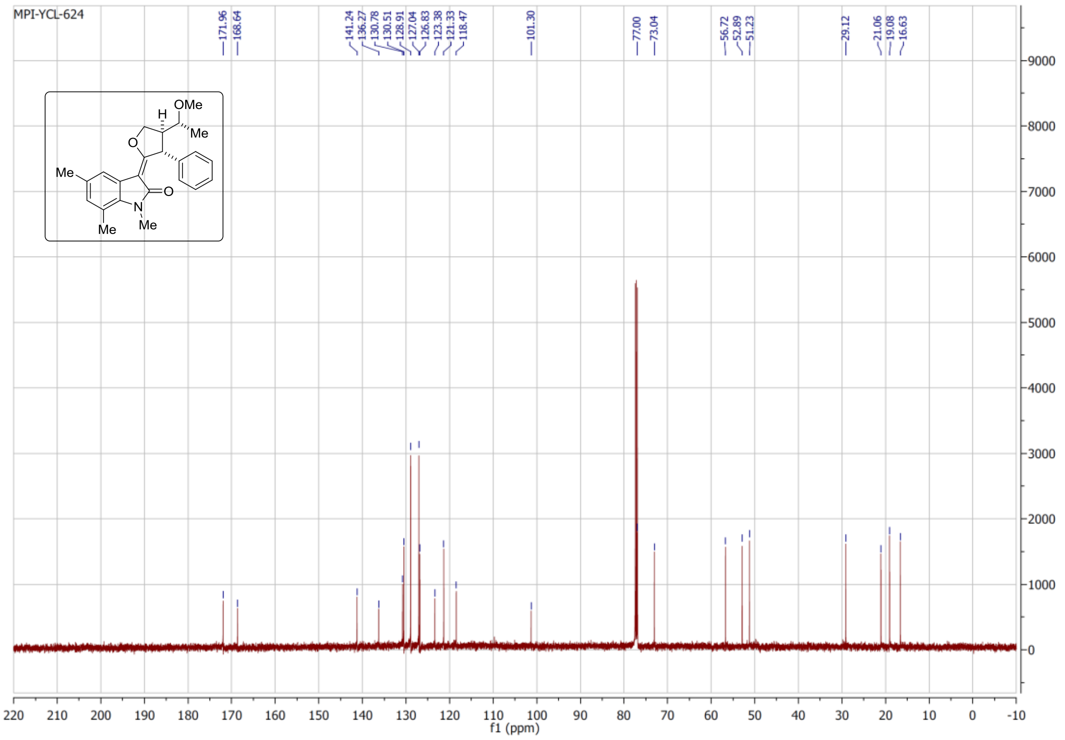

Supplementary Figure 100. ^{1}H and ^{13}C NMR spectra for **4f**.

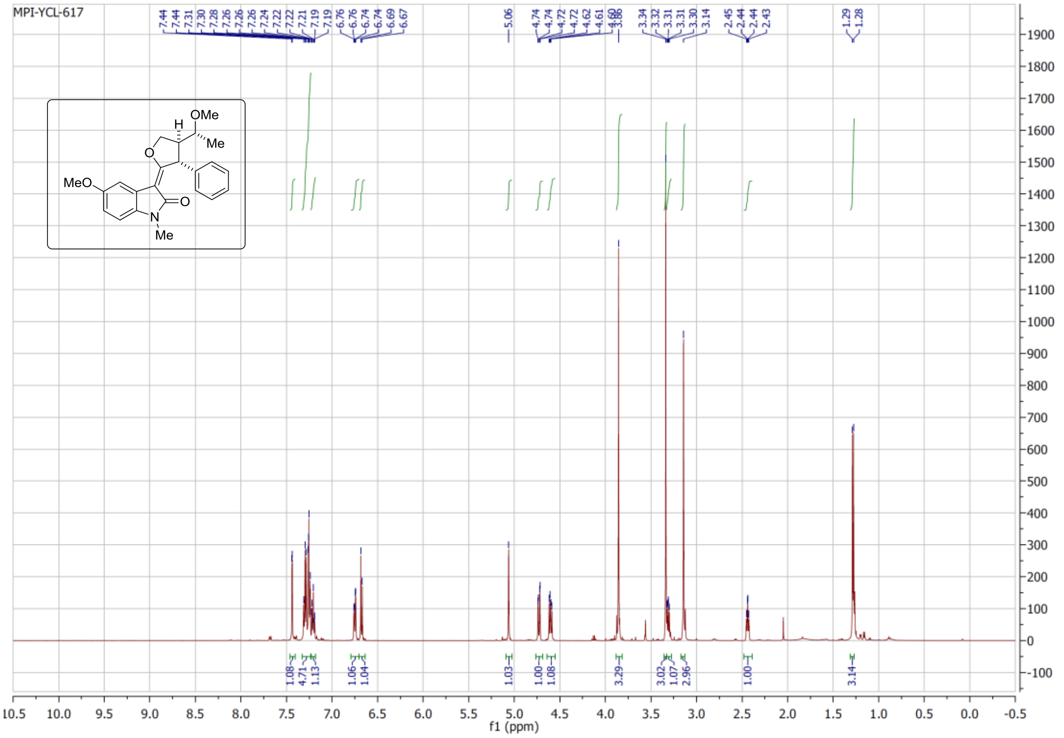

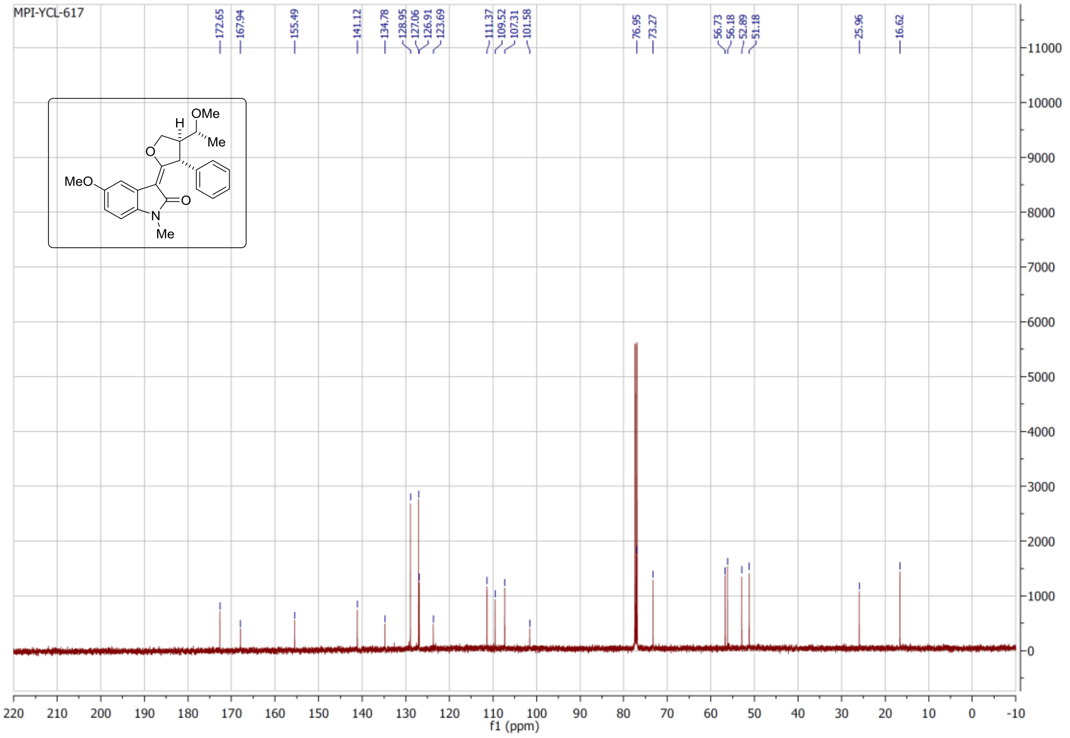

Supplementary Figure 101. ^{1}H and ^{13}C NMR spectra for **4g**.

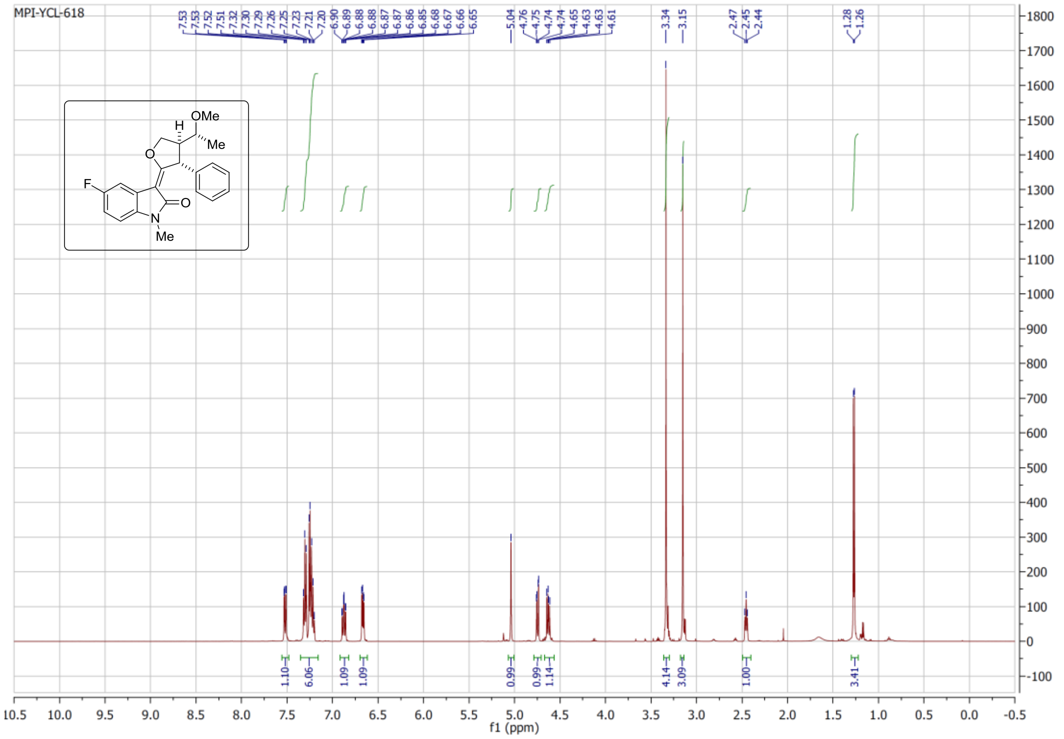

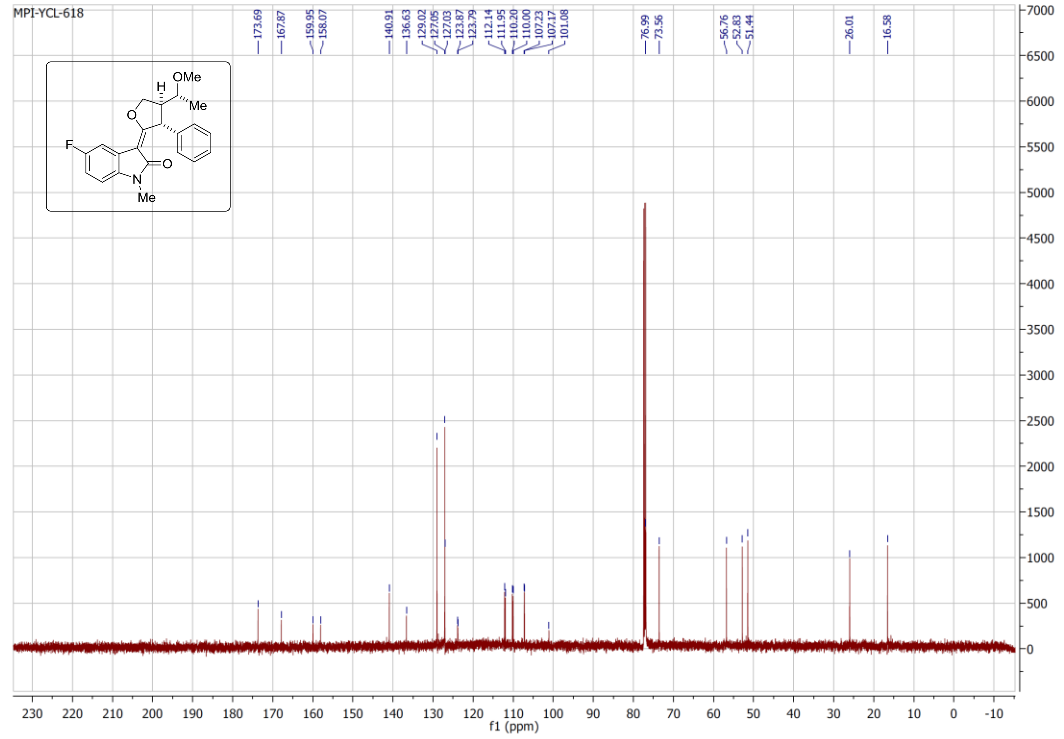

Supplementary Figure 102. ^{1}H and ^{13}C NMR spectra for **4h**.

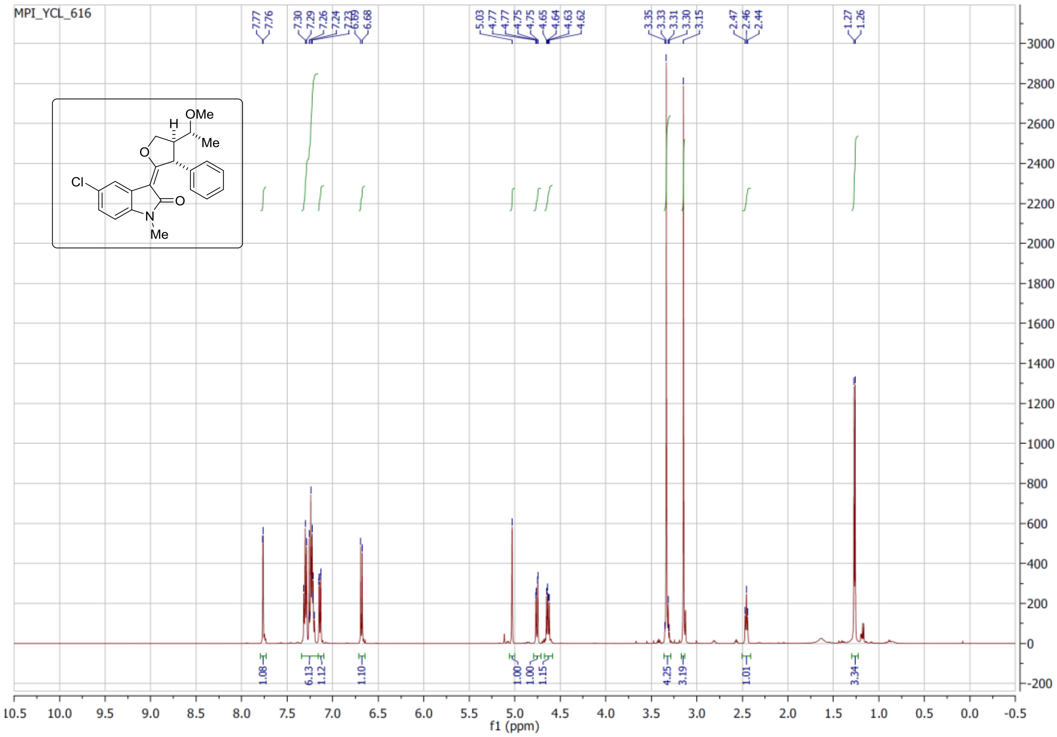

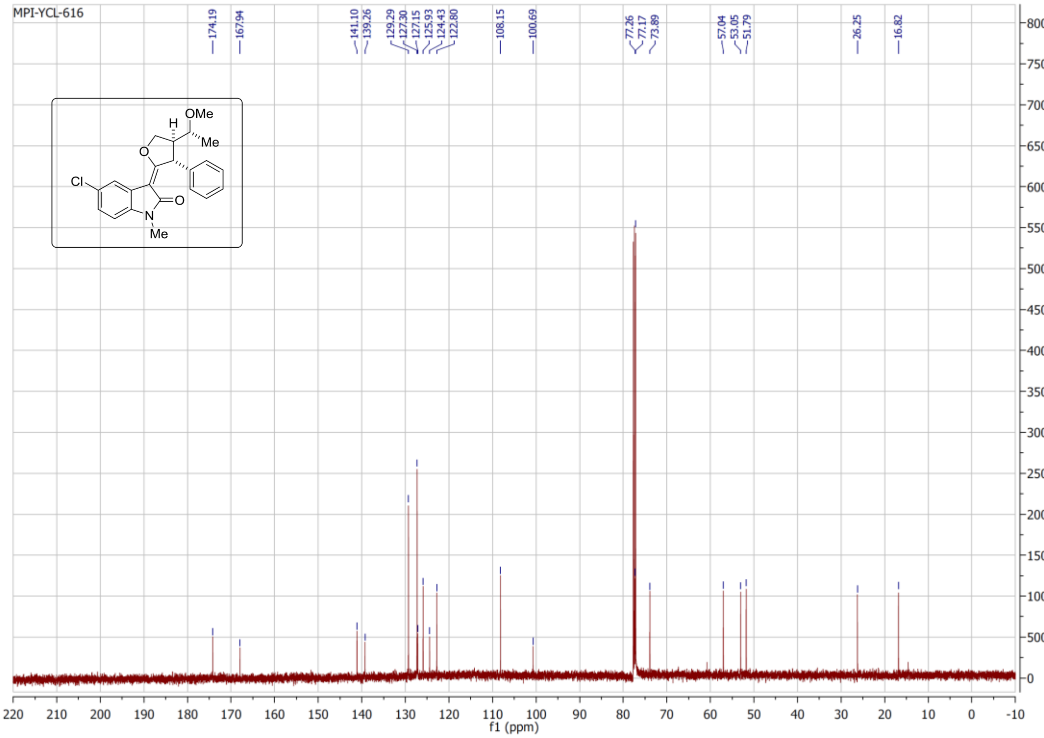

Supplementary Figure 103. ^{1}H and ^{13}C NMR spectra for **4i**.

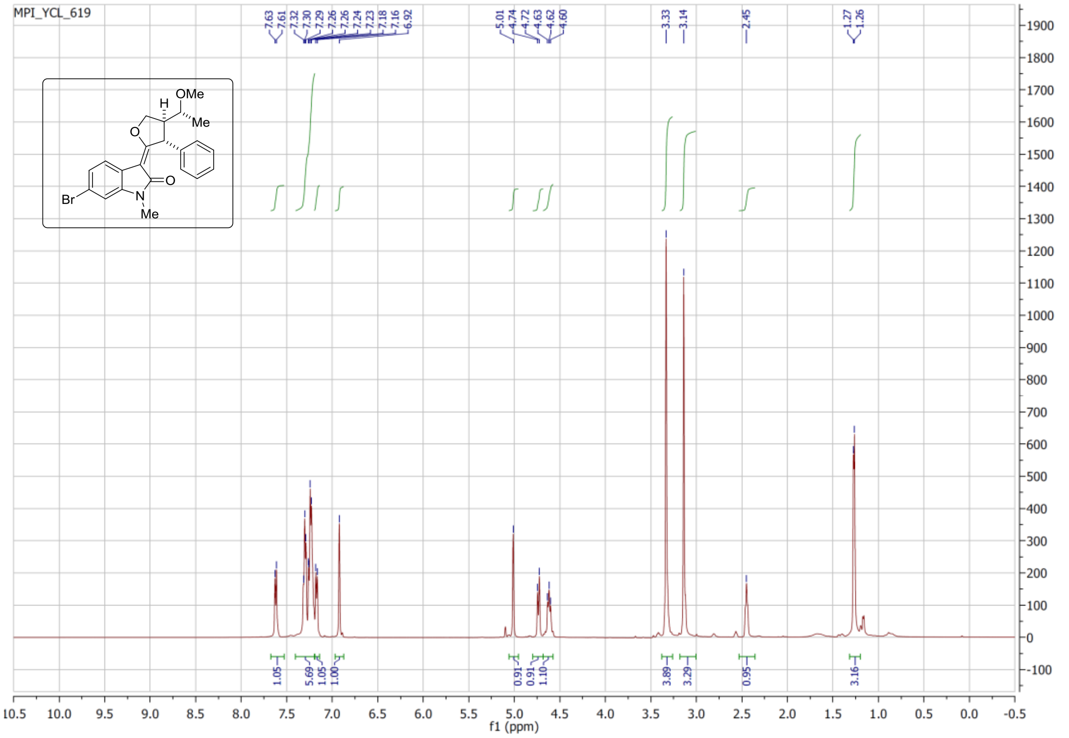

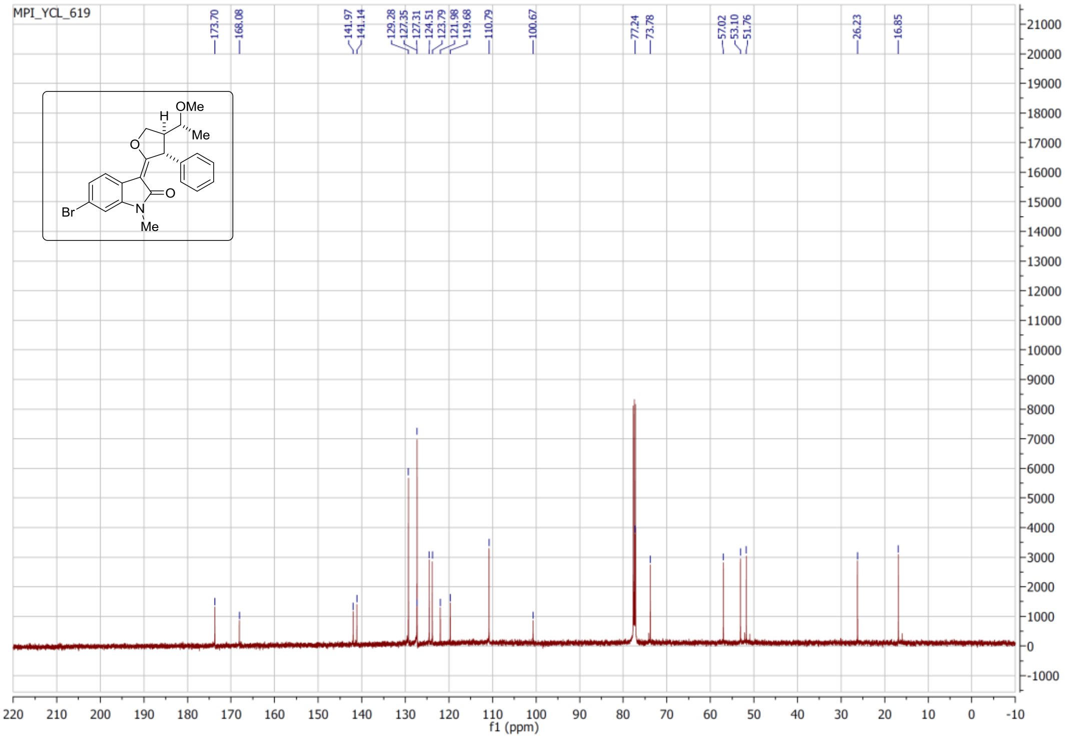

Supplementary Figure 104. ^{1}H and ^{13}C NMR spectra for **4j**.

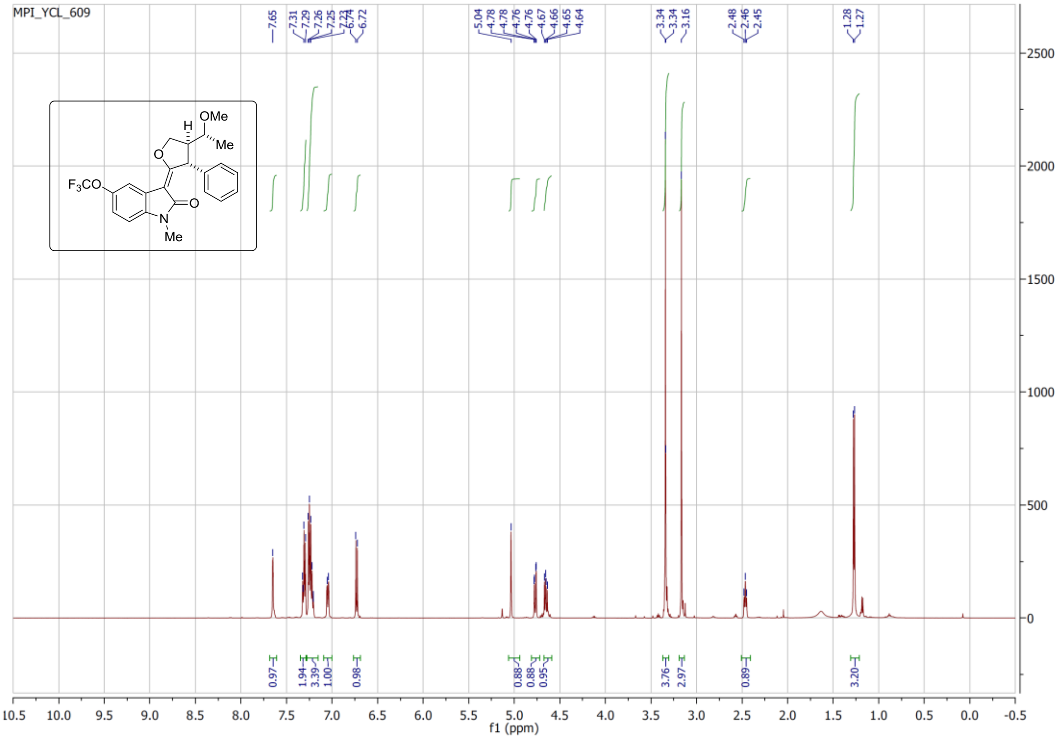

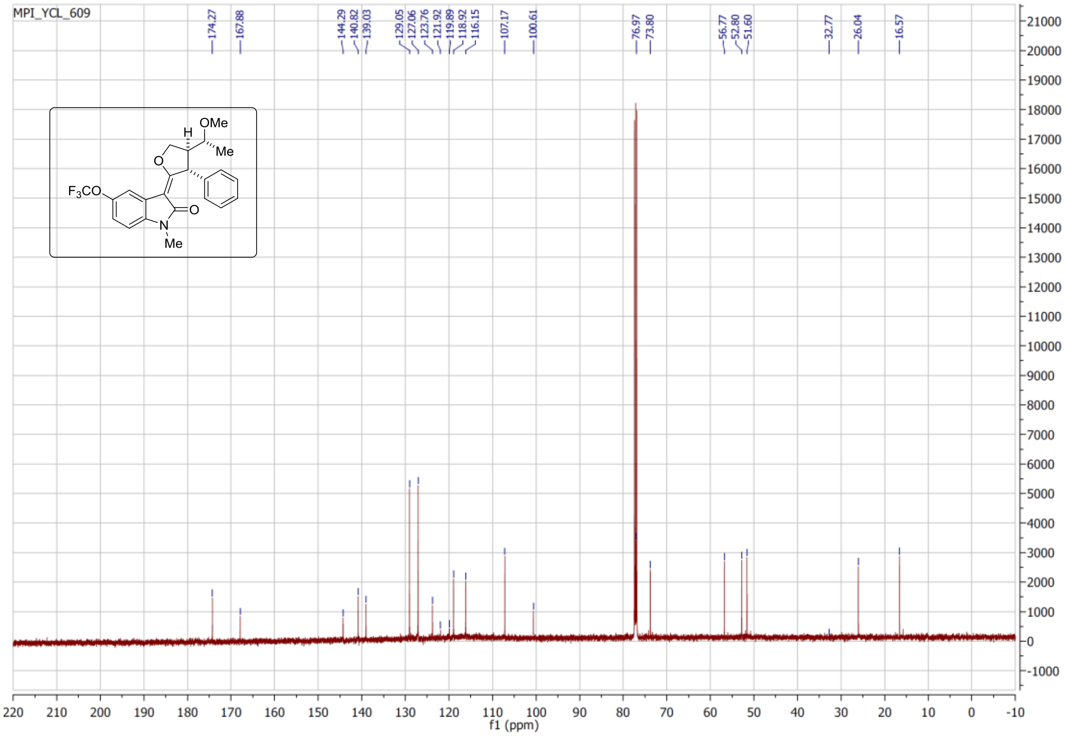

Supplementary Figure 105. ^{1}H and ^{13}C NMR spectra for **4k**.

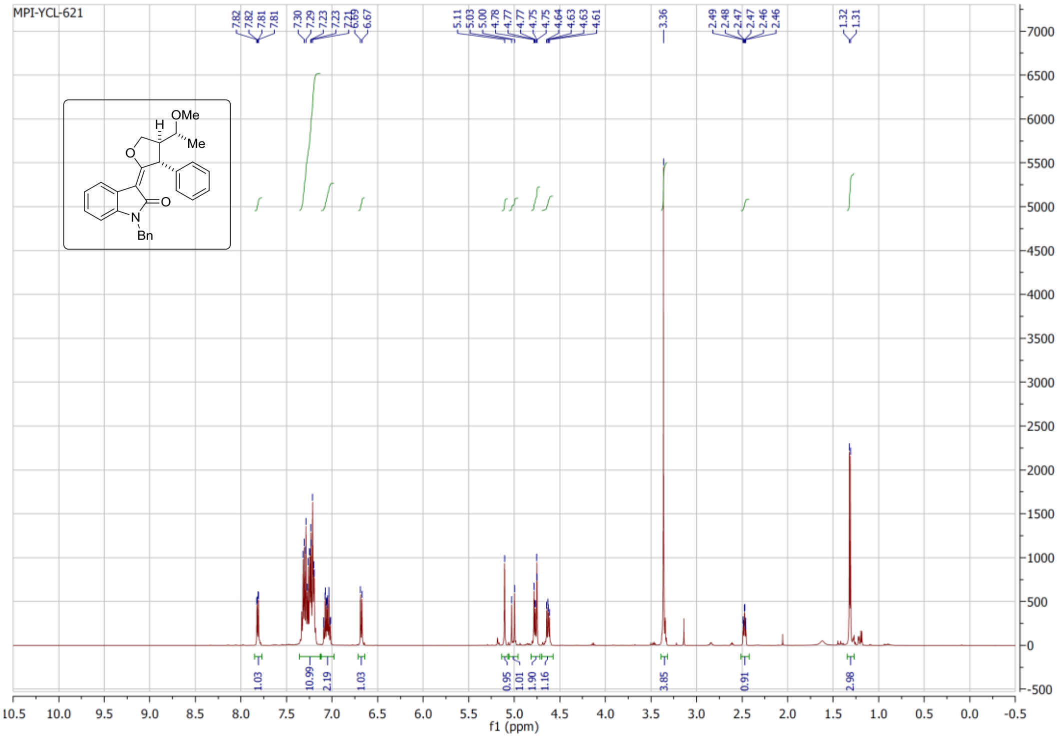

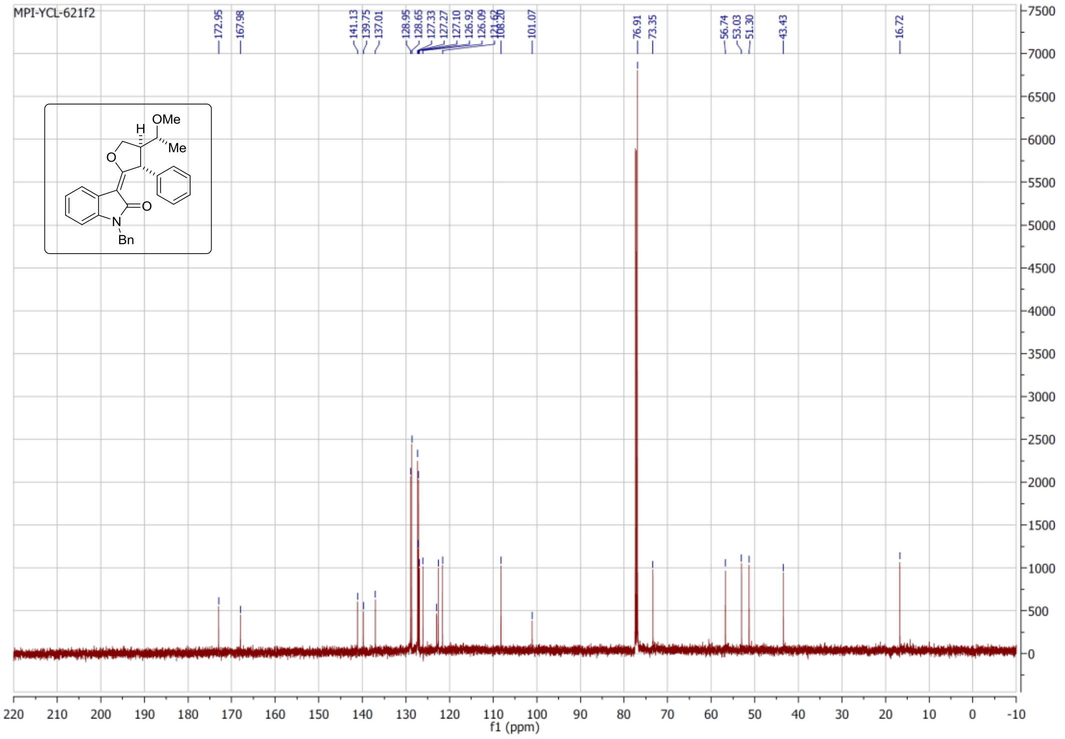

Supplementary Figure 106. ^{1}H and ^{13}C NMR spectra for **4l**.

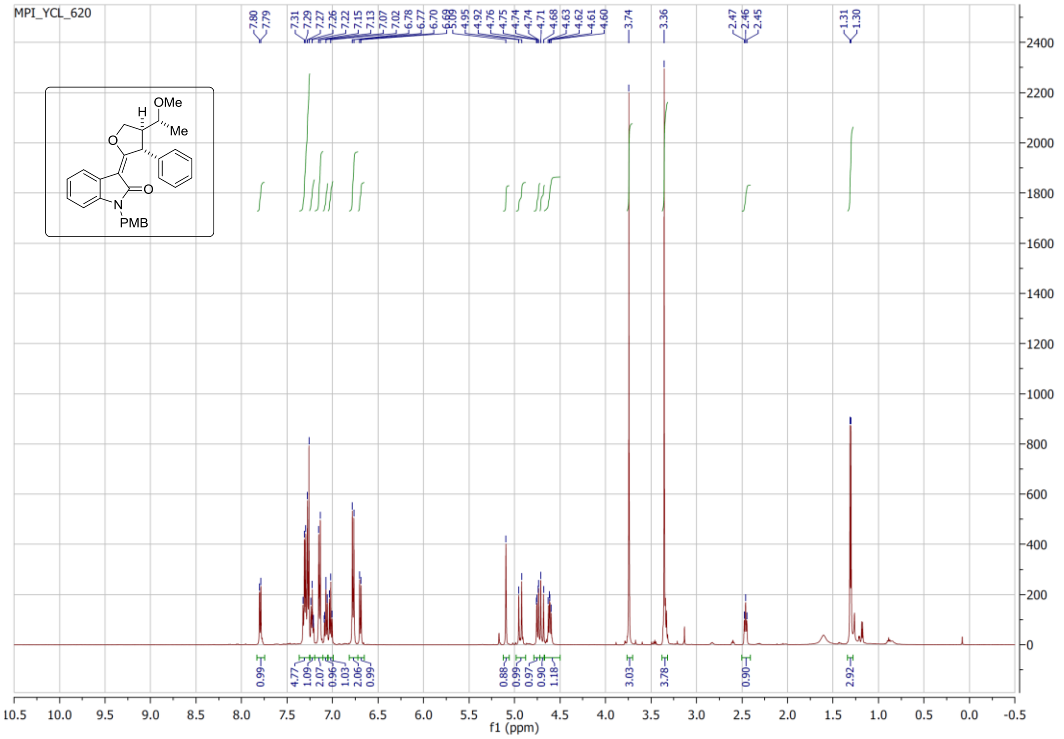

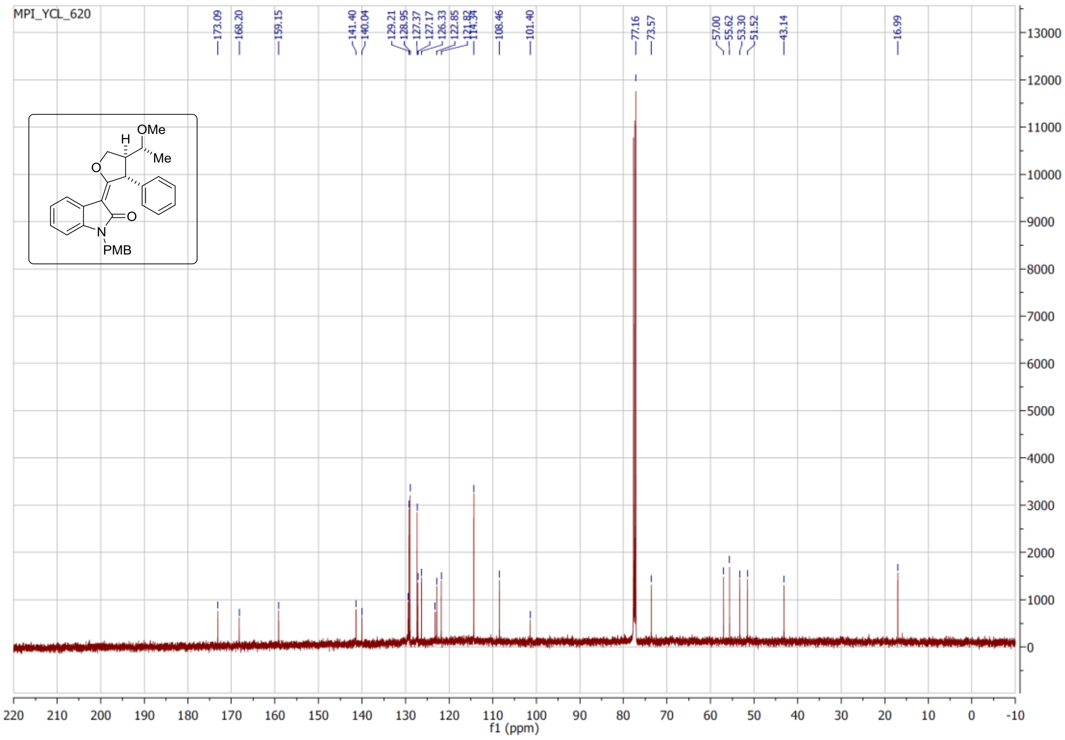

Supplementary Figure 107. ^{1}H and ^{13}C NMR spectra for **4m**.

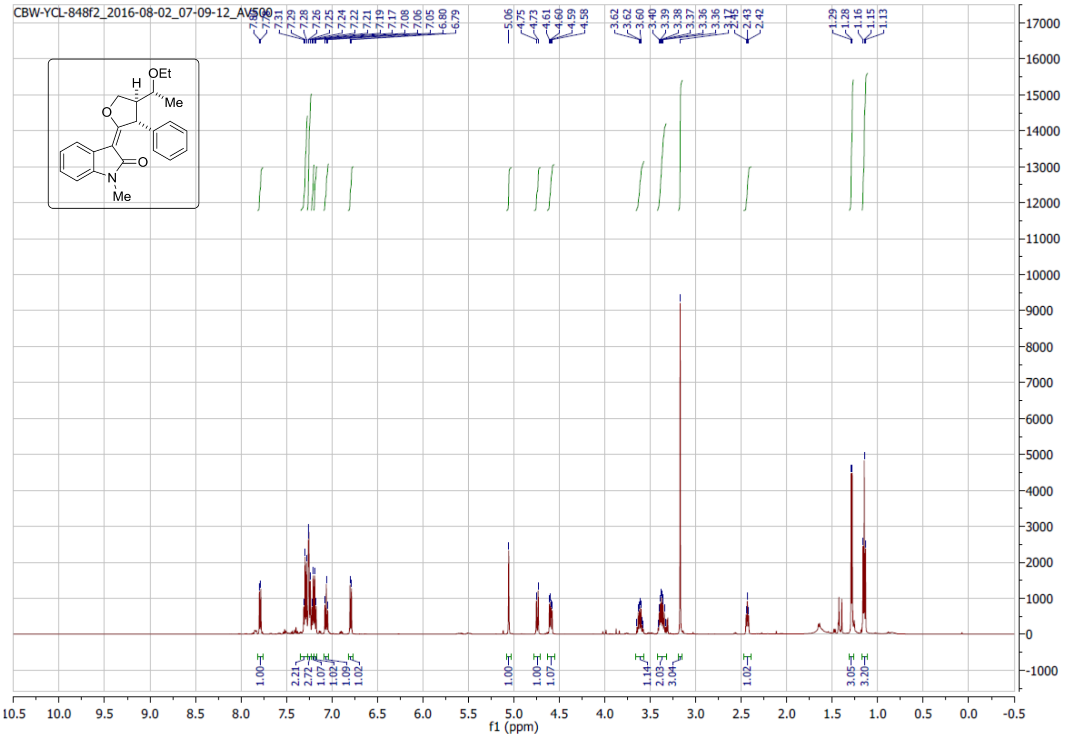

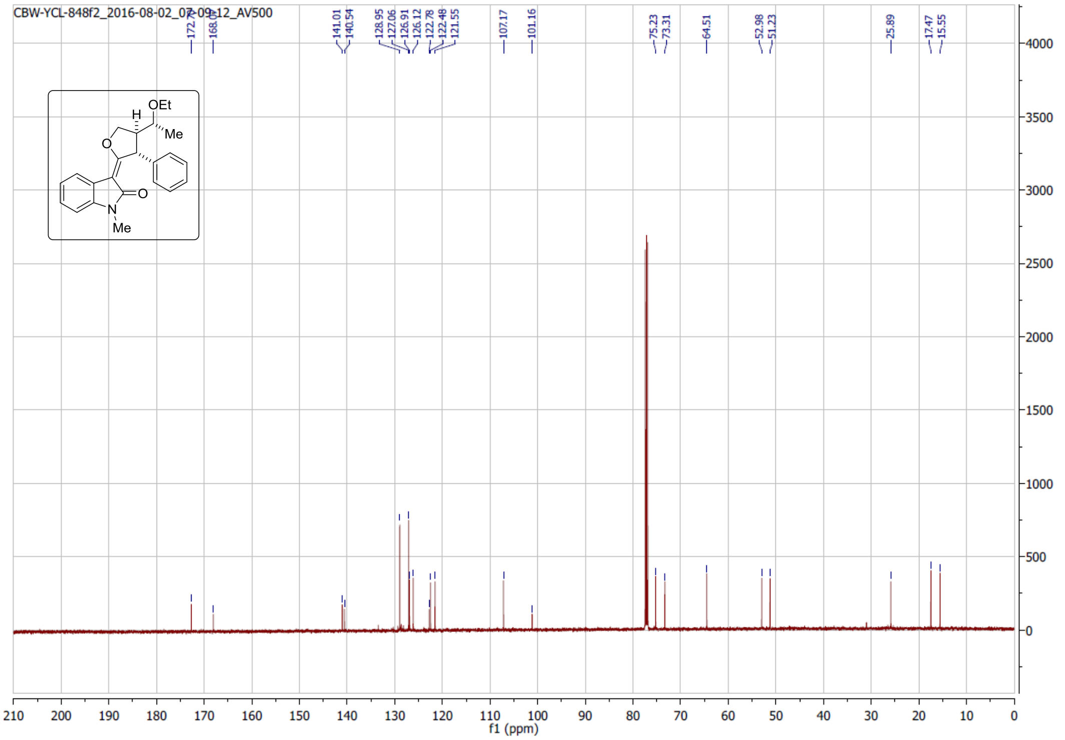

Supplementary Figure 108. ^{1}H and ^{13}C NMR spectra for **4n**.

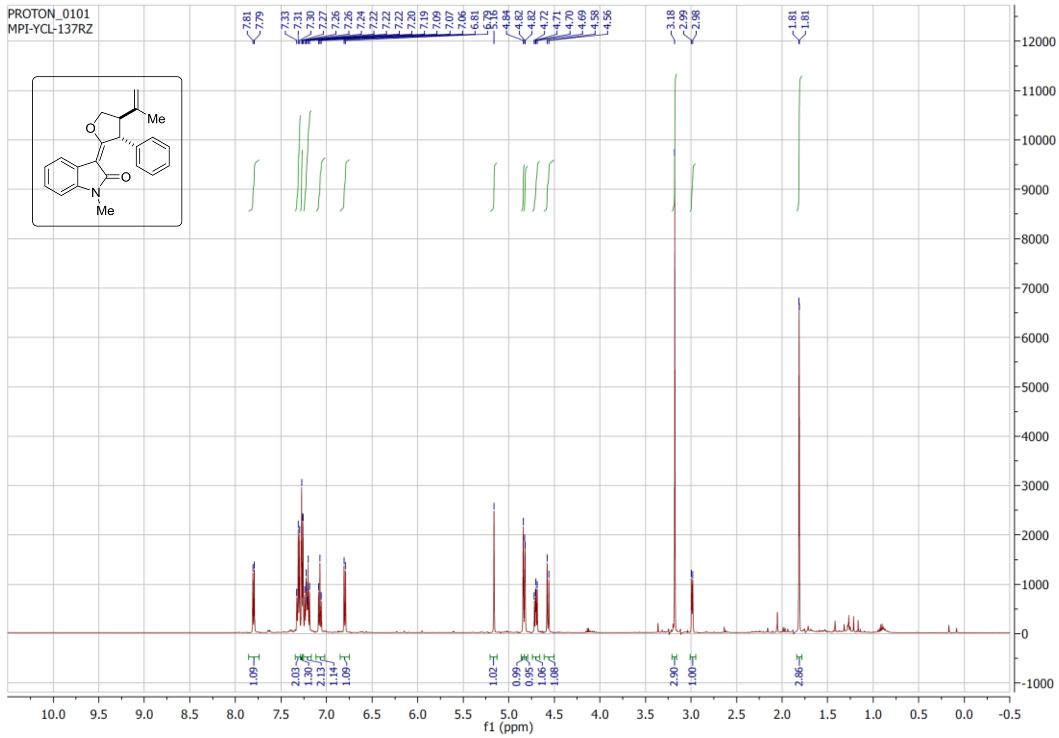

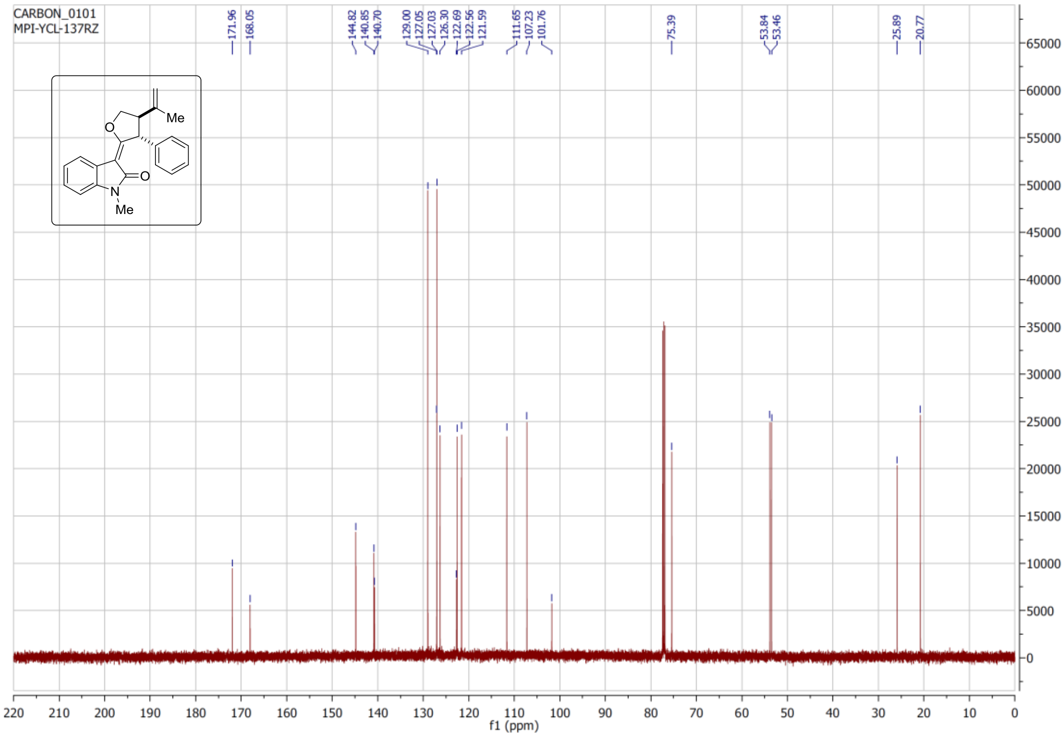

Supplementary Figure 109. ^{1}H and ^{13}C NMR spectra for 1**4a**.

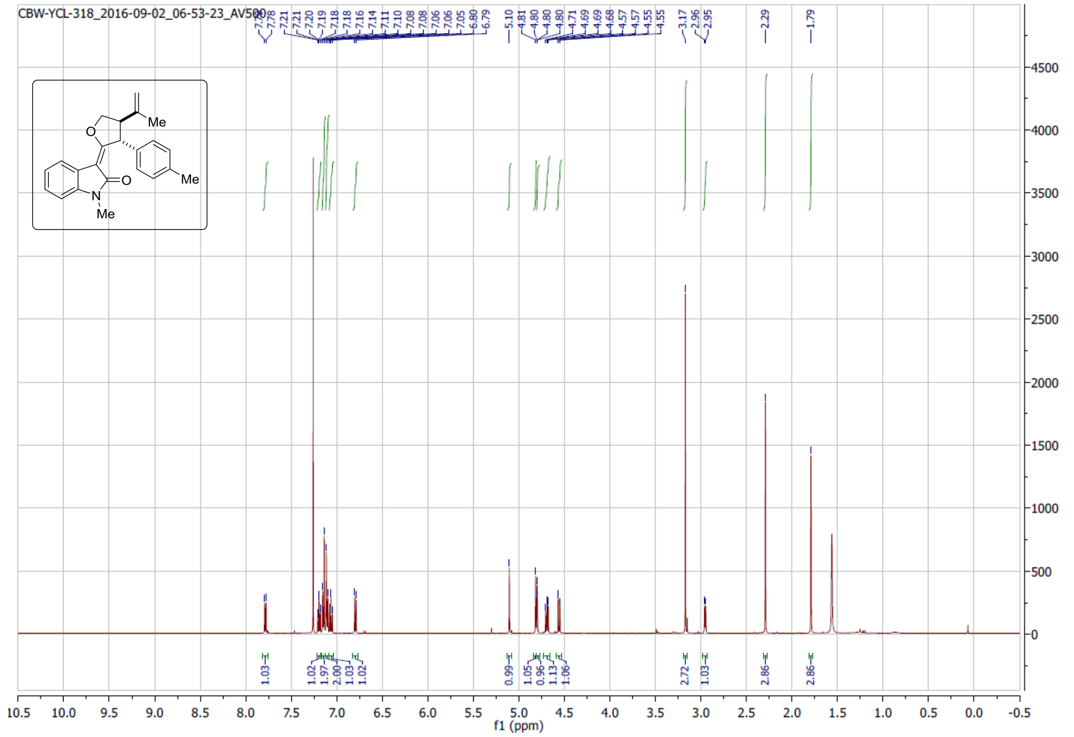

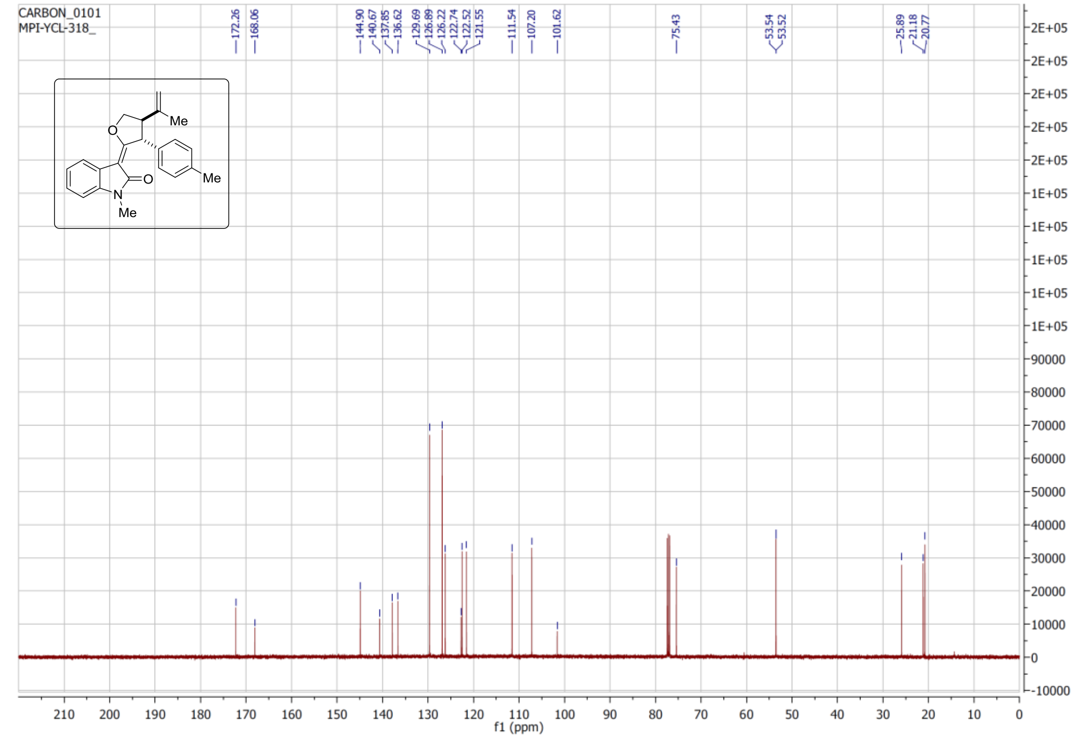

Supplementary Figure 110. ^{1}H and ^{13}C NMR spectra for 1**4b**.

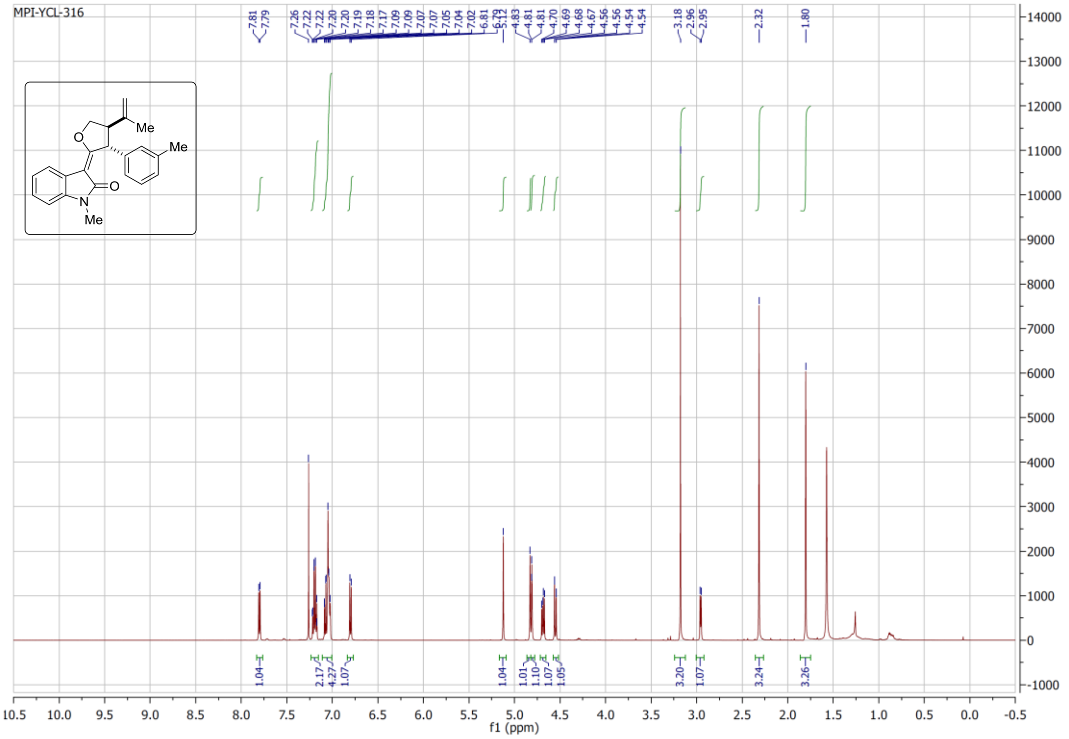

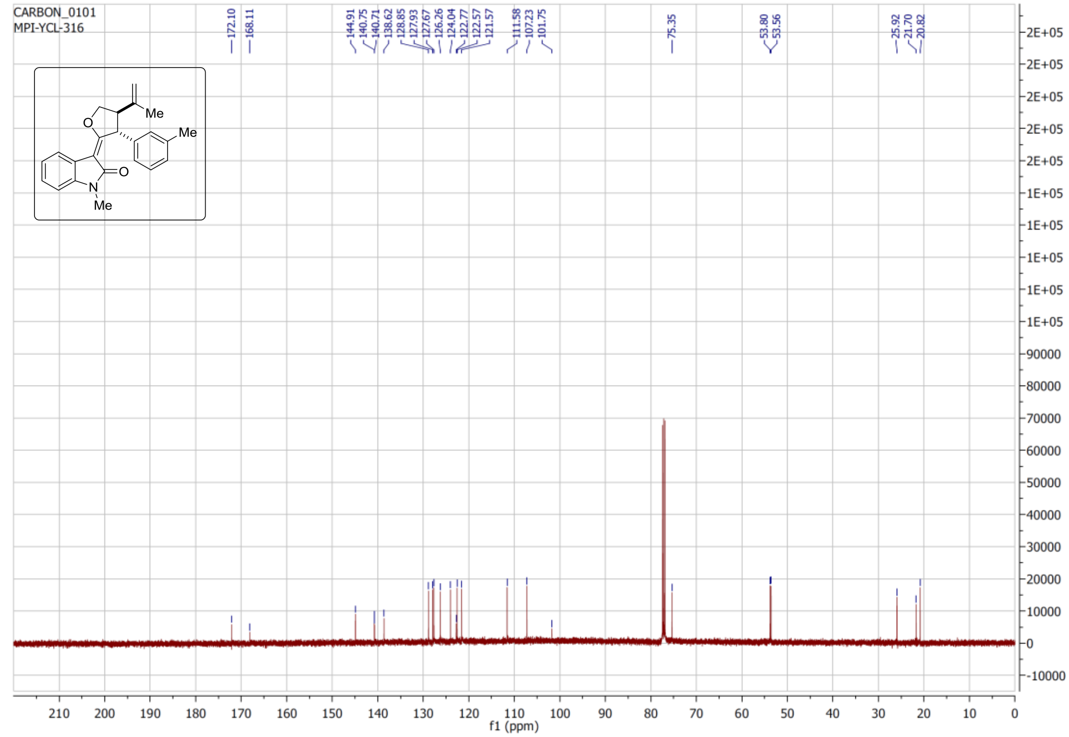

Supplementary Figure 111. ^{1}H and ^{13}C NMR spectra for 1**4c**.

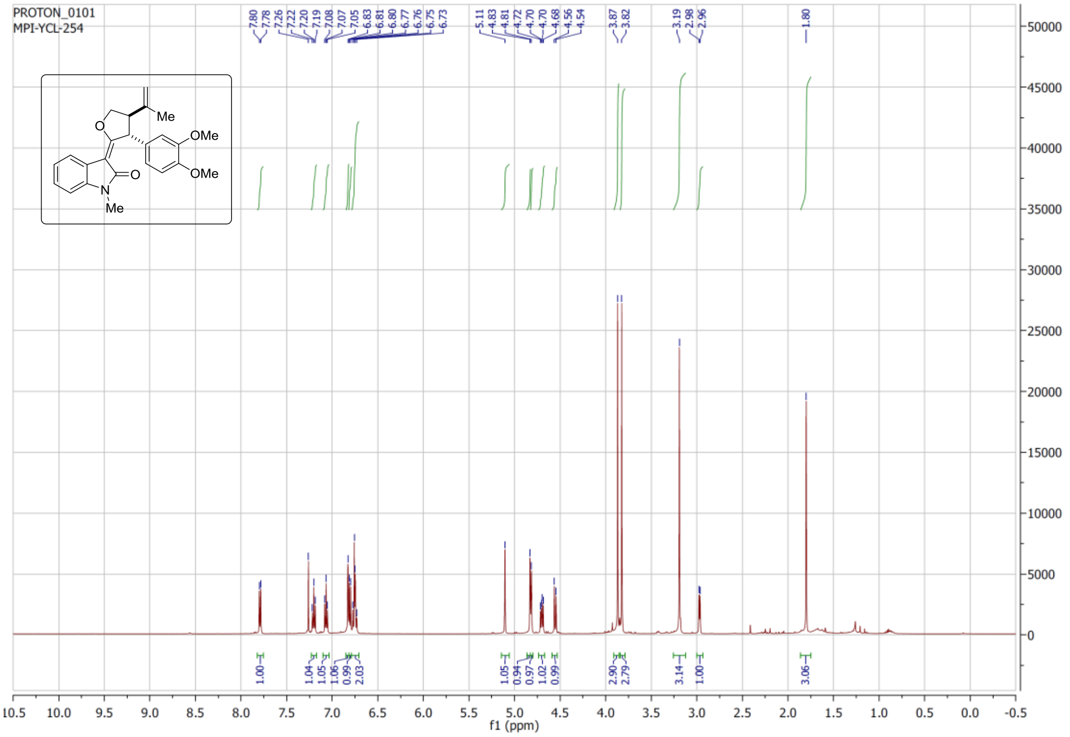

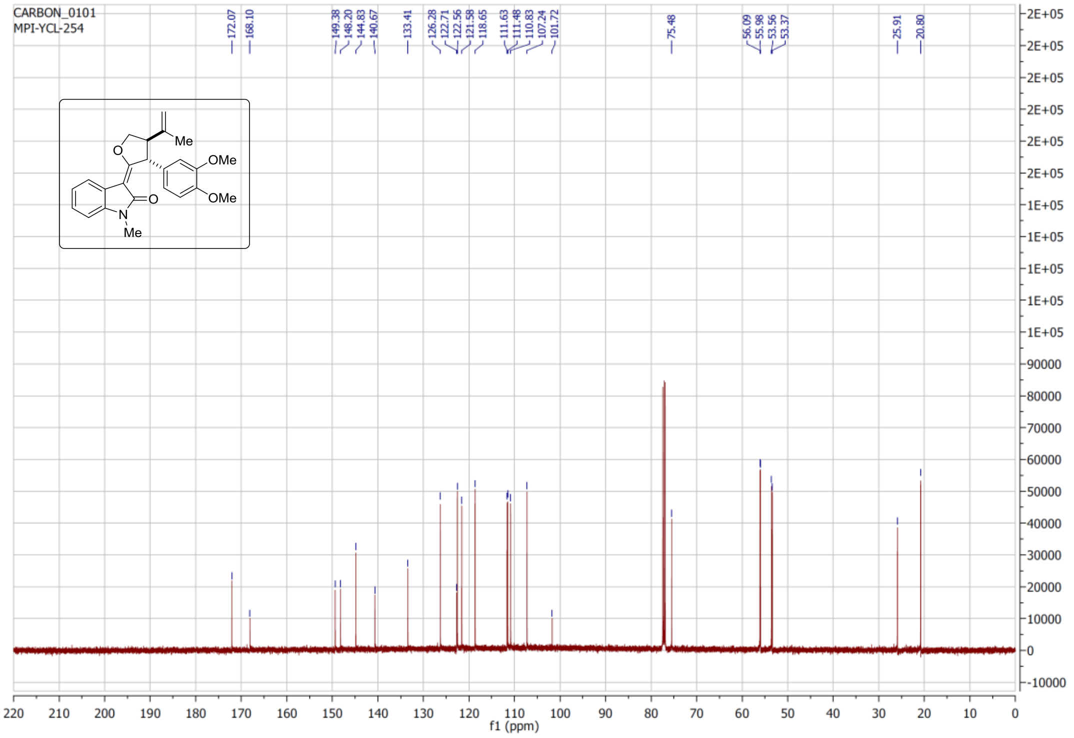

Supplementary Figure 112. ^{1}H and ^{13}C NMR spectra for 1**4d**.

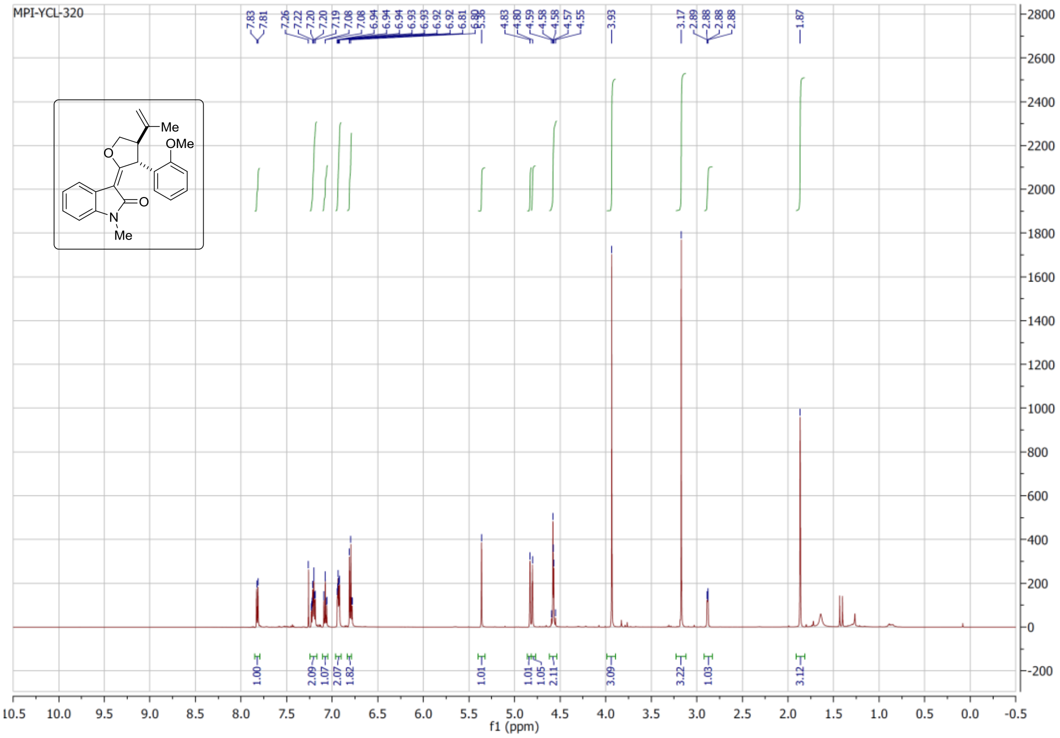

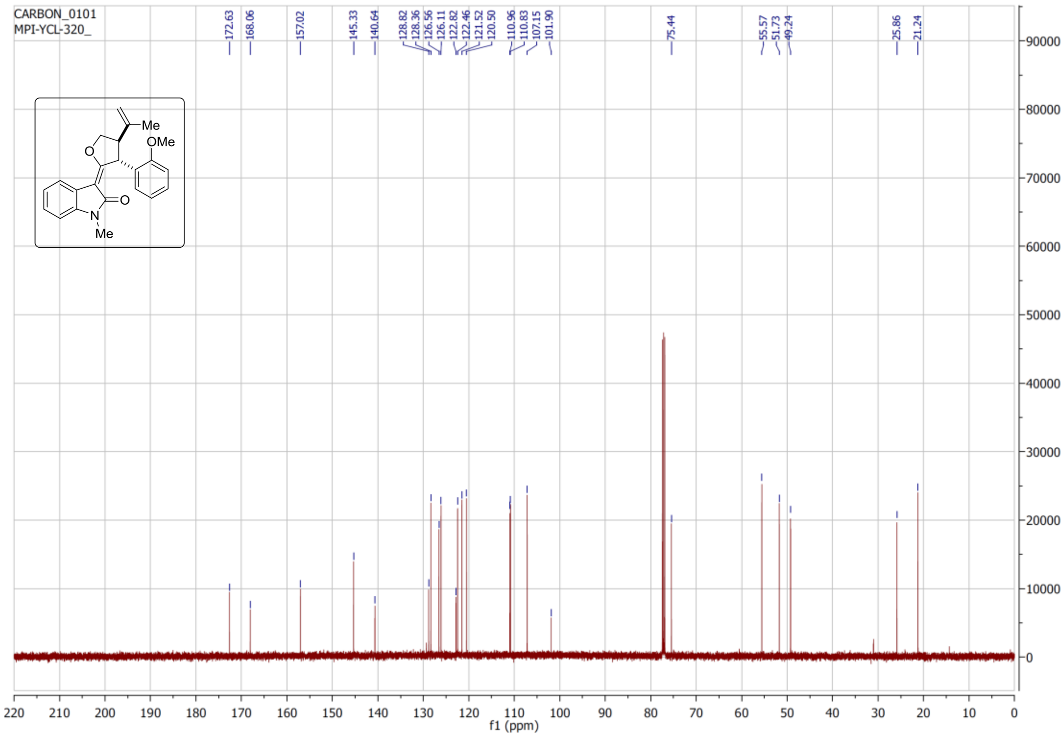

Supplementary Figure 113. ^{1}H and ^{13}C NMR spectra for 1**4e**.

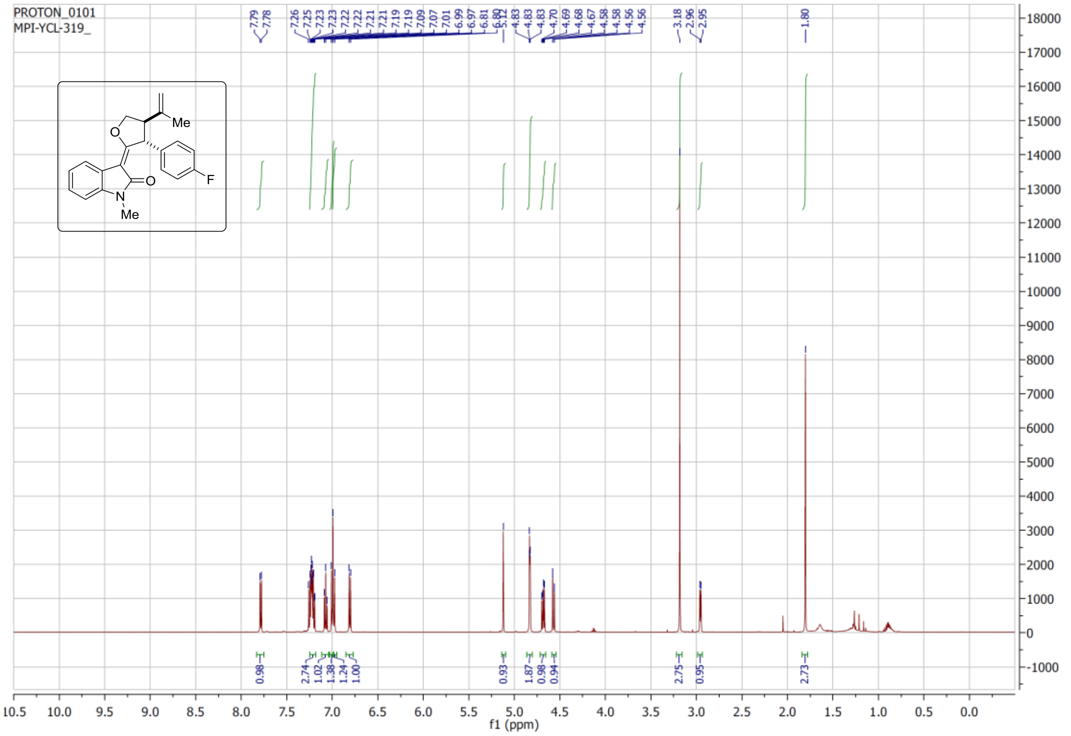

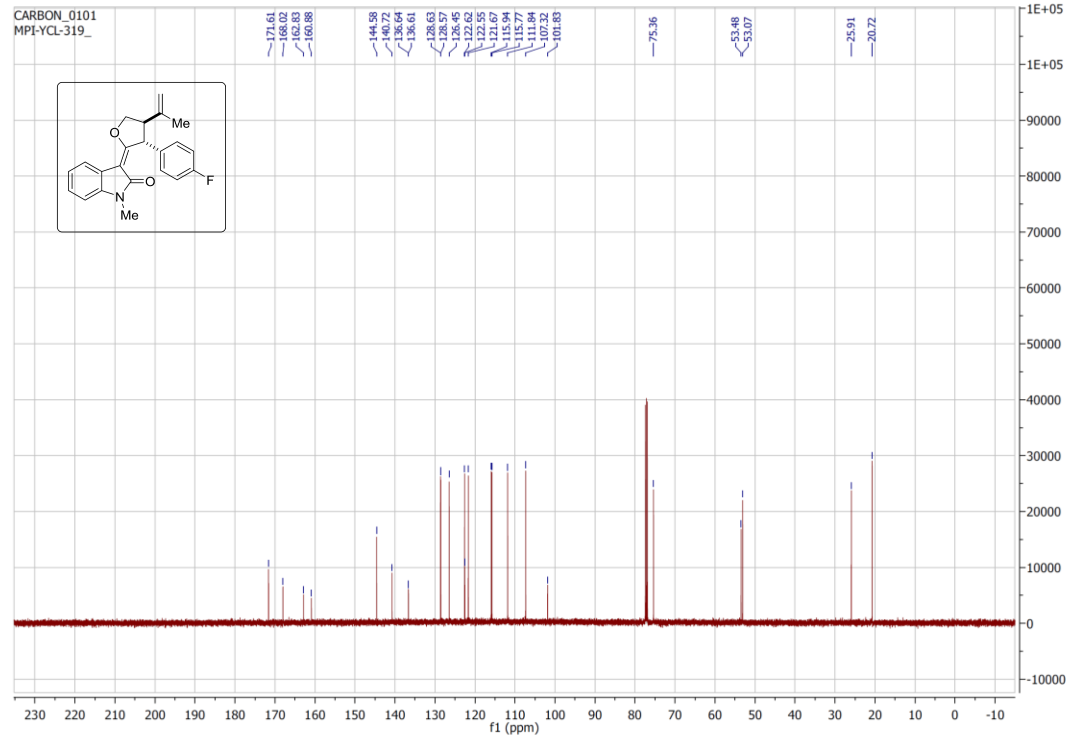

Supplementary Figure 114. ^{1}H and ^{13}C NMR spectra for 1**4f**.

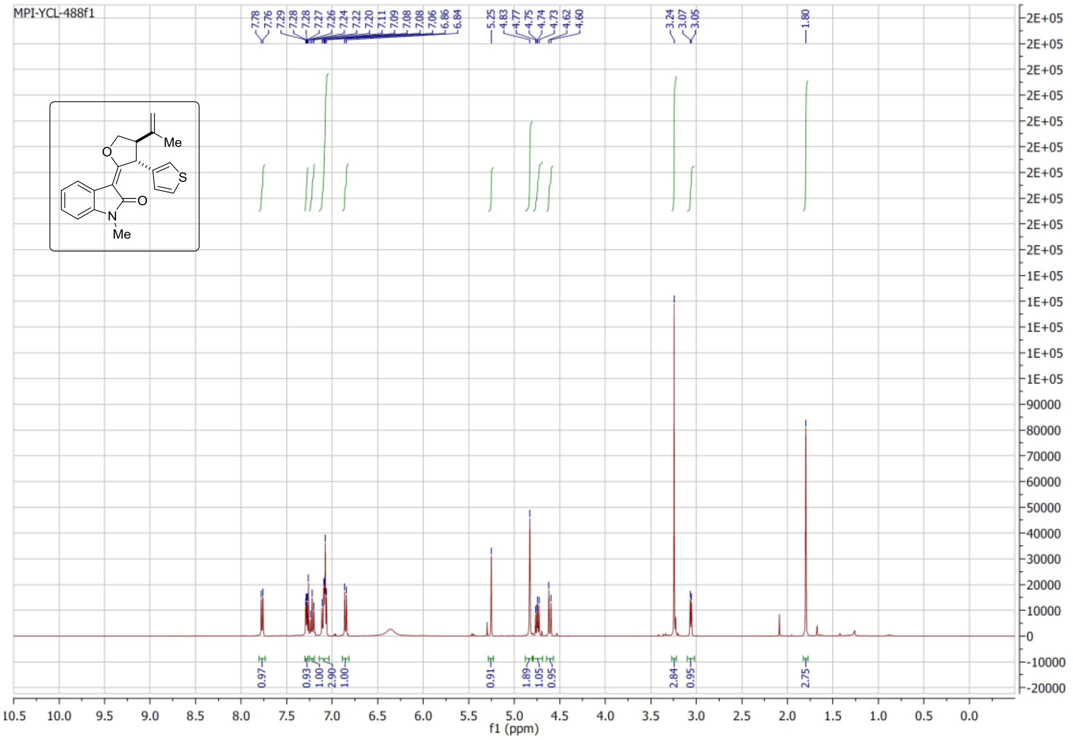

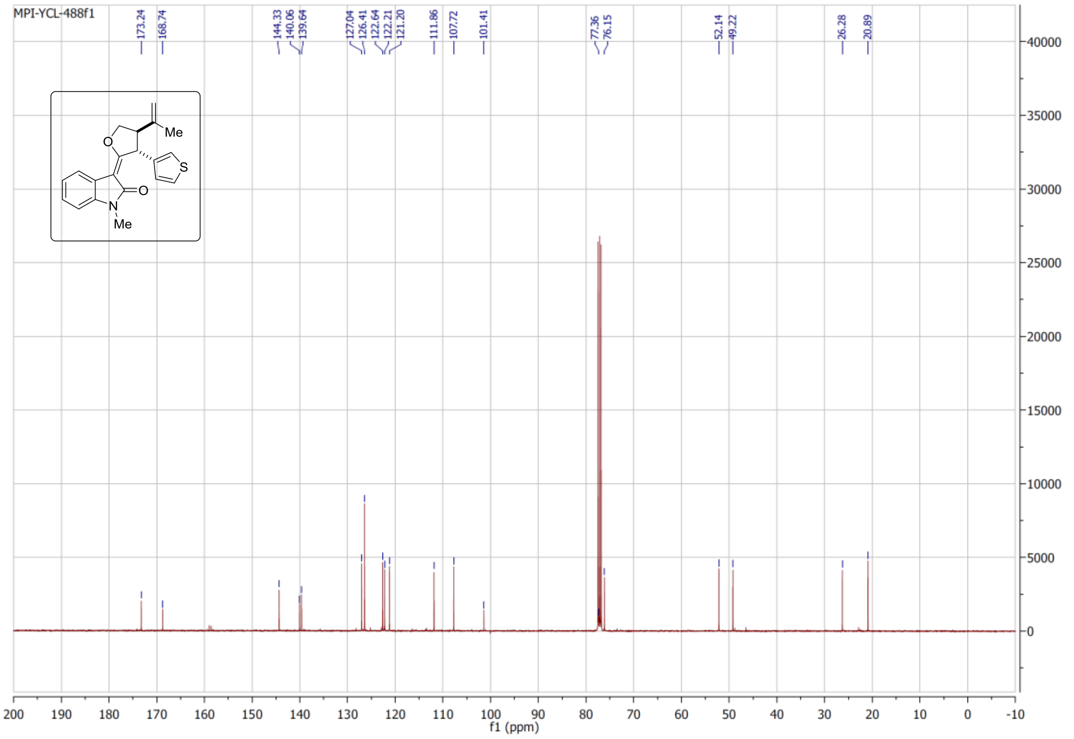

Supplementary Figure 115. ^{1}H and ^{13}C NMR spectra for 1**4g**.

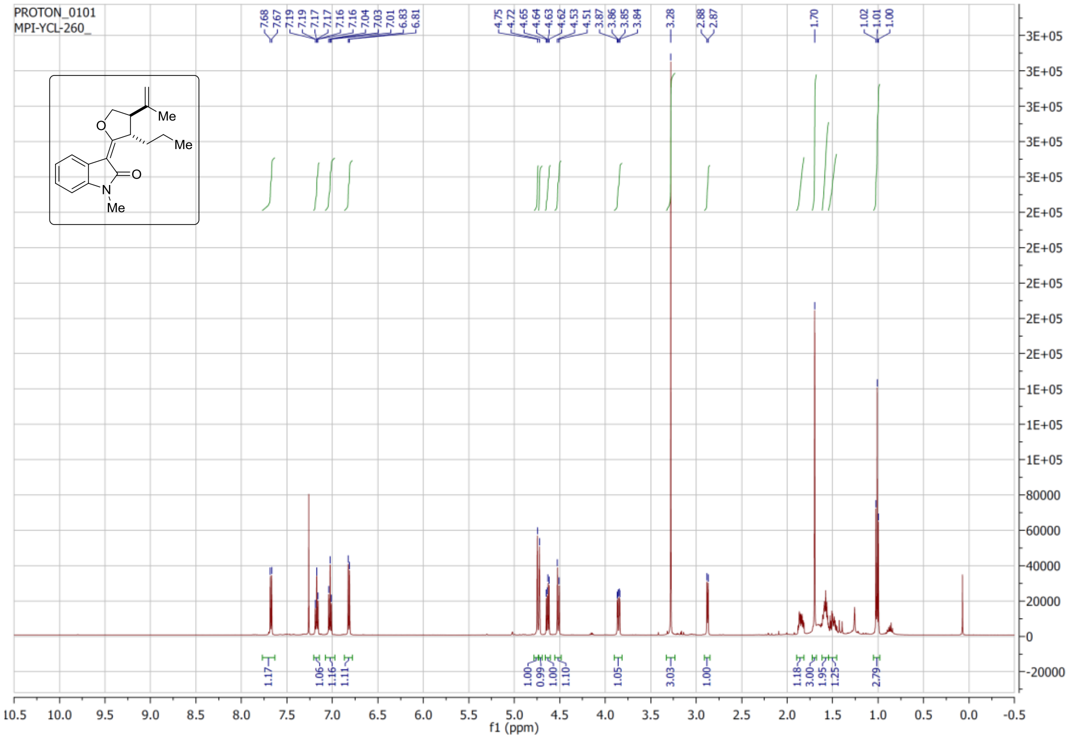

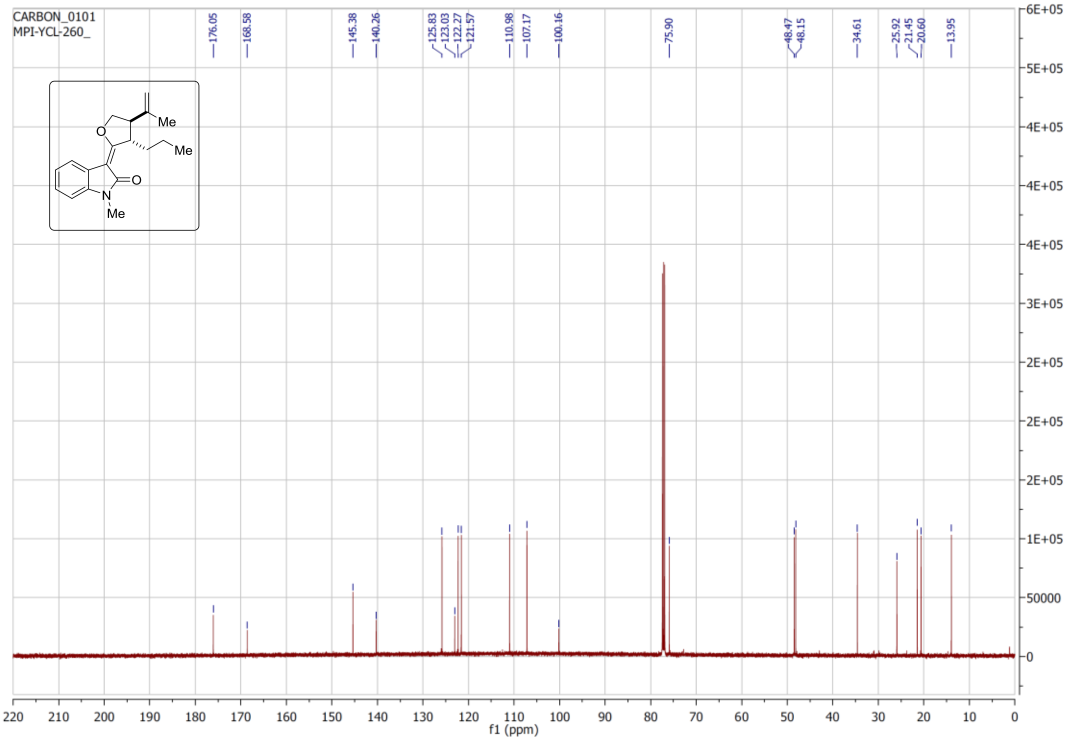

Supplementary Figure 116. ^{1}H and ^{13}C NMR spectra for 1**4h**.

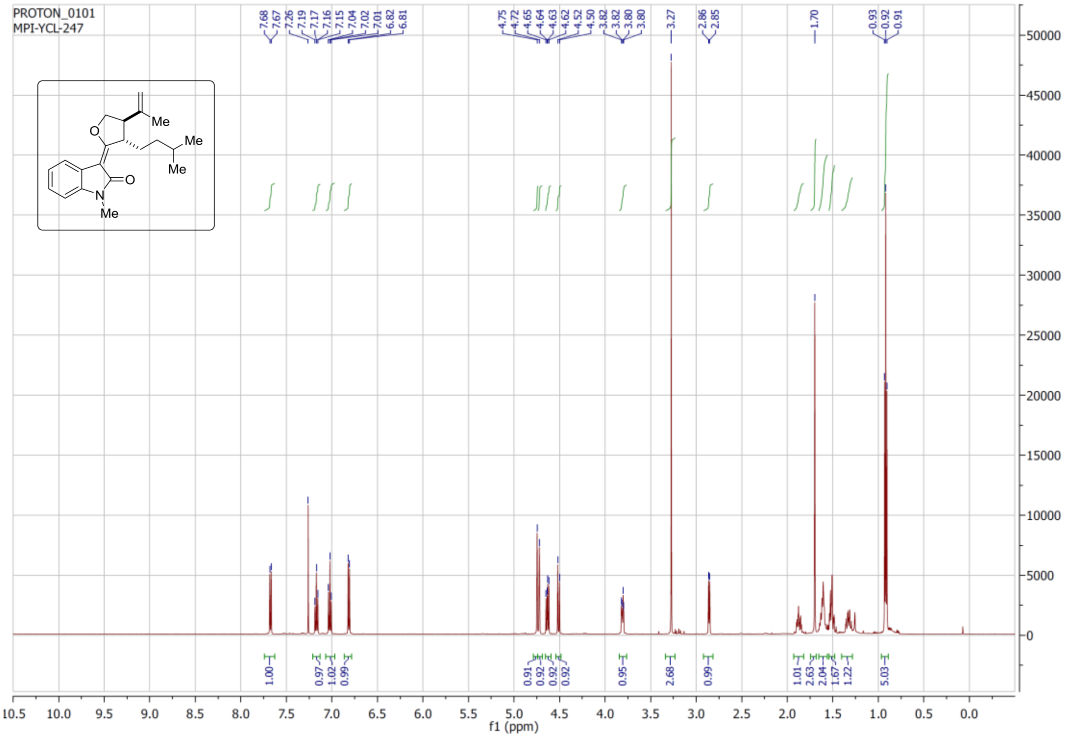

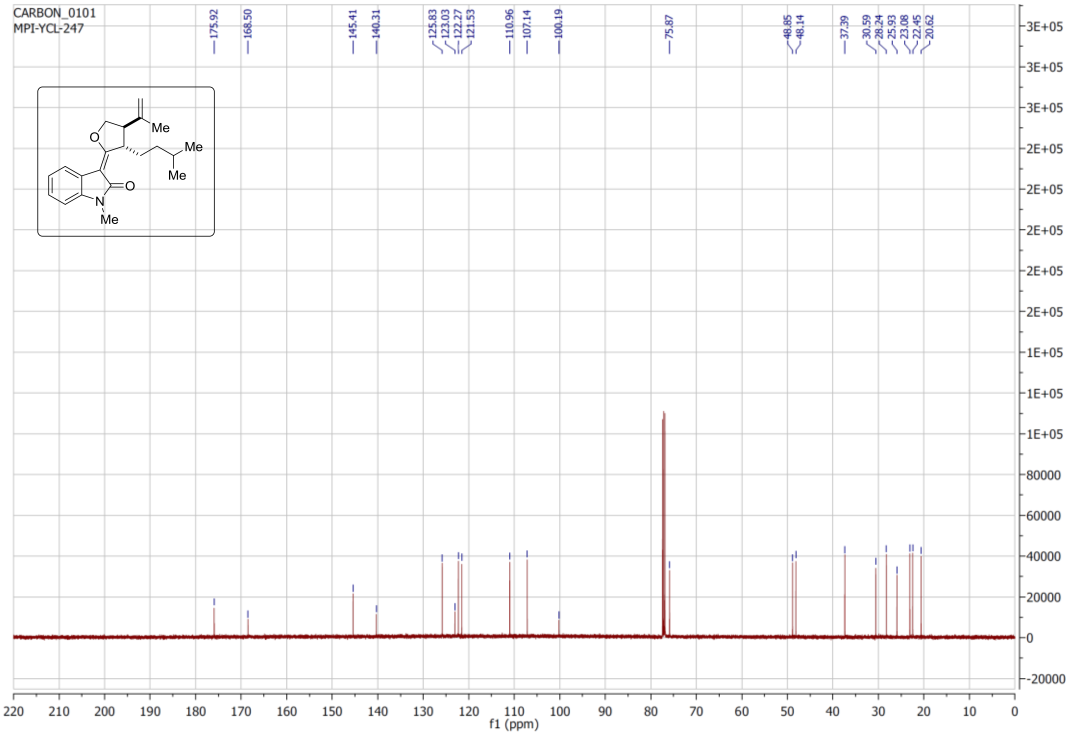

Supplementary Figure 117. ^{1}H and ^{13}C NMR spectra for 1**4i**.

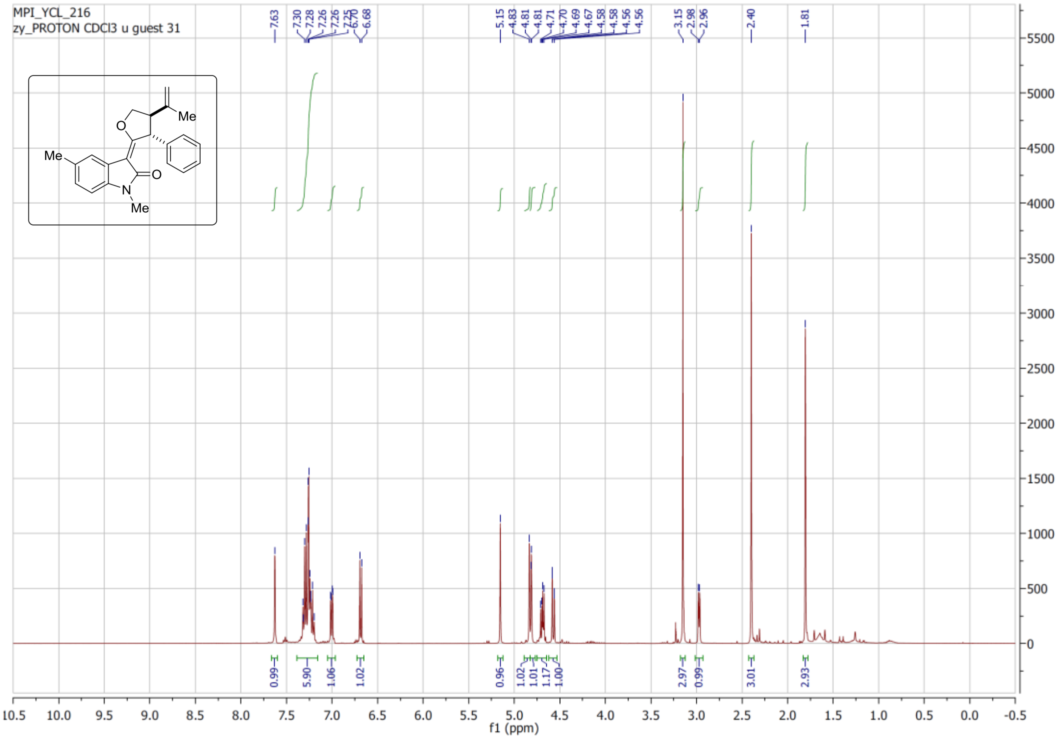

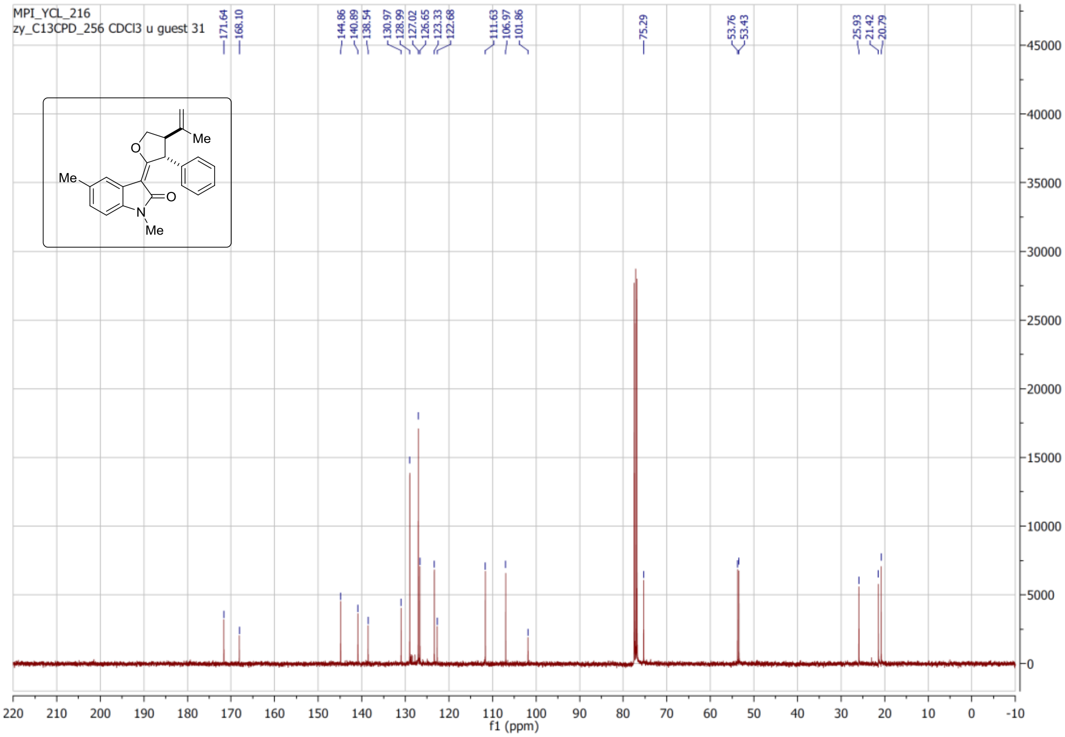

Supplementary Figure 118. ^{1}H and ^{13}C NMR spectra for 1**4j**.

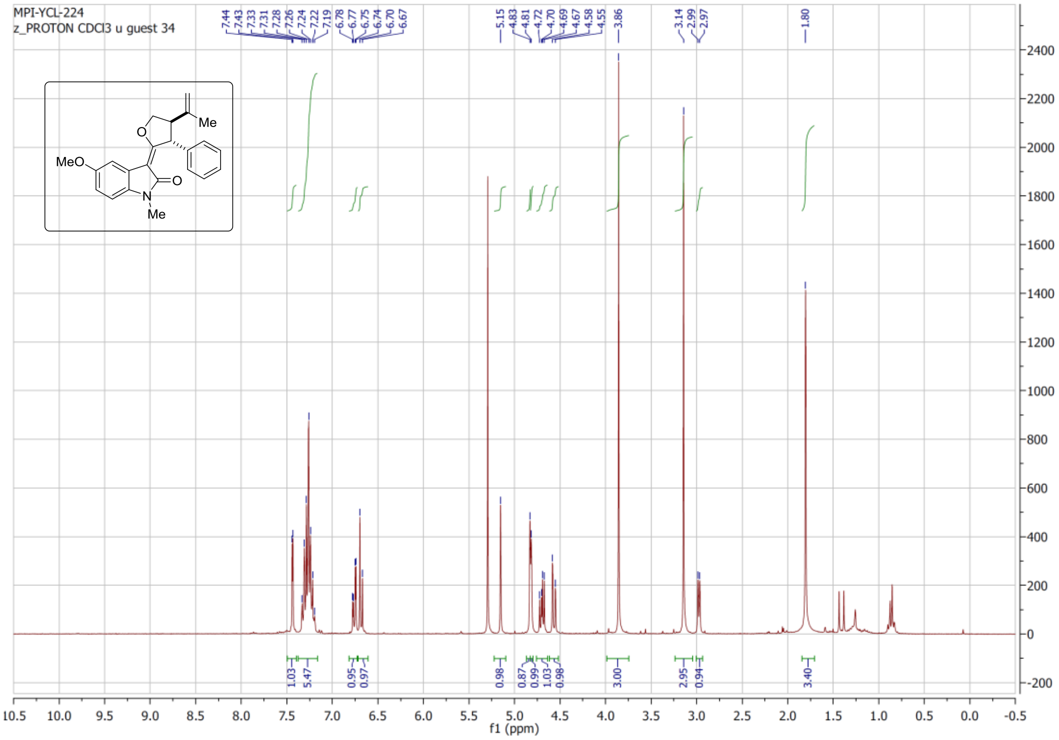

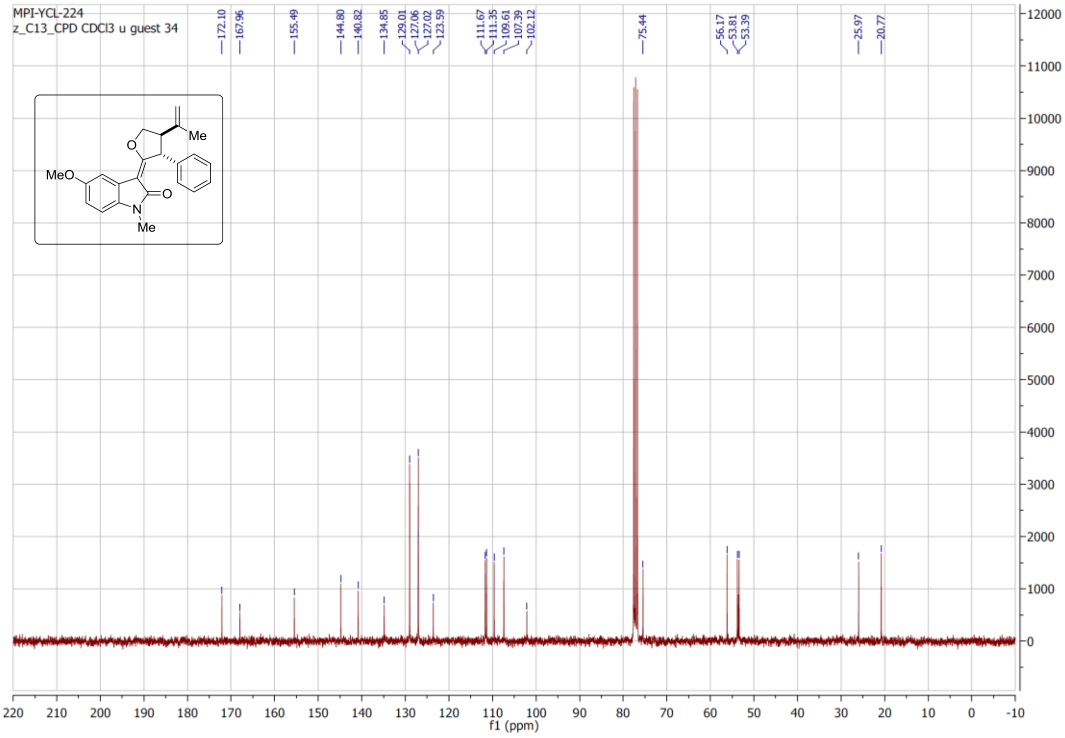

Supplementary Figure 119. ^{1}H and ^{13}C NMR spectra for 1**4k**.

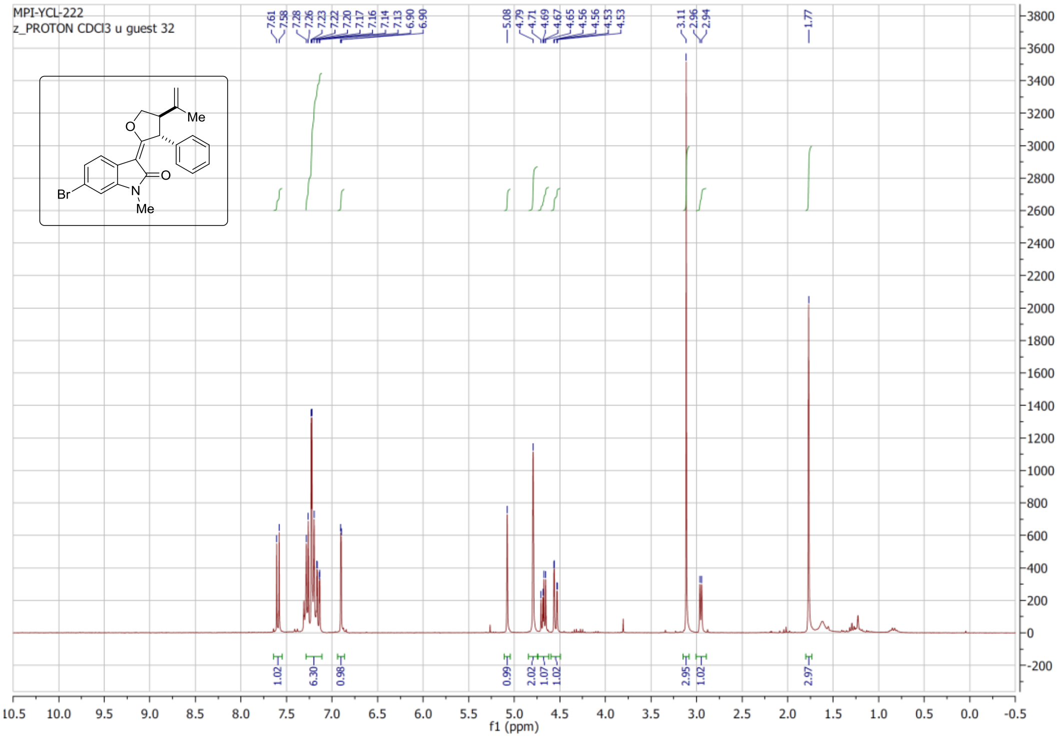

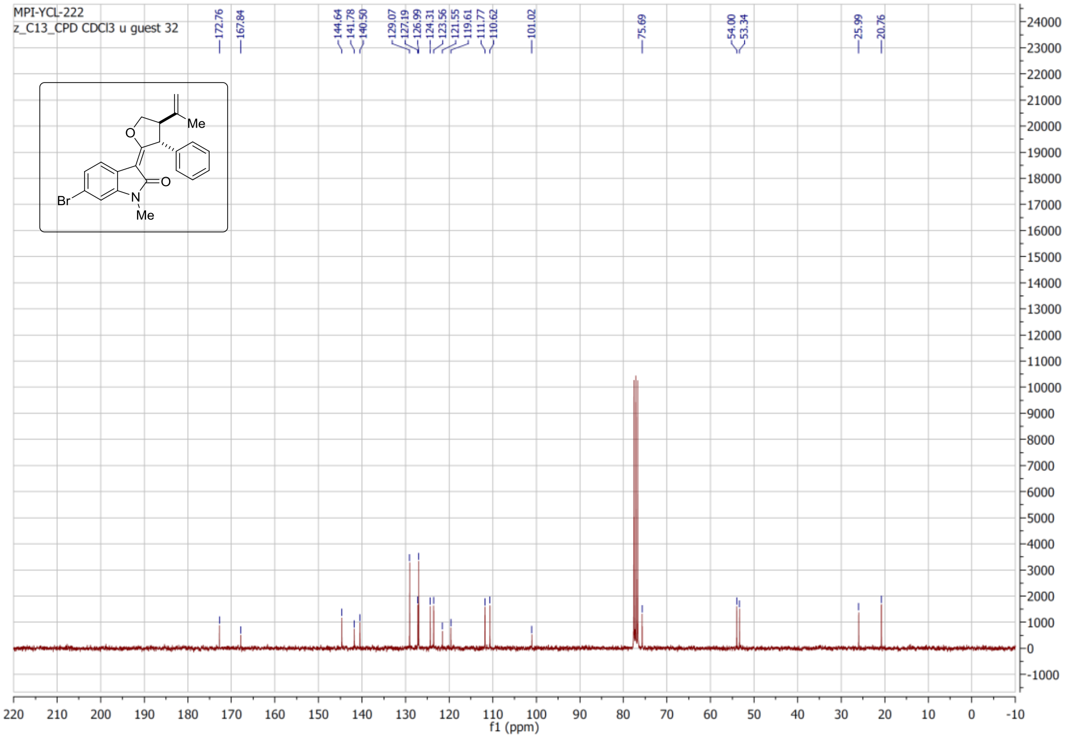

Supplementary Figure 120. ^{1}H and ^{13}C NMR spectra for 1**4l**.

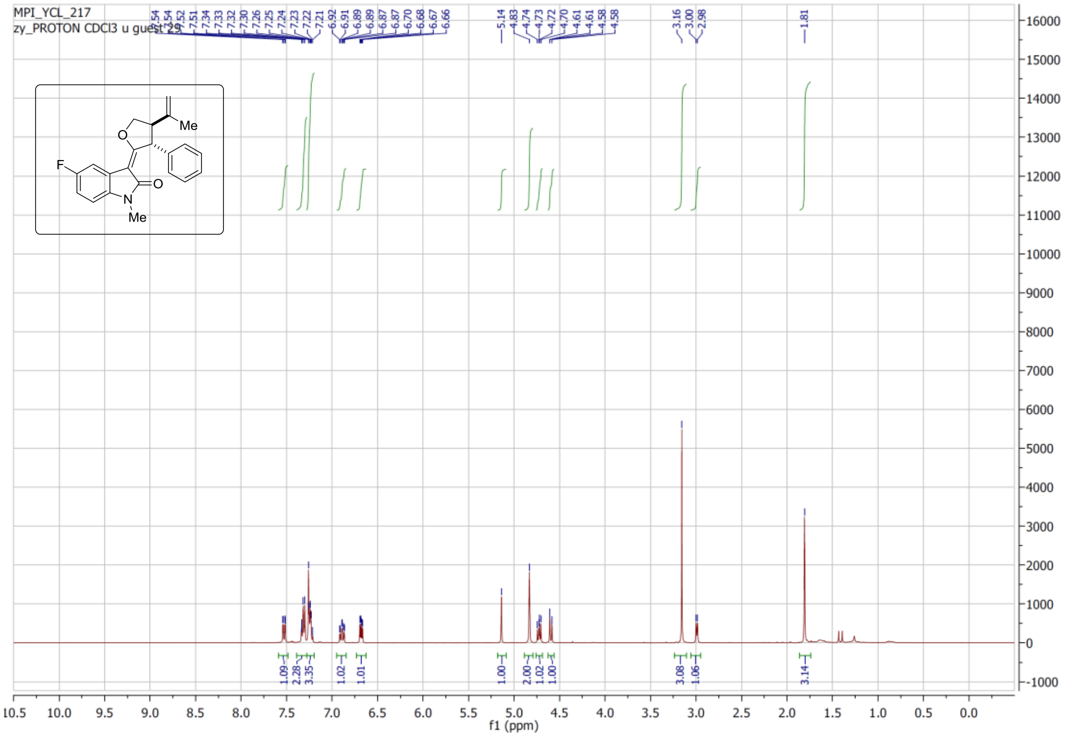

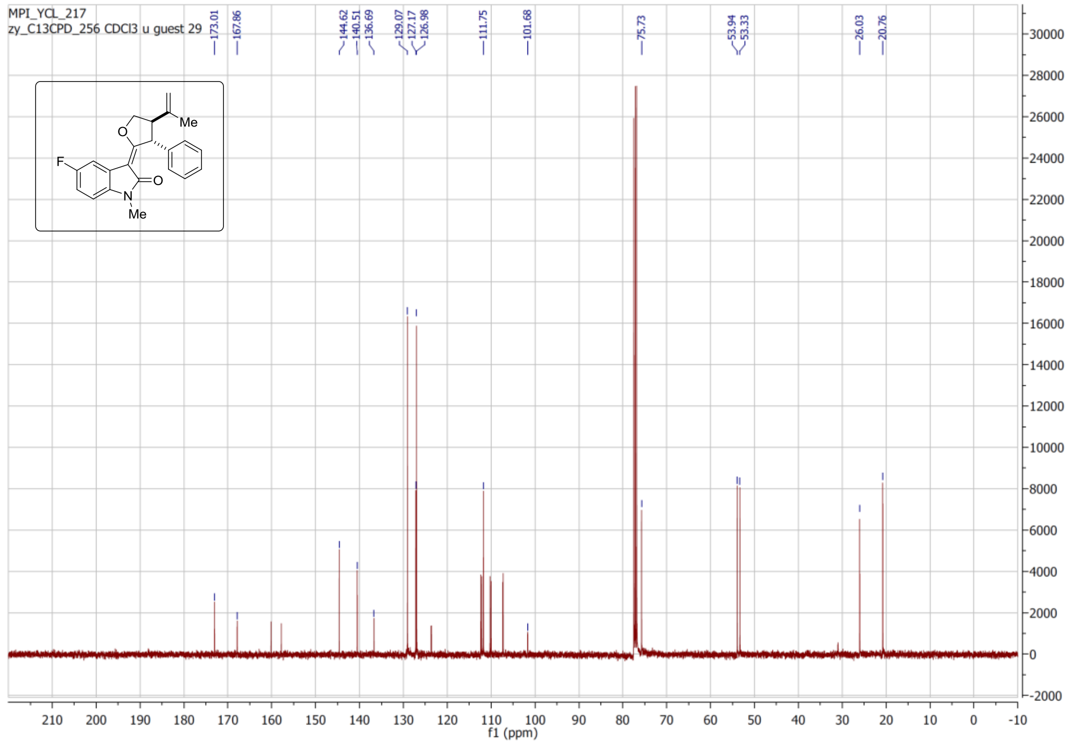

Supplementary Figure 121. ^{1}H and ^{13}C NMR spectra for 1**4m**.

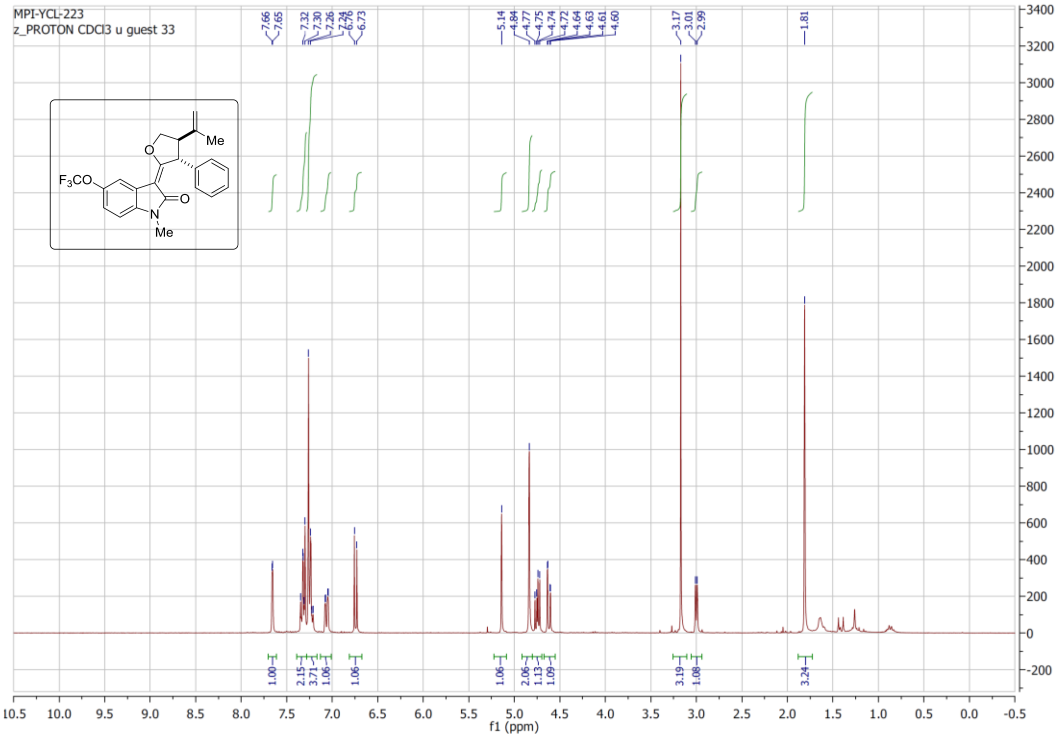

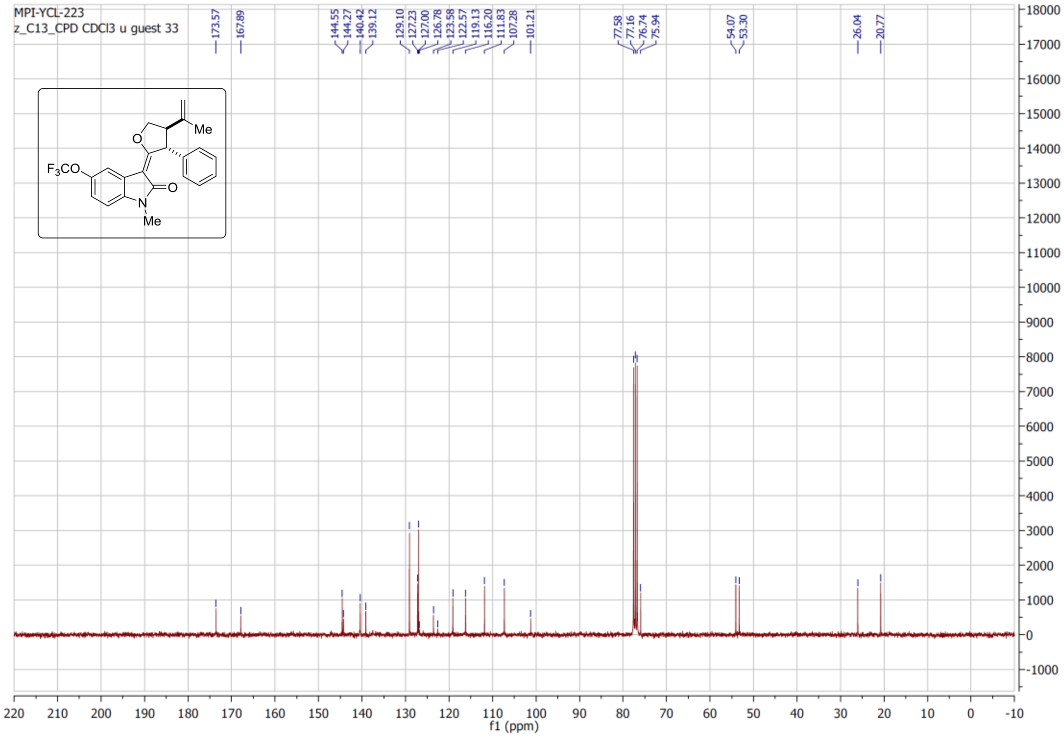

Supplementary Figure 122. ^{1}H and ^{13}C NMR spectra for 1**4n**.

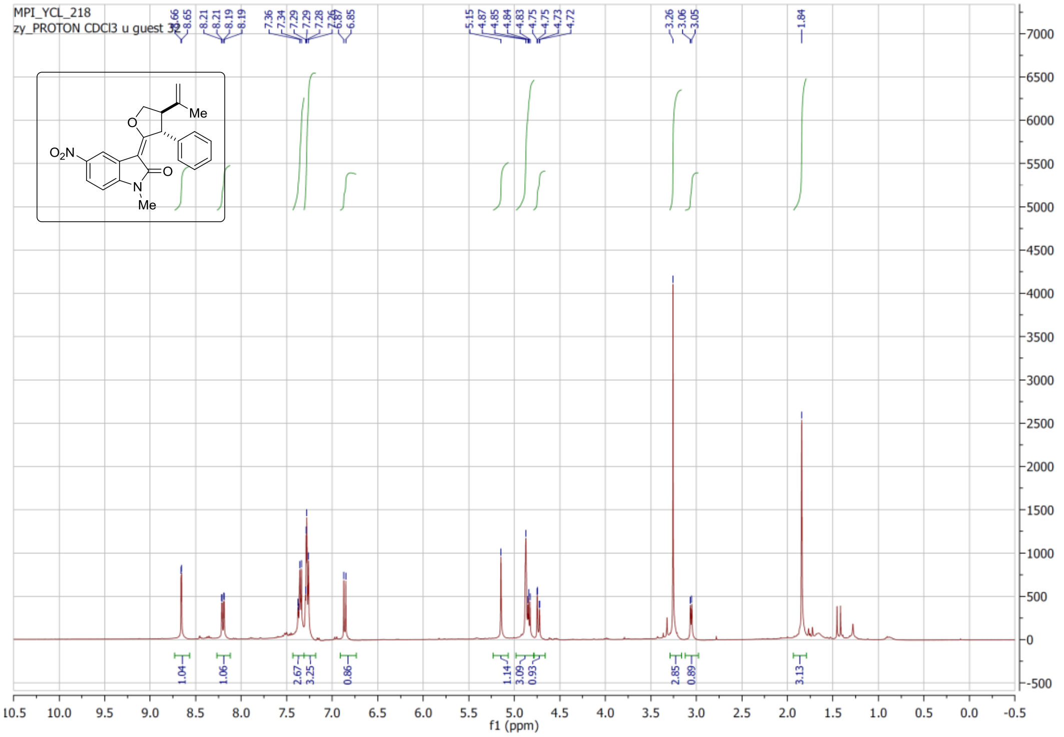

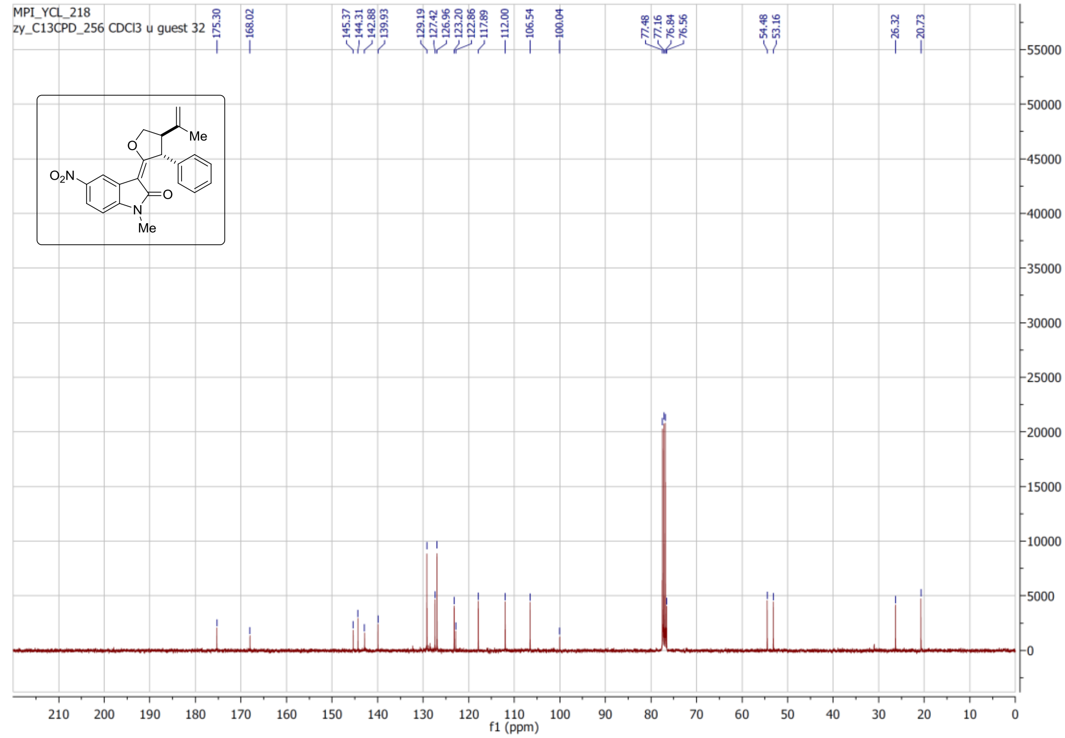

Supplementary Figure 123. ^{1}H and ^{13}C NMR spectra for 1**4o**.

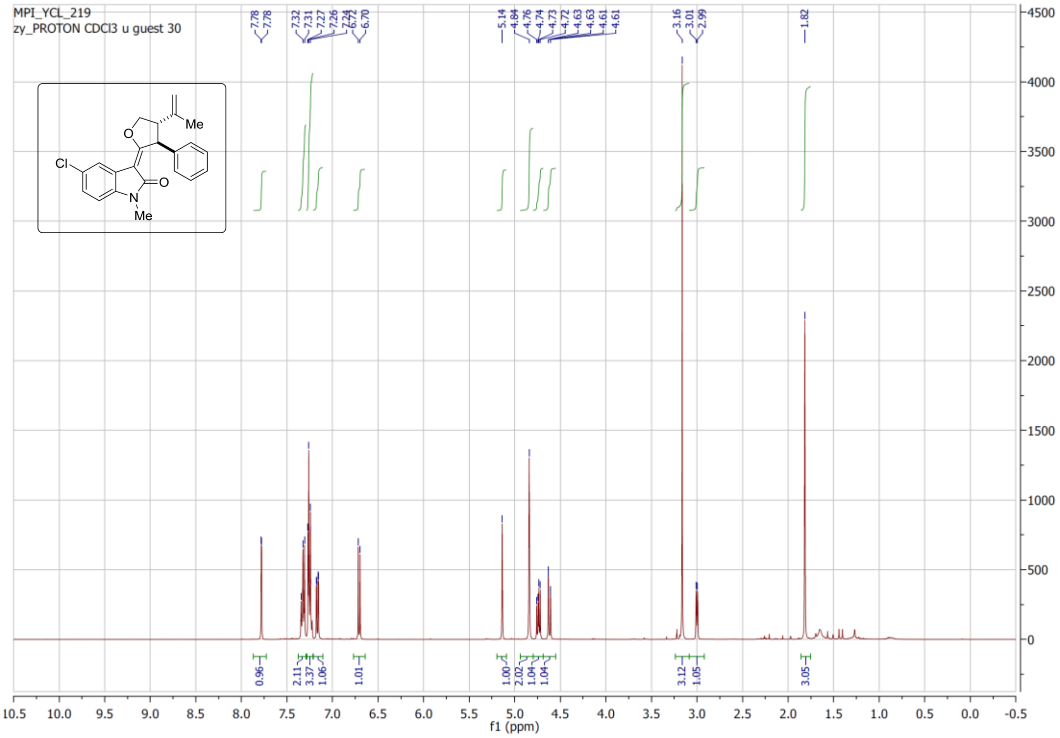

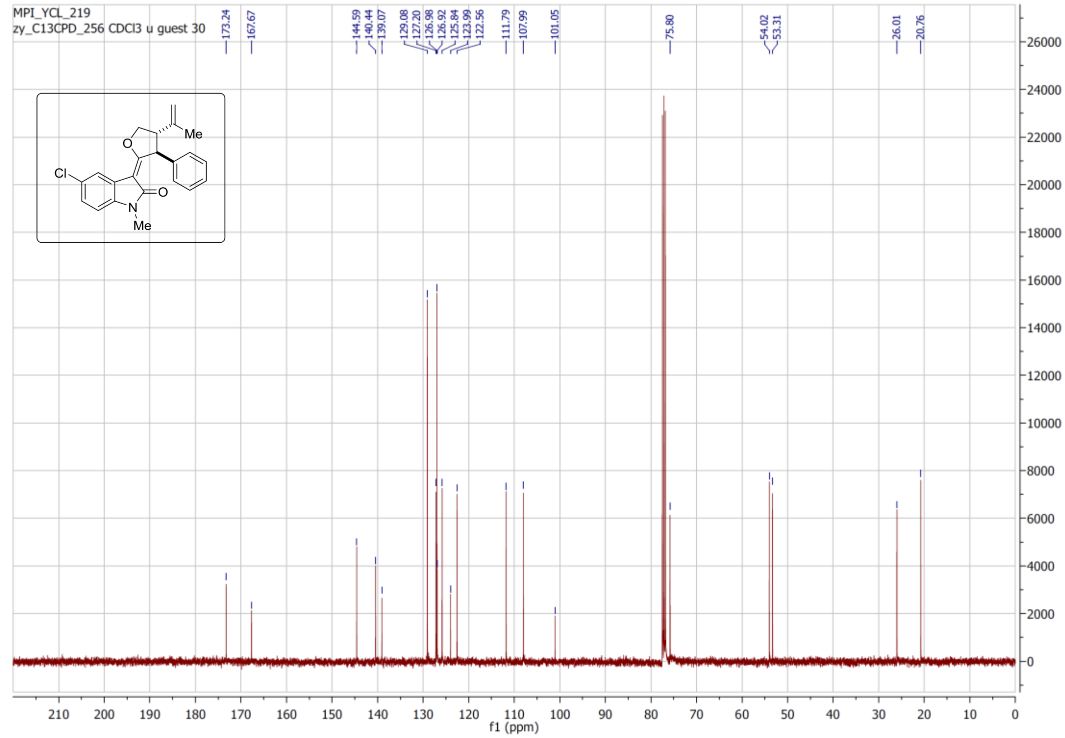

Supplementary Figure 124. ^{1}H and ^{13}C NMR spectra for 1**4p**.

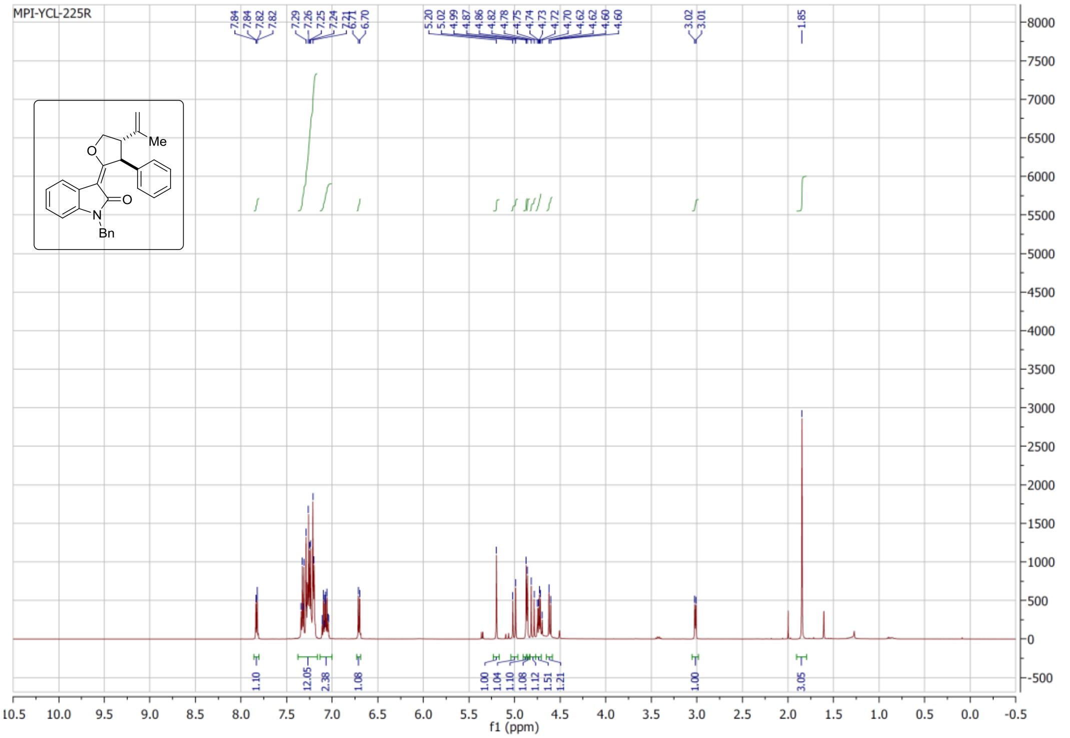

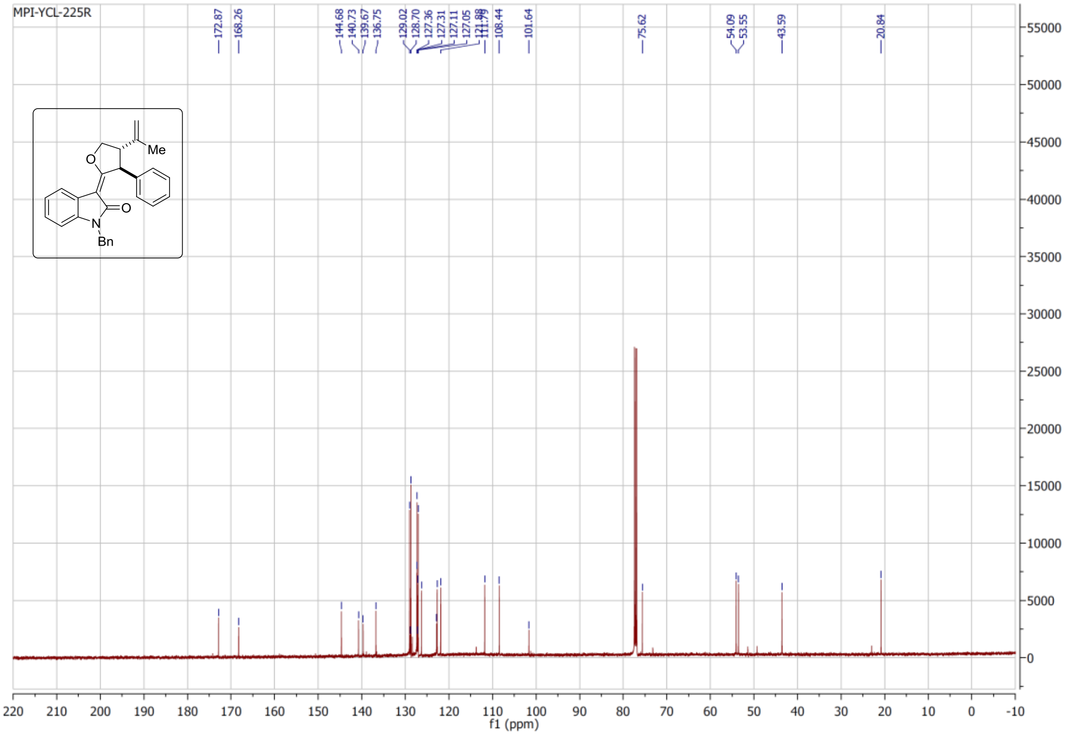

Supplementary Figure 125. ^{1}H and ^{13}C NMR spectra for 1**4q**.

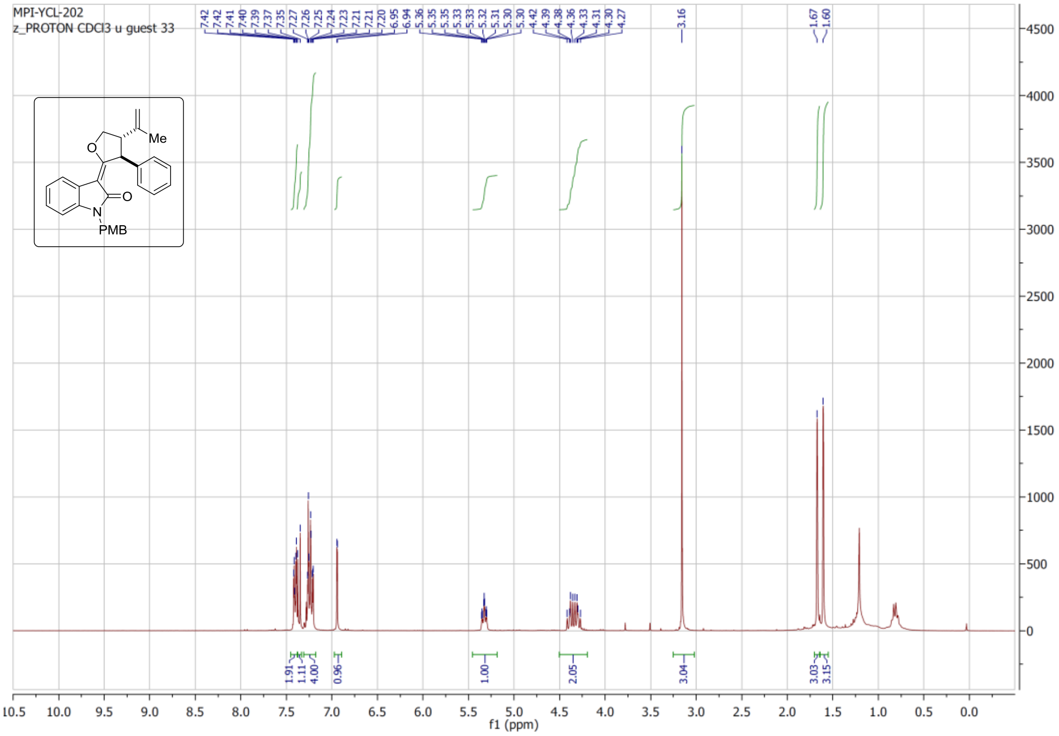

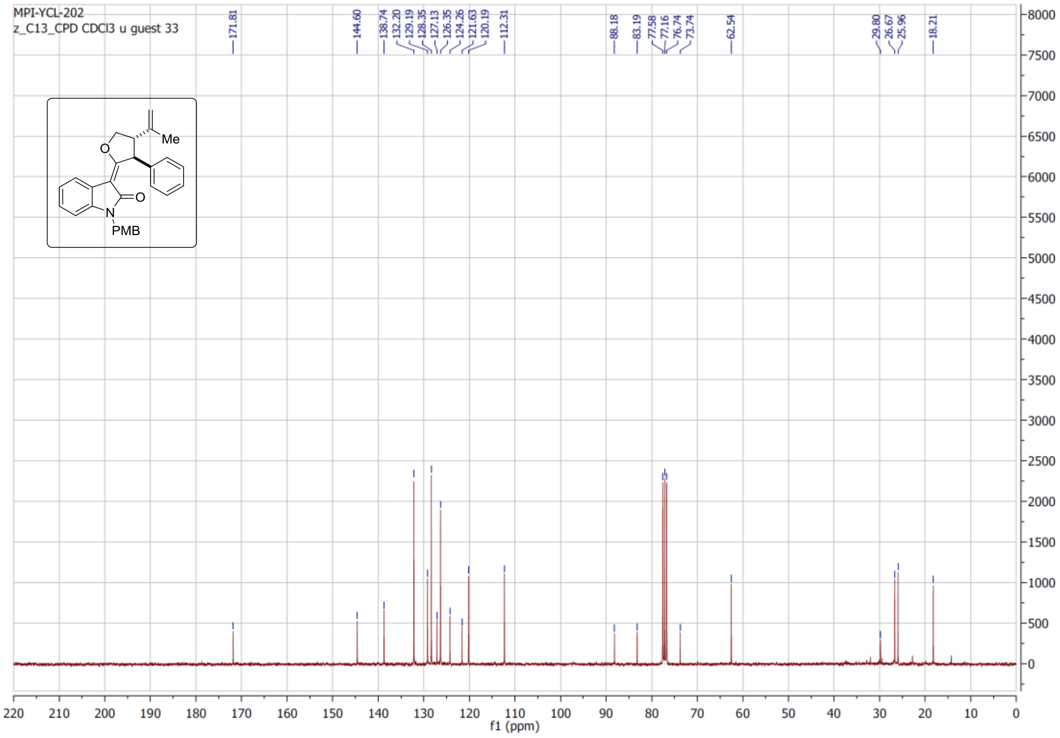

Supplementary Figure 126. ^{1}H and ^{13}C NMR spectra for 1**4r**.

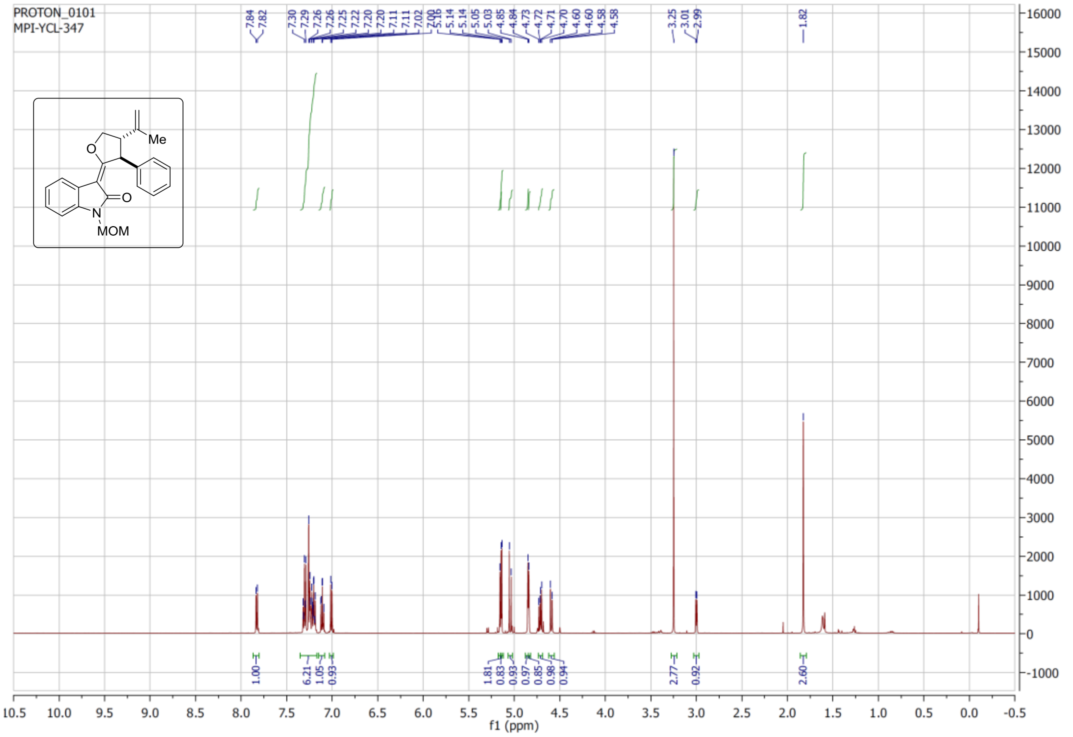

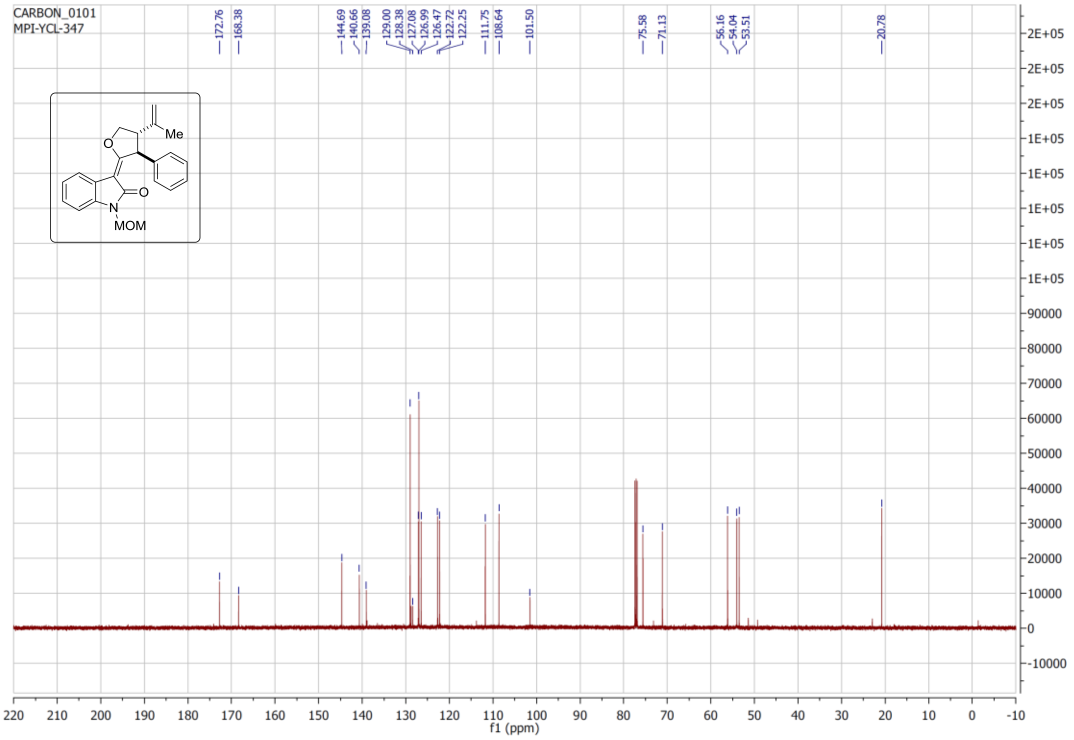

Supplementary Figure 127. ^{1}H and ^{13}C NMR spectra for 1**4s**.

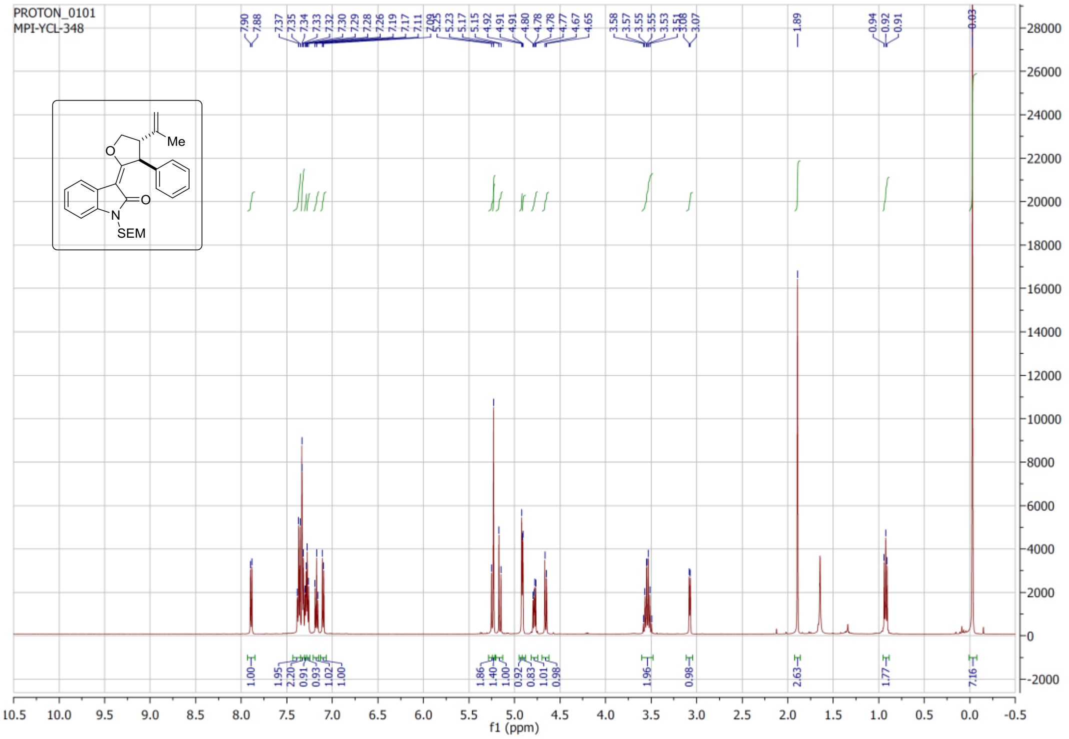

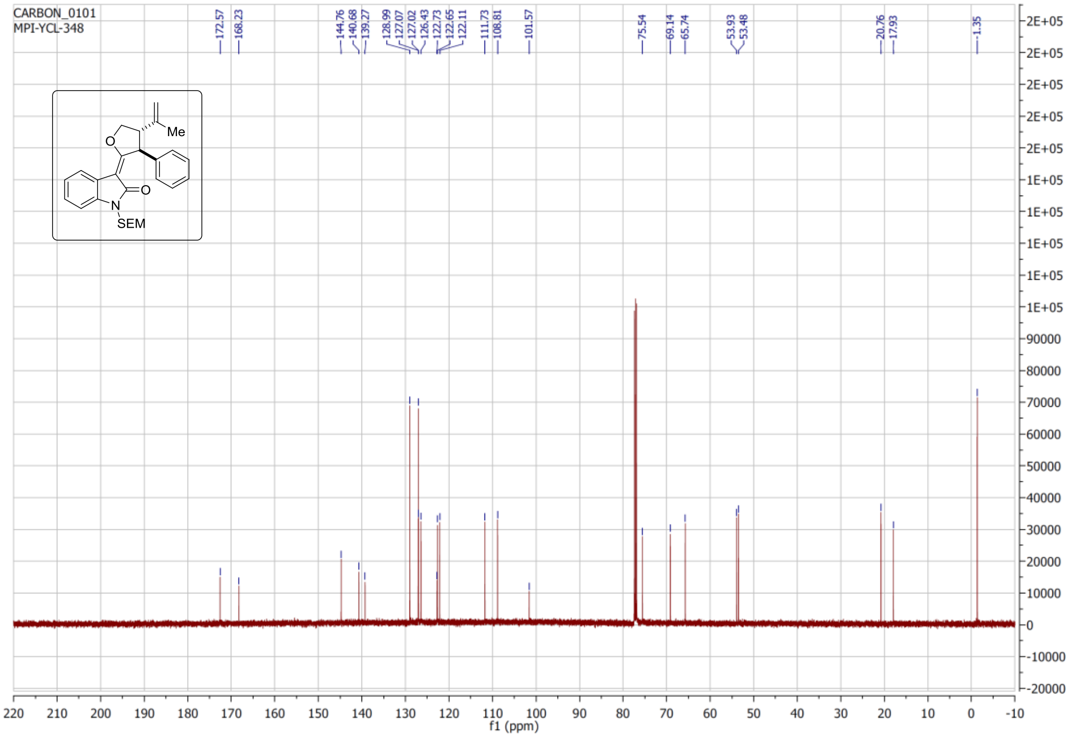

Supplementary Figure 128. ^{1}H and ^{13}C NMR spectra for 1**4t**.

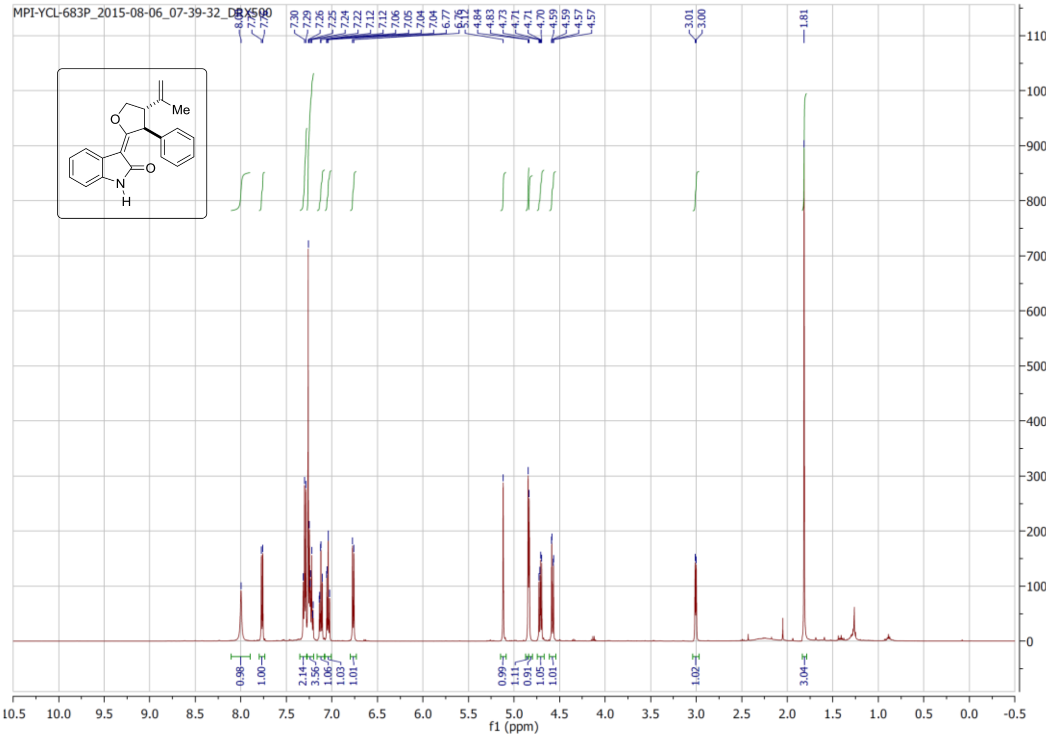

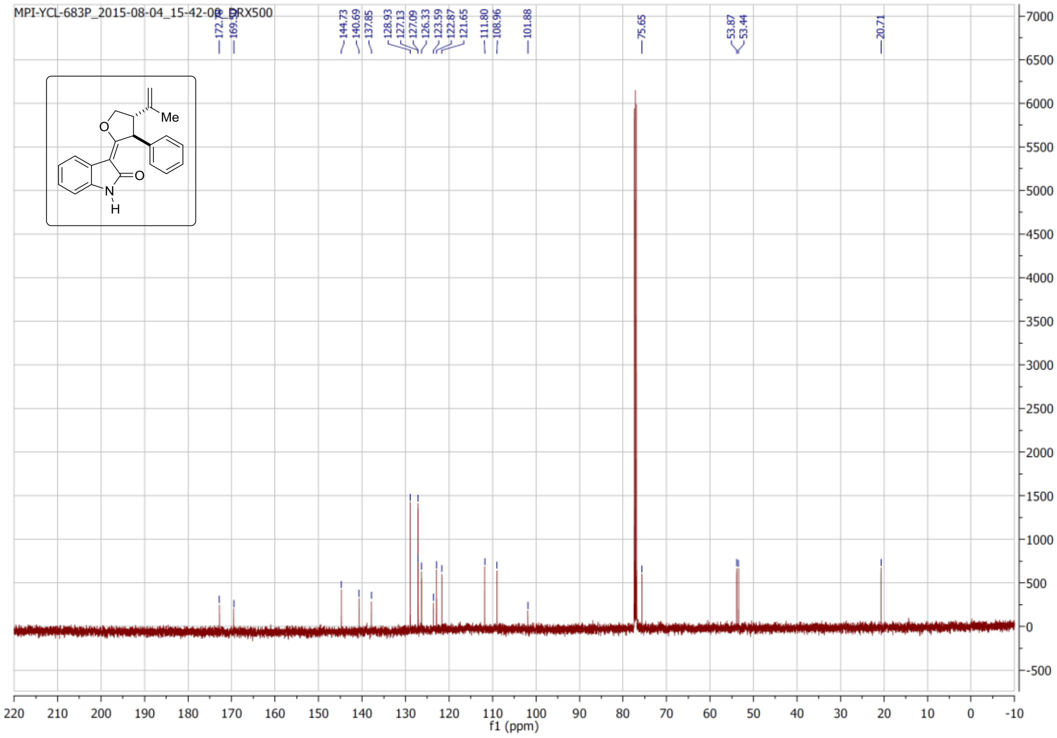

Supplementary Figure 129. ^{1}H and ^{13}C NMR spectra for 1**4u**.

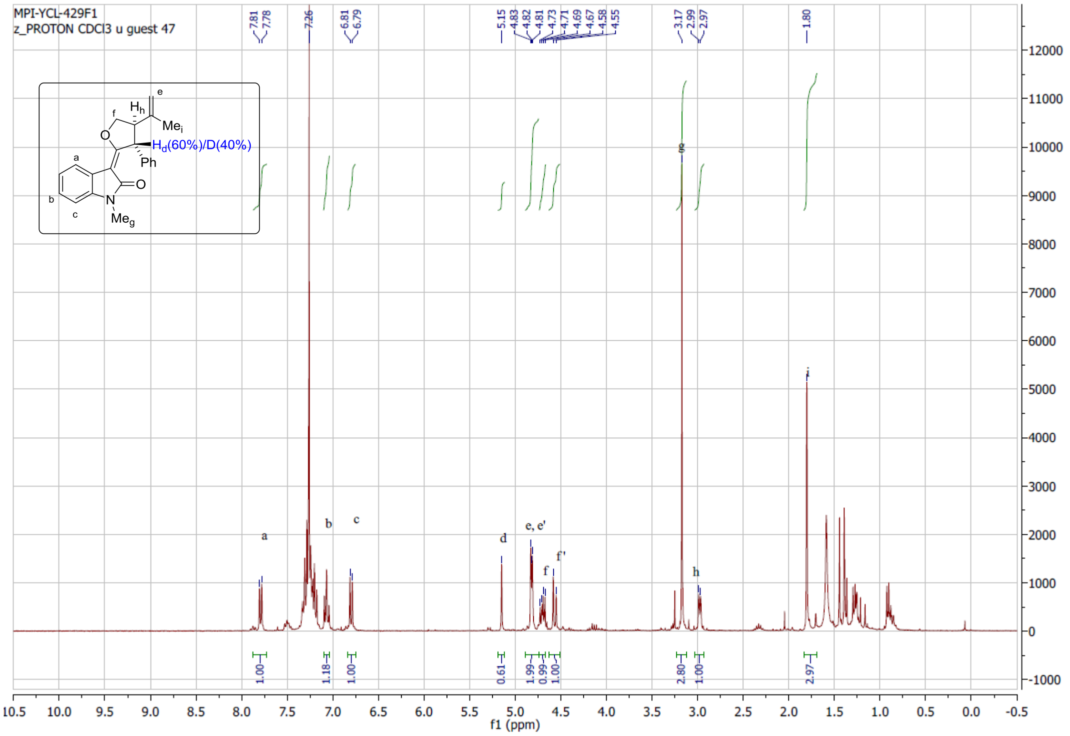

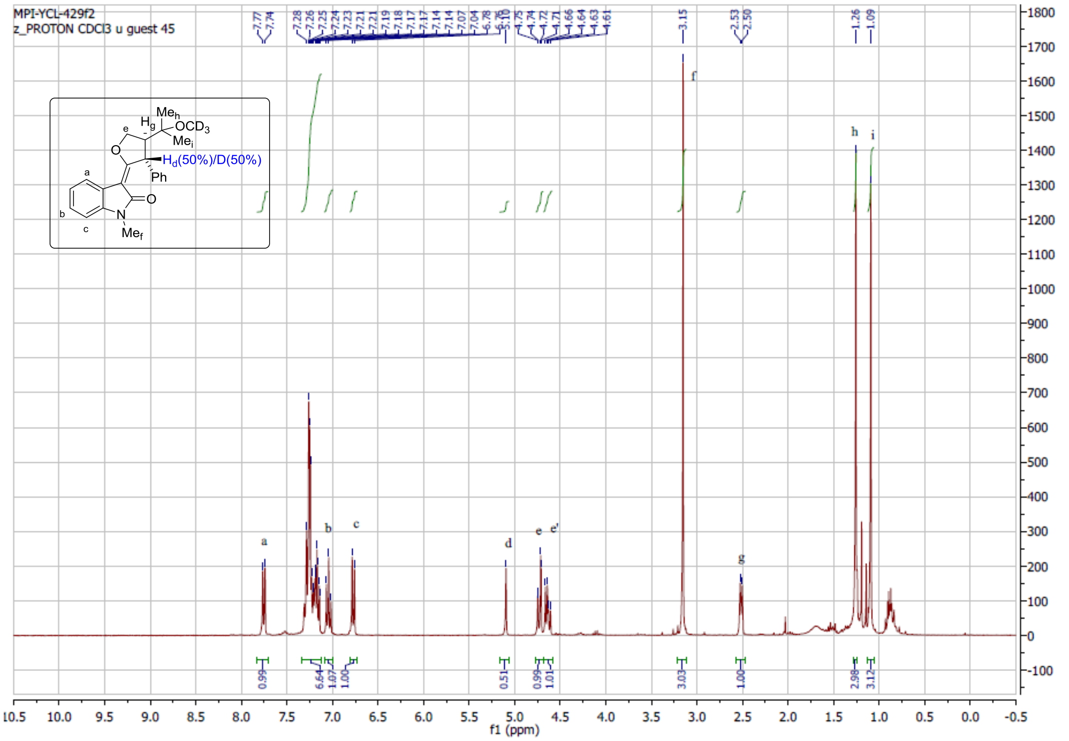

Supplementary Figure 130. ^{1}H NMR spectra for 1**4** and **20** with deuterated CD_3OD as nucleophile.

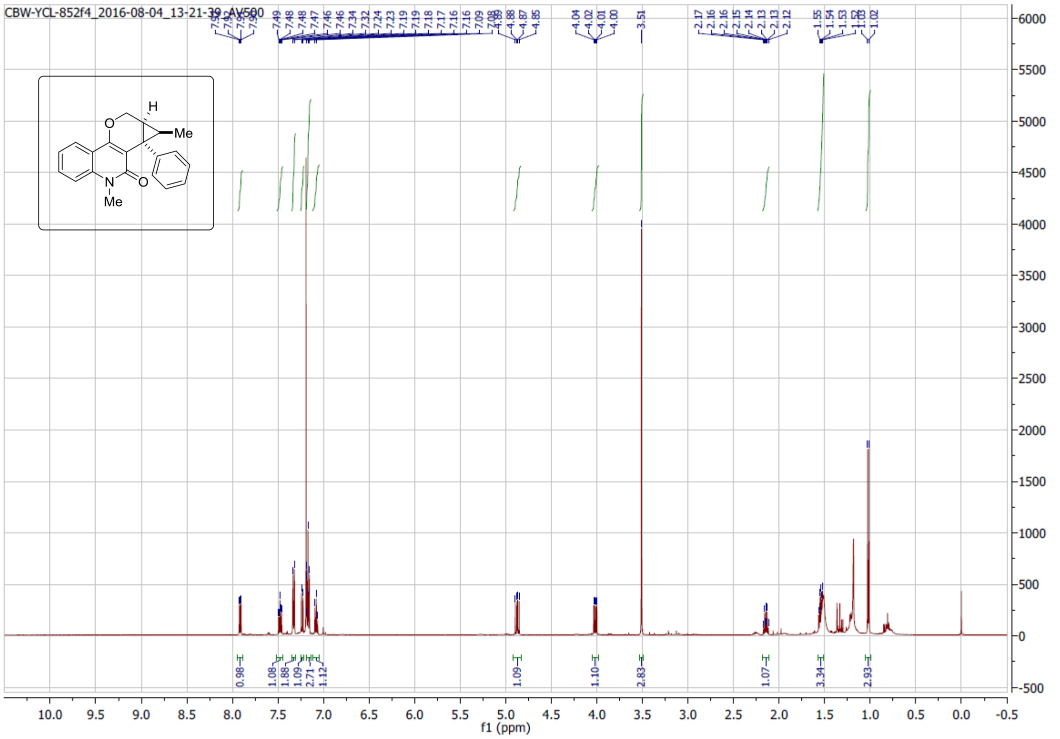

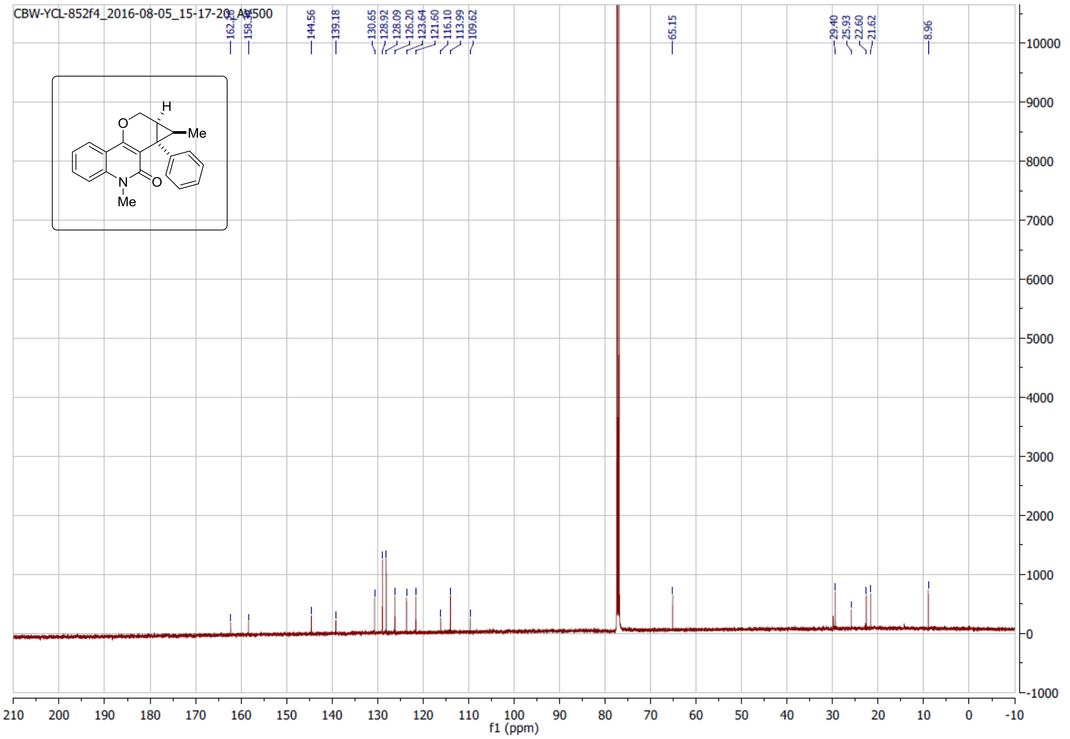

Supplementary Figure 131. ^{1}H and ^{13}C NMR spectra for **epi-3a**.

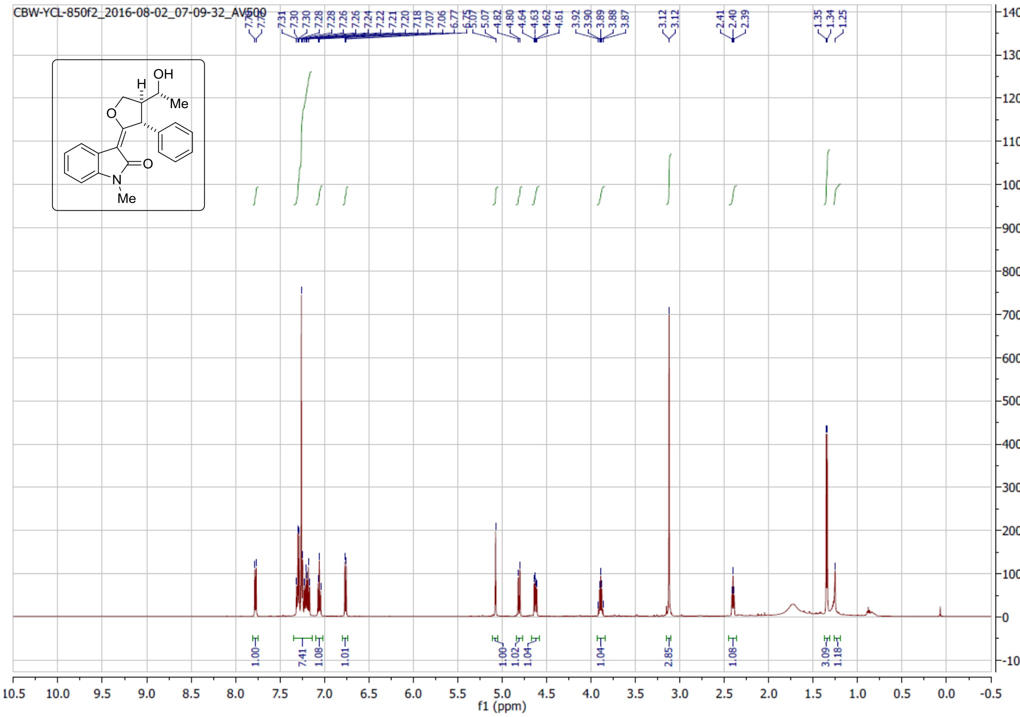

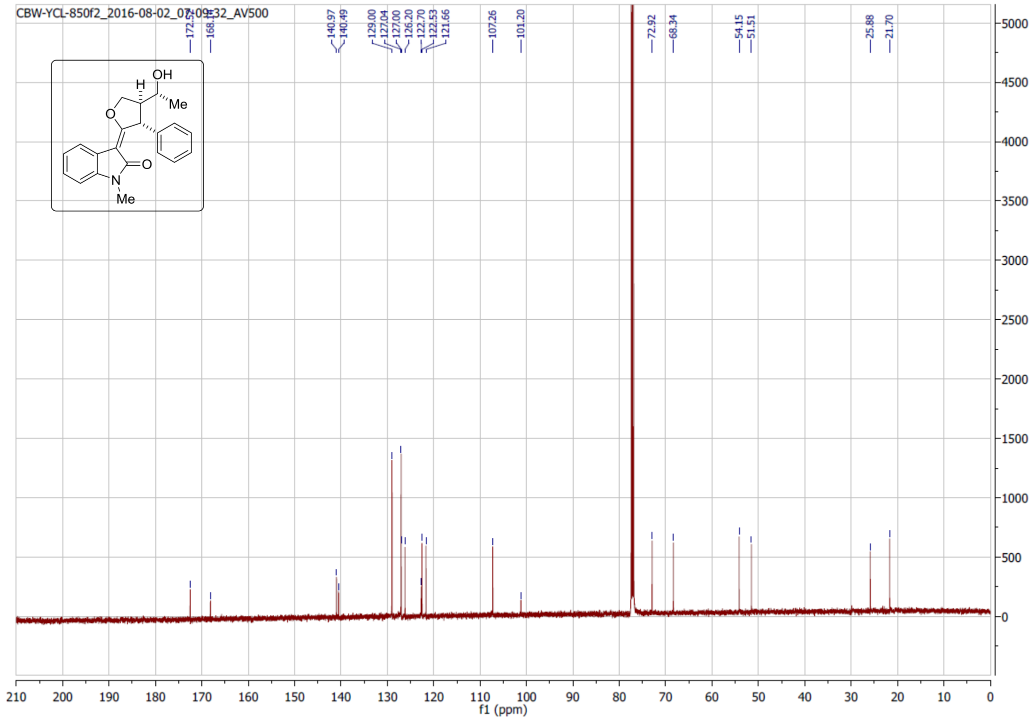

Supplementary Figure 132. ^{1}H and ^{13}C NMR spectra for **4OH**.

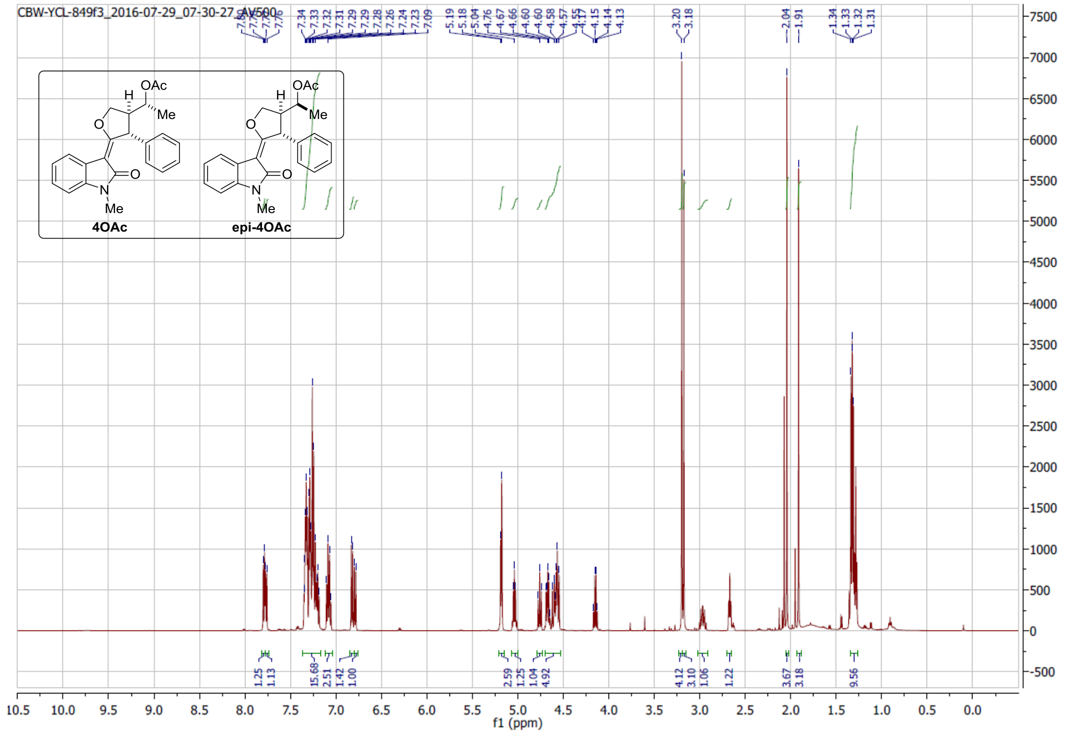

Supplementary Figure 133. ^{1}H NMR spectrum for **4OAc**.

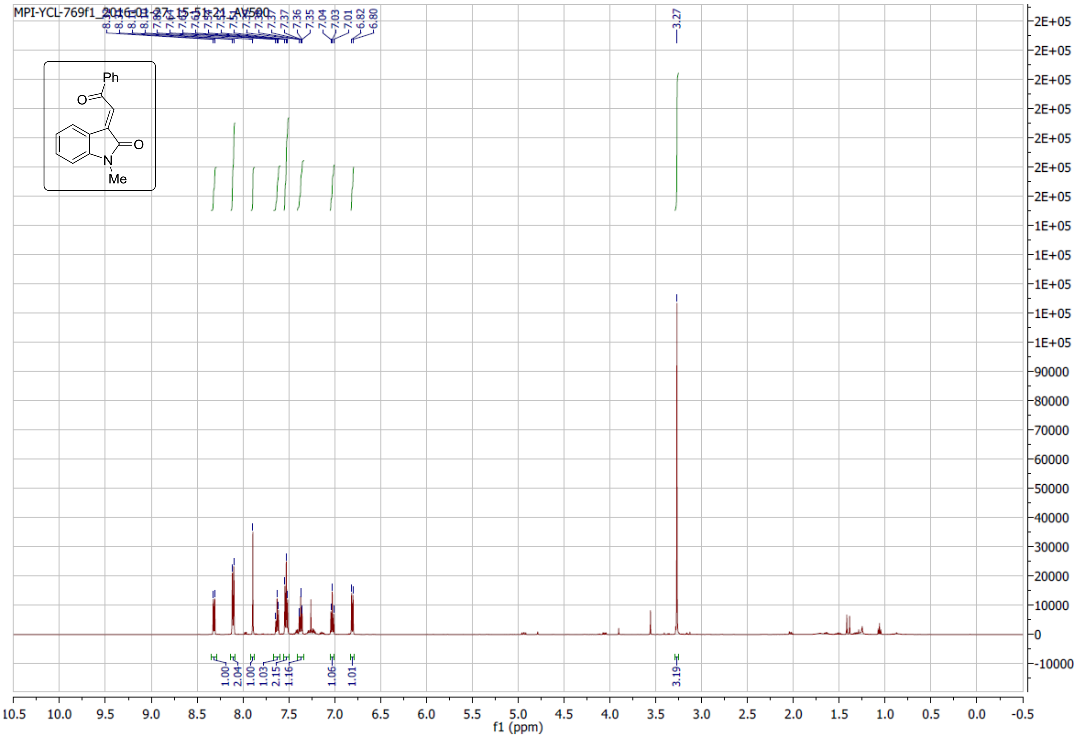

Supplementary Figure 134. ^{1}H NMR spectrum for **MS**.

Supplementary Table 1. Complete screening of 1,6-enyne cycloisomerization (**1a**)

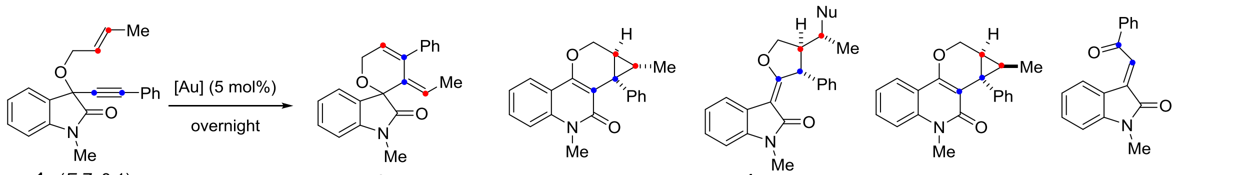

1a (*E*:*Z*=3:1) **2a** **3a** **4a** **epi-3a** **Meyer-Shuster rear. (MS)**

Entry	[Au]	mol%	Solvent	Nu (eq)	Temp.	Yield (%)					
						**2a**	**3a**	**4a**	**epi-3a**	**MS**	**1a (*Z*)**
1	$Au(BF_4)PPh_3$	5	DCM	-	rt	33	-	-	-	-	-
2	$AuCl_3$	5	DCM	-	rt	-	23	-	-	-	-
3	$AuCl_3$	20	DCM	-	rt	Messy					
4	**I**	5	DCM	-	rt	43	7	-	-	-	-
5	**II**	5	DCM	-	rt	-	57	-	-	-	-
**6**	**III**	**5**	**DCM**	**-**	**rt**	**-**	**67**	**-**	**20**	**-**	**-**
7	**III**	3	DCM	-	rt	-	27	-	-	-	-
8	**IV**	5	DCM	-	rt	-	43	-	-	-	-
**9**	**V**	**5**	**DCM**	**-**	**rt**	**60**	**-**	**-**	**-**	**-**	**10**
10	**V**	3	**DCM**	**-**	**rt**	58	-	-	-	-	
11	**III**	5	Toluene	-	rt	messy					
12	**III**	5	ACN	-	rt	no reaction					
13	**III**	5	DMF	-	rt	no reaction					
14	**III**	5	THF	-	rt	THF polymerized					
15	**V**	5	Toluene	-	rt	40	-	-	-	-	-
16	**V**	5	ACN	-	rt	no reaction					
17	**V**	5	DMF	-	rt	no reaction					
18	**V**	5	THF	-	rt	THF polymerized					
19	**II**	5	DCM	MeOH(20.0)	rt	no reaction					
20	**II**	5	DCE	MeOH(20.0)	60 °C	-	-	62	-	19	-
21	**III**	5	DCE	MeOH(20.0)	60 °C	-	-	56	-	-	-
22	**IV**	5	DCE	MeOH(20.0)	60 °C	-	-	50	-	-	-
23	**V**	5	DCE	MeOH(20.0)	60 °C	-	-	20	-	-	-
24	**II**	5	DCE	H_2O (20.0)	60 °C	-	30	23 (**4OH**)	15	-	-
25	**II**	5	DCE	AcOH (20.0)	60 °C	-	20	56 (**4OAc**)	13	-	-
26	**II**	5	DCE	Indole(2.0)	60 °C	-	37	-	-	23	-
27	**II**	**5**	**DCE**	**MeOH(10.0)**	**60 °C**	-	-	**73**	-	**18**	-
28	**II**	3	DCE	MeOH(10.0)	60 °C	-	-	64	-	-	-
29	**II**	5	DCE	MeOH(3.0)	60 °C	-	11	56	-	7	-
30	**II**	5	DCE	MeOH(1.0)	60 °C	-	32	43	-	-	-
31	**II**	5	DCE	MeOH(0.5)	60 °C	-	24	30	-	-	-

Supplementary Table 2. Complete screening of 1,6-enyne cycloisomerization (**13a**)

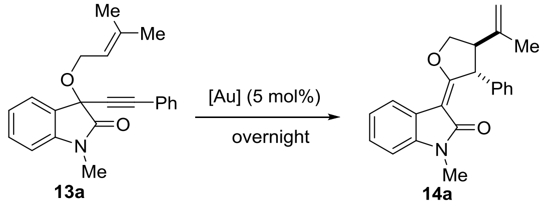

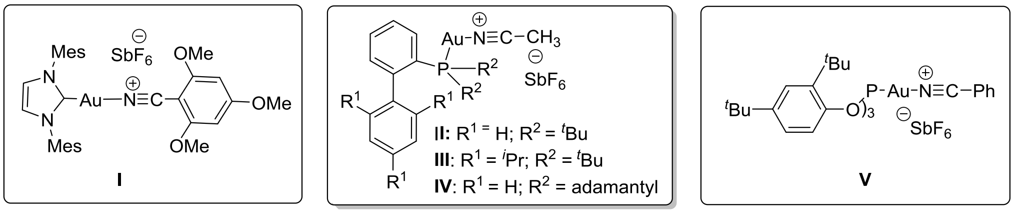

Entry	[Au] (5 mol%)	Conc. [M]	Solvent	Yield **14** (%)
1	Au(OTf)PPh_3	0.10	DCM	19
2	Au(BF_4)PPh_3	0.01	DCM	no reaction
3	$AuCl_3$	0.10	DCM	no reaction
4	**I**	0.10	DCM	no reaction
5	**I**	0.20	DCM	messy
6	**II**	0.10	DCM	50
7	**III**	0.10	DCM	49
8	**IV**	0.10	DCM	40
9	**V**	0.10	DCM	17
10	**II**	0.10	Toluene	40
11	**II**	0.10	Ether	38
**12**	**II**	**0.10**	**THF**	**95**
13	**II** (3 mol%)	0.10	THF	91
14	**II**	0.10	1,4-dioxane	59
15	**II**	0.10	$MeNO_2$	38
16	**II**	0.10	DMF	no reaction
17	**II**	0.10	ACN	no reaction

Supplementary Table 3. Crystal data and structure refinement for **2a**.

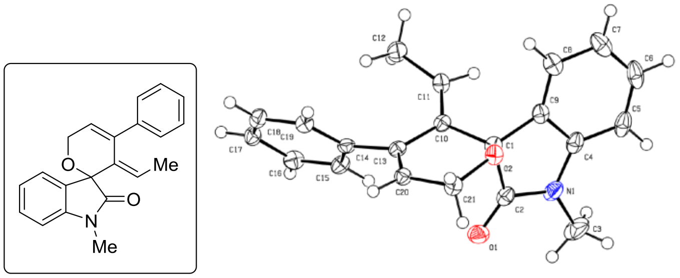

**Identification code**	CCDC# 1448677	
**Chemical formula**	$C_{21}H_{19}NO_2$	
**Formula weight**	317.37 g/mol	
**Temperature**	100(2) K	
**Wavelength**	1.54178 Å	
**Crystal size**	0.042 x 0.227 x 0.279 mm	
**Crystal system**	monoclinic	
**Space group**	P 1 21/c 1	
**Unit cell dimensions**	a = 12.8527(5) Å	α = 90°
	b = 9.9453(4) Å	β = 114.4410(10)°
	c = 13.9172(5) Å	γ = 90°
**Volume**	1619.54(11) $Å^3$	
**Z**	4	
**Density (calculated)**	1.302 g/cm^3	
**Absorption coefficient**	0.662 mm^{-1}	
**F(000)**	672	
**Diffractometer**	Bruker APEX-II CCD	
**Theta range for data collection**	3.78 to 65.31°	
**Index ranges**	-15<=h<=15, -11<=k<=11, -16<=l<=16	
**Reflections collected**	13387	
**Independent reflections**	2757 [R(int) = 0.0417]	
**Coverage of independent reflections**	99.5%	
**Absorption correction**	none	
**Max. and min. transmission**	0.9730 and 0.8370	
**Structure solution technique**	direct methods	
**Structure solution program**	SHELXS-97 (Sheldrick 2008)	
**Refinement method**	Full-matrix least-squares on F^2	

**Refinement program**	SHELXL-2014 (Sheldrick 2014)
**Function minimized**	$\Sigma\ w(F_o^2 - F_c^2)^2$
**Data / restraints / parameters**	2757 / 0 / 219
**Goodness-of-fit on F^2**	1.025
**Final R indices**	2323 data; I>2σ(I) R1 = 0.0481, wR2 = 0.1171
	all data R1 = 0.0585, wR2 = 0.1243
**Weighting scheme**	$w=1/[\sigma^2(F_o^2)+(0.0686P)^2+0.8411P]$ where $P=(F_o^2+2F_c^2)/3$
**Largest diff. peak and hole**	0.258 and -0.290 $eÅ^{-3}$
**R.M.S. deviation from mean**	0.055 $eÅ^{-3}$

Supplementary Table 4. Crystal data and structure refinement for **3a**.

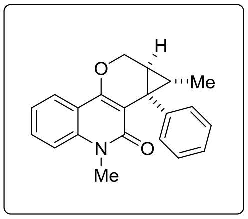

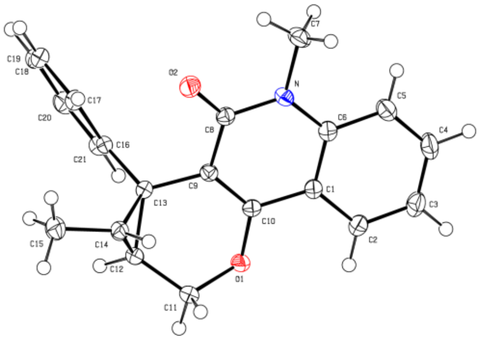

**Identification code**	CCDC# 1448646
**Empirical formula**	$C_{21}H_{19}NO_2$
**Formula weight**	317.37
**Temperature/K**	173(2)
**Crystal system**	monoclinic
**Space group**	$P2_1/n$
**a/Å**	13.3456(6)
**b/Å**	7.5048(3)
**c/Å**	16.0725(7)
**α/°**	90
**β/°**	99.871(4)
**γ/°**	90
**Volume/Å^3**	1585.93(11)
**Z**	4
**ρ_{calc}g/cm^3**	1.329
**μ/mm^{-1}**	0.085
**F(000)**	672.0
**Crystal size/mm^3**	? × ? × ?
**Radiation**	MoKα (λ = 0.71073)
**2Θ range for data collection/°**	5.146 to 58.228
**Index ranges**	$-16 \le h \le 18$, $-10 \le k \le 10$, $-21 \le l \le 21$
**Reflections collected**	21487
**Independent reflections**	3879 [R_{int} = 0.0357, R_{sigma} = 0.0290]
**Data/restraints/parameters**	3879/0/219
**Goodness-of-fit on F^2**	1.026

**Final R indexes [I>=2σ (I)]**	R_1 = 0.0444, wR_2 = 0.1023
**Final R indexes [all data]**	R_1 = 0.0576, wR_2 = 0.1093
**Largest diff. peak/hole / e Å^{-3}**	0.28/-0.25

Supplementary Table 5. Crystal data and structure refinement for 4a.

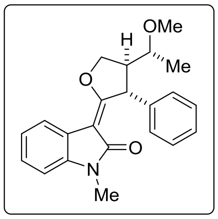

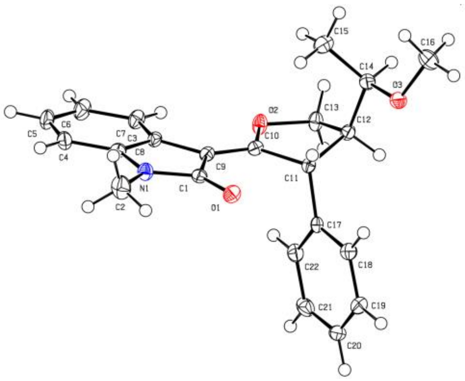

**Identification code**	CCDC# 1448652
**Empirical formula**	$C_{22}H_{23}NO_3$
**Formula weight**	349.41
**Temperature/K**	100.03
**Crystal system**	monoclinic
**Space group**	$P2_1/c$
**a/Å**	14.9063(10)
**b/Å**	7.2965(4)
**c/Å**	17.0837(11)
**α/°**	90
**β/°**	102.332(2)
**γ/°**	90
**Volume/Å^3**	1815.2(2)
**Z**	4
**ρ_{calc}g/cm^3**	1.279
**μ/mm^{-1}**	0.085
**F(000)**	744.0
**Crystal size/mm^3**	0.603 × 0.388 × 0.378
**Radiation**	MoKα (λ = 0.71073)
**2Θ range for data collection/°**	4.882 to 55.796
**Index ranges**	$-19 \le h \le 19$, $-9 \le k \le 9$, $-22 \le l \le 22$
**Reflections collected**	35891
**Independent reflections**	4339 [R_{int} = 0.0490, R_{sigma} = 0.0314]
**Data/restraints/parameters**	4339/0/238

**Goodness-of-fit on F^2**	1.076
**Final R indexes [I>=2σ (I)]**	R_1 = 0.0466, wR_2 = 0.1015
**Final R indexes [all data]**	R_1 = 0.0646, wR_2 = 0.1102
**Largest diff. peak/hole / e $Å^{-3}$**	0.35/-0.27

Supplementary Table 6. Crystal data and structure refinement for **14a**.

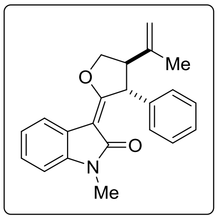

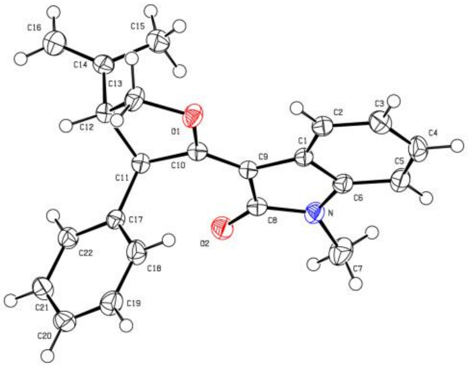

**Identification code**	CCDC# 1448645
**Empirical formula**	$C_{22}H_{21}NO_2$
**Formula weight**	331.40
**Temperature/K**	173(2)
**Crystal system**	monoclinic
**Space group**	$P2_1/c$
**a/Å**	10.5511(7)
**b/Å**	9.3096(5)
**c/Å**	17.9883(10)
**α/°**	90
**β/°**	90.151(6)
**γ/°**	90
**Volume/Å^3**	1766.93(19)
**Z**	4
**ρ_{calc}g/cm^3**	1.246
**μ/mm^{-1}**	0.079
**F(000)**	704.0
**Crystal size/mm^3**	0.3 × 0.25 × 0.15
**Radiation**	MoKα (λ = 0.71073)
**2Θ range for data collection/°**	4.528 to 53.996
**Index ranges**	$-13 \leq h \leq 12$, $-11 \leq k \leq 11$, $-22 \leq l \leq 22$
**Reflections collected**	16629
**Independent reflections**	3819 [R_{int} = 0.0392, R_{sigma} = 0.0338]
**Data/restraints/parameters**	3819/0/228

**Goodness-of-fit on F^2**	1.036
**Final R indexes [I>=2σ (I)]**	R_1 = 0.0436, wR_2 = 0.0994
**Final R indexes [all data]**	R_1 = 0.0599, wR_2 = 0.1088
**Largest diff. peak/hole / e Å^{-3}**	0.21/-0.24

Supplementary Table 7. Summary of primary screening data for Hh, Wnt and Autophagy inhibition. Data are mean values of three independent experiments (n = 3) ± s.d.. n.a.: No activity.

Compound	Inhibition of Hh signaling IC50 [µM]	Inhibition of Wnt signaling IC50 [µM]	Inhibition of Autophagy IC50 [µM]
**2a**	n.a	n.a	n.a
**2b**	n.a	n.a	n.a
**2c**	n.a	n.a	n.a
**2d**	n.a	n.a	n.a
**2e**	n.a	n.a	n.a
**2f**	n.a	n.a	n.a
**2g**	n.a	n.a	n.a
**2h**	n.a	n.a	n.a
**2i**	n.a	n.a	n.a
**2j**	n.a	n.a	n.a
**2k**	n.a	n.a	n.a
**2l**	n.a	n.a	n.a
**3a**	n.a	12 (1.32)	n.a
**3b**	n.a	n.a	5.61 (0.25)
**3c**	n.a	n.a	n.a
**3d**	n.a	17 (4.03)	n.a
**3e**	n.a	n.a	n.a
**3f**	n.a	n.a	4.81 (0.25)
**3g**	n.a	n.a	n.a
**3h**	n.a	8.6 (2.32)	n.a
**3i**	9.6 (2.98)	n.a	n.a
**3j**	n.a	4.2 (1.35)	n.a
**4a**	n.a	n.a	n.a
**4b**	n.a	n.a	n.a
**4c**	n.a	n.a	n.a
**4d**	6.8 (1.89)	n.a	n.a
**4e**	n.a	n.a	n.a
**4f**	n.a	n.a	8.33 (0.11)
**4g**	6.26 (2.56)	n.a	n.a
**4h**	n.a	n.a	n.a
**4i**	n.a	n.a	6.88 (0.36)
**4j**	n.a	n.a	n.a

**4k**	n.a	n.a	n.a
**4l**	n.a	n.a	n.a
**4m**	n.a	n.a	n.a
**14a**	5.26 (1.25)	n.a	8.92 (0.74)
**14b**	n.a	n.a	n.a
**14c**	n.a	n.a	n.a
**14d**	n.a	n.a	n.a
**14e**	6.96 (2.66)	17.03 (6.22)	n.a
**14f**	n.a	n.a	n.a
**14g**	n.a	n.a	n.a
**14h**	n.a	n.a	n.a
**14i**	n.a	n.a	n.a
**14j**	n.a	n.a	n.a
**14k**	n.a	n.a	n.a
**14l**	n.a	n.a	n.a
**14m**	6.53 (0.56)	13.65 (3.89)	6.71 (0.79)
**14n**	n.a	n.a	n.a
**14o**	n.a	n.a	n.a
**14p**	n.a	n.a	6.47 (0.60)
**14q**	2.75 (0.62)	n.a	n.a
**14r**	3.13 (1.02)	n.a	n.a
**14s**	9.56 (3.32)	n.a	n.a
**14t**	6.33 (0.65)	n.a	n.a
**14u**	n.a	n.a	n.a

Supplementary Table 8. Reactivity of the compounds towards GSH.

Compounds (30 µM) were incubated with 5 µM GSH or PBS. Reactivity of the compounds towards GSH was measured by HPLC. Numbers are percent abundance of the compounds in GSH as compared to their abundance in PBS. Data are mean values of three independent experiments (n = 3) ± s.d

**Compound**	**1 h**	**24 h**	**48 h**
**2d**	102.0 (4.6)	95.8 (6.9)	89.1 (8.3)
**3f**	90.9 (3.9)	80.5 (5.1)	76.7 (2.8)
**3j**	100.6 (1.5)	96.5 (5.2)	94.0 (2.3)
**4i**	104.4 (8.2)	89.7 (6.2)	87.9 (5.9)
**14q**	99.9 (2.9)	96.8 (3.7)	90.3 (1.5)
**14r**	98.3 (4.6)	95.1 (3.6)	92.2 (3.6)

Supplementary Methods

General information

All commercially obtained chemicals and reagents were used without further purification. Dry dichloromethane, THF, DMF were used the Solvent Purification System M-BRAUN Glovebox Technology SPS-800. All reactions were performed in flame dried glassware with dry solvent under argon atmosphere. Analytical thin-layer chromatography (TLC) was performed on Merck silica gel aluminium plates with F-254 indicator. Compounds were visualized by irradiation with UV light. Column chromatography was performed by using silica gel Merck 60 (particle size 0.040-0.063 mm).

^{1}H-NMR and ^{13}C-NMR were recorded on a *Bruker DPX300* (300 MHz), *Bruker DRX400* (400 MHz), *Bruker DRX500* (500MHz), *Bruker DRX600* (600 MHz) and *Varian INOVA500* (500 MHz) at 300 K using $CDCl_3$ or *d*-DMSO as solvent. Chemical shifts of spectra were expressed in parts per million (ppm, δ) and calibrated relative to residual proton and carbon signals in deuterated NMR solvent ($CDCl_3$: δ = 7.26 ppm for ^{1}H-NMR and δ = 77.16 ppm for ^{13}C-NMR. *d*-DMSO: δ = 2.50 ppm for ^{1}H-NMR and δ = 39.52 ppm for ^{13}C-NMR). Multiplicities are indicated as following: (s = singlet, d = doublet, t = triplet, q = quartet, m = multiplet), and coupling constants (J) are represented in Hertz (Hz). High resolution mass spectra (HRMS) were recorded on a LTQ Orbitrap mass spectrometer coupled to an Acceka HPLC-System (HPLC column: Hypersyl GOLD, 50 mm x 1 mm, particle size 1.9 μm, ionization method: electron spray ionization).

The substrates of 1-methylisatin [2058-74-4] and 1-benzyl-1*H*-indole-2,3-dione [1217-89-6] were directly purchased from Sigma-Aldrich for later studies. The substrate of 1,6-enynes with the substituent of (*E*)-crotyl group were prepared from crotyl bromide [29576-14-5] with a small amount of inseparable (*Z*)-isomer. Inseparable (*Z*)-isomers were proceed through the 1,6-enyne synthesizes and following gold catalyzed cycloisomerization reactions. The ratio of (*E*,*Z*)-isomers in 1,6-enynes were determined by ^{1}H-NMR, and the yield of gold catalyzed cycloisomerizations was calculated on the basis of (*E*,*Z*)-mixture. For NMR spectra of the compounds in this article, see Supplementary Figures 4-129.

The known gold complexes (**I**[1], **II** [866641-66-9], **III** [1140531-94-7], **IV**[2] and **V**[2,3]) were either purchased from Sigma-Aldrich or synthesized as following :

Synthesis of gold(l) catalysts (I)

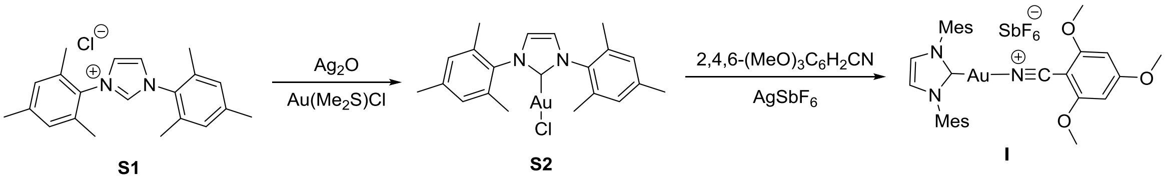

To a solution of the 1,3-dimesitylimidazolium chloride **S1** (1 mmol) in DCM was added Ag_2O (0.5 mmol). The suspension became clear after stirring for 3 h at 23 ^{o}C. A solution of [Au(Me_2S)Cl] (1 mmol) in DCM was added dropwise, the reaction mixture was stirred for another 4 h, the solution was filtered through Celite, and the solvent was partially evaporated. Addition of hexane resulted in the precipitation of the gold(l) complex (**S2**). A solution of **S2** (0.10 mmol) and 2,4,6-trimethoxybenzonitrile (0.10 mmol) in DCM was added over solid $AgSbF_6$ (0.10 mmol) and stirred for 5 min. The mixture was filtered (HPLC Teflon filter) and the solid residue washed with DCM twice. The gold complex precipitated from the filtrate upon addition of Et_2O. Filtration and air-drying furnished a bright white solid (**I**).

Synthesis of gold(l) catalysts (II, III, IV)

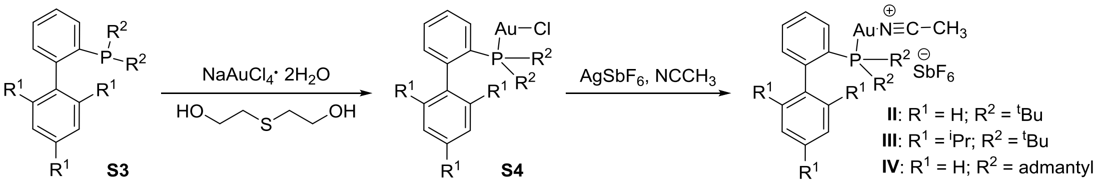

At 0 ^{o}C, to the orange solution of sodium tetrachloroaurate dihydrate (1 mmol) in water was slowly added 2,2'-thiodiethanol (3 mmol) with 45 min stirring. The corresponded phosphine ligand (1 mmol) was added to the mixture and a white precipitate was formed during the process. After stirring for 20 min, the solid was filtered off, washed with MeOH, and dried in vacuo to provide the Au(L)Cl **(S4)** .

At 0 ^{o}C, to a solution of **S4** (0.5 mmol) in DCM was added MeCN (1 mmol), followed by the addition of $AgSbF_6$ (0.5 mmol). The reaction mixture was stirred at room temperature overnight,

the crude product was filtered through a pad of Celite®, and the solvent was again filtered through a syringe filter to give a clear solution. After the evaporation of solvent, white solids were obtained as the desired cationic gold(l) complexes **(II**, **III**, or **IV)**.

Synthesis of gold(l) catalysts (V)

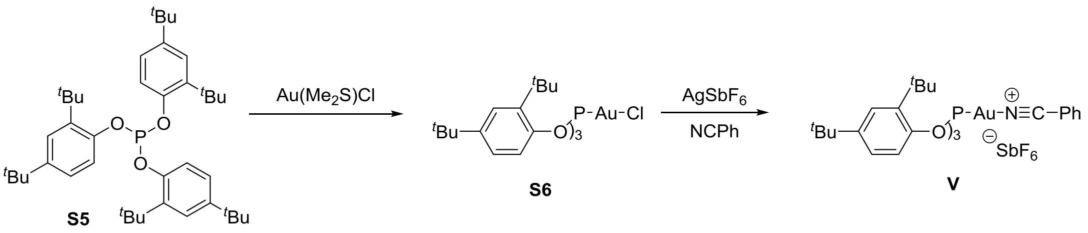

At 0 ^{o}C, to a solution of Au(Me_2S)Cl (1 mmol) in DCM was slowly added a solution of tris(2,4-di-tert-butylphenyl) phosphite (1.05 mmol) in DCM and the resulting reaction mixture was allowed to warm to room temperature. The reaction was monitored by the consumption of the ligand through TLC. After the reaction was complete, the mixture was filtered through a syringe filter and concentrated to provide the desired Au(l)Cl complex (**S6**).

At 0 ^{o}C, to a solution of **S6** (1.00 mmol) and PhCN (1.1 mmol) in DCM was added $AgSbF_6$ (1.00 mmol). A white precipitate appeared immediately and the reaction mixture was further stirred at room temperature overnight. The resulting reaction mixture was filtered through a pad of Celite®, and the solvent was again filtered through a syringe filter to give a clear solution. After the evaporation of solvent and drying *in vacuo*, white solids were obtained as the desired cationic gold(l) complexes **(V)**.

Preparation of starting materials

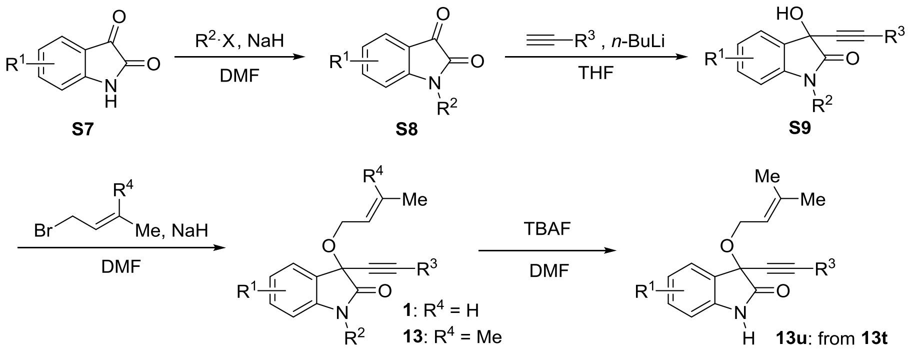

General procedure A

To a solution of a isatin analog (10 mmol) in DMF (30 ml) at 0 °C was added NaH 60% wt (12 mmol) in one portion and the mixture was stirred at the same temperature for 1 h. To the resulting mixture was added dropwise the respective alkyl halide (13 mmol). The mixture was warmed to room temperature and stirred overnight. The reaction was quenched with $NH_4Cl_{(sat)}$ and diluted with EtOAc (100 ml). After extraction, the organic layer was washed with H_2O (300 ml) three times and once with brine, dried over $MgSO_{4(s)}$, filtered, and concentrated under reduced pressure to provide the crude product. The product was purified by flash column chromatography (petroleum ether (PE) / EtOAc (EA) mixture as eluent) to afford the desired product.

General procedure B

At -78 °C, to a solution of acetylene (2.4 mmol) in THF (30 ml) was slowly added 2.5 M *n*-BuLi in hexanes (2.3 mmol) and the mixture was stirred for 1 h at the same temperature. The *N*-protected isatin analog (2 mmol) was then added to the rection mixture in one portion. Afterwards, the resulting mixture was slowly warmed to room temperature and stirred overnight. The reaction was quenched with $NH_4Cl_{(sat)}$ and extracted with EtOAc (30 ml) three times. The combined organic layers were washed with brine and dried over $MgSO_{4(s)}$. After concentration under reduced pressure, the crude product was purified by flash column chromatography (EtOAc / petroleum ether or DCM / EtOAc mixture as eluent) to afford the desired product.

General procedure C

To a solution of the propargyl alcohol (0.5 mmol) in DMF (5 ml) at 0 °C was added NaH 60% wt (0.55 mmol) in one portion and the mixture was stirred at same temperature for 1 h. To the resulting mixture was added dropwise the respective allylic halide (0.6 mmol). The mixture was warmed to room temperature and stirred overnight. The reaction was quenched with $NH_4Cl_{(sat)}$ and the mixture was diluted with EtOAc (50 ml). After extraction, the organic layer was washed with H_2O (50 ml) three times and once with brine, dried over $MgSO_{4(s)}$, filtered, and concentrated under reduced pressure to provide the crude product. The product was purified by flash column chromatography (petroleum ether / EtOAc mixture as eluent) to afford the desired product.

General procedure D

Tetrabutylammonium fluoride (1.0 M in THF, 15 µl, 0.015 mmol) was added to a solution of **1as** (22 mg, 0.05 mmol) in DMF (2 ml). After 1 h stirring at 100 °C, the reaction mixture was cooled, and then diluted with water and ethyl acetate. The aqueous phase was extracted twice with ethyl acetate. The combined organic phases were washed with 1N HCl and saturated aqueous $NaHCO_3$ (2 mL), dried over $MgSO_{4(s)}$ and concentrated to give the crude product, which was further purified by silica gel column chromatography to afford the desired product.

1,5-dimethylindoline-2,3-dione (S8a) was prepared according to the general procedure A, by using 5-methylindoline-2,3-dione (150 mg, 0.93 mmol) and iodomethane. After silica gel column chromatography with EtOAc / petroleum ether = 1/2 (R_f = 0.30) as eluents, the desired product was obtained in 89% yield (145 mg, 0.83 mmol) as a red solid. The analytical data were identical to literature data.[4] **^{1}H NMR** (500 MHz, $CDCl_3$) δ 7.41 (s, 1H), 7.40 (d, *J* = 8.0 Hz, 1H), 6.78 (d, *J* = 8.0 Hz, 1H), 3.23 (s, 3H), 2.34 (s, 3H).

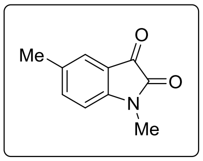

5-methoxy-1-methylindoline-2,3-dione (S8b) was prepared according to the general procedure

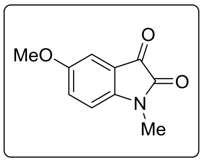

A, by using 5-methoxyindoline-2,3-dione (1074 mg, 6.06 mmol) and iodomethane. After silica gel column chromatography with EtOAc / petroleum ether = 1/2 (R_f = 0.23) as eluents the desired product was obtained in 89% yield (1026 mg, 5.37 mmol) as a black solid. The analytical data were identical to literature data.[5] **^{1}H NMR** (400 MHz, $CDCl_3$) δ 7.13 (dd, *J* = 8.5, 2.7 Hz, 1H), 7.09 (d, *J* = 2.7 Hz, 1H), 6.79 (d, *J* = 8.5 Hz, 1H), 3.78 (s, 3H), 3.19 (s, 3H).

6-bromo-1-methylindoline-2,3-dione (S8c) was prepared according to the general procedure A,

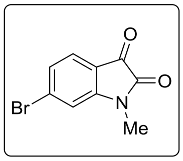

by using 6-bromoindoline-2,3-dione (1370 mg, 6.06 mmol) and iodomethane. After silica gel column chromatography with EtOAc / petroleum ether = 1/2 (R_f = 0.65) as eluents the desired product was obtained in 80% yield (1162 mg, 4.84 mmol) as an orange solid. The analytical data were identical to literature data.[5] **^{1}H NMR** (400 MHz, $CDCl_3$) δ 7.47 (d, *J* = 8.0 Hz, 1H), 7.30 (dd, *J* = 8.0, 1.5 Hz, 1H), 7.10 (d, *J* = 1.5 Hz, 1H), 3.26 (s, 3H).

5-fluoro-1-methylindoline-2,3-dione (S8d) was prepared according to the general procedure A,

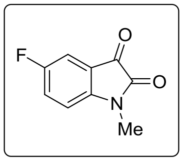

by using 5-fluoroindoline-2,3-dione (1001 mg, 6.06 mmol) and iodomethane. After silica gel column chromatography with EtOAc / petroleum ether = 1/2 (R_f = 0.3) as eluents the desired product was obtained in 87% yield (945 mg, 5.27 mmol) as a dark red solid. The analytical data were identical to literature data.[4] **^{1}H NMR** (400 MHz, $CDCl_3$) δ 7.39 – 7.25 (m, 2H), 6.89 (dd, *J* = 8.5, 3.6 Hz, 1H), 3.26 (s, 3H).

1-methyl-5-(trifluoromethoxy)indoline-2,3-dione (**S8e**) was prepared according to the general

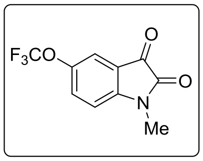

procedure A, by using 5-(trifluoromethoxy)indoline-2,3-dione (420 mg, 1.82 mmol) and iodomethane. After silica gel column chromatography with EtOAc / petroleum ether = 1/2 (R_f = 0.35) as eluents the desired product was obtained in 94% yield (418 mg, 1.71 mmol) as a red solid. **^{1}H NMR** (400 MHz, $CDCl_3$) δ 7.56 – 7.38 (m, 2H), 6.95 (d, *J* = 8.3 Hz, 1H), 3.26 (s, 3H). **^{13}C NMR** (101 MHz, $CDCl_3$) δ 182.5, 158.0, 149.9,

145.4, 145.4, 131.3, 120.5 (q, J = 258.2 Hz), 118.5, 118.0, 111.2, 26.5. **HRMS** (ESI): Calcd for $(M + H)^+$ $[C_{10}H_7O_3NF_3]^+$: 246.0373, found: 246.0379.

1-methyl-5-nitroindoline-2,3-dione (S8f) was prepared according to the general procedure A,

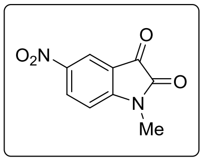

by using 5-nitroindoline-2,3-dione (800 mg, 4.16 mmol) and iodomethane. After silica gel column chromatography with EtOAc / petroleum ether = 1/2 (R_f = 0.13) as eluents the desired product was obtained in 99% yield (849 mg, 4.12 mmol) as a brown solid. The analytical data were identical to literature data.[6] **^{1}H NMR** (500 MHz, $CDCl_3$) δ 8.56 (dd, J = 8.7, 2.3 Hz, 1H), 8.48 (d, J = 2.3 Hz, 1H), 7.05 (d, J = 8.7 Hz, 1H), 3.36 (s, 3H).

5-chloro-1-methylindoline-2,3-dione (S8g) was prepared according to the general procedure A,

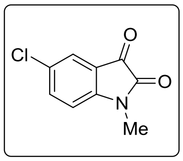

by using 5-chloroindoline-2,3-dione (1100 mg, 6.06 mmol) and iodomethane. After silica gel column chromatography with EtOAc / petroleum ether = 1/2 (R_f = 0.33) as eluents the desired product was obtained in 96% yield (1142 mg, 5.84 mmol) as a red solid. The analytical data were identical to literature data.[4] **^{1}H NMR** (400 MHz, $CDCl_3$) δ 7.55 (dd, J = 8.3, 2.2 Hz, 1H), 7.52 (d, J = 2.2 Hz, 1H), 6.86 (d, J = 8.3 Hz, 1H), 3.23 (s, 3H).

1-(4-methoxybenzyl)indoline-2,3-dione (S8h) was prepared according to the general procedure

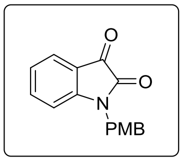

A, by using indoline-2,3-dione (2.000 g, 13.59 mmol) and 4-methoxybenzyl chloride. After silica gel column chromatography with EtOAc / petroleum ether = 1/2 (R_f = 0.53) as eluents the desired product was obtained in 95% yield (3.452 g, 12.91 mmol) as an orange solid. The analytical data were identical to literature data.[7] **^{1}H NMR** (400 MHz, $CDCl_3$) δ 7.60 (dd, J = 7.6, 0.8 Hz, 1H), 7.48 (td, J = 7.6, 1.3 Hz, 1H), 7.31-7.22 (m, 2H), 7.08 (t, J = 7.6 Hz, 1H), 6.89 – 6.85 (m, 2H), 6.80 (d, J = 8.0 Hz, 1H), 4.87 (s, 2H), 3.79 (s, 3H).

1-(methoxymethyl)indoline-2,3-dione (S8i) was prepared according to the general procedure A,

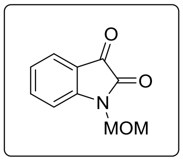

by using indoline-2,3-dione (2.000 g, 13.95 mmol) and chloromethyl methyl ether. After silica gel column chromatography with EtOAc / petroleum ether = 1/2 (R_f = 0.51) as eluents the desired product was obtained in 64% yield (1.662 g, 8.69 mmol) as an orange solid. The analytical data were identical to the literature data.[8] **^{1}H NMR** (500 MHz, $CDCl_3$) δ 7.65 (d, *J* = 7.7 Hz, 1H), 7.62 (td, *J* = 7.7, 1.2 Hz, 1H), 7.18 (t, *J* = 7.7 Hz, 1H), 7.12 (d, *J* = 7.7 Hz, 1H), 5.16 (s, 2H), 3.38 (s, 3H).

1-((2-(trimethylsilyl)ethoxy)methyl)indoline-2,3-dione (S8j) was prepared according to the

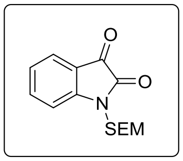

general procedure A, by using indoline-2,3-dione (2.000 g, 13..59 mmol) and 2-(trimethylsilyl)ethoxymethyl chloride. After silica gel column chromatography with EtOAc / petroleum ether = 1/2 (R_f = 0.79) as eluents the desired product was obtained in 97% yield (3.640 g, 13.12 mmol) as an orange solid. The analytical data were identical to the literature data.[9] **^{1}H NMR** (500 MHz, $CDCl_3$) δ 7.69 – 7.60 (m, 2H), 7.18 (td, *J* = 7.6, 0.7 Hz, 1H), 7.14 (d, *J* = 8.2 Hz, 1H), 5.18 (s, 2H), 3.60 (dd, *J* = 8.3, 7.8 Hz, 2H), 0.93 (dd, *J* = 8.3, 7.8 Hz, 2H), -0.02 (s, 9H).

1,5,7-trimethylindoline-2,3-dione (S8k) was prepared according to the general procedure A, by

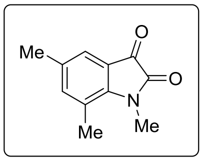

using 5,7-dimethylindoline-2,3-dione (1000 mg, 5.71 mmol) and iodomethane. After silica gel column chromatography with EtOAc / DCM = 1/2 (R_f = 0.3) as eluents the desired product was obtained in 72% yield (781 mg, 4.13 mmol) as a dark red solid. **^{1}H NMR** (500 MHz, $CDCl_3$) δ 7.25 (s, 1H), 7.13 (s, 1H), 3.48 (s, 3H), 2.51 (s, 3H), 2.27 (s, 3H). **^{13}C NMR** (126 MHz, $CDCl_3$) δ 184.15, 159.49, 146.92, 142.83, 133.72, 123.83, 121.71, 118.76, 29.78, 20.47, 18.79. **HRMS** (ESI): Calcd for $(M + H)^+$ $[C_{11}H_{12}O_2N]^+$: 190.0863, found: 190.0869.

3-hydroxy-1-methyl-3-(phenylethynyl)indolin-2-one (**S9a**) was prepared according to the

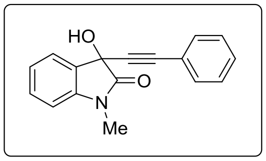

general procedure B, by using 1-methylisatin (1.000 g, 6.21 mmol) and phenylacetylene. After silica gel column chromatography with EtOAc / petroleum ether = 1/2 (R_f = 0.32) as eluents the desired product was

obtained in 81% yield (1.326 g, 5.04 mmol) as a pale yellow solid. The analytical data were identical to the literature data.[10] ^{1}**H NMR** (500 MHz, $CDCl_3$) δ 7.61 (dd, *J* = 7.6, 0.7 Hz, 1H), 7.46 – 7.40 (m, 2H), 7.38 (td, *J* = 7.6, 1.2 Hz, 1H), 7.34 – 7.21 (m, 3H), 7.16 (td, *J* = 7.6, 0.7 Hz, 1H), 6.85 (d, *J* = 7.6 Hz, 1H), 3.86 (s, 1H), 3.23 (s, 3H).

3-hydroxy-1-methyl-3-(p-tolylethynyl)indolin-2-one (**S9b**) was prepared according to the

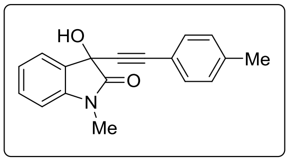

general procedure B, by using 1-methylisatin (500 mg, 3.10 mmol) and 4-ethynyltoluene. After silica gel column chromatography with EtOAc / DCM = 1/15 (R_f = 0.34) as eluents the desired product was obtained in 88% yield (755 mg, 2.72 mmol) as a yellow solid The analytical data were identical to the literature data.[10] ^{1}**H NMR** (500 MHz, $CDCl_3$) δ 7.61 (d, *J* = 7.6 Hz, 1H), 7.37 (t, *J* = 7.6 Hz, 1H), 7.32 (d, *J* = 8.0 Hz, 2H), 7.15 (t, *J* = 7.6 Hz, 1H), 7.06 (d, *J* = 8.0 Hz, 2H), 6.84 (d, *J* = 7.6 Hz, 1H), 3.82 (s, 1H), 3.23 (s, 3H), 2.32 (s, 3H).

3-hydroxy-1-methyl-3-(m-tolylethynyl)indolin-2-one (**S9c**) was prepared according to the

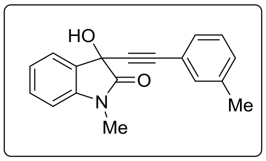

general procedure B, by using 1-methylisatin (300 mg, 1.86 mmol) and 3-ethynyltoluene. After silica gel column chromatography with EtOAc / DCM = 1/15 (R_f = 0.32) as eluents the desired product was obtained in 90% yield (466 mg, 1.68 mmol) as a pale yellow solid. **mp**: 159 - 161 ^oC ^{1}**H NMR** (500 MHz, $CDCl_3$) δ 7.61 (d, *J* = 7.6 Hz, 1H), 7.37 (t, *J* = 7.6 Hz, 1H), 7.28 – 7.21 (m, 2H), 7.20 – 7.08 (m, 3H), 6.85 (d, *J* = 7.6 Hz, 1H), 3.84 (s, 1H), 3.23 (d, *J* = 0.9 Hz, 3H), 2.27 (s, 3H). ^{13}**C NMR** (126 MHz, $CDCl_3$) δ 174.10, 143.21, 138.04, 132.78, 130.59, 130.00, 129.25, 129.12, 128.23, 124.85, 123.86, 121.55, 108.96, 86.69, 85.29, 69.69, 26.77, 21.24. **HRMS** (ESI): Calcd for $(M + Na)^+$ $[C_{18}H_{15}O_2NNa]^+$: 300.0995, found: 300.1004.

3-((3,4-dimethoxyphenyl)ethynyl)-3-hydroxy-1-methylindolin-2-one (**S9d**) was prepared

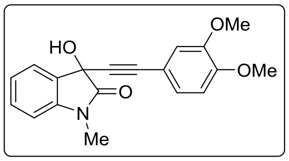

according to the general procedure B, by using 1-methylisatin (100 mg, 0.62 mmol) and 3,4-dimethoxyphenyl acetylene. After silica gel column chromatography with EtOAc / DCM = 1/6 (R_f = 0.35) as eluents the desired product was obtained in 90% yield (180 mg, 0.56 mmol) as a pale yellow solid. **mp**: 160

- 161 °C **^{1}H NMR** (500 MHz, $CDCl_3$) δ 7.58 (dd, *J* = 7., 0.6 Hz, 1H), 7.28 (td, *J* = 7.6, 1.1 Hz, 1H), 7.09 (td, *J* = 7.6, 0.6 Hz, 1H), 6.96 (dd, *J* = 8.3, 1.8 Hz, 1H), 6.87 (d, *J* = 1.8 Hz, 1H), 6.74 (d, *J* = 7.6 Hz, 1H), 6.63 (d, *J* = 8.3 Hz, 1H), 5.12 (s, 1H), 3.77 (s, 3H), 3.68 (s, 3H), 3.12 (s, 3H). **^{13}C NMR** (126 MHz, $CDCl_3$) δ 174.32, 149.79, 148.32, 142.76, 130.17, 129.39, 125.44, 124.52, 123.61, 114.62, 113.74, 110.71, 108.75, 86.27, 84.24, 69.53, 55.71, 26.49. **HRMS** (ESI): Calcd for $(M +Na)^+$ $[C19H17O4NNa]^+$: 346.1050, found: 346.1055.

3-hydroxy-3-((2-methoxyphenyl)ethynyl)-1-methylindolin-2-one (**S9e**) was prepared

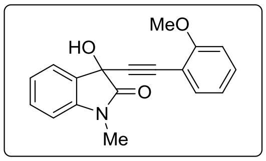

according to the general procedure B, by using 1-methylisatin (300 mg, 1.86 mmol) and 2-ethynylanisole. After silica gel column chromatography with EtOAc / DCM = 1/10 (R_f = 0.24) as eluents the desired product was obtained in 92% yield (503 mg, 1.71 mmol) as a white solid The analytical data were identical to the literature data.[10] **^{1}H NMR** (500 MHz, $CDCl_3$) δ 7.62 (dd, *J* = 7.6, 0.8 Hz, 1H), 7.40 – 7.33 (m, 2H), 7.29 (ddd, *J* = 8.0, 7.6, 1.7 Hz, 1H), 7.15 (td, *J* = 8.0, 0.8 Hz, 1H), 6.90 – 6.81 (m, 3H), 3.85 (s, 3H), 3.24 (s, 3H).

3-((4-fluorophenyl)ethynyl)-3-hydroxy-1-methylindolin-2-one (**S9f**) was prepared according

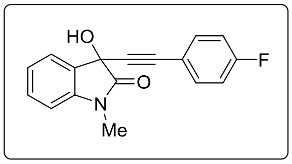

to the general procedure B, by using 1-methylisatin (288 mg, 1.79 mmol) and 1-ethynyl-4-fluorobenzene. After silica gel column chromatography with EtOAc / petroleum ether = 1/2 (R_f = 0.32) as eluents the desired product was obtained in 84% yield (422 mg, 1.50 mmol) as a white solid. **mp**: 159 - 160 °C **^{1}H NMR** (500 MHz, $cdcl_3$) δ 7.60 (d, *J* = 7.6 Hz, 1H), 7.45 – 7.34 (m, 3H), 7.16 (t, *J* = 7.6 Hz, 1H), 6.96 (t, *J* = 8.7 Hz, 2H), 6.86 (d, *J* = 7.6 Hz, 1H), 3.80 (s, 1H), 3.24 (s, 3H). **^{13}C NMR** (126 MHz, $cdcl_3$) δ 174.07, 163.01 (d, *J* = 250.6 Hz), 143.17, 134.21 (d, *J* = 8.6 Hz), 130.66, 129.00, 124.83, 123.93, 117.86 (d, *J* = 3.5 Hz), 115.68 (d, *J* = 22.2 Hz), 109.02, 85.46 (d, *J* = 1.5 Hz), 85.39, 69.64, 26.79. **HRMS** (ESI): Calcd for $(M + H)^+$ $[C_{17}H_{13}O_2NF]^+$: 282.0925, found: 282.0924.

3-hydroxy-1-methyl-3-(thiophen-3-ylethynyl)indolin-2-one (**S9g**) was prepared according to

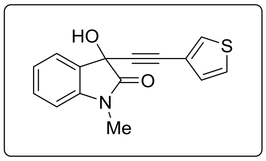

the general procedure B, by using 1-methylisatin (292 mg, 1.81 mmol) and 3-ethynylthiophene. After silica gel column chromatography with EtOAc / petroleum ether = 1/10 (R_f = 0.38) as eluents the desired product was obtained in 87% yield (426 mg, 1.58 mmol) as a brown solid. **mp**: 199 - 201 ^{o}C **^{1}H NMR** (500 MHz, $CDCl_3$) δ 7.60 (d, *J* = 7.6 Hz, 1H), 7.48 (d, *J* = 2.9 Hz, 1H), 7.38 (t, *J* = 7.6 Hz, 1H), 7.22 (dd, *J* = 5.0, 2.9 Hz, 1H), 7.16 (t, *J* = 7.6 Hz, 1H), 7.09 (d, *J* = 5.0 Hz, 1H), 6.86 (d, *J* = 7.6 Hz, 1H), 3.58 (s, 1H), 3.24 (s, 3H). **^{13}C NMR** (126 MHz, $CDCl_3$) δ 173.95, 143.24, 130.68, 130.51, 130.10, 128.92, 125.48, 124.87, 123.89, 120.82, 109.00, 85.28, 81.77, 69.71, 26.80. **HRMS** (ESI): Calcd for $(M + H)^+$ $[C_{15}H_{12}O_2NS]^+$: 270.0583, found: 270.0591.

3-hydroxy-1-methyl-3-(pent-1-yn-1-yl)indolin-2-one (**S9h**) was prepared according to the

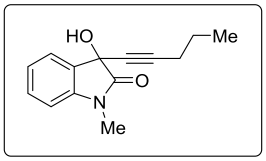

general procedure B, by using 1-methylisatin (1.000 g, 6.21 mmol) and 1-pentyne. After silica gel column chromatography with EtOAc / petroleum ether = 1/2 (R_f = 0.26) as eluents the desired product was obtained in 72% yield (1.021 g, 4.45 mmol) as a brownf solid. **mp**: 122 - 124 ^{o}C **^{1}H NMR** (300 MHz, $CDCl_3$) δ 7.52 (dd, *J* = 7.6, 0.8 Hz, 1H), 7.35 (td, *J* = 7.6, 1.3 Hz, 1H), 7.13 (td, *J* = 7.6, 0.8 Hz, 1H), 6.83 (d, *J* = 7.6 Hz, 1H), 3.21 (s, 3H), 2.18 (t, *J* = 7.1 Hz, 2H), 1.51 (qt, *J* = 7.4, 7.1 Hz, 2H), 0.93 (t, *J* = 7.4 Hz, 3H). **^{13}C NMR** (75 MHz, $CDCl_3$) δ 174.42, 143.12, 130.41, 129.42, 124.58, 123.78, 108.87, 88.03, 77.36, 69.30, 26.69, 21.80, 20.93, 13.57. **HRMS** (ESI): Calcd for $(M + H)^+$ $[C_{14}H_{16}O_2N]^+$: 230.1176, found: 230.1184.

3-hydroxy-1-methyl-3-(5-methylhex-1-yn-1-yl)indolin-2-one (**S9i**) was prepared according to

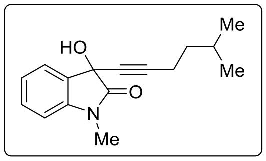

the general procedure B, by using 1-methylisatin (200 mg, 1.24 mmol) and 5-methyl-1-hexyne. After silica gel column chromatography with EtOAc / petroleum ether = 1/2 (R_f = 0.44) as eluents the desired product was obtained in 51% yield (162 mg, 0.63 mmol) as a brown solid. **mp**: 99 - 100 ^{o}C **^{1}H NMR** (300 MHz, $CDCl_3$) δ 7.51 (dd, *J* = 7.6, 0.8 Hz, 1H), 7.32 (td, *J* = 7.6, 1.3 Hz, 1H), 7.11 (td, *J* = 7.6, 0.8 Hz, 1H), 6.81 (d, *J* = 7.6 Hz, 1H), 4.12 (s, 1H), 3.18 (s, 3H), 2.18 (t, *J* = 7.4 Hz, 2H), 1.66 - 1.50 (m, 1H), 1.36 (td, *J* = 7.4, 7.4 Hz, 2H), 0.82 (dd, *J* = 6.6, 0.9 Hz, 6H). **^{13}C NMR** (75

MHz, $CDCl_3$) δ 174.58, 142.96, 130.24, 129.59, 124.51, 123.71, 108.78, 88.07, 77.36, 69.25, 37.16, 27.32, 26.61, 22.16, 16.95. **HRMS** (ESI): Calcd for $(M + Na)^+$ $[C_{16}H_{19}O_2NNa]^+$: 280.1308, found: 280.1314.

3-hydroxy-1,5-dimethyl-3-(phenylethynyl)indolin-2-one (**S9j**) was prepared according to the

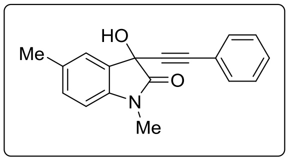

general procedure B, by using **S8a** (88 mg, 0.50 mmol) and phenylacetylene. After silica gel column chromatography with EtOAc / petroleum ether = 1/2 (R_f = 0.29) as eluents the desired product was obtained in 88% yield (122 mg, 0.44 mmol) as a brown solid. **mp**: 189 - 191 ^oC **^{1}H NMR** (500 MHz, $CDCl_3$) δ 7.49 – 7.40 (m, 3H), 7.37 – 7.22 (m, 3H), 7.17 (dd, *J* = 7.9, 0.8 Hz, 1H), 6.74 (d, *J* = 7.9 Hz, 1H), 3.73 (s, 1H), 3.22 (s, 3H), 2.37 (s, 3H). **^{13}C NMR** (126 MHz, $CDCl_3$) δ 173.96, 140.84, 133.63, 132.21, 130.88, 129.09, 128.92, 128.34, 125.59, 121.82, 108.75, 86.41, 85.80, 69.79, 26.81, 21.20. **HRMS** (ESI): Calcd for $(M + H)^+$ $[C_{18}H_{16}O_2N]^+$: 278.1176, found: 278.1177.

3-hydroxy-5-methoxy-1-methyl-3-(phenylethynyl)indolin-2-one (**S9k**) was prepared

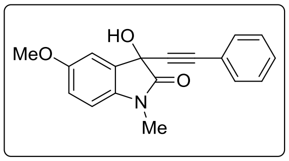

according to the general procedure B, by using **S8b** (585 mg, 3.06 mmol) and phenylacetylene. After silica gel column chromatography with EtOAc / petroleum ether = 1/2 (R_f = 0.25) as eluents the desired product was obtained in 82% yield (734 mg, 2.50 mmol) as a brown solid. **mp**: 157 - 158 ^oC **^{1}H NMR** (500 MHz, $CDCl_3$) δ 7.41 (d, *J* = 7.1 Hz, 2H), 7.37 – 7.18 (m, 4H), 6.89 (dd, *J* = 8.5, 2.5 Hz, 1H), 6.74 (d, *J* = 8.5 Hz, 1H), 4.41 (s, 1H), 3.82 (s, 3H), 3.19 (s, 3H). **^{13}C NMR** (126 MHz, $CDCl_3$) δ 173.96, 156.92, 136.44, 132.18, 130.21, 129.03, 128.28, 121.78, 115.49, 111.56, 109.52, 86.46, 85.76, 70.00, 56.04, 26.82. **HRMS** (ESI): Calcd for $(M + H)^+$ $[C_{18}H_{16}O_3N]^+$: 294.1125, found: 294.1126.

6-bromo-3-hydroxy-1-methyl-3-(phenylethynyl)indolin-2-one (**S9l**) was prepared according

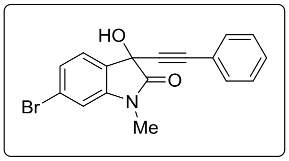

to the general procedure B, by using **S8c** (400 mg, 1.67 mmol) and phenylacetylene. After silica gel column chromatography with EtOAc / petroleum ether = 1/2 (R_f = 0.46) as eluents the desired product was

obtained in % yield (492 mg, 1.44 mmol) as a pale yellow solid. **mp**: 177 - 179 °C **^{1}H NMR** (500 MHz, cdcl$_3$) δ 7.46 (d, *J* = 7.9 Hz, 2H), 7.44 – 7.38 (m, 4H), 7.34 – 7.23 (m, 9H), 7.00 (d, *J* = 1.6 Hz, 2H), 3.89 (s, 2H), 3.21 (s, 6H). **^{13}C NMR** (126 MHz, CDCl$_3$) δ 174.03, 144.39, 132.16, 129.22, 128.35, 128.05, 126.71, 126.13, 124.28, 121.51, 112.56, 86.78, 85.04, 69.29, 26.88. HRMS (ESI): Calcd for $(M+Na)^+$ [C17H12O2NBrNa]$^+$: 363.9944, found: 363.9951.

5-fluoro-3-hydroxy-1-methyl-3-(phenylethynyl)indolin-2-one (**S9m**) was prepared according

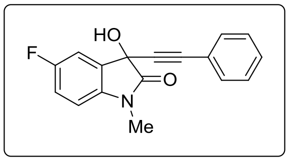

to the general procedure B, by using **S8d** (400 mg, 2.23 mmol) and phenylacetylene. After silica gel column chromatography with EtOAc / petroleum ether = 2/3 (R_f = 0.34) as eluents the desired product was obtained in 55% yield (345 mg, 1.23mmol) as a brown solid. The analytical data were identical to the literature data.[10] **^{1}H NMR** (500 MHz, CDCl$_3$) δ 7.44 (d, *J* = 7.4 Hz, 2H), 7.39 (dd, *J* = 7.2, 1.9 Hz, 1H), 7.37 – 7.26 (m, 3H), 7.09 (td, *J* = 8.7, 2.0 Hz, 1H), 6.79 (dd, *J* = 8.4, 3.7 Hz, 1H), 4.43 (s, 1H), 3.24 (s, 3H).

3-hydroxy-1-methyl-3-(phenylethynyl)-5-(trifluoromethoxy)indolin-2-one (**S9n**) was

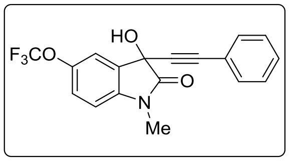

prepared according to the general procedure B, by using **S8e** (500 mg, 2.04 mmol) and phenylacetylene. After silica gel column chromatography with EtOAc / petroleum ether = 1/2 (R_f = 0.33) as eluents the desired product was obtained in 68% yield (485 mg, 1.40 mmol) as a yellow solid. **mp**: 130 - 133 °C ^{1}H NMR (500 MHz, CDCl$_3$) δ 7.50 (s, 1H), 7.43 (d, *J* = 7.0 Hz, 2H), 7.33 - 7.24 (m, 4H), 6.84 (d, *J* = 8.5 Hz, 1H), 3.90 (s, 1H), 3.24 (s, 3H). **^{13}C NMR** (126 MHz, CDCl$_3$) δ 173.93, 145.65, 141.80, 132.24, 130.39, 129.38, 128.42, 123.66, 121.39, 120.66 (d, *J* = 257.0 Hz), 118.91, 109.59, 87.18, 84.79, 69.54, 26.96. **HRMS** (ESI): Calcd for $(M + H)^+$ $[C_{18}H_{13}O_3NF_3]^+$: 348.0842, found: 348.0848.

3-hydroxy-1-methyl-5-nitro-3-(phenylethynyl)indolin-2-one (**S9o**) was prepared according to

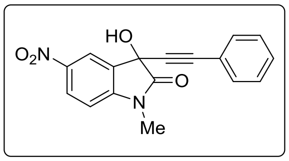

the general procedure B, by using **S8f** (400 mg, 1.94 mmol) and phenylacetylene. After silica gel column chromatography with EtOAc / petroleum ether = 1/2 (R_f = 0.22) as eluents the desired product was

obtained in 58% yield (346 mg, 1.12 mmol) as a brown solid. **mp**: 176 - 178 °C **^{1}H NMR** (400 MHz, $CDCl_3$) δ 8.48 (d, *J* = 2.3 Hz, 1H), 8.33 (dd, *J* = 8.7, 2.3 Hz, 1H), 7.47 – 7.38 (m, 2H), 7.36 – 7.21 (m, 3H), 6.94 (d, *J* = 8.7 Hz, 1H), 4.50 (s, 1H), 3.29 (s, 3H). **^{13}C NMR** (126 MHz, $CDCl_3$) δ 174.24, 148.57, 144.41, 132.25, 129.89, 129.59, 128.46, 127.46, 121.02, 120.96, 108.79, 87.77, 84.00, 68.98, 27.23. **HRMS** (ESI): Calcd for $(M + H)^+$ $[C_{17}H_{13}O_4N_2]^+$: 309.0870, found: 309.0868.

5-chloro-3-hydroxy-1-methyl-3-(phenylethynyl)indolin-2-one (**S9p**) was prepared according to the general procedure B, by using **S8g** (400 mg, 2.04 mmol) and phenylacetylene. After silica gel column chromatography with EtOAc / petroleum ether = 1/2 (R_f = 0.53) as eluents the desired product was obtained in 94% yield (574 mg, 1.93 mmol) as a brown solid. The analytical data were identical to the literature data.[10] **^{1}H NMR** (500 MHz, $CDCl_3$) δ 7.59 (d, *J* = 2.0 Hz, 1H), 7.47 – 7.39 (m, 2H), 7.36 – 7.29 (m, 2H), 7.29 – 7.22 (m, 2H), 6.76 (dd, *J* = 8.3, 2.1 Hz, 1H), 4.15 (s, 1H), 3.21 (d, *J* = 1.9 Hz, 3H).

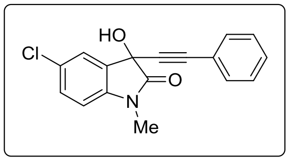

1-benzyl-3-hydroxy-3-(phenylethynyl)indolin-2-one (**S9q**) was prepared according to the general procedure B, by using 1-benzyl-1H-indole-2,3-dione (500 mg, 2.11 mmol) and phenylacetylene. After silica gel column chromatography with EtOAc / petroleum ether = 1/2 (R_f = 0.29) as eluents the desired product was obtained in 71% yield (511 mg, 1.51 mmol) as a white solid. The analytical data were identical to the literature data.[10] **^{1}H NMR** (500 MHz, $CDCl_3$) δ 7.62 (d, *J* = 7.4 Hz, 1H), 7.46 (d, *J* = 7.0 Hz, 2H), 7.37 – 7.20 (m, 11H), 7.12 (t, *J* = 7.4 Hz, 1H), 6.73 (d, *J* = 7.4 Hz, 1H), 4.94 (s, 2H), 3.58 (s, 1H).

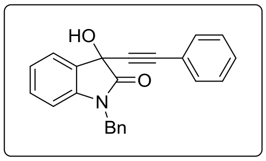

3-hydroxy-1-(4-methoxybenzyl)-3-(phenylethynyl)indolin-2-one (**S9r**) was prepared according to the general procedure B, by using **S8h** (134 mg, 0.50 mmol) and phenylacetylene. After silica gel column chromatography with EtOAc / petroleum ether = 1/2 (R_f = 0.39) as eluents the desired product was obtained in 38% yield (70 mg, 0.19 mmol) as a pale yellow solid. **mp**: 228 - 229 °C **^{1}H NMR**

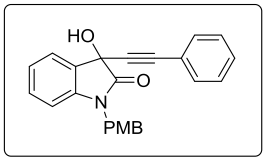

(500 MHz, DMSO) δ 7.52 (dd, *J* = 7.6, 0.7 Hz, 1H), 7.47 – 7.34 (m, 4H), 7.34 – 7.24 (m, 4H), 7.10 (td, *J* = 7.6, 0.7 Hz, 1H), 6.98 (d, *J* = 7.6 Hz, 1H), 6.90 (d, *J* = 8.7 Hz, 2H), 4.87 (d, *J* = 15.5 Hz, 1H), 4.82 (d, *J* = 15.5 Hz, 1H), 3.71 (s, 3H). **^{13}C NMR** (126 MHz, DMSO) δ 173.14, 158.65, 141.50, 131.49, 130.27, 129.88, 129.17, 128.73, 128.65, 127.80, 124.22, 123.11, 121.17, 114.07, 109.81, 87.58, 84.41, 68.81, 55.04, 42.25. **HRMS** (ESI): Calcd for $(M + H)^+$ $[C_{24}H_{20}O_3N]^+$: 370.1438, found: 370.1452.

3-hydroxy-1-(methoxymethyl)-3-(phenylethynyl)indolin-2-one (**S9s**) was prepared according to the general procedure B, by using **S8i** (200 mg, 1.05 mmol) and phenylacetylene. After silica gel column chromatography with EtOAc / petroleum ether = 1/3 (R_f = 0.37) as eluents the desired product was obtained in 73% yield (224 mg, 0.76 mmol) as a brown solid. **mp**: 158 - 159 °C **^{1}H NMR** (300 MHz, $CDCl_3$) δ 7.62 (dd, *J* = 7.6, 0.8 Hz, 1H), 7.44 – 7.37 (m, 2H), 7.34 (dd, *J* = 7.6, 1.3 Hz, 1H), 7.31 – 7.22 (m, 3H), 7.17 (td, *J* = 7.6, 0.8 Hz, 1H), 7.05 (d, *J* = 7.6 Hz, 1H), 5.15 (d, *J* = 11.0 Hz, 1H), 5.11 (d, *J* = 11.0 Hz, 1H), 4.22 (s, 1H), 3.33 (s, 3H). **^{13}C NMR** (75 MHz, $CDCl_3$) δ 174.85, 141.36, 132.16, 130.72, 129.18, 128.68, 128.35, 124.99, 124.37, 121.62, 110.51, 86.84, 85.44, 71.97, 69.93, 56.52. **HRMS** (ESI): Calcd for $(M + Na)^+$ $[C_{18}H_{15}O_3NNa]^+$: 316.0944, found: 316.0950.

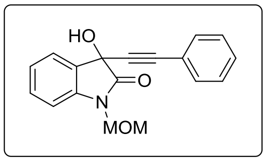

3-hydroxy-3-(phenylethynyl)-1-((2-(trimethylsilyl)ethoxy)methyl)indolin-2-one (**S9t**) was prepared according to the general procedure B, by using **S8j** (100 mg, 0.36 mmol) and phenylacetylene. After silica gel column chromatography with EtOAc / petroleum ether = 1/5 (R_f = 0.44) as eluents the desired product was obtained in 52% yield (71 mg, 0.19 mmol) as a brown solid. **mp**: 97 - 99 °C **^{1}H NMR** (300 MHz, $CDCl_3$) δ 7.62 (dd, *J* = 7.6, 0.9 Hz, 1H), 7.47 – 7.23 (m, 6H), 7.19 (td, *J* = 7.6, 0.9 Hz, 1H), 7.09 (d, *J* = 7.6 Hz, 1H), 5.21 (d, *J* = 11.1 Hz, 1H), 5.15 (d, *J* = 11.1 Hz, 1H), 3.69 – 3.56 (m, 2H), 1.69 (s, 1H), 0.99 – 0.87 (m, 2H), -0.05 (s, 9H). **^{13}C NMR** (75 MHz, $CDCl_3$) δ 174.44, 141.65, 132.19, 130.74, 129.22, 128.56, 128.38, 124.93, 124.25, 121.64, 110.64, 86.83, 85.46, 70.04, 69.95, 66.42, 17.85, -1.34. **HRMS** (ESI): Calcd for $(M + Na)^+$ $[C_{22}H_{25}O_3NSiNa]^+$: 402.1496, found: 402.1508.

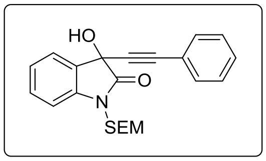

3-((3-chlorophenyl)ethynyl)-3-hydroxy-1-methylindolin-2-one (**S9u**) was prepared according

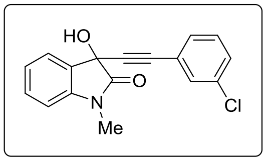

to the general procedure B, by using 1-methylisatin (337 mg, 2.09 mmol) and 3-chloro-1-ethynylbenzene. After silica gel column chromatography with EtOAc / petroleum ether = 1/2 (R_f = 0.38) as eluents the desired product was obtained in 79% yield (489 mg, 1.64 mmol) as a pale yellow solid. **mp**: 147 - 149 ^{o}C **^{1}H NMR** (500 MHz, $CDCl_3$) δ 7.60 (dd, *J* = 7.6, 0.8 Hz, 1H), 7.44 – 7.41 (m, 1H), 7.39 (td, *J* = 7.6, 1.2 Hz, 1H), 7.34 – 7.27 (m, 2H), 7.19 (t, *J* = 7.6 Hz, 1H), 7.17 (td, *J* = 7.6, 0.8 Hz, 1H), 6.87 (d, *J* = 7.6 Hz, 1H), 3.86 (s, 1H), 3.24 (s, 3H). **^{13}C NMR** (126 MHz, $CDCl_3$) δ 173.88, 143.23, 134.25, 132.06, 130.80, 130.29, 129.61, 129.44, 128.77, 124.89, 123.99, 123.48, 109.08, 86.88, 84.95, 69.62, 26.82. **HRMS** (ESI): Calcd for $(M + H)^+$ $[C_{17}H_{13}O_2NCl]^+$: 298.0629, found: 298.0634.

3-((4-bromophenyl)ethynyl)-3-hydroxy-1-methylindolin-2-one (**S9v**) was prepared according

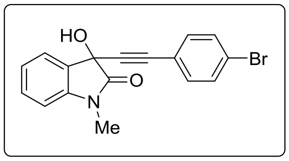

to the general procedure B, by using 1-methylisatin (300 mg, 1.86 mmol) and 1-bromo-4-ethynylbenzene. After silica gel column chromatography with EtOAc / DCM = 1/15 (R_f = 0.31) as eluents the desired product was obtained in 85% yield (543 mg, 1.59 mmol) as a pale yellow solid. The analytical data were identical to the literature data.[10] **^{1}H NMR** (300 MHz, $CDCl_3$) δ 7.63 (d, *J* = 7.4 Hz, 1H), 7.48 – 7.35 (m, 3H), 7.32 – 7.24 (m, 2H), 7.18 (t, *J* = 7.7 Hz, 1H), 6.86 (d, *J* = 7.7 Hz, 1H), 4.42 (s, 1H), 3.24 (s, 3H).

3-hydroxy-1,5,7-trimethyl-3-(phenylethynyl)indolin-2-one (**S9w**) was prepared according to

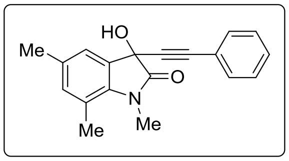

the general procedure B, by using **S8k** (400 mg, 2.11 mmol) and phenylacetylene. After silica gel column chromatography with EtOAc / petroleum ether = 1/2 (R_f = 0.32) as eluents the desired product was obtained in 64% yield (393 mg, 1.35 mmol) as a brown solid. **mp**: 172 - 174 ^{o}C **^{1}H NMR** (500 MHz, $CDCl_3$) δ 7.43 (d, *J* = 8.0 Hz, 2H), 7.35 – 7.20 (m, 4H), 6.90 (s, 1H), 3.59 (s, 1H), 3.48 (s, 3H), 2.51 (s, 3H), 2.31 (s, 3H). **^{13}C NMR** (126 MHz, $CDCl_3$) δ 174.64, 138.38, 134.78, 133.49, 132.19, 129.61, 129.03, 128.31, 123.51, 121.90, 120.36, 86.29, 86.09, 69.30, 30.18, 20.86, 18.89. **HRMS** (ESI): Calcd for $(M + H)^+$ $[C_{19}H_{18}O_2N]^+$: 292.1332, found: 292.1336.

(E)-3-(but-2-en-1-yloxy)-1-methyl-3-(phenylethynyl)indolin-2-one (**1a**) was prepared

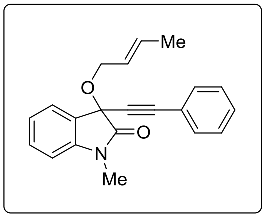

according to the general procedure C, by using **S9a** (582 mg, 2.21 mmol) and crotyl bromide. After silica gel column chromatography with EtOAc / petroleum ether = 1/7 (R_f = 0.34) as eluents the desired product was obtained in 91% yield (640 mg, 2.02 mmol, 82% (E)-isomer) as a pale yellow oil. **^{1}H NMR** (500 MHz, $CDCl_3$) δ 7.55 (d, *J* = 7.4 Hz, 1H, E and Z), 7.46 (dd, *J* = 8.0, 1.6 Hz, 2H, E and Z), 7.35 (td, *J* = 7.6, 0.8 Hz, 1H, E and Z), 7.32 – 7.22 (m, 3H, E and Z), 7.13 (td, *J* = 7.6, 0.8 Hz, 1H, E and Z), 6.82 (d, *J* = 7.6 Hz, 1H, E and Z), 5.80 – 5.69 (m, 1H, E and Z), 5.68 – 5.58 (m, 1H, E and Z), 4.53 (dd, *J* = 10.7, 6.0 Hz, 1H, Z), 4.47 (dd, *J* = 10.7, 6.0 Hz, 1H, Z), 4.39 (dd, *J* = 10.7, 6.3 Hz, 4H, E), 4.31 (dd, *J* = 10.7, 6.3 Hz, 1H, E), 3.21 (s, 3H, Z), 3.21 (s, 3H, E), 1.68 (dd, *J* = 6.3, 1.0 Hz, 3H, E), 1.64 (d, *J* = 5.3 Hz, 3H, Z). **HRMS** (ESI): Calcd for $(M +Na)^+$ $[C21H19O2NNa]^+$: 340.1308, found: 340.1319.

(E)-3-(but-2-en-1-yloxy)-1-methyl-3-(p-tolylethynyl)indolin-2-one (**1b**) was prepared

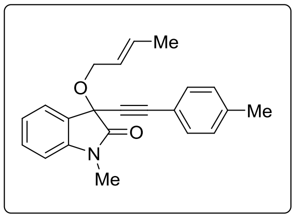

according to the general procedure C, by using **S9b** (180 mg, 0.65 mmol) and crotyl bromide. After silica gel column chromatography with EtOAc / petroleum ether = 1/7 (R_f = 0.30) as eluents the desired product was obtained in 57% yield (122 mg, 0.37 mmol, 77% (E)-isomer) as a brown oil. **^{1}H NMR** (500 MHz, $CDCl_3$) δ 7.55 (d, *J* = 7.6 Hz, 1H, E and Z), 7.35 (d, *J* = 8.0 Hz, 3H, E and Z), 7.12 (t, *J* = 7.6 Hz, 1H, E and Z), 7.08 (d, *J* = 8.0 Hz, 2H, E and Z), 6.82 (d, *J* = 7.6 Hz, 1H, E and Z), 5.77 – 5.68 (m, 1H, E and Z), 5.68 – 5.58 (m, 1H, E and Z), 4.52 (dd, *J* = 10.6, 5.8 Hz, 1H, Z), 4.47 (dd, *J* = 10.6, 5.8 Hz, 1H, Z), 4.38 (dd, *J* = 10.3, 6.6 Hz, 1H, E), 4.31 (dd, *J* = 10.3, 6.6 Hz, 1H, E), 3.21 (s, 3H, Z), 3.20 (s, 3H, E), 2.32 (s, 3H, E and Z), 1.67 (d, *J* = 6.2 Hz, 3H, E), 1.64 (d, *J* = 5.7 Hz, 3H, Z). **HRMS** (ESI): Calcd for $(M + Na)^+$ $[C_{22}H_{21}O_2NNa]^+$: 354.1465, found: 354.1479.

(E)-3-(but-2-en-1-yloxy)-3-((4-fluorophenyl)ethynyl)-1-methylindolin-2-one (**1c**) was

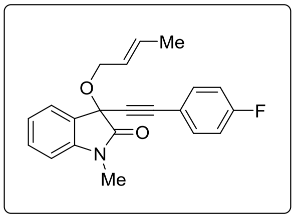

prepared according to the general procedure C, by using **S9f** (234 mg, 0.70 mmol) and crotyl bromide. After silica gel column chromatography with EtOAc / petroleum ether = 1/5 (R_f = 0.32) as eluents the desired product was obtained in 98% yield (234 mg, 0.70 mmol, 78% (E)-isomer) as a brown oil. **^{1}H NMR** (500 MHz, $CDCl_3$) δ 7.54 (d, *J* = 7.5 Hz, 1H, E and Z), 7.51 – 7.39 (m, 2H, E and Z), 7.37 (td, *J* = 7.5, 0.8 Hz, 1H, E and Z), 7.14 (t, *J* = 7.5 Hz, 1H, E and Z), 6.98 (t, *J* = 8.7 Hz, 2H, E and Z), 6.84 (d, *J* = 7.5 Hz, 1H, E and Z), 5.78 – 5.68 (m, 1H, E and Z), 5.67 – 5.57 (m, 1H, E and Z), 4.47 (dd, *J* = 11.0, 6.5 Hz, 1H, Z), 4.42 (dd, *J* = 10.9, 6.6 Hz, 1H, Z), 4.34 (dd, *J* = 10.5, 6.5 Hz, 1H, E), 4.26 (dd, *J* = 10.5, 6.4 Hz, 1H, E), 3.23 (s, 3H, Z), 3.23 (s, 3H, E), 1.67 (d, *J* = 6.3 Hz, 3H, E), 1.63 (d, *J* = 6.1 Hz, 3H, Z). **HRMS** (ESI): Calcd for $(M +Na)^+$ $[C21H18O2NFNa]^+$: 358.1214, found: 358.1229.

(E)-3-(but-2-en-1-yloxy)-3-((3-chlorophenyl)ethynyl)-1-methylindolin-2-one (**1d**) was

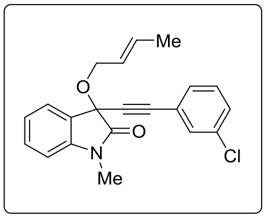

prepared according to the general procedure C, by using **S9u** (200 mg, 0.67 mmol) and crotyl bromide. After silica gel column chromatography with EtOAc / petroleum ether = 1/5 (R_f = 0.30) as eluents the desired product was obtained in 94% yield (223 mg, 0.63 mmol, 80% (E)-isomer) as a brown oil. **^{1}H NMR** (500 MHz, $CDCl_3$) δ 7.53 (d, *J* = 6.6 Hz, 1H, E and Z), 7.45 (s, 1H, E and Z), 7.37 (td, *J* = 7.8, 1.2 Hz, 1H, E and Z), 7.34 (d, *J* = 7.7 Hz, 1H, E and Z), 7.30 (d, *J* = 8.1 Hz, 1H, E and Z), 7.22 (t, *J* = 7.9 Hz, 1H, E and Z), 7.14 (td, *J* = 7.6, 0.8 Hz, 1H, E and Z), 6.84 (d, *J* = 7.8 Hz, 1H, E and Z), 5.77 – 5.70 (m, 1H, E and Z), 5.70 – 5.59 (m, 1H, E and Z), 4.49 (dd, *J* = 11.1, 6.7 Hz, 1H, Z), 4.42 (dd, *J* = 11.1, 6.6 Hz, 1H, Z), 4.35 (dd, *J* = 10.6, 6.4 Hz, 1H, E), 4.27 (dd, *J* = 10.6, 6.4 Hz, 1H, E), 3.24 (s, 3H, Z), 3.23 (s, 3H, E), 1.68 (dd, *J* = 6.3, 1.0 Hz, 3H, E), 1.63 (d, *J* = 6.2 Hz, 1H, Z). **HRMS** (ESI): Calcd for $(M +Na)^+$ $[C21H18O2NClNa]^+$: 374.0918, found: 370.0934.

(E)-3-(but-2-en-1-yloxy)-1-methyl-3-(thiophen-3-ylethynyl)indolin-2-one (**1e**) was prepared

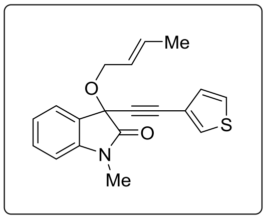

according to the general procedure C, by using **S9g** (200 mg, 0.74 mmol) and crotyl bromide. After silica gel column chromatography with EtOAc / petroleum ether = 1/6 (R_f = 0.31) as eluents the desired product was obtained in 88% yield (211 mg, 0.65 mmol, 83% (E)-isomer) as a yellow oil. **^{1}H NMR** (500 MHz, $CDCl_3$) δ 7.53 (d, *J* = 7.6 Hz, 1H, E and Z), 7.50 (dd, *J* = 3.0, 0.9 Hz, 1H, E nd Z), 7.36 (td, *J* = 7.6, 1.2 Hz, 1H, E nd Z), 7.22 (dd, *J* = 5.0, 3.0 Hz, 1H, E nd Z), 7.16 – 7.09 (m, 2H, E nd Z), 6.83 (d, *J* = 7.6 Hz, 1H, E nd Z), 5.77 – 5.67 (m, 1H, E nd Z), 5.67 – 5.57 (m, 1H, E nd Z), 4.48 (dd, *J* = 11.2, 6.3 Hz, 1H, Z), 4.42 (dd, *J* = 11.2, 6.3 Hz, 1H, Z), 4.35 (dd, *J* = 10.7, 6.3 Hz, 1H, E), 4.27 (dd, *J* = 10.7, 6.3 Hz, 1H, E), 3.22 (s, 3H, Z), 3.21 (s, 3H, E), 1.67 (dd, *J* = 6.3, 1.1 Hz, 3H, E), 1.63 (dd, *J* = 5.9, 0.5 Hz, 1H, Z). **HRMS** (ESI): Calcd for $(M +Na)^+$ $[C_{19}H_{17}O_2NSNa]^+$: 346.0872, found: 346.0882.

(E)-3-(but-2-en-1-yloxy)-5-methoxy-1-methyl-3-(phenylethynyl)indolin-2-one (**1f**) was

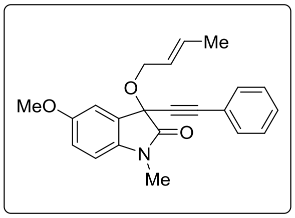

prepared according to the general procedure C, by using **S9k** (200 mg, 0.68 mmol) and crotyl bromide. After silica gel column chromatography with EtOAc / petroleum ether = 1/5 (R_f = 0.33) as eluents the desired product was obtained in 92% yield (218 mg, 0.63 mmol, 83% (E)-isomer) as a brown oil. **^{1}H NMR** (500 MHz, $CDCl_3$) δ 7.46 (d, *J* = 6.6 Hz, 2H, E and Z), 7.38 – 7.23 (m, 3H, E and Z), 7.17 (d, *J* = 2.5 Hz, 1H, E and Z), 6.88 (dd, *J* = 8.5, 2.5 Hz, 1H, E and Z), 6.74 (d, *J* = 8.5 Hz, 1H, E and Z), 5.78 – 5.68 (m, 1H), 5.69 – 5.59 (m, 1H), 4.50 (dd, *J* = 11.3, 6.0 Hz, 1H, Z), 4.45 (dd, *J* = 11.3, 6.0 Hz, 1H, Z), 4.36 (dd, *J* = 10.7, 6.4 Hz, 1H, E), 4.29 (dd, *J* = 10.7, 6.4 Hz, 1H, E), 3.81 (s, 3H, E and Z), 3.20 (s, 3H, Z), 3.19 (s, 3H, E), 1.68 (dd, *J* = 6.3, 1.0 Hz, 3H, E), 1.64 (d, *J* = 5.4 Hz, 3H, Z). **HRMS** (ESI): Calcd for $(M +Na)^+$ $[C22H21O3NNa]^+$: 370.1414, found: 370.1427.

(E)-3-(but-2-en-1-yloxy)-5-fluoro-1-methyl-3-(phenylethynyl)indolin-2-one (**1g**) was

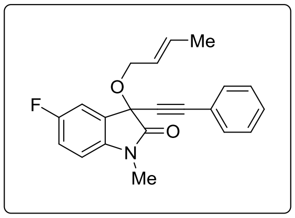

prepared according to the general procedure C, by using **S9m** (200 mg, 0.71 mmol) and crotyl bromide. After silica gel column chromatography with EtOAc / petroleum ether = 1/6 (R_f = 0.34) as eluents the desired product was obtained in 81% yield (194 mg, 0.58 mmol, 77% (E)-isomer) as a yellow oil. **^{1}H NMR** (500 MHz, $CDCl_3$) δ 7.46 (d, *J* = 6.6 Hz, 2H, E and Z), 7.41 – 7.23 (m, 4H, E and Z), 7.06 (td, *J* = 8.7, 2.6 Hz, 1H, E and Z), 6.76 (dd, *J* = 8.7, 3.9 Hz, 1H, E and Z), 5.81 – 5.70 (m, 1H, E and Z), 5.70 – 5.56 (m, 1H, E and Z), 4.55 (dd, *J* = 11.0, 6.7 Hz, 1H, Z), 4.49 (dd, *J* = 11.0, 6.7 Hz, 1H, Z), 4.41 (dd, *J* = 10.7, 6.4 Hz, 1H, E), 4.34 (dd, *J* = 10.7, 6.4 Hz, 1H, E), 3.21 (s, 3H, Z), 3.21 (s, 3H, E), 1.69 (dd, *J* = 6.4, 1.1 Hz, 3H, E), 1.66 (d, *J* = 6.7 Hz, 1H, Z). **HRMS** (ESI): Calcd for $(M+Na)^+$ $[C21H18O2NFNa]^+$: 358.1214, found: 358.1228.

(E)-3-(but-2-en-1-yloxy)-5-chloro-1-methyl-3-(phenylethynyl)indolin-2-one (**1h**) was

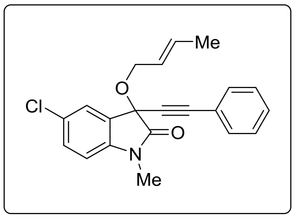

prepared according to the general procedure C, by using **S9p** (200 mg, 0.67 mmol) and crotyl bromide. After silica gel column chromatography with EtOAc / petroleum ether = 1/6 (R_f = 0.34) as eluents the desired product was obtained in 80% yield (190 mg, 0.54 mmol, 77% (E)-isomer) as a brown oil. **^{1}H NMR** (500 MHz, $CDCl_3$) δ 7.52 (d, *J* = 2.1 Hz, 1H, E and Z), 7.47 (d, *J* = 6.7 Hz, 2H, E and Z), 7.36 – 7.25 (m, 4H, E and Z), 6.76 (d, *J* = 8.3 Hz, 1H, E and Z), 5.80 – 5.67 (m, 1H, E and Z), 5.67 – 5.55 (m, 1H, E and Z), 4.57 (dd, *J* = 11.0, 6.8 Hz, 1H, Z), 4.51 (dd, *J* = 11.0, 6.8 Hz, 1H, Z), 4.43 (dd, *J* = 10.6, 6.5 Hz, 1H, E), 4.35 (dd, *J* = 10.6, 6.5 Hz, 1H, E), 3.21 (d, *J* = 3.6 Hz, 3H, Z), 3.20 (s, 3H, E), 1.69 (dd, *J* = 6.4, 0.9 Hz, 3H, E), 1.67 (d, *J* = 6.7 Hz, 3H, Z). **HRMS** (ESI): Calcd for $(M+Na)^+$ $[C21H18O2NClNa]^+$: 374.0918, found: 374.0932.

(E)-6-bromo-3-(but-2-en-1-yloxy)-1-methyl-3-(phenylethynyl)indolin-2-one (**1i**) was

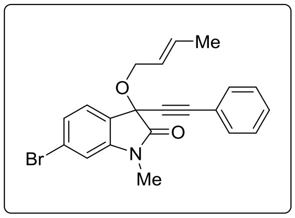

prepared according to the general procedure C, by using **S9l** (200 mg, 0.58 mmol) and crotyl bromide. After silica gel column chromatography with EtOAc / petroleum ether = 1/8 (R_f = 0.37) as eluents the desired product was obtained in 93% yield (220 mg, 0.56 mmol, 76% (E)-isomer) as a yellow oil. **^{1}H NMR** (500 MHz, $CDCl_3$) δ 7.45 (d, *J* = 6.7 Hz, 2H, E and Z), 7.43 –

7.38 (m, 1H, E and Z), 7.36 – 7.25 (m, 4H, E and Z), 6.99 (d, *J* = 1.6 Hz, 1H, E and Z), 5.79 – 5.67 (m, 1H, E and Z), 5.66 – 5.56 (m, 1H, E and Z), 4.53 (dd, *J* = 11.0, 6.7 Hz, 1H, Z), 4.47 (dd, *J* = 11.0, 6.7 Hz, 1H, Z), 4.39 (dd, *J* = 10.7, 6.5 Hz, 1H, E), 4.31 (dd, *J* = 10.7, 6.5 Hz, 1H, E), 3.21 (s, 3H, Z), 3.20 (s, 3H, E), 1.68 (dd, *J* = 6.4, 1.2 Hz, 3H, E), 1.65 (d, *J* = 6.7 Hz, 3H, Z). **HRMS** (ESI): Calcd for $(M +Na)^+$ $[C21H18O2NBrNa]^+$: 418.0413, found: 418.0430.

(E)-3-(but-2-en-1-yloxy)-1-methyl-3-(phenylethynyl)-5-(trifluoromethoxy)indolin-2-one (**1j**)

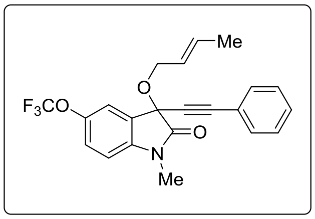

was prepared according to the general procedure C, by using **S9n** (200 mg, 0.58 mmol) and crotyl bromide. After silica gel column chromatography with EtOAc / petroleum ether = 1/5 (R_f = 0.29) as eluents the desired product was obtained in 80% yield (202 mg, 0.50 mmol, 80% (E)-isomer) as a pale yellow oil. **^{1}H NMR** (500 MHz, $CDCl_3$) δ 7.47 (d, *J* = 6.7 Hz, 2H, E and Z), 7.44 (d, *J* = 1.5 Hz, 1H, E and Z), 7.37 – 7.28 (m, 3H, E and Z), 7.24 (d, *J* = 8.5 Hz, 1H, E and Z), 6.82 (d, *J* = 8.5 Hz, 1H, E and Z), 5.80 – 5.68 (m, 1H, E and Z), 5.67 – 5.59 (m, 1H, E and Z), 4.57 (dd, *J* = 11.0, 6.7 Hz, 1H, Z), 4.50 (dd, *J* = 11.0, 6.7 Hz, 1H, Z), 4.43 (dd, *J* = 10.7, 6.5 Hz, 1H, E), 4.36 (dd, *J* = 10.7, 6.5 Hz, 1H, E), 3.24 (s, 3H, Z), 3.23 (s, 3H, E), 1.69 (dd, *J* = 6.4, 1.1 Hz, 3H, E), 1.65 (d, *J* = 6.7 Hz, 3H, Z). **HRMS** (ESI): Calcd for $(M +Na)^+$ $[C_{22}H_{18}O_3NF_3Na]^+$: 424.1131, found: 424.1142.

(E)-1-benzyl-3-(but-2-en-1-yloxy)-3-(phenylethynyl)indolin-2-one (**1k**) was prepared

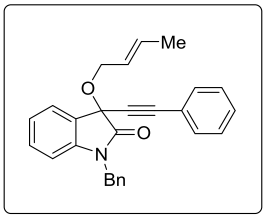

according to the general procedure C, by using **S9q** (200 mg, 0.59 mmol) and crotyl bromide. After silica gel column chromatography with EtOAc / petroleum ether = 1/8 (R_f = 0.33) as eluents the desired product was obtained in 90% yield (195 mg, 0.60 mmol, 76% (E)-isomer) as a brown oil. **^{1}H NMR** (500 MHz, $CDCl_3$) δ 7.57 (d, *J* = 7.1 Hz, 1H, E and Z), 7.49 (d, *J* = 6.5 Hz, 2H, E and Z), 7.46 – 7.17 (m, 9H, E and Z), 7.10 (t, *J* = 7.5 Hz, 1H, E and Z), 6.71 (d, *J* = 7.5 Hz, 1H, E and Z), 5.84 – 5.71 (m, 1H, E and Z), 5.71 – 5.62 (m, 1H, E and Z), 4.93 (s, 2H, E and Z), 4.56 (dd, *J* = 10.5, 5.0 Hz, 1H, Z), 4.51 (dd, *J* = 10.5, 5.0 Hz, 1H, Z), 4.43 (dd, *J* = 10.6, 6.4 Hz, 1H, E), 4.37 (dd, *J* = 10.6, 6.4 Hz, 1H, E), 1.70 (d, *J* = 6.2 Hz, 3H, E), 1.66 (d, *J* = 5.1 Hz, 3H, Z). **HRMS** (ESI): Calcd for $(M +Na)^+$ $[C_{27}H_{23}O_2NNa]^+$: 416.1621, found: 416.1637.

(E)-3-(but-2-en-1-yloxy)-1-(4-methoxybenzyl)-3-(phenylethynyl)indolin-2-one (**1l**) was

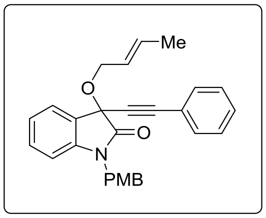

prepared according to the general procedure C, by using **S9r** (96 mg, 0.26 mmol) and crotyl bromide. After silica gel column chromatography with EtOAc / petroleum ether = 1/6 (R_f = 0.38) as eluents the desired product was obtained in 87% yield (96 mg, 0.23 mmol, 73% (E)-isomer) as a yellow oil. **^{1}H NMR** (500 MHz, $CDCl_3$) δ 7.55 (d, *J* = 7.5 Hz, 1H, E and Z), 7.48 (d, *J* = 6.5 Hz, 2H, E and Z), 7.36 – 7.19 (m, 6H, E and Z), 7.09 (t, *J* = 7.5 Hz, 1H, E and Z), 6.85 (d, *J* = 8.6 Hz, 2H, E and Z), 6.73 (d, *J* = 7.5 Hz, 1H, E and Z), 5.80 – 5.70 (m, 1H, E and Z), 5.70 – 5.60 (m, 1H, E and Z), 4.87 (s, 2H, E and Z), 4.52 (dd, *J* = 10.4, 5.5 Hz, 1H, Z), 4.48 (dd, *J* = 10.3, 5.5 Hz, 1H, Z), 4.39 (dd, *J* = 10.7, 6.4 Hz, 1H, E), 4.34 (dd, *J* = 10.7, 6.4 Hz, 1H, E), 3.77 (s, 3H, E and Z), 1.69 (d, *J* = 6.3 Hz, 3H, E), 1.65 (d, *J* = 5.4 Hz, 3H, Z). **HRMS** (ESI): Calcd for $(M +Na)^+$ $[C_{28}H_{25}O_3NNa]^+$: 446.1727, found: 446.1737.

(E)-3-((4-bromophenyl)ethynyl)-3-(but-2-en-1-yloxy)-1-methylindolin-2-one (**1m**) was

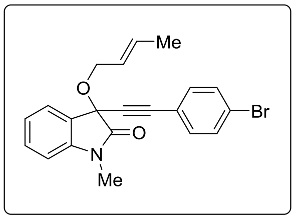

prepared according to the general procedure C, by using **S9v** (130 mg, 0.38 mmol) and crotyl bromide. After silica gel column chromatography with EtOAc / petroleum ether = 1/7 (R_f = 0.35) as eluents the desired product was obtained in 66% yield (100 mg, 0.25 mmol, 76% (E)-isomer) as a brown oil. **^{1}H NMR** (600 MHz, $CDCl_3$) δ 7.53 (d, *J* = 7.8 Hz, 1H, E and Z), 7.42 (d, *J* = 8.6 Hz, 2H, E and Z), 7.37 (td, *J* = 7.8, 1.2 Hz, 1H, E and Z), 7.31 (d, *J* = 8.6 Hz, 2H, E and Z), 7.14 (td, *J* = 7.8, 0.9 Hz, 1H, E and Z), 6.84 (d, *J* = 7.8 Hz, 1H, E and Z), 5.76 – 5.67 (m, 1H, E and Z), 5.67 – 5.57 (m, 1H, E and Z), 4.45 (dd, *J* = 11.0, 6.7 Hz, 1H, Z), 4.40 (dd, *J* = 11.0, 6.7 Hz, 1H, Z), 4.32 (dd, *J* = 10.6, 6.5 Hz, 1H, E), 4.24 (dd, *J* = 10.6, 6.5 Hz, 1H, E), 3.23 (s, 3H, Z), 3.22 (s, 3H, E), 1.67 (dd, *J* = 6.4, 1.2 Hz, 3H, E), 1.62 (dd, *J* = 6.7, 1.3 Hz, 3H, Z). **HRMS** (ESI): Calcd for $(M +Na)^+$ [C21H18O2NBrNa]$^+$: 418.0413, found: 418.0421.

(E)-3-(but-2-en-1-yloxy)-1,5,7-trimethyl-3-(phenylethynyl)indolin-2-one (**1n**) was prepared

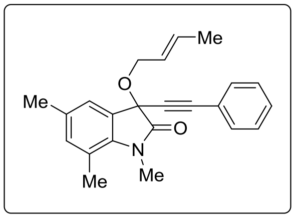

according to the general procedure C, by using **S9w** (200 mg, 0.69 mmol) and crotyl bromide. After silica gel column chromatography with EtOAc / petroleum ether = 1/5 (R_f = 0.38) as eluents, the desired product was obtained in 95% yield (225 mg, 0.65 mmol, 80% (E)-isomer) as a brown oil. **^{1}H NMR** (500 MHz, $CDCl_3$) δ 7.49 (d, *J* = 6.5 Hz, 2H, E and Z), 7.35 – 7.30 (m, 3H, E and Z), 7.23 (s, 1H, E and Z), 6.91 (s, 1H, E and Z), 5.81 – 5.70 (m, 1H, E and Z), 5.70 – 5.60 (m, 1H, E and Z), 4.53 (dd, *J* = 11.3, 6.1 Hz, 1H, Z), 4.49 (dd, *J* = 11.3, 6.1 Hz, 1H, Z), 4.33 (dd, *J* = 10.7, 6.4 Hz, 1H, E), 3.51 (s, 3H), 3.50 (s, 3H), 2.54 (s, 3H), 2.33 (s, 3H), 1.71 (d, *J* = 6.3 Hz, 3H), 1.68 (d, *J* = 6.1 Hz, 3H). **HRMS** (ESI): Calcd for $(M +Na)^+$ $[C_{23}H_{23}O_2NNa]^+$: 368.1621, found: 368.1632.

1-methyl-3-((3-methylbut-2-en-1-yl)oxy)-3-(phenylethynyl)indolin-2-one (**13a**) was prepared

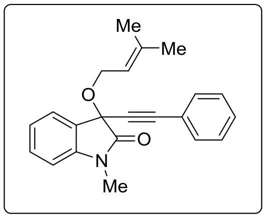

according to the general procedure C, by using **S9a** (79 mg, 0.30 mmol) and 3,3-dimethylallyl bromide. After silica gel column chromatography with EtOAc / petroleum ether = 1/7 (R_f = 0.32) as eluents the desired product was obtained in 93% yield (92 mg, 0.28 mmol) as a brown oil.**^{1}H NMR** (400 MHz, $CDCl_3$) δ 7.55 (d, *J* = 7.6 Hz, 1H), 7.46 (d, *J* = 6.3 Hz, 2H), 7.36 (t, *J* = 7.6 Hz, 1H), 7.30 - 7.26 (m, 3H), 7.13 (t, *J* = 7.6 Hz, 1H), 6.84 (d, *J* = 7.6 Hz, 1H), 5.39 (t, *J* = 7.1 Hz, 1H), 4.42 (dd, *J* = 10.4, 7.1 Hz, 1H), 4.35 (dd, *J* = 10.4, 7.1 Hz, 1H), 3.23 (s, 3H), 1.72 (s, 3H), 1.64 (s, 3H). **^{13}C NMR** (126 MHz, $CDCl_3$) δ 172.08, 143.41, 138.39, 132.26, 130.54, 129.04, 128.32, 128.27, 125.13, 123.56, 121.98, 120.51, 108.76, 87.80, 83.98, 74.26, 62.51, 26.60, 25.97, 18.22. **HRMS** (ESI): Calcd for $(M + H)^+$ $[C_{22}H_{22}O_2N]^+$: 332.1645, found: 332.1645.

1-methyl-3-((3-methylbut-2-en-1-yl)oxy)-3-(p-tolylethynyl)indolin-2-one (**13b**) was prepared

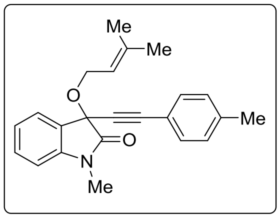

according to the general procedure C, by using **S9b** (100 mg, 0.36 mmol) and 3,3-dimethylallyl bromide. After silica gel column chromatography with EtOAc / petroleum ether = 1/7 (R_f = 0.33) as eluents the desired product was obtained in 90% yield (112 mg, 0.32 mmol) as a brown oil.

^{1}H NMR (500 MHz, $CDCl_3$) δ 7.55 (d, *J* = 7.5 Hz, 1H), 7.40 – 7.30 (m, 3H), 7.12 (t, *J* = 7.5 Hz, 1H), 7.08 (d, *J* = 7.7 Hz, 2H), 6.81 (d, *J* = 7.5 Hz, 1H), 5.39 (t, *J* = 6.5 Hz, 1H), 4.42 (dd, *J* = 8.0, 6.5 Hz, 1H), 4.36 (dd, *J* = 8.0, 6.5 Hz, 1H), 3.21 (s, 3H), 2.31 (s, 3H), 1.71 (s, 3H), 1.64 (s, 3H). **^{13}C NMR** (126 MHz, $CDCl_3$) δ 172.05, 143.30, 139.16, 138.13, 132.06, 130.40, 129.00, 128.28, 124.99, 123.42, 120.52, 118.82, 108.66, 87.95, 83.25, 74.22, 62.37, 26.45, 25.86, 21.53, 18.12. **HRMS** (ESI): Calcd for $(M + H)^+$ $[C_{23}H_{24}O_2N]^+$: 346.1802, found: 346.1804.

1-methyl-3-((3-methylbut-2-en-1-yl)oxy)-3-(m-tolylethynyl)indolin-2-one (**13c**) was prepared according to the general procedure C, by using **S9c** (131 mg, 0.47 mmol) and 3,3-dimethylallyl bromide. After silica gel column chromatography with EtOAc / petroleum ether = 1/7 (R_f = 0.29) as eluents the desired product was obtained in 78% yield (127 mg, 0.37 mmol) as a brown oil.

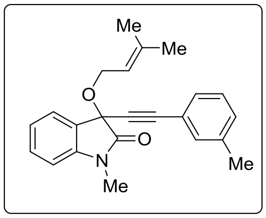

^{1}H NMR (500 MHz, cdcl3) δ 7.68 (dd, *J* = 7.6, 0.6 Hz, 1H), 7.48 (td, *J* = 7.6, 0.5 Hz, 1H), 7.46 – 7.37 (m, 2H), 7.30 (t, *J* = 7.6 Hz, 1H), 7.28 – 7.23 (m, 2H), 6.96 (d, *J* = 7.6 Hz, 1H), 5.52 (t, *J* = 7.4 Hz, 1H), 4.57 (dd, *J* = 10.5, 7.4 Hz, 1H), 4.50 (dd, *J* = 10.5, 7.4 Hz, 1H), 3.35 (s, 3H), 2.42 (s, 3H), 1.85 (s, 3H), 1.78 (s, 3H). **^{13}C NMR** (126 MHz, $CDCl_3$) δ 172.12, 143.39, 138.33, 138.02, 132.85, 130.50, 129.93, 129.30, 128.36, 128.22, 125.11, 123.54, 121.76, 120.56, 108.73, 88.06, 83.58, 74.25, 62.50, 26.57, 25.97, 21.25, 18.22. **HRMS** (ESI): Calcd for $(M + Na)^+$ $[C_{23}H_{23}O_2NNa]^+$: 368.1621, found: 368.1623.

3-((3,4-dimethoxyphenyl)ethynyl)-1-methyl-3-((3-methylbut-2-en-1-yl)oxy)indolin-2-one (**13d**) was prepared according to the general procedure C, by using **S9d** (223 mg, 0.69 mmol) and 3,3-dimethylallyl bromide. After silica gel column chromatography with EtOAc / petroleum ether = 1/1 (R_f = 0.59) as eluents the desired product was obtained in 94% yield (256 mg, 0.64 mmol) as a brown oil. **^{1}H NMR** (400 MHz, $CDCl_3$) δ 7.54 (dd, *J* = 7.4, 0.6 Hz, 1H), 7.33 (td, *J* = 7.8, 1.1 Hz, 1H), 7.11 (t, *J* = 7.5 Hz, 1H), 7.05 (dd, *J* = 8.3, 1.8 Hz, 1H), 6.94 (d, *J* = 1.8 Hz, 1H), 6.81 (d, *J* = 7.8 Hz, 1H), 6.74 (d, *J* = 8.4 Hz, 1H), 5.37 (t, *J* = 7.2 Hz, 1H), 4.42 – 4.32 (m, 1H), 4.32 – 4.23 (m, 1H), 3.83 (s, 3H), 3.81 (s, 3H), 3.20 (s, 3H), 1.69 (s, 3H), 1.61 (s, 3H). **^{13}C NMR** (101 MHz, $CDCl_3$) δ 172.05, 149.92, 148.42, 143.23, 138.28, 130.41, 128.04, 125.66, 124.95,

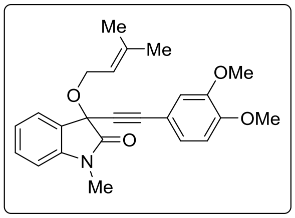

123.44, 120.33, 114.66, 113.87, 110.75, 108.68, 87.72, 82.36, 74.22, 62.25, 55.92, 55.85, 26.47, 25.87, 18.10. **HRMS** (ESI): Calcd for $(M + Na)^+$ $[C_{24}H_{25}O_4NNa]^+$: 414.1676, found: 414.1688.

3-((2-methoxyphenyl)ethynyl)-1-methyl-3-((3-methylbut-2-en-1-yl)oxy)indolin-2-one (**13e**)

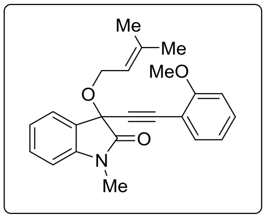

was prepared according to the general procedure C, by using **S9e** (100 mg, 0.34 mmol) and 3,3-dimethylallyl bromide. After silica gel column chromatography with EtOAc / petroleum ether = 1/7 (R_f = 0.16) as eluents the desired product was obtained in 61% yield (75 mg, 0.21 mmol) as a brown oil. **^{1}H NMR** (500 MHz, $cdcl_3$) δ 7.57 (d, *J* = 7.6 Hz, 1H), 7.40 (dd, *J* = 7.6, 1.5 Hz, 1H), 7.33 (td, *J* = 7.6, 0.5 Hz, 1H), 7.31 – 7.26 (m, 1H), 7.11 (t, *J* = 7.6 Hz, 1H), 6.90 – 6.78 (m, 3H), 5.41 (t, *J* = 7.3 Hz, 1H), 4.55 (dd, *J* = 10.4, 7.3 Hz, 1H), 4.48 (dd, *J* = 10.4, 7.3 Hz, 1H), 3.83 (s, 3H), 3.21 (s, 3H), 1.72 (s, 3H), 1.67 (s, 3H). **^{13}C NMR** (126 MHz, $cdcl_3$) δ 172.10, 160.79, 143.41, 138.14, 133.99, 130.47, 130.39, 128.61, 125.23, 123.43, 120.74, 120.36, 111.35, 110.86, 108.64, 87.64, 84.76, 74.33, 62.49, 55.87, 26.53, 25.97, 18.20. **HRMS** (ESI): Calcd for $(M + Na)^+$ $[C_{23}H_{24}O_3NNa]^+$: 384.1570, found: 384.1574.

3-((4-fluorophenyl)ethynyl)-1-methyl-3-((3-methylbut-2-en-1-yl)oxy)indolin-2-one (**13f**) was

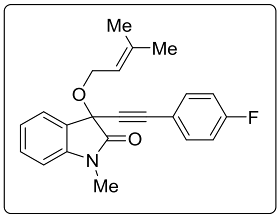

prepared according to the general procedure C, by using **S9f** (100 mg, 0.36 mmol) and 3,3-dimethylallyl bromide. After silica gel column chromatography with EtOAc / petroleum ether = 1/7 (R_f = 0.37) as eluents, the desired product was obtained in 74% yield (92 mg, 0.26 mmol) as a brown oil. **^{1}H NMR** (500 MHz, $cdcl_3$) δ 7.54 (dd, *J* = 7.6, 0.8 Hz, 1H), 7.48 – 7.40 (m, 2H), 7.36 (td, *J* = 7.6, 0.8 Hz, 1H), 7.13 (td, *J* = 7.6, 0.8 Hz, 1H), 6.97 (t, *J* = 8.8 Hz, 2H), 6.84 (d, *J* = 7.6 Hz, 1H), 5.41 – 5.35 (m, 1H), 4.37 (dd, *J* = 10.5, 7.3 Hz, 1H), 4.31 (dd, *J* = 10.5, 7.1 Hz, 1H), 3.22 (s, 3H), 1.71 (s, 3H), 1.63 (s, 3H). **^{13}C NMR** (126 MHz, $cdcl_3$) δ 171.99, 162.97 (d, *J* = 250.5 Hz), 143.39, 138.45, 134.24 (d, *J* = 8.5 Hz), 130.60, 128.05, 125.07, 123.58, 120.40, 118.03 (d, *J* = 3.5 Hz), 115.66 (d, *J* = 22.1 Hz), 108.80, 86.61, 83.81, 74.23, 62.47, 26.59, 25.95, 18.19. **HRMS** (ESI): Calcd for $(M + Na)^+$ $[C_{22}H_{20}O_2NFNa]^+$: 372.1370, found: 372.1376.

1-methyl-3-((3-methylbut-2-en-1-yl)oxy)-3-(thiophen-3-ylethynyl)indolin-2-one (**13g**) was

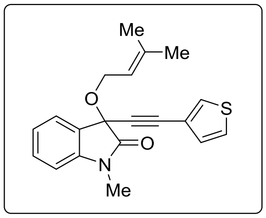

prepared according to the general procedure C, by using **S9g** (50 mg, 0.19 mmol) and 3,3-dimethylallyl bromide. After silica gel column chromatography with EtOAc / petroleum ether = 1/6 (R_f = 0.43) as eluents the desired product was obtained in 83% yield (52 mg, 0.15 mmol) as a brown oil. **^{1}H NMR** (500 MHz, $CDCl_3$) δ 7.54 (d, *J* = 7.6 Hz, 1H), 7.52 – 7.47 (m, 1H), 7.36 (td, *J* = 7.6, 1.1 Hz, 1H), 7.22 (dd, *J* = 5.0, 3.0 Hz, 1H), 7.17 – 7.07 (m, 2H), 6.83 (d, *J* = 7.6 Hz, 1H), 5.38 (t, *J* = 7.3 Hz, 1H), 4.39 (dd, *J* = 10.4, 7.3 Hz, 1H), 4.32 (dd, *J* = 10.4, 7.3 Hz, 1H), 3.22 (s, 3H), 1.71 (s, 3H), 1.63 (s, 3H). **^{13}C NMR** (126 MHz, $CDCl_3$) δ 172.05, 143.40, 138.39, 130.55, 130.42, 130.20, 128.16, 125.37, 125.13, 123.56, 121.05, 120.49, 108.77, 83.66, 82.95, 74.31, 62.50, 26.60, 25.97, 18.22. **HRMS** (ESI): Calcd for $(M + Na)^+$ $[C_{20}H_{19}O_2NSNa]^+$: 360.1029, found: 360.1045.

1-methyl-3-((3-methylbut-2-en-1-yl)oxy)-3-(pent-1-yn-1-yl)indolin-2-one (**13h**) was prepared

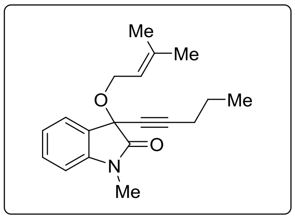

according to the general procedure C, by using **S9h** (48 mg, 0.21 mmol) and 3,3-dimethylallyl bromide. After silica gel column chromatography with EtOAc / petroleum ether = 1/2 (R_f = 0.80) as eluents the desired product was obtained in 99% yield (61 mg, 0.21 mmol) as a yellow oil.
^{1}H NMR (500 MHz, $cdcl_3$) δ 7.46 (dd, *J* = 7.6, 0.8 Hz, 1H), 7.31 (td, *J* = 7.6, 1.2 Hz, 1H), 7.09 (td, *J* = 7.6, 0.8 Hz, 1H), 6.79 (d, *J* = 7.6 Hz, 1H), 5.34 (t, *J* = 7.2 Hz, 1H), 4.31 (dd, *J* = 10.5, 7.2 Hz, 1H), 4.25 (dd, *J* = 10.5, 7.2 Hz, 1H), 3.18 (s, 3H), 2.22 (td, *J* = 7.2, 1.9 Hz, 2H), 1.69 (s, 3H), 1.61 (s, 3H),1.53 (qt, *J* = 7.2, 7.2 Hz, 2H),, 0.95 (t, *J* = 7.2 Hz, 3H). **^{13}C NMR** (126 MHz, $cdcl_3$) δ 172.43, 143.30, 138.01, 130.24, 128.69, 124.84, 123.39, 120.61, 108.59, 89.21, 75.19, 73.90, 62.16, 26.45, 25.92, 21.87, 21.05, 18.13, 13.60. **HRMS** (ESI): Calcd for $(M + H)^+$ $[C_{19}H_{24}O_2N]^+$: 298.1802, found: 298.1807.

1-methyl-3-((3-methylbut-2-en-1-yl)oxy)-3-(5-methylhex-1-yn-1-yl)indolin-2-one (**13i**) was

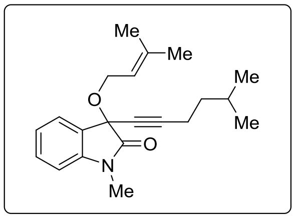

prepared according to the general procedure C, by using **S9i** (64 mg, 0.25 mmol) and 3,3-dimethylallyl bromide. After silica gel column chromatography with EtOAc / petroleum ether = 1/2 (R_f = 0.8) as eluents the desired product was obtained in 86% yield (70 mg, 0.22 mmol) as a yellow oil. **^{1}H NMR** (500 MHz, cdcl$_3$) δ 7.45 (dd, *J* = 7.4, 0.7 Hz, 1H), 7.31 (td, *J* = 7.8, 1.2 Hz, 1H), 7.09 (td, *J* = 7.6, 0.8 Hz, 1H), 6.79 (d, *J* = 7.8 Hz, 1H), 5.40 – 5.30 (m, 1H), 4.31 (dd, *J* = 10.5, 7.3 Hz, 1H), 4.24 (dd, *J* = 10.5, 7.1 Hz, 1H), 3.18 (s, 3H), 2.24 (td, *J* = 7.4, 1.6 Hz, 2H), 1.69 (s, 3H), 1.67 – 1.61 (m, 1H), 1.61 (s, 3H), 1.41 (td, *J* = 7.4, 7.4 Hz, 2H), 0.86 (d, *J* = 2.3 Hz, 3H), 0.85 (d, *J* = 2.3 Hz, 3H). **^{13}C NMR** (126 MHz, cdcl$_3$) δ 172.42, 143.29, 137.98, 130.23, 128.68, 124.84, 123.38, 120.62, 108.58, 89.47, 74.84, 73.90, 62.15, 37.33, 27.44, 26.45, 25.91, 22.22, 18.13, 17.12. **HRMS** (ESI): Calcd for $(M + Na)^+$ $[C_{21}H_{27}O_2NNa]^+$: 348.1934, found: 348.1949.

1,5-dimethyl-3-((3-methylbut-2-en-1-yl)oxy)-3-(phenylethynyl)indolin-2-one (**13j**) was

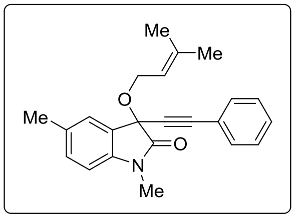

prepared according to the general procedure C, by using **S9j** (44 mg, 0.16 mmol) and 3,3-dimethylallyl bromide. After silica gel column chromatography with EtOAc / petroleum ether = 1/4 (R_f = 0.59) as eluents the desired product was obtained in 82% yield (45 mg, 0.13 mmol) as a brown oil. **^{1}H NMR** (300 MHz, CDCl$_3$) δ 7.50 – 7.39 (m, 2H), 7.33 (s, 1H), 7.29 – 7.18 (m, 3H), 7.11 (d, *J* = 7.9 Hz, 1H), 6.68 (d, *J* = 7.9 Hz, 1H), 5.36 (t, *J* = 7.2 Hz, 1H), 4.41 (d, *J* = 10.5, 7.2 Hz, 1H), 4.31 (d, *J* = 10.5, 7.2 Hz, 1H), 3.16 (s, 3H), 2.32 (s, 3H), 1.68 (s, 3H), 1.62 (s, 3H). **^{13}C NMR** (75 MHz, CDCl$_3$) δ 171.99, 140.92, 138.34, 133.22, 132.20, 130.74, 128.97, 128.28, 128.13, 125.78, 121.96, 120.51, 108.49, 87.70, 84.06, 74.30, 62.41, 26.56, 25.96, 21.17, 18.19. **HRMS** (ESI): Calcd for $(M +Na)^+$ [C23H23O2NNa]$^+$: 368.1621, found: 368.1637.

5-methoxy-1-methyl-3-((3-methylbut-2-en-1-yl)oxy)-3-(phenylethynyl)indolin-2-one (**13k**)

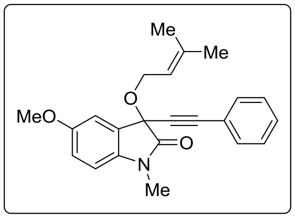

was prepared according to the general procedure C, by using **S9k** (97 mg, 0.33 mmol) and 3,3-dimethylallyl bromide. After silica gel column chromatography with EtOAc / petroleum ether = 1/4 (R_f = 0.35) as eluents the desired product was obtained in 74% yield (89 mg, 0.25 mmol) as a brown oil. ^{1}**H NMR** (300 MHz, $CDCl_3$) δ 7.51 – 7.37 (m, 2H), 7.31 – 7.19 (m, 3H), 7.14 (d, *J* = 2.5 Hz, 1H), 6.85 (dd, *J* = 8.5, 2.5 Hz, 1H), 6.71 (d, *J* = 8.5 Hz, 1H), 5.37 (t, *J* = 7.5 Hz, 1H), 4.37 (dd, *J* = 10.5, 7.5 Hz, 1H), 4.30 (dd, *J* = 10.5, 7.5 Hz, 1H), 3.77 (s, 3H), 3.16 (s, 3H), 1.68 (s, 3H), 1.62 (s, 3H). ^{13}**C NMR** (75 MHz, $CDCl_3$) δ 171.65, 156.56, 138.31, 136.55, 132.07, 129.01, 128.92, 128.18, 121.72, 120.27, 115.10, 111.78, 109.16, 87.63, 83.85, 74.42, 62.32, 55.85, 26.49, 25.82, 18.08. **HRMS** (ESI): Calcd for $(M + H)^+$ $[C_{23}H_{24}O_3N]^+$: 362.1750, found: 362.1760.

6-bromo-1-methyl-3-((3-methylbut-2-en-1-yl)oxy)-3-(phenylethynyl)indolin-2-one (**13l**) was

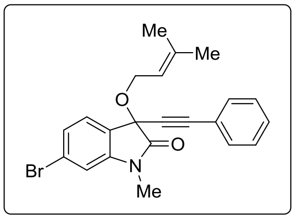

prepared according to the general procedure C, by using **S9l** (117 mg, 0.34 mmol) and 3,3-dimethylallyl bromide. After silica gel column chromatography with EtOAc / petroleum ether = 1/4 (R_f = 0.91) as eluents the desired product was obtained in 46% yield (65 mg, 0.16 mmol) as a brown oil. ^{1}**H NMR** (300 MHz, $CDCl_3$) δ 7.45 – 7.38 (m, 2H), 7.36 (d, *J* = 7.9 Hz, 1H), 7.31 – 7.18 (m, 4H), 6.94 (d, *J* = 1.6 Hz, 1H), 5.33 (t, *J* = 7.2 Hz, 1H), 4.39 (dd, *J* = 10.2, 7.2 Hz, 1H), 4.30 (dd, *J* = 10.2, 7.2 Hz, 1H), 3.16 (s, 3H), 1.67 (s, 3H), 1.60 (s, 3H). ^{13}**C NMR** (75 MHz, $CDCl_3$) δ 171.81, 144.60, 138.74, 132.20, 129.19, 128.35, 127.13, 126.35, 124.26, 121.63, 120.19, 112.31, 88.18, 83.19, 73.74, 62.54, 29.80, 26.67, 25.96, 18.21. **HRMS** (ESI): Calcd for $(M + Na)^+$ $[C_{22}H_{20}O_2NBrNa]^+$: 432.0570, found: 432.0571.

5-fluoro-1-methyl-3-((3-methylbut-2-en-1-yl)oxy)-3-(phenylethynyl)indolin-2-one (**13m**) was

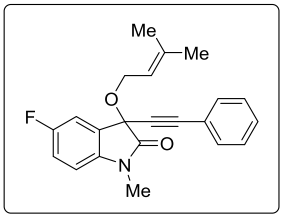

prepared according to the general procedure C, by using **S9m** (40 mg, 0.14 mmol) and 3,3-dimethylallyl bromide. After silica gel column chromatography with EtOAc / petroleum ether = 1/4 (R_f = 0.45) as eluents the desired product was obtained in 99% yield (49 mg, 0.14

mmol) as a yellow oil. **^{1}H NMR** (300 MHz, $CDCl_3$) δ 7.56 – 7.36 (m, 2H), 7.33 – 7.17 (m, 4H), 7.02 (td, *J* = 8.6, 2.6 Hz, 1H), 6.72 (dd, *J* = 8.6, 4.0 Hz, 1H), 5.34 (t, *J* = 7.4 Hz, 1H), 4.42 (dd, *J* = 10.3, 7.4 Hz, 1H), 4.34 (dd, *J* = 10.3, 7.4 Hz, 1H), 3.17 (s, 3H), 1.68 (s, 3H), 1.62 (s, 3H). **^{13}C NMR** (75 MHz, $CDCl_3$) δ 171.78, 159.68 (d, *J* = 242.5 Hz), 139.24 (d, *J* = 2.0 Hz), 138.77, 132.22, 129.67 (d, *J* = 7.9 Hz), 129.21, 128.35, 121.60, 120.19, 116.78 (d, *J* = 23.6 Hz), 113.18 (d, *J* = 25.1), 88.29, 83.22, 77.36, 74.12, 62.59, 26.67, 25.94, 18.19. **HRMS** (ESI): Calcd for $(M + Na)^+$ $[C_{22}H_{20}O_2NFNa]^+$: 372.1370, found: 372.1375.

1-methyl-3-((3-methylbut-2-en-1-yl)oxy)-3-(phenylethynyl)-5-(trifluoromethoxy)indolin-2-one (**13n**) was prepared according to the general procedure C, by using **S9n** (102 mg, 0.29 mmol) and 3,3-dimethylallyl bromide. After silica gel column chromatography with EtOAc / petroleum ether = 1/4 (R_f = 0.57) as eluents the desired product was obtained in 59% yield (72 mg, 0.17 mmol) as a brown oil. **^{1}H NMR** (300 MHz, $CDCl_3$) δ 7.54 – 7.36 (m, 3H), 7.34 – 7.14 (m, 4H), 6.80 (d, *J* = 8.5 Hz, 1H), 5.36 (t, *J* = 7.3 Hz, 1H), 4.44 (dd, *J* = 10.1, 7.3 Hz, 1H), 4.36 (dd, *J* = 10.1, 7.3 Hz, 1H), 3.19 (s, 3H), 1.69 (s, 3H), 1.62 (s, 3H). **^{13}C NMR** (75 MHz, $CDCl_3$) δ 171.83, 145.33 (d, *J* = 1.9 Hz), 141.94, 138.96, 132.24, 129.66, 129.30, 128.39, 123.60, 121.51, 120.62 (q, *J* = 257.1 Hz), 120.09, 119.07, 88.55, 82.93, 73.95, 62.67, 29.80, 26.70, 25.94, 18.13. **HRMS** (ESI): Calcd for $(M + Na)^+$ $[C_{23}H_{20}O_3NF_3Na]^+$: 438.1288, found: 438.11289.

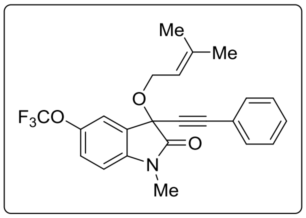

1-methyl-3-((3-methylbut-2-en-1-yl)oxy)-5-nitro-3-(phenylethynyl)indolin-2-one (**13o**) was prepared according to the general procedure C, by using **S9o** (40 mg, 0.13 mmol) and 3,3-dimethylallyl bromide. After silica gel column chromatography with EtOAc / petroleum ether = 1/4 (R_f = 0.26) as eluents the desired product was obtained in 90% yield (44 mg, 0.12 mmol) as a yellow oil. **^{1}H NMR** (300 MHz, $CDCl_3$) δ 8.43 (d, *J* = 2.3 Hz, 1H), 8.33 (dd, *J* = 8.6, 2.3 Hz, 1H), 7.59 – 7.44 (m, 2H), 7.42 – 7.28 (m, 3H), 6.93 (d, *J* = 8.7 Hz, 1H), 5.39 (t, *J* = 7.2 Hz, 1H), 4.59 (dd, *J* = 10.4, 7.5 Hz, 1H), 4.51 (dd, *J* = 10.4, 7.2 Hz, 1H), 3.29 (s, 3H), 1.74 (s, 3H), 1.70 (s, 3H). **^{13}C NMR** (75 MHz, $CDCl_3$) δ 172.07, 148.73, 144.10, 139.36, 132.28, 129.55,

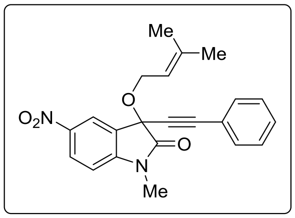

129.35, 128.48, 127.44, 121.08, 119.88, 108.52, 89.44, 81.97, 73.21, 62.93, 29.80, 26.98, 25.98, 18.26. **HRMS** (ESI): Calcd for $(M + H)^+$ $[C_{22}H_{21}O_4N_2]^+$: 377.1496, found: 377.1501.

5-chloro-1-methyl-3-((3-methylbut-2-en-1-yl)oxy)-3-(phenylethynyl)indolin-2-one (**13p**) was prepared according to the general procedure C, by using **S9p** (37 mg, 0.12 mmol) and 3,3-dimethylallyl bromide. After silica gel column chromatography with EtOAc / petroleum ether = 1/4 (R_f = 0.49) as eluents the desired product was obtained in 99% yield (45 mg, 0.12 mmol) as a yellow oil. **^{1}H NMR** (300 MHz, $CDCl_3$) δ 7.53 (d, *J* = 2.1 Hz, 1H), 7.50 – 7.43 (m, 2H), 7.38 – 7.20 (m, 4H), 6.76 (d, *J* = 8.3 Hz, 1H), 5.39 (t, *J* = 7.2 Hz, 1H), 4.48 (dd, *J* = 10.3, 7.3 Hz, 1H), 4.41 (dd, *J* = 10.3, 7.3 Hz, 1H), 3.21 (s, 3H), 1.73 (s, 3H), 1.67 (s, 3H). **^{13}C NMR** (75 MHz, $CDCl_3$) δ 171.60, 141.82, 138.85, 132.23, 130.39, 129.80, 129.25, 128.91, 128.37, 125.55, 121.57, 120.17, 109.75, 88.47, 83.06, 73.92, 62.63, 26.66, 25.96, 18.22. **HRMS** (ESI): Calcd for $(M + Na)^+$ $[C_{22}H_{20}O_2NClNa]^+$: 388.1075, found: 388.1087.

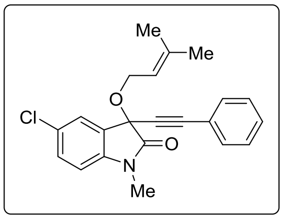

1-benzyl-3-((3-methylbut-2-en-1-yl)oxy)-3-(phenylethynyl)indolin-2-one (**13q**) was prepared according to the general procedure C, by using **S9q** (47 mg, 0.14 mmol) and 3,3-dimethylallyl bromide. After silica gel column chromatography with EtOAc / petroleum ether = 1/4 (R_f = 0.69) as eluents the desired product was obtained in 90% yield (51 mg, 0.13 mmol) as a brown oil. **^{1}H NMR** (300 MHz, $CDCl_3$) δ 7.57 (d, *J* = 6.6 Hz, 1H), 7.53 – 7.44 (m, 2H), 7.38 – 7.17 (m, 9H), 7.10 (t, *J* = 7.6 Hz, 1H), 6.71 (d, *J* = 7.6 Hz, 1H), 5.44 (t, *J* = 7.5 Hz, 1H), 4.96 (d, *J* = 15.8 Hz, 1H), 4.90 (d, *J* = 15.8 Hz, 1H), 4.46 (dd, *J* = 10.4, 7.5 Hz, 1H), 4.40 (dd, *J* = 10.4, 7.5 Hz, 1H), 1.74 (s, 3H), 1.68 (s, 3H). **^{13}C NMR** (75 MHz, $CDCl_3$) δ 172.18, 142.41, 138.58, 135.38, 132.23, 130.40, 129.05, 128.94, 128.32, 128.21, 127.82, 127.30, 125.14, 123.59, 121.91, 120.40, 109.79, 87.89, 83.94, 74.30, 62.44, 44.02, 25.96, 18.20. **HRMS** (ESI): Calcd for $(M + Na)^+$ $[C_{28}H_{25}O_2NNa]^+$: 430.1778, found: 430.1792.

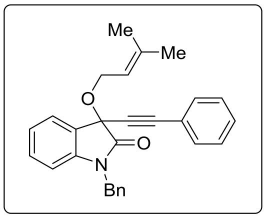

1-(4-methoxybenzyl)-3-((3-methylbut-2-en-1-yl)oxy)-3-(phenylethynyl)indolin-2-one (**13r**)

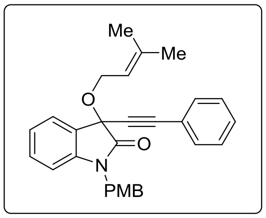

was prepared according to the general procedure C, by using **S9r** (50 mg, 0.14 mmol) and 3,3-dimethylallyl bromide. After silica gel column chromatography with EtOAc / petroleum ether = 1/4 (R_f = 0.60) as eluents the desired product was obtained in 90% yield (53 mg, 0.12 mmol) as a transparent oil. **^{1}H NMR** (300 MHz, $CDCl_3$) δ 7.57 (dd, *J* = 7.4, 0.8 Hz, 1H), 7.53 – 7.45 (m, 2H), 7.41 – 7.19 (m, 6H), 7.10 (t, *J* = 7.4 Hz, 1H), 6.85 (d, *J* = 8.7 Hz, 2H), 6.74 (d, *J* = 7.8 Hz, 1H), 5.44 (t, *J* = 7.6 Hz, 1H), 4.90 (d, *J* = 15.5 Hz, 1H), 4.84 (d, *J* = 15.5 Hz, 1H), 4.44 (dd, *J* = 10.4, 7.6 Hz, 1H), 4.39 (dd, *J* = 10.4, 7.6 Hz, 1H), 3.77 (s, 3H), 1.75 (s, 3H), 1.68 (s, 3H). **^{13}C NMR** (75 MHz, $CDCl_3$) δ 172.12, 159.23, 142.45, 138.52, 132.23, 130.37, 129.03, 128.74, 128.31, 128.22, 127.44, 125.11, 123.52, 121.93, 120.42, 114.31, 109.81, 87.81, 84.00, 74.31, 62.40, 55.33, 43.53, 25.95, 18.20. **HRMS** (ESI): Calcd for $(M + Na)^+$ $[C_{29}H_{27}O_3NNa]^+$: 460.1883, found: 460.1878.

1-(methoxymethyl)-3-((3-methylbut-2-en-1-yl)oxy)-3-(phenylethynyl)indolin-2-one (**13s**)

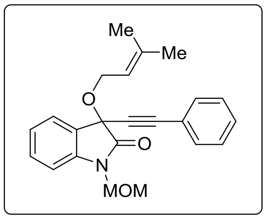

was prepared according to the general procedure C, by using **S9s** (191 mg, 0.65 mmol) and 3,3-dimethylallyl bromide. After silica gel column chromatography with EtOAc / petroleum ether = 1/5 (R_f = 0.75) as eluents the desired product was obtained in 89% yield (210 mg, 0.58 mmol) as a yellow oil. **^{1}H NMR** (300 MHz, $CDCl_3$) δ 7.58 (dd, *J* = 7.5, 0.8 Hz, 1H), 7.50 – 7.42 (m, 2H), 7.36 (td, *J* = 7.8, 1.3 Hz, 1H), 7.32 – 7.27 (m, 4H), 7.17 (td, *J* = 7.6, 0.9 Hz, 1H), 7.04 (d, *J* = 7.8 Hz, 1H), 5.41 (t, *J* = 7.7 Hz, 1H), 5.14 (s, 2H), 4.43 (dd, *J* = 10.4, 7.7 Hz, 1H), 4.35 (dd, *J* = 10.5, 7.7 Hz, 1H), 3.37 (s, 3H), 1.72 (s, 3H), 1.65 (s, 3H). **^{13}C NMR** (75 MHz, $CDCl_3$) δ 172.53, 141.53, 138.55, 132.13, 130.59, 129.06, 128.28, 127.72, 125.17, 124.00, 121.74, 120.26, 110.20, 88.07, 83.71, 74.45, 71.73, 62.41, 56.41, 25.88, 18.11. **HRMS** (ESI): Calcd for $(M + Na)^+$ $[C_{23}H_{23}O_3NNa]^+$: 384.1570, found: 384.1585.

3-((3-methylbut-2-en-1-yl)oxy)-3-(phenylethynyl)-1-((2-(trimethylsilyl)ethoxy)methyl)

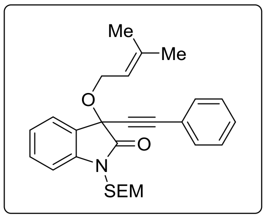

indolin-2-one (**13t**) was prepared according to the general procedure C, by using **S9t** (57 mg, 0.15 mmol) and 3,3-dimethylallyl bromide. After silica gel column chromatography with EtOAc / petroleum ether = 1/7 (R_f = 0.63) as eluents the desired product was obtained in 76% yield (51 mg, 0.11 mmol) as a brown oil. **^{1}H NMR** (500 MHz, $CDCl_3$) δ 7.57 (d, *J* = 7.5 Hz, 1H), 7.48 – 7.42 (m, 2H), 7.36 (td, *J* = 7.8, 1.2 Hz, 1H), 7.34 – 7.26 (m, 3H), 7.16 (td, *J* = 7.6, 0.9 Hz, 1H), 7.07 (d, *J* = 7.8 Hz, 1H), 5.45 – 5.32 (m, 1H), 5.19 (d, *J* = 11.1 Hz, 1H), 5.16 (d, *J* = 11.1 Hz, 1H), 4.42 – 4.35 (m, 1H), 4.35 – 4.29 (m, 1H), 3.62 (d, *J* = 7.9 Hz, 1H), 3.60 (d, *J* = 7.8 Hz, 1H), 1.72 (s, 3H), 1.64 (s, 3H), 0.96 – 0.89 (m, 2H), -0.05 (s, 9H). **^{13}C NMR** (75 MHz, $CDCl_3$) δ 172.45, 141.78, 138.57, 132.23, 130.59, 129.07, 128.31, 127.81, 125.17, 123.97, 121.87, 120.33, 110.38, 88.02, 83.83, 74.54, 69.81, 66.30, 62.44, 25.95, 18.19, 17.85, -1.37. **HRMS** (ESI): Calcd for $(M + Na)^+$ $[C_{27}H_{33}O_3NSiNa]^+$: 470.2122, found: 470.2137.

3-((3-methylbut-2-en-1-yl)oxy)-3-(phenylethynyl)indolin-2-one (13u) was prepared according

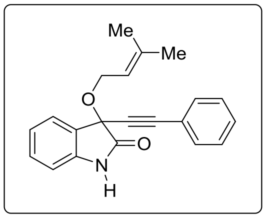

to the general procedure D, by using **13t** (120 mg, 0.27 mmol) as starting material. After silica gel column chromatography with EtOAc / petroleum ether = 1/4 (R_f = 0.21) as eluents, the desired product was obtained in 40% yield (34 mg, 0.11 mmol) as a brown oil. **^{1}H NMR** (500 MHz, $CDCl_3$) δ 8.94 (s, 1H), 7.54 (d, *J* = 7.6 Hz, 1H), 7.46 (dd, *J* = 8.1, 1.5 Hz, 2H), 7.35 – 7.24 (m, 4H), 7.11 (td, *J* = 7.6, 0.8 Hz, 1H), 6.95 (d, *J* = 7.6 Hz, 1H), 5.41 (t, *J* = 7.2 Hz, 1H), 4.37 (dd, *J* = 10.2, 7.2 Hz, 1H), 4.31 (dd, *J* = 10.5, 7.2 Hz, 1H), 1.72 (s, 3H), 1.64 (s, 3H). **^{13}C NMR** (126 MHz, $CDCl_3$) δ 174.49, 140.66, 138.59, 132.25, 130.59, 129.07, 128.57, 128.32, 125.46, 123.55, 121.90, 120.38, 110.88, 87.88, 83.81, 74.78, 62.48, 25.93, 18.19. **HRMS** (ESI): Calcd for $(M +Na)^+$ [C21H19O2NNa]$^+$: 340.1308, found: 340.1322.

Gold catalyzed single cleavage rearrangement

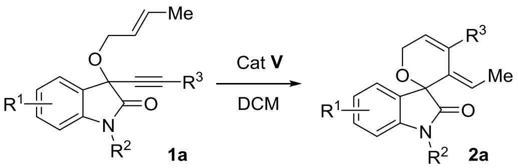

At 0 °C, to a DCM (0.6 ml) solution of 1,6-enyne (0.1 mmol) was added a solution of cat. **V** (5.9 mg, 5 µmol) in DCM (0.4 ml). After warming to room temperature, the reaction mixture was stirred overnight and then passed through a short pad of silica gel (Et_2O as the eluent). The resulting solution was concentrated under reduced pressure, followed by silica gel column chromatography (EA / PE as the eluent) to obtain the desired product.

(E)-3'-ethylidene-1-methyl-4'-phenyl-3',6'-dihydrospiro[indoline-3,2'-pyran]-2-one (**2a**) was

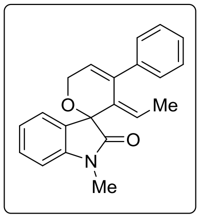

prepared according to the general procedure for the gold catalyzed *C*-migration reaction, by using **1a** (30 mg, 0.09 mmol, 82% (E)-isomer) as starting material. After the purification done by silica gel column chromatography with EtOAc / petroleum ether = 1/7 (R_f = 0.20) as eluents, the desired product was obtained in 60% yield (18 mg, 0.06 mmol) as a yellow oil. **^{1}H NMR** (500 MHz, $CDCl_3$) δ 7.45 (d, *J* = 7.6 Hz, 1H), 7.40 (td, *J* = 7.6, 1.1 Hz, 1H), 7.38 – 7.33 (m, 2H), 7.33 – 7.28 (m, 1H), 7.25 (dd, *J* = 8.1, 1.3 Hz, 2H), 7.09 (td, *J* = 7.6, 1.1 Hz, 1H), 6.95 (d, *J* = 7.6 Hz, 1H), 6.11 (s, 1H), 5.49 (q, *J* = 7.3Hz, 1H), 4.87 (dd, *J* = 17.8, 2.9 Hz, 1H), 4.65 (dd, *J* = 17.8, 3.1 Hz, 1H), 3.30 (s, 3H), 1.19 (d, *J* = 7.3 Hz, 3H). **^{13}C NMR** (126 MHz, $CDCl_3$) δ 174.34, 143.80, 141.86, 136.54, 130.84, 129.76, 129.40, 128.48, 128.39, 127.23, 127.17, 125.95, 124.84, 122.96, 108.73, 79.61, 63.77, 26.35, 16.34. **HRMS** (ESI): Calcd for $(M + Na)^+$ $[C21H19O2NNa]^+$: 340.1308, found: 340.1321.

(E)-3'-ethylidene-1-methyl-4'-(p-tolyl)-3',6'-dihydrospiro[indoline-3,2'-pyran]-2-one (**2b**)

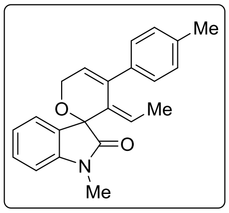

was prepared according to the general procedure for the gold catalyzed *C*-migration reaction, by using **1b** (40 mg, 0.12 mmol, 77% (E)-isomer) as starting material. After the purification done by silica gel column chromatography with EtOAc / petroleum ether = 1/5 (R_f = 0.35) as eluents, the desired product was obtained in 60% yield (24 mg, 0.07 mmol) as a yellow oil. **^{1}H NMR** (500 MHz, $CDCl_3$) δ 7.40 (d, *J* = 7.5 Hz, 1H), 7.35 (t, *J* = 7.7 Hz, 1H), 7.12 (d, *J* = 8.1 Hz, 2H),

7.09 (d, *J* = 8.1 Hz, 2H), 7.03 (t, *J* = 7.5 Hz, 1H), 6.89 (d, *J* = 7.5 Hz, 1H), 6.04 (s, 1H), 5.44 (q, *J* = 7.3 Hz, 1H), 4.80 (dd, *J* = 17.7, 3.0 Hz, 1H), 4.59 (dd, *J* = 17.7, 3.0 Hz, 1H), 3.26 (s, 3H), 2.35 (s, 3H), 1.16 (d, *J* = 7.3 Hz, 3H). **^{13}C NMR** (126 MHz, $CDCl_3$) δ 174.45, 143.84, 139.01, 136.96, 136.57, 131.02, 129.75, 129.56, 129.21, 127.91, 127.15, 125.91, 124.87, 122.99, 108.76, 79.74, 63.86, 26.42, 21.29, 16.45. **HRMS** (ESI): Calcd for $(M +Na)^+$ $[C_{22}H_{21}O_2NNa]^+$: 354.1465, found: 354.1480.

(E)-3'-ethylidene-4'-(4-fluorophenyl)-1-methyl-3',6'-dihydrospiro[indoline-3,2'-pyran]-2-one (**2c**) was prepared according to the general procedure for the gold catalyzed *C*-migration reaction, by using **1c** (49 mg, 0.15 mmol, 78% (E)-isomer) as starting material. After the purification done by silica gel column chromatography with EtOAc / petroleum ether = 1/7 (R_f = 0.18) as eluents, the desired product was obtained in 51% yield (25 mg, 0.07 mmol) as a yellow oil. **^{1}H NMR** (500 MHz, $CDCl_3$) δ 7.41 – 7.31 (m, 1H), 7.17 (dd, *J* = 8.7, 5.5 Hz, 1H), 7.05 (t, *J* = 7.5 Hz, 1H), 7.00 (t, *J* = 8.7 Hz, 1H), 6.89 (d, *J* = 7.5 Hz, 1H), 6.03 (s, 1H), 5.42 (q, *J* = 7.5 Hz, 1H), 4.81 (dd, *J* = 17.8, 3.0 Hz, 1H), 4.59 (dd, *J* = 17.8, 3.0 Hz, 1H), 3.25 (s, 3H), 1.14 (d, *J* = 7.5 Hz, 3H). **^{13}C NMR** (126 MHz, $CDCl_3$) δ 174.34, 162.26 (d, *J* = 246.1 Hz), 143.91, 137.96, 135.56, 130.94, 129.90, 129.35, 128.85 (d, *J* = 7.8 Hz), 128.60, 126.16, 124.91, 123.05, 115.44 (d, *J* = 21.4 Hz), 108.81, 79.61, 63.79, 26.41, 16.40. **HRMS** (ESI): Calcd for $(M +Na)^+$ $[C21H18O2NFNa]^+$: 358.1214, found: 358.1223.

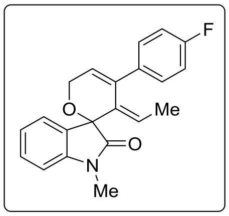

(E)-4'-(3-chlorophenyl)-3'-ethylidene-1-methyl-3',6'-dihydrospiro[indoline-3,2'-pyran]-2-one (**2d**) was prepared according to the general procedure for the gold catalyzed *C*-migration reaction, by using **1d** (48 mg, 0.14 mmol, 80% (E)-isomer) as starting material. After the purification done by silica gel column chromatography with EtOAc / petroleum ether = 1/7 (R_f = 0.19) as eluents, the desired product was obtained in 31% yield (15 mg, 0.04 mmol) as a brown oil. **^{1}H NMR** (500 MHz, $CDCl_3$) δ 7.39 – 7.33 (m, 2H), 7.25 – 7.23 (m, 2H), 7.20 (s, 1H), 7.11 – 7.08 (m, 1H), 7.07 (td, *J* = 7.6, 0.9 Hz, 1H), 6.90 (d, *J* = 7.6 Hz, 1H), 6.08 (s, 1H), 5.44 (q, *J* = 7.3 Hz, 1H), 4.83 (dd, *J* = 18.0, 2.9 Hz, 1H), 4.60 (dd, *J* = 18.0, 3.0 Hz, 1H), 3.25 (s, 3H), 1.16 (d, *J* = 7.3 Hz,

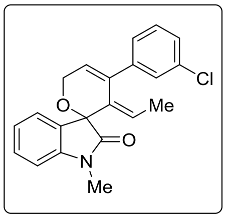

3H). **^{13}C NMR** (126 MHz, $CDCl_3$) δ 174.23, 143.94, 143.73, 135.26, 134.46, 130.48, 129.98, 129.84, 129.46, 129.17, 127.36, 126.42, 125.55, 124.97, 123.11, 108.84, 79.47, 63.79, 26.43, 16.59.**HRMS** (ESI): Calcd for $(M +Na)^+$ [C21H18O2NClNa]$^+$: 374.0918, found: 374.0929.

(E)-3'-ethylidene-1-methyl-4'-(thiophen-2-yl)-3',6'-dihydrospiro[indoline-3,2'-pyran]-2-one

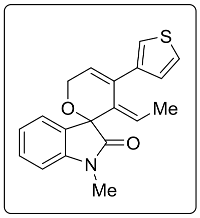

(**2e**) was prepared according to the general procedure for the gold catalyzed *C*-migration reaction, by using **1e** (44 mg, 0.14 mmol, 83% (E)-isomer) as starting material. After the purification done by silica gel column chromatography with EtOAc / petroleum ether = 1/5 (R_f = 0.33) as eluents, the desired product was obtained in 45% yield (20 mg, 0.06 mmol) as a dark brown oil. **^{1}H NMR** (300 MHz, $CDCl_3$) δ 7.46 – 7.30 (m, 2H), 7.26 – 7.22 (m, 1H), 7.10 – 6.98 (m, 2H), 6.93 (d, *J* = 5.0 Hz, 1H), 6.89 (d, *J* = 7.8 Hz, 1H), 6.12 (s, 1H), 5.43 (q, *J* = 7.2 Hz, 1H), 4.79 (dd, *J* = 17.7, 3.0 Hz, 1H), 4.56 (dd, *J* = 17.7, 3.0 Hz, 1H), 3.25 (s, 3H), 1.24 (d, *J* = 7.2 Hz, 3H). **^{13}C NMR** (75 MHz, $CDCl_3$) δ 174 .38, 143.78, 142.66, 131.55, 131.10, 129.82, 129.42, 127.80, 127.50, 126.09, 125.52, 124.86, 123.04, 121.07, 108.78, 79.63, 63.71, 26.42, 15.89. **HRMS** (ESI): Calcd for $(M +Na)^+$ $[C_{19}H_{17}O_2NSNa]^+$: 346.0872, found: 346.0884.

(E)-3'-ethylidene-5-methoxy-1-methyl-4'-phenyl-3',6'-dihydrospiro[indoline-3,2'-pyran]-2-

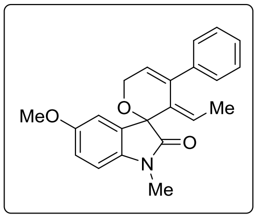

one (**2f**) was prepared according to the general procedure for the gold catalyzed *C*-migration reaction, by using **1f** (45 mg, 0.13 mmol, 83% (E)-isomer) as starting material. After the purification done by silica gel column chromatography with EtOAc / petroleum ether = 1/4 (R_f = 0.35) as eluents, the desired product was obtained in 51% yield (23 mg, 0.07 mmol) as a dark red oil. **^{1}H NMR** (300 MHz, $CDCl_3$) δ 7.42 – 7.27 (m, 3H), 7.25 – 7.16 (m, 2H), 7.03 (d, *J* = 2.5 Hz, 1H), 6.88 (dd, *J* = 8.5, 2.5 Hz, 1H), 6.80 (d, *J* = 8.5 Hz, 1H), 6.06 (s, 1H), 5.44 (q, *J* = 7.4 Hz, 1H), 4.82 (dd, *J* = 17.8, 3.1 Hz, 1H), 4.60 (dd, *J* = 17.8, 3.1 Hz, 1H), 3.74 (s, 3H), 3.23 (s, 3H), 1.13 (d, *J* = 7.4 Hz, 3H). **^{13}C NMR** (75 MHz, $CDCl_3$) δ 174.17, 156.23, 141.87, 137.26, 136.55, 130.77, 130.60, 128.52, 128.38, 127.30, 127.21, 126.23, 113.87, 112.41, 109.08, 79.93, 63.78, 55.99, 26.50, 16.42. **HRMS** (ESI): Calcd for $(M +Na)^+$ $[C_{22}H_{21}O_3NNa]^+$: 370.1414, found: 370.1426.

(E)-3'-ethylidene-5-fluoro-1-methyl-4'-phenyl-3',6'-dihydrospiro[indoline-3,2'-pyran]-2-one

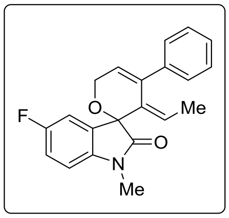

(**2g**) was prepared according to the general procedure for the gold catalyzed *C*-migration reaction, by using **1g** (41 mg, 0.12 mmol, 77% (E)-isomer) as starting material. After the purification done by silica gel column chromatography with EtOAc / petroleum ether = 1/5 (R_f = 0.35) as eluents, the desired product was obtained in 51% yield (21 mg, 0.06 mmol) as an orange oil. **^{1}H NMR** (300 MHz, $CDCl_3$) δ 7.37 – 7.20 (m, 3H), 7.18 – 7.09 (m, 3H), 7.02 (td, *J* = 8.7, 2.6 Hz, 1H), 6.78 (dd, *J* = 8.7, 4.1 Hz, 1H), 6.03 (s, 1H), 5.41 (q, *J* = 7.3 Hz, 1H), 4.78 (dd, *J* = 17.9, 3.0 Hz, 1H), 4.55 (dd, *J* = 17.9, 3.0 Hz, 1H), 3.21 (s, 3H), 1.10 (d, *J* = 7.3 Hz, 3H). **^{13}C NMR** (75 MHz, $CDCl_3$) δ 174.14, 159.38 (d, *J* = 241.3 Hz), 141.61, 139.75, 136.49, 130.86 (d, *J* = 7.7 Hz), 130.43, 128.60, 128.28, 127.36, 127.23, 126.53, 115.94 (d, *J* = 23.5 Hz), 113.04 (d, *J* = 25.3 Hz), 109.27 (d, *J* = 8.0 Hz), 79.69, 63.84, 26.58, 16.45. **HRMS** (ESI): Calcd for $(M+Na)^+$ $[C_{21}H_{18}O_2NFNa]^+$: 358.1214, found: 358.1225.

(E)-5-chloro-3'-ethylidene-1-methyl-4'-phenyl-3',6'-dihydrospiro[indoline-3,2'-pyran]-2-one

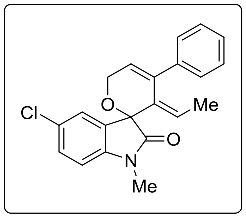

(**2h**) was prepared according to the general procedure for the gold catalyzed *C*-migration reaction, by using **1h** (40 mg, 0.11 mmol, 77% (E)-isomer) as starting material. After the purification done by silica gel column chromatography with EtOAc / petroleum ether = 1/5 (R_f = 0.34) as eluents, the desired product was obtained in 50% yield (20 mg, 0.06 mmol) as an orange oil. **^{1}H NMR** (300 MHz, $CDCl_3$) δ 7.41 – 7.27 (m, 5H), 7.21 (d, *J* = 1.8 Hz, 1H), 7.19 (s, 1H), 6.82 (d, *J* = 8.2 Hz, 1H), 6.07 (d, *J* = 1.1 Hz, 1H), 5.42 (q, *J* = 7.3 Hz, 1H), 4.82 (dd, *J* = 17.8, 3.1 Hz, 1H), 4.59 (dd, *J* = 17.8, 3.1 Hz, 1H), 3.24 (s, 3H), 1.14 (d, *J* = 7.3 Hz, 3H). **^{13}C NMR** (75 MHz, $CDCl_3$) δ 174.00, 142.40, 141.65, 136.42, 131.03, 130.38, 129.69, 128.60, 128.41, 128.33, 127.35, 127.29, 126.58, 125.27, 109.73, 79.58, 63.83, 26.54, 16.44. **HRMS** (ESI): Calcd for $(M+H)^+$ $[C_{21}H_{19}O_2NCl]^+$: 352.1099, found: 352.1107.

(E)-6-bromo-3'-ethylidene-1-methyl-4'-phenyl-3',6'-dihydrospiro[indoline-3,2'-pyran]-2-one (**2i**) was prepared according to the general procedure for the gold catalyzed *C*-migration reaction, by using **1i** (43 mg, 0.11 mmol, 76% (E)-isomer) as starting material. After the purification done by silica gel column chromatography with EtOAc / petroleum ether = 1/8 (R_f = 0.28) as eluents, the desired product was obtained in 58% yield (25 mg, 0.06 mmol) as an orange oil. **^{1}H NMR** (600 MHz, cdcl$_3$) δ 7.35 – 7.22 (m, 4H), 7.21 – 7.16 (m, 3H), 7.05 (d, *J* = 1.7 Hz, 1H), 6.08 – 6.03 (m, 1H), 5.42 (q, *J* = 7.1 Hz, 1H), 4.80 (dd, *J* = 17.8, 3.1 Hz, 1H), 4.57 (dd, *J* = 17.8, 3.1 Hz, 1H), 3.24 (s, 3H), 1.14 (d, *J* = 7.1 Hz, 3H). **^{13}C NMR** (151 MHz, cdcl$_3$) δ 174.18, 145.20, 141.64, 136.52, 130.51, 128.60, 128.39, 128.36, 127.36, 127.21, 126.34, 126.15, 125.78, 123.57, 112.35, 79.33, 63.86, 26.54, 16.43. **HRMS** (ESI): Calcd for $(M+H)^+$ $[C21H19O2NBr]^+$: 396.0594, found: 396.0600.

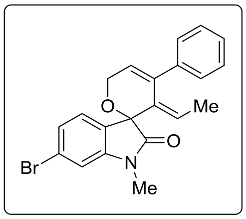

(E)-3'-ethylidene-1-methyl-4'-phenyl-5-(trifluoromethoxy)-3',6'-dihydrospiro[indoline-3,2'-pyran]-2-one (**2j**) was prepared according to the general procedure for the gold catalyzed *C*-migration reaction, by using **1j** (49 mg, 0.12 mmol, 80% (E)-isomer) as starting material. After the purification done by silica gel column chromatography with EtOAc / petroleum ether = 1/7 (R_f = 0.20) as eluents, the desired product was obtained in 65% yield (32 mg, 0.08 mmol) as a brown oil. **^{1}H NMR** (500 MHz, CDCl$_3$) δ 7.39 – 7.27 (m, 4H), 7.24 (d, *J* = 8.5 Hz, 1H), 7.18 (d, *J* = 6.8 Hz, 2H), 6.88 (d, *J* = 8.5 Hz, 1H), 6.09 (s, 1H), 5.43 (q, *J* = 7.2 Hz, 1H), 4.82 (dd, *J* = 17.7, 2.8 Hz, 1H), 4.58 (dd, *J* = 17.7, 3.0 Hz, 1H), 3.27 (s, 3H), 1.15 (d, *J* = 7.3 Hz, 3H). **^{13}C NMR** (126 MHz, CDCl$_3$) δ 174.26, 144.95, 142.52, 141.54, 136.64, 130.95, 130.50, 128.63, 128.39, 127.42, 127.21, 126.59, 122.92, 120.67 (d, *J* = 256.9 Hz), 119.08, 109.17, 79.59, 63.86, 26.60, 16.38. **HRMS** (ESI): Calcd for $(M+H)^+$ $[C22H19O3NF_3]^+$: 402.1312, found: 402.1323.

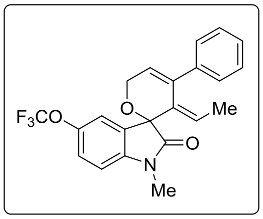

(E)-1-benzyl-3'-ethylidene-4'-phenyl-3',6'-dihydrospiro[indoline-3,2'-pyran]-2-one (**2k**)

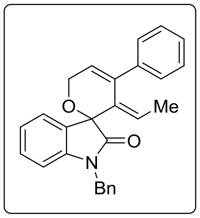

was prepared according to the general procedure for the gold catalyzed *C*-migration reaction, by using **1k** (42 mg, 0.11 mmol, 76% (E)-isomer) as starting material. After the purification done by silica gel column chromatography with EtOAc / petroleum ether = 1/8 (R_f = 0.31) as eluents, the desired product was obtained in 45% yield (19 mg, 0.05 mmol) as a yellow oil. **^{1}H NMR** (500 MHz, $CDCl_3$) δ 7.41 (d, *J* = 6.8 Hz, 1H), 7.38 – 7.30 (m, 6H), 7.30 – 7.27 (m, 2H), 7.25 – 7.19 (m, 3H), 7.01 (td, *J* = 7.6, 0.8 Hz, 1H), 6.76 (d, *J* = 7.6 Hz, 1H), 6.10 (s, 1H), 5.48 (q, *J* = 7.1 Hz, 1H), 5.05 (d, *J* = 15.8 Hz, 1H), 4.89 (d, *J* = 15.8 Hz, 1H), 4.86 (dd, *J* = 17.7, 3.0 Hz, 1H), 4.63 (dd, *J* = 17.7, 3.1 Hz, 1H), 1.16 (d, *J* = 7.3 Hz, 3H). **^{13}C NMR** (126 MHz, $CDCl_3$) δ 174.59, 142.95, 141.90, 136.64, 135.82, 131.13, 129.68, 129.60, 128.96, 128.58, 128.55, 127.75, 127.35, 127.31, 127.25, 126.06, 124.97, 123.04, 109.83, 79.75, 63.85, 43.87, 16.48. **HRMS** (ESI): Calcd for $(M +H)^+$ $[C27H24O2N]^+$: 394.1802, found: 394.1810.

(E)-3'-ethylidene-1-(4-methoxybenzyl)-4'-phenyl-3',6'-dihydrospiro[indoline-3,2'-pyran]-2-one (**2l**)

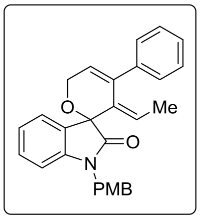

was prepared according to the general procedure for the gold catalyzed *C*-migration reaction, by using **1l** (24 mg, 0.06 mmol, 73% (E)-isomer) as starting material. After the purification done by silica gel column chromatography with EtOAc / petroleum ether = 1/7 (R_f = 0.35) as eluents, the desired product was obtained in 42% yield (10 mg, 0.02 mmol) as a yellow oil. **^{1}H NMR** (600 MHz, $cdcl_3$) δ 7.39 (dd, *J* = 7.6, 0.9 Hz, 1H), 7.35 – 7.19 (m, 8H), 7.00 (td, *J* = 7.6, 0.9 Hz, 1H), 6.90 – 6.83 (m, 2H), 6.79 (d, *J* = 7.8 Hz, 1H), 6.09 (td, *J* = 3.1, 1.4 Hz, 1H), 5.47 (q, *J* = 7.0 Hz, 1H), 4.98 (d, *J* = 15.4 Hz, 1H), 4.85 (dd, *J* = 17.7, 3.2 Hz, 1H), 4.83 (d, *J* = 15.4 Hz, 1H), 4.62 (dd, *J* = 17.7, 3.2 Hz, 1H), 3.78 (s, 3H), 1.15 (d, *J* = 7.0 Hz, 3H). **^{13}C NMR** (151 MHz, $cdcl_3$) δ 174.52, 159.22, 142.96, 141.91, 136.65, 131.09, 129.65, 129.59, 128.76, 128.56, 127.87, 127.31, 127.25, 126.02, 124.92, 122.98, 114.36, 109.86, 79.72, 63.86, 55.42, 43.36, 16.51. **HRMS** (ESI): Calcd for $(M + Na)^+$ $[C_{28}H_{25}O_3NNa]^+$: 446.1727, found: 446.1734.

Gold catalyzed acyl-migration reaction

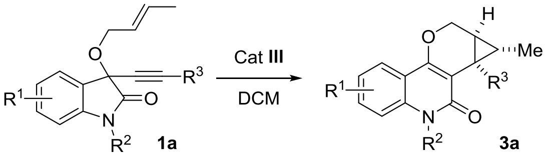

At 0 ^{o}C, to a DCM (0.5 ml) solution of 1,6-enyne (0.1 mmol) was added a solution of cat. **IIl** (4.5 mg, 5 μmol) in DCM (0.5 ml). After warming to room temperature, the reaction mixture was stirred overnight. After TLC showed full conversion of the starting material, the mixture was passed through a short pad of silica gel (Et_2O as the eluent). The resulting solution was concentrated under reduced pressure, followed by silica gel column chromatography (EA / PE as the eluent) to obtain the desired product.

(1S,1aR,9aS)-1,3-dimethyl-1a-phenyl-1a,3,9,9a-tetrahydrocyclopropa[4,5]pyrano[3,2-c]quinolin-2(1H)-one (**3a**) was prepared according to the general procedure for the gold catalyzed carbonyl-migration reaction, by using **1a** (30 mg, 0.09 mmol, 82% (E)-isomer) as starting material. After the purification done by silica gel column chromatography EtOAc / petroleum ether = 1/5 (R_f = 0.24) as eluents, the desired product was obtained in 67% yield (20 mg, 0.06 mmol) as a brown oil. The recrystallization was performed from DCM and petroleum ether.

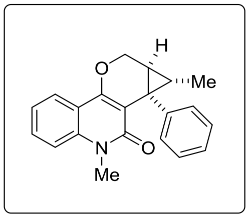

^{1}H NMR (500 MHz, cdcl$_3$) δ 7.94 (dd, *J* = 8.0, 1.5 Hz, 1H), 7.68 (d, *J* = 7.8 Hz, 2H), 7.51 – 7.45 (m, 1H), 7.27 (t, *J* = 7.8 Hz, 2H), 7.22 (d, *J* = 8.4 Hz, 1H), 7.21 – 7.15 (m, 2H), 5.05 (dd, *J* = 11.9, 7.9 Hz, 1H), 3.93 (dd, *J* = 11.9, 7.9 Hz, 1H), 3.57 (s, 3H), 1.61 – 1.54 (m, 1H), 1.30 – 1.23 (m, 4H). **^{13}C NMR** (126 MHz, cdcl$_3$) δ 162.29, 158.12, 140.01, 138.71, 132.63, 130.44, 127.53, 126.50, 123.53, 121.50, 116.44, 115.72, 113.80, 71.77, 31.49, 29.26, 26.70, 26.50, 16.50. **HRMS** (ESI): Calcd for $(M +Na)^+$ $[C21H19O2NNa]^+$: 340.1308, found: 340.1320.

(1S,1aR,9aS)-1a-(3-chlorophenyl)-1,3-dimethyl-1a,3,9,9a-tetrahydrocyclopropa[4,5]pyrano[3,2-c]quinolin-2(1H)-one (**3b**) was prepared according to the general procedure for the gold catalyzed carbonyl-migration reaction, by using **1d** (27 mg, 0.08 mmol, 80% (E)-isomer) as starting material. After the purification done by silica gel column chromatography with EtOAc /

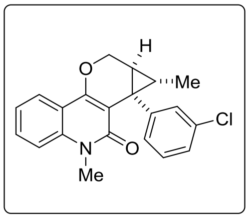

petroleum ether = 1/7 (R_f = 0.25) as eluents, the desired product was obtained in 52% yield (16 mg, 0.05 mmol) as an orange oil. **^{1}H NMR** (500 MHz, $CDCl_3$) δ 7.94 (dd, *J* = 8.0, 1.5 Hz, 1H), 7.65 (d, *J* = 7.7 Hz, 1H), 7.56 (t, *J* = 1.5 Hz, 1H), 7.54 – 7.47 (m, 1H), 7.25 (d, *J* = 8.0 Hz, 1H), 7.20 (t, *J* = 7.7 Hz, 2H), 7.18 – 7.13 (m, 1H), 5.03 (dd, *J* = 11.9, 7.8 Hz, 1H), 3.94 (dd, *J* = 11.9, 5.9 Hz, 1H), 3.58 (s, 3H), 1.62 – 1.50 (m, 1H), 1.25 (s, 4H). **^{13}C NMR** (126 MHz, $CDCl_3$) δ 162.25, 158.26, 142.13, 138.85, 133.26, 132.41, 131.20, 130.69, 128.64, 126.84, 123.66, 121.67, 116.40, 115.03, 113.92, 71.42, 31.57, 29.35, 26.61, 26.50, 16.52. **HRMS** (ESI): Calcd for $(M +H)^+$ $[C21H19O2NCl]^+$: 352.1099, found: 352.1113.

(1S,1aS,9aS)-1,3-dimethyl-1a-(thiophen-3-yl)-1a,3,9,9a-tetrahydrocyclopropa[4,5]pyrano

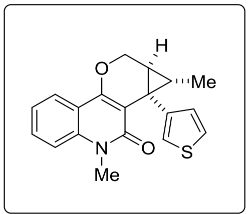

[3,2-c]quinolin-2(1H)-one (**3c**) was prepared according to the general procedure for the gold catalyzed carbonyl-migration reaction, by using **1e** (51 mg, 0.16 mmol, 83% (E)-isomer) as starting material. After the purification done by silica gel column chromatography with EtOAc / petroleum ether = 1/7 (R_f = 0.16) as eluents, the desired product was obtained in 59% yield (30 mg, 0.09 mmol) as a yellow oil. **^{1}H NMR** (300 MHz, $CDCl_3$) δ 7.92 (d, *J* = 8.0 Hz, 1H), 7.50 (t, *J* = 7.8 Hz, 1H), 7.42 (d, *J* = 2.9 Hz, 1H), 7.35 (d, *J* = 4.9 Hz, 1H), 7.29 – 7.10 (m, 4H), 5.04 (dd, *J* = 11.9, 8.0 Hz, 1H), 3.88 (dd, *J* = 11.9, 6.1 Hz, 1H), 3.61 (s, 3H), 1.63 (t, *J* = 6.7 Hz, 1H), 1.20 (s, 4H). **^{13}C NMR** (75 MHz, $CDCl_3$) δ 162.40, 158.21, 140.10, 138.69, 131.47, 130.52, 125.63, 123.51, 123.42, 121.61, 116.47, 115.22, 113.89, 71.97, 31.90, 29.34, 26.14, 21.87, 15.85. **HRMS** (ESI): Calcd for $(M +H)^+$ $[C19H18O2NS]^+$: 324.1053, found: 324.1062.

(1S,1aR,9aS)-6-methoxy-1,3-dimethyl-1a-phenyl-1a,3,9,9a-tetrahydrocyclopropa[4,5]

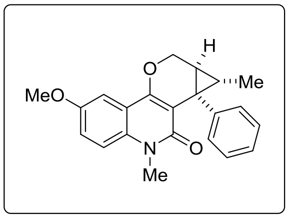

pyrano[3,2-c]quinolin-2(1H)-one (**3d**) was prepared according to the general procedure for the gold catalyzed carbonyl-migration reaction, by using **1f** (52 mg, 0.15 mmol, 83% (E)-isomer) as starting material. After the purification done by silica gel column chromatography with EtOAc / petroleum ether = 1/5 (R_f= 0.29) as eluents, the desired product was obtained in 40% yield (21 mg, 0.06 mmol) as a brown oil. **^{1}H NMR** (500 MHz, $CDCl_3$) δ 7.67 (d, *J* = 7.8 Hz, 2H), 7.39 (d, *J* = 2.9 Hz, 1H), 7.31 – 7.22 (m, 3H), 7.22 – 7.12 (m, 2H), 7.11 (dd, *J* = 9.1, 2.9 Hz, 1H), 5.06

(dd, J = 11.9, 7.9 Hz, 1H), 3.92 (dd, J = 11.9, 6.1 Hz, 1H), 3.87 (s, 3H), 3.56 (s, 3H), 1.63 – 1.51 (m, 1H), 1.26 (s, 4H). **^{13}C NMR** (126 MHz, $CDCl_3$) δ 162.14, 157.94, 154.84, 140.30, 133.70, 132.93, 127.83, 126.80, 119.74, 117.32, 116.55, 115.57, 105.38, 72.21, 56.10, 31.81, 29.68, 27.15, 26.85, 16.78. **HRMS** (ESI): Calcd for $(M+H)^+$ $[C22H22O3N]^+$: 348.1594, found: 348.1605.

(1S,1aR,9aS)-6-fluoro-1,3-dimethyl-1a-phenyl-1a,3,9,9a-tetrahydrocyclopropa[4,5]pyrano

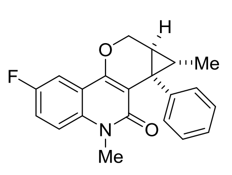

[3,2-c]quinolin-2(1H)-one (**3e**) was prepared according to the general procedure for the gold catalyzed carbonyl-migration reaction, by using **1g** (44 mg, 0.13 mmol, 77% (E)-isomer) as starting material. After the purification done by silica gel column chromatography with EtOAc / petroleum ether = 1/7 (R_f = 0.14) as eluents, the desired product was obtained in 48% yield (21 mg, 0.06 mmol) as a yellow oil. **^{1}H NMR** (300 MHz, $CDCl_3$) δ 7.63 (d, J = 7.2 Hz, 2H), 7.57 (dd, J = 8.9, 1.8 Hz, 1H), 7.31 – 7.07 (m, 5H), 5.01 (dd, J = 11.9, 7.8 Hz, 1H), 3.91 (dd, J = 11.9, 5.9 Hz, 1H), 3.52 (s, 3H), 1.59 – 1.49 (m, 1H), 1.22 (s, 4H). **^{13}C NMR** (75 MHz, $CDCl_3$) δ 161.96, 159.43, 156.72 (d, J = 73.4 Hz), 139.73, 135.26, 132.66, 127.61, 126.65, 118.13 (d, J = 23.9 Hz), 117.42 (d, J = 8.4 Hz), 116.71, 115.43 (d, J = 8.0 Hz), 109.08 (d, J = 24.1 Hz), 71.81, 31.61, 29.56, 26.75, 26.52, 16.50. **HRMS** (ESI): Calcd for $(M+H)^+$ $[C21H19O2NF]^+$: 336.1394, found: 336.1404.

(1S,1aR,9aS)-6-chloro-1,3-dimethyl-1a-phenyl-1a,3,9,9a-tetrahydrocyclopropa[4,5]pyrano

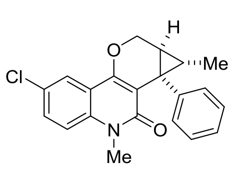

[3,2-c]quinolin-2(1H)-one (**3f**) was prepared according to the general procedure for the gold catalyzed carbonyl-migration reaction, by using **1h** (44 mg, 0.13 mmol, 77% (E)-isomer) as starting material. After the purification done by silica gel column chromatography with EtOAc / petroleum ether = 1/7 (R_f = 0.17) as eluents, the desired product was obtained in 50% yield (22 mg, 0.06 mmol) as a brown oil. **^{1}H NMR** (500 MHz, $CDCl_3$) δ 7.92 (d, J = 2.4 Hz, 1H), 7.66 (d, J = 7.2 Hz, 2H), 7.43 (dd, J = 8.9, 2.4 Hz, 1H), 7.33 – 7.23 (m, 2H), 7.23 – 7.12 (m, 2H), 5.05 (dd, J = 11.9, 7.8 Hz, 1H), 3.96 (dd, J = 11.9, 5.8 Hz, 1H), 3.55 (s, 3H), 1.65 – 1.51 (m, 1H),

1.27 (s, 4H). **^{13}C NMR** (75 MHz, $CDCl_3$) δ 161.99, 157.03, 139.67, 137.21, 132.65, 130.41, 127.62, 127.31, 126.68, 123.09, 117.56, 116.66, 115.32, 71.79, 31.62, 29.47, 26.71, 26.50, 16.49. **HRMS** (ESI): Calcd for $(M + H)^+$ [C21H19O2NCl]$^+$: 352.1099, found: 352.1112.

(1S,1aR,9aS)-5-bromo-1,3-dimethyl-1a-phenyl-1a,3,9,9a-tetrahydrocyclopropa[4,5]pyrano[3,2-c]quinolin-2(1H)-one (**3g**) was prepared according to the general procedure for the gold catalyzed carbonyl-migration reaction, by using **1i** (51 mg, 0.13 mmol, 76% (E)-isomer) as starting material. After the purification done by silica gel column chromatography with EtOAc / petroleum ether = 1/7 (R_f = 0.30) as eluents, the desired product was obtained in 47% yield (24 mg, 0.06 mmol) as a red oil.

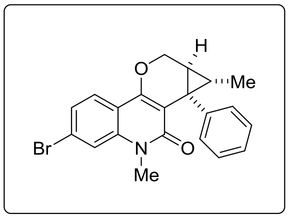

^{1}H NMR (300 MHz, $CDCl_3$) δ 7.75 (d, J = 8.5 Hz, 1H), 7.62 (d, J = 7.2 Hz, 2H), 7.36 (s, 1H), 7.34 – 7.19 (m, 4H), 7.17 (d, J = 7.2 Hz, 1H), 5.01 (dd, J = 11.9, 7.8 Hz, 1H), 3.91 (dd, J = 11.9, 5.9 Hz, 1H), 3.50 (s, 3H), 1.53 (t, J = 6.4 Hz, 1H), 1.22 (s, 4H). **^{13}C NMR** (75 MHz, $CDCl_3$) δ 162.11, 157.70, 139.70, 139.59, 132.63, 127.61, 126.66, 124.99, 124.84, 124.75, 116.79, 115.95, 115.31, 71.74, 31.54, 29.41, 26.64, 26.46, 16.49. **HRMS** (ESI): Calcd for $(M + H)^+$ [C21H19O2NBr]$^+$: 396.0594, found: 396.0602.

(1S,1aR,9aS)-1,3-dimethyl-1a-phenyl-6-(trifluoromethoxy)-1a,3,9,9a-tetrahydrocyclopropa[4,5]pyrano[3,2-c]quinolin-2(1*H*)-one (**3h**) was prepared according to the general procedure for the gold catalyzed carbonyl-migration reaction, by using **1j** (34 mg, 0.08 mmol, 80% (E)-isomer) as starting material. After the purification done by silica gel column chromatography with EtOAc / petroleum ether = 1/7 (R_f = 0.24) as eluents, the desired product was obtained in 44% yield (15 mg, 0.04 mmol) as an orange oil.

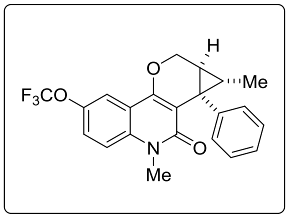

^{1}H NMR (500 MHz, $CDCl_3$) δ 7.79 (s, 1H), 7.65 (d, J = 7.2 Hz, 2H), 7.34 (dd, J = 9.1, 2.0 Hz, 1H), 7.32 – 7.16 (m, 5H), 5.05 (dd, J = 11.9, 7.8 Hz, 1H), 3.97 (dd, J = 11.9, 5.8 Hz, 1H), 3.57 (s, 3H), 1.26 (s, 5H). **^{13}C NMR** (126 MHz, $CDCl_3$) δ 162.04, 157.10, 143.65, 139.63, 137.25, 132.67, 127.64, 126.73, 123.58, 120.73 (d, J = 257.0 Hz), 117.24, 116.85, 115.89, 115.25, 71.75, 31.71, 29.58, 26.79, 26.60, 16.49. **HRMS** (ESI): Calcd for $(M + H)^+$ $[C_{22}H_{19}O_3NF_3]^+$: 402.1312, found: 402.1323.

(1S,1aR,9aS)-3-benzyl-1-methyl-1a-phenyl-1a,3,9,9a-tetrahydrocyclopropa[4,5]pyrano[3,2-

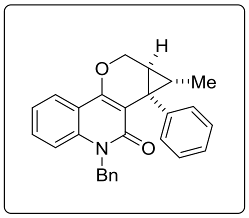

c]quinolin-2(1*H*)-one (**3i**) was prepared according to the general procedure for the gold catalyzed carbonyl-migration reaction, by using **1k** (46 mg, 0.12 mmol, 76% (E)-isomer) as starting material. After the purification done by silica gel column chromatography with EtOAc / petroleum ether = 1/7 (R_f = 0.40) as eluents, the desired product was obtained in 52% yield (24 mg, 0.06 mmol) as a brown oil. **^{1}H NMR** (500 MHz, DMSO) δ 7.88 (dd, *J* = 8.0, 1.5 Hz, 1H), 7.59 (d, *J* = 7.0 Hz, 2H), 7.47 – 7.42 (m, 1H), 7.32 – 7.12 (m, 9H), 7.05 (d, *J* = 7.3 Hz, 2H), 5.47 (bs, 1H), 5.27 (bs, 1H), 5.14 (dd, *J* = 11.9, 8.0 Hz, 1H), 3.99 (dd, *J* = 12.0, 6.1 Hz, 1H), 1.66 – 1.59 (m, 1H), 1.33 – 1.26 (m, 1H), 1.14 (d, *J* = 6.3 Hz, 3H). **^{13}C NMR** (126 MHz, $CDCl_3$) δ 162.65, 158.66, 140.22, 138.47, 137.22, 132.90, 130.72, 129.00, 127.82, 127.30, 126.80, 123.92, 121.88, 116.94, 115.84, 114.99, 72.17, 45.97, 31.83, 27.11, 26.61, 16.79. **HRMS** (ESI): Calcd for $(M +H)^+$ $[C27H24O2N]^+$: 394.1802, found: 394.1809.

(1S,1aR,9aS)-3-(4-methoxybenzyl)-1-methyl-1a-phenyl-1a,3,9,9a-tetrahydrocyclopropa[4,5]

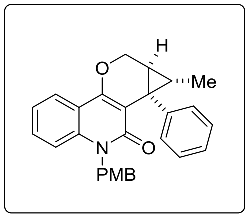

pyrano[3,2-c]quinolin-2(1*H*)-one (**3j**) was prepared according to the general procedure for the gold catalyzed carbonyl-migration reaction, by using **1l** (23 mg, 0.05 mmol, 73% (E)-isomer) as starting material. After the purification done by silica gel column chromatography with EtOAc / petroleum ether = 1/7 (R_f = 0.28) as eluents, the desired product was obtained in 43% yield (10 mg, 0.02 mmol) as a red oil. **^{1}H NMR** (500 MHz, $CDCl_3$) δ 7.94 (d, *J* = 7.9 Hz, 1H), 7.69 (d, *J* = 7.6 Hz, 2H), 7.34 (m, 1H), 7.28 – 7.25 (m, 2H), 7.20 – 7.12 (m, 3H), 6.99 (d, *J* = 8.2 Hz, 2H), 6.74 (d, *J* = 8.2 Hz, 2H), 5.49 (bs, 1H), 5.19 (bs, 1H), 5.09 (dd, *J* = 11.9, 8.0 Hz, 1H), 3.96 (dd, *J* = 11.9, 6.1 Hz, 1H), 3.72 (s, 3H), 1.62 – 1.58 (m, 1H), 1.34 – 1.27 (m, 4H). **^{13}C NMR** (126 MHz, $CDCl_3$) δ 162.63, 158.94, 158.62, 140.23, 138.46, 132.90, 130.66, 129.32, 128.15, 127.81, 126.77, 123.89, 121.82, 116.94, 115.88, 114.97, 114.43, 72.20, 55.60, 45.40, 31.81, 27.12, 26.60, 16.79. **HRMS** (ESI): Calcd for $(M + Na)^+$ $[C_{28}H_{25}O_3NNa]^+$: 446.1727, found: 446.1734.

Gold catalyzed *O*-migration reaction with MeOH as nucleophile

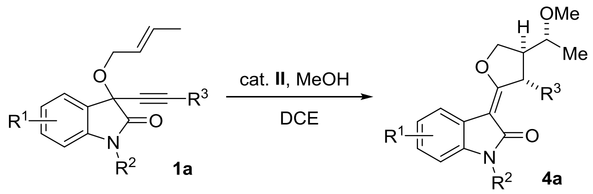

To a DCE (1,2-dichloroethane) (0.5 ml) solution of 1,6-enyne (0.5 mmol) and MeOH (41 µL, 1 mmol) in a pressure tube equipped with a stirring bar was added a solution of cat. **II** (3.9 mg, 5 µmol) in DCE (0.5 mL) and the mixture was stirred at 60 ^oC overnight until TLC showed full conversion of the starting material. After cooling to room temperature, the reaction mixture was passed through a short pad of silica gel (Et_2O as the eluent). The resulting solution was concentrated under reduced pressure, followed by silica gel column chromatography (EA / PE as the eluent) to obtain the desired product.

(E)-3-((3R,4S)-4-((R)-1-methoxyethyl)-3-phenyldihydrofuran-2(3H)-ylidene)-1-

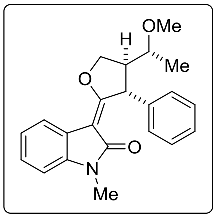

methylindolin-2-one (**4a**) was prepared from according to the general procedure for the gold catalyzed *O*-migration reaction with MeOH as nucleophile, by using **1a** (30 mg, 0.09 mmol, 82% (E)-isomer) as starting material. After the purification done by silica gel column chromatography with EtOAc / petroleum ether = 1/3 (R_f = 0.20) as eluents, the desired product was obtained in 73% yield (24 mg, 0.07 mmol) as a yellow oil. The recrystallization was performed from $CHCl_3$ and petroleum ether. **^{1}H NMR** (500 MHz, $CDCl_3$) δ 7.79 (d, *J* = 7.6 Hz, 1H), 7.35 – 7.23 (m, 5H), 7.22 (d, *J* = 7.6 Hz, 1H), 7.18 (dd, *J* = 7.6, 1.2 Hz, 1H), 7.06 (td, *J* = 7.6, 0.9 Hz, 1H), 6.79 (d, *J* = 7.6 Hz, 1H), 5.06 (s, 1H), 4.73 (dd, *J* = 9.5, 1.5 Hz, 1H), 4.60 (dd, *J* = 9.5, 6.5 Hz, 1H), 3.36 – 3.26 (m, 4H), 3.17 (s, 3H), 2.44 (t, *J* = 6.5 Hz, 1H), 1.28 (d, *J* = 6.1 Hz, 3H). **^{13}C NMR** (126 MHz, $CDCl_3$) δ 172.58, 168.06, 141.13, 140.61, 128.96, 127.08, 126.92, 126.16, 122.81, 122.53, 121.55, 107.17, 101.20, 76.98, 73.23, 56.74, 52.93, 51.21, 25.88, 16.63. **HRMS** (ESI): Calcd for $(M+H)^+$ $[C22H24O3N]^+$: 350.1751, found: 350.1763.

(E)-3-((3R,4S)-3-(4-fluorophenyl)-4-((R)-1-methoxyethyl)dihydrofuran-2(3H)-ylidene)-1-methylindolin-2-one (**4b**) was prepared according to the general procedure for the gold catalyzed *O*-migration reaction with MeOH as nucleophile, by using **1c** (36 mg, 0.11 mmol, 78% (E)-isomer) as starting material. After the purification done by silica gel column chromatography with EtOAc / petroleum ether = 1/4 (R_f = 0.14) as eluents, the desired product was obtained in 58% yield (23 mg, 0.06 mmol) as a yellow oil. **^{1}H NMR** (500 MHz, $CDCl_3$) δ 7.78 (d, *J* = 7.6 Hz, 1H), 7.25 – 7.14 (m, 3H), 7.06 (td, *J* = 7.6, 0.8 Hz, 1H), 6.98 (t, *J* = 8.7 Hz, 2H), 6.80 (d, *J* = 7.6 Hz, 1H), 5.03 (s, 1H), 4.72 (dd, *J* = 9.5, 1.5 Hz, 1H), 4.59 (dd, *J* = 9.5, 6.4 Hz, 1H), 3.37 – 3.30 (m, 4H), 3.18 (s, 3H), 2.42 (t, *J* = 6.4 Hz, 1H), 1.27 (d, *J* = 6.1 Hz, 3H). **^{13}C NMR** (126 MHz, $CDCl_3$) δ 172.25, 168.03, 161.77 (d, *J* = 245.1 Hz), 140.63, 136.96, 128.63 (d, *J* = 8.0 Hz), 126.30, 122.67, 122.57, 121.63, 115.81 (d, *J* = 21.4 Hz), 107.25, 101.25, 76.91, 73.21, 56.73, 52.91, 50.42, 25.90, 16.53. **HRMS** (ESI): Calcd for $(M +H)^+$ $[C_{22}H_{23}O_3NF]^+$: 368.1657, found: 368.1669.

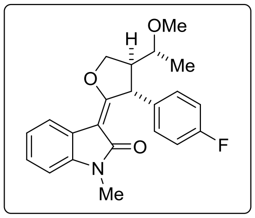

(E)-3-((3R,4S)-3-(3-chlorophenyl)-4-((R)-1-methoxyethyl)dihydrofuran-2(3H)-ylidene)-1-methylindolin-2-one (**4c**) was prepared according to the general procedure for the gold catalyzed *O*-migration reaction with MeOH as nucleophile, by using **1d** (34 mg, 0.10 mmol, 80% (E)-isomer) as starting material. After the purification done by silica gel column chromatography with EtOAc / petroleum ether = 1/4 (R_f = 0.15) as eluents, the desired product was obtained in 67% yield (25 mg, 0.07 mmol) as a brown oil. **^{1}H NMR** (500 MHz, $CDCl_3$) δ 7.78 (d, *J* = 7.6 Hz, 1H), 7.25 – 7.12 (m, 5H), 7.07 (td, *J* = 7.6, 0.9 Hz, 1H), 6.80 (d, *J* = 7.6 Hz, 1H), 5.03 (s, 1H), 4.71 (dd, *J* = 9.6, 1.6 Hz, 1H), 4.58 (dd, *J* = 9.6, 6.5 Hz, 1H), 3.33 (s, 4H), 3.18 (s, 3H), 2.43 (t, *J* = 6.5 Hz, 1H), 1.26 (d, *J* = 6.1 Hz, 3H). **^{13}C NMR** (126 MHz, $CDCl_3$) δ 171.40, 168.01, 143.18, 140.70, 134.75, 130.18, 127.24, 127.05, 126.39, 125.49, 122.66, 122.59, 121.66, 107.29, 101.48, 76.86, 73.09, 56.75, 52.79, 50.81, 25.91, 16.49. **HRMS** (ESI): Calcd for $(M +H)^+$ $[C_{22}H_{23}O_3NCl]^+$: 384.1361, found: 384.1378.

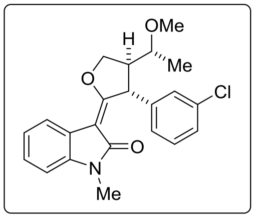

(E)-3-((3R,4S)-3-(4-bromophenyl)-4-((R)-1-methoxyethyl)dihydrofuran-2(3H)-ylidene)-1-methylindolin-2-one (**4d**) was prepared according to the general procedure for the gold catalyzed *O*-migration reaction with MeOH as nucleophile, by using **1m** (41 mg, 0.10 mmol, 76% (E)-isomer) as starting material. After the purification done by silica gel column chromatography with EtOAc / petroleum ether = 1/4 (R_f = 0.17) as eluents, the desired product was obtained in 75% yield (33 mg, 0.08 mmol) as an orange oil. **^{1}H NMR** (500 MHz, $CDCl_3$) δ 7.77 (d, *J* = 7.5 Hz, 1H), 7.41 (d, *J* = 8.4 Hz, 2H), 7.20 (td, *J* = 7.5, 0.9 Hz, 1H), 7.13 (d, *J* = 8.4 Hz, 2H), 7.06 (t, *J* = 7.5 Hz, 1H), 6.80 (d, *J* = 7.5 Hz, 1H), 4.99 (s, 1H), 4.71 (dd, *J* = 9.6, 1.5 Hz, 1H), 4.57 (dd, *J* = 9.5, 6.5 Hz, 1H), 3.36 – 3.30 (m, 4H), 3.17 (s, 3H), 2.41 (t, *J* = 6.5 Hz, 1H), 1.26 (d, *J* = 6.1 Hz, 3H). **^{13}C NMR** (126 MHz, $CDCl_3$) δ 171.77, 167.99, 140.66, 140.30, 132.06, 128.85, 126.37, 122.60, 121.65, 120.85, 107.28, 101.35, 76.88, 73.20, 56.74, 52.76, 50.63, 25.91, 16.49. **HRMS** (ESI): Calcd for $(M+H)^+$ $[C_{22}H_{23}O_3NBr]^+$: 428.0856, found: 428.0865.

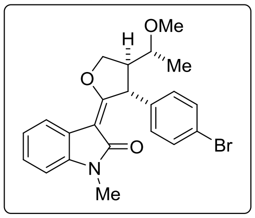

(E)-3-((3R,4S)-4-((R)-1-methoxyethyl)-3-(thiophen-3-yl)dihydrofuran-2(3H)-ylidene)-1-methylindolin-2-one (**4e**) was prepared according to the general procedure for the gold catalyzed *O*-migration reaction with MeOH as nucleophile, by using **1e** (44 mg, 0.14 mmol, 83% (E)-isomer) as starting material. After the purification done by silica gel column chromatography with EtOAc / petroleum ether = 1/4 (R_f = 0.10) as eluents, the desired product was obtained in 77% yield (37 mg, 0.10 mmol) as a red oil. **^{1}H NMR** (500 MHz, $CDCl_3$) δ 7.74 (d, *J* = 7.5 Hz, 1H), 7.26 – 7.24 (m, 1H), 7.18 (td, *J* = 7.5, 1.2 Hz, 1H), 7.13 – 6.99 (m, 3H), 6.79 (d, *J* = 7.5 Hz, 1H), 5.16 (s, 1H), 4.73 (dd, *J* = 9.4, 1.1 Hz, 1H), 4.60 (dd, *J* = 9.4, 6.3 Hz, 1H), 3.31 (s, 3H), 3.29 – 3.23 (m, 1H), 3.21 (s, 3H), 2.50 (t, *J* = 6.3 Hz, 1H), 1.25 (d, *J* = 6.1 Hz, 3H). **^{13}C NMR** (126 MHz, $CDCl_3$) δ 172.29, 168.14, 140.51, 140.13, 127.08, 126.19, 126.16, 122.79, 122.52, 121.57, 120.95, 107.17, 100.93, 76.72, 73.50, 56.73, 51.74, 46.38, 25.91, 16.65. **HRMS** (ESI): Calcd for $(M+H)^+$ $[C_{20}H_{22}O_3NS]^+$: 356.1315, found: 356.1326.

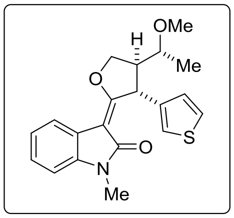

(E)-3-((3R,4S)-4-((R)-1-methoxyethyl)-3-phenyldihydrofuran-2(3H)-ylidene)-1,5,7-trimethylindolin-2-one (**4f**) was prepared according to the general procedure for the gold catalyzed *O*-migration reaction with MeOH as nucleophile, by using **1n** (40 mg, 0.12 mmol, 80% (E)-isomer) as starting material. After the purification done by silica gel column chromatography with EtOAc / petroleum ether = 1/4 (R_f = 0.18) as eluents, the desired product was obtained in 78% yield (34 mg, 0.09 mmol) as a brown oil. **^{1}H NMR** (500 MHz, $CDCl_3$) δ 7.54 (s, 1H), 7.34 – 7.15 (m, 5H), 6.74 (s, 1H), 5.09 (s, 1H), 4.73 (dd, *J* = 9.5, 1.3 Hz, 1H), 4.58 (dd, *J* = 9.5, 6.4 Hz, 1H), 3.43 (s, 3H), 3.34 (s, 3H), 3.30 (dd, *J* = 7.3, 6.3 Hz, 1H), 2.52 (s, 3H), 2.42 (dd, *J* = 7.3, 6.4 Hz, 1H), 2.34 (s, 3H), 1.29 (d, *J* = 6.3 Hz, 3H). **^{13}C NMR** (126 MHz, $CDCl_3$) δ 171.96, 168.64, 141.24, 136.27, 130.78, 130.51, 128.91, 127.04, 126.83, 123.38, 121.33, 118.47, 101.30, 77.00, 73.04, 56.72, 52.89, 51.23, 29.12, 21.06, 19.08, 16.63. **HRMS** (ESI): Calcd for $(M+H)^+$ $[C_{24}H_{28}O_3N]^+$: 378.2064, found: 378.2075.

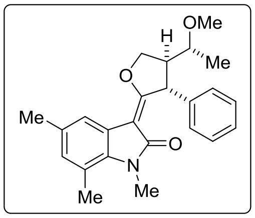

(E)-5-methoxy-3-((3R,4S)-4-((R)-1-methoxyethyl)-3-phenyldihydrofuran-2(3H)-ylidene)-1-methylindolin-2-one (**4g**) was prepared according to the general procedure for the gold catalyzed *O*-migration reaction with MeOH as nucleophile, by using **1f** (47 mg, 0.14 mmol, 83% (E)-isomer) as starting material. After the purification done by silica gel column chromatography with EtOAc / petroleum ether = 1/3 (R_f = 0.22) as eluents, the desired product was obtained in 64% yield (33 mg, 0.09 mmol) as a brown oil. **^{1}H NMR** (500 MHz, $CDCl_3$) δ 7.44 (d, *J* = 2.5 Hz, 1H), 7.43 – 7.41 (m, 1H), 7.33 – 7.23 (m, 4H), 7.23 – 7.18 (m, 1H), 6.75 (dd, *J* = 8.4, 2.5 Hz, 1H), 6.68 (d, *J* = 8.4 Hz, 1H), 5.06 (s, 1H), 4.73 (dd, *J* = 9.5, 1.5 Hz, 1H), 4.60 (dd, *J* = 9.5, 6.3 Hz, 1H), 3.86 (s, 3H), 3.34 (s, 3H), 3.31 (dd, *J* = 7.4, 6.1 Hz, 1H), 3.14 (s, 3H), 2.44 (dd, *J* = 7.4, 6.3 Hz, 1H), 1.28 (d, *J* = 6.1 Hz, 3H). **^{13}C NMR** (126 MHz, $CDCl_3$) δ 172.65, 167.94, 155.49, 141.12, 134.78, 128.95, 127.06, 126.91, 123.69, 111.37, 109.52, 107.31, 101.58, 76.95, 73.27, 56.73, 56.18, 52.89, 51.18, 25.96, 16.62. **HRMS** (ESI): Calcd for $(M+H)^+$ $[C_{23}H_{26}O_4N]^+$: 380.1856, found: 380.1867.

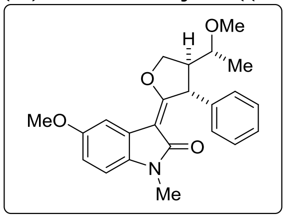

(E)-5-fluoro-3-((3R,4S)-4-((R)-1-methoxyethyl)-3-phenyldihydrofuran-2(3H)-ylidene)-1-

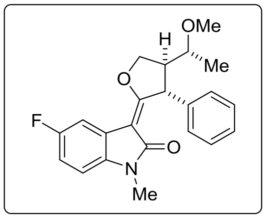

methylindolin-2-one (**4h**) was prepared according to the general procedure for the gold catalyzed *O*-migration reaction with MeOH as nucleophile, by using **1g** (39 mg, 0.12 mmol, 77% (E)-isomer) as starting material. After the purification done by silica gel column chromatography with EtOAc / petroleum ether = 1/3 (R_f = 0.30) as eluents, the desired product was obtained in 63% yield (27 mg, 0.07 mmol) as a brown oil. **^{1}H NMR** (500 MHz, $CDCl_3$) δ 7.52 (dd, *J* = 8.6, 2.6 Hz, 1H), 7.35 – 7.16 (m, 5H), 6.92 – 6.82 (m, 1H), 6.67 (dd, *J* = 8.6, 4.2 Hz, 1H), 5.04 (s, 1H), 4.75 (dd, *J* = 9.6, 1.6 Hz, 1H), 4.63 (dd, *J* = 9.6, 6.4 Hz, 1H), 3.34 (s, 4H), 3.15 (s, 3H), 2.50 – 2.41 (m, 1H), 1.27 (d, *J* = 6.1 Hz, 3H). **^{13}C NMR** (126 MHz, $CDCl_3$) δ 173.69, 167.87, 159.01 (d, *J* = 236.5 Hz), 140.91, 136.63, 129.02, 127.05, 127.03, 123.83 (d, *J* = 9.9 Hz), 112.04 (d, *J* = 24.0 Hz), 110.10 (d, *J* = 26.1 Hz), 107.20 (d, *J* = 8.6 Hz), 101.08, 76.99, 73.56, 56.76, 52.83, 51.44, 26.01, 16.58. **HRMS** (ESI): Calcd for $(M+H)^+$ $[C_{22}H_{24}O_3N]^+$: 350.1751, found: 350.1757.

(E)-5-chloro-3-((3R,4S)-4-((R)-1-methoxyethyl)-3-phenyldihydrofuran-2(3H)-ylidene)-1-

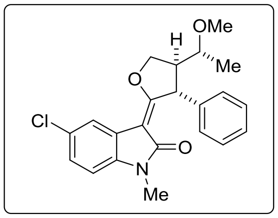

methylindolin-2-one (**4i**) was prepared according to the general procedure for the gold catalyzed *O*-migration reaction with MeOH as nucleophile, by using **1h** (37 mg, 0.11 mmol, 77% (E)-isomer) as starting material. After the purification done by silica gel column chromatography with EtOAc / petroleum ether = 1/3 (R_f = 0.28) as eluents, the desired product was obtained in 62% yield (25 mg, 0.07 mmol) as a yellow oil. **^{1}H NMR** (500 MHz, $CDCl_3$) δ 7.77 (d, *J* = 2.0 Hz, 1H), 7.34 – 7.16 (m, 5H), 7.14 (dd, *J* = 8.2, 2.0 Hz, 1H), 6.68 (d, *J* = 8.2 Hz, 1H), 5.03 (s, 1H), 4.76 (dd, *J* = 9.6, 1.5 Hz, 1H), 4.64 (dd, *J* = 9.6, 6.4 Hz, 1H), 3.36 – 3.29 (m, 4H), 3.15 (s, 3H), 2.46 (t, *J* = 6.4 Hz, 1H), 1.27 (d, *J* = 6.1 Hz, 3H). **^{13}C NMR** (126 MHz, $CDCl_3$) δ 174.19, 167.94, 141.10, 139.26, 129.29, 127.30, 127.15, 125.93, 124.43, 122.80, 108.15, 100.69, 77.26, 77.17, 73.89, 57.04, 53.05, 51.79, 26.25, 16.82. **HRMS** (ESI): Calcd for $(M+H)^+$ $[C_{22}H_{23}O_3NCl]^+$: 384.1361, found: 384.1379.

(E)-6-bromo-3-((3R,4S)-4-((R)-1-methoxyethyl)-3-phenyldihydrofuran-2(3H)-ylidene)-1-methylindolin-2-one (**4j**) was prepared according to the general procedure for the gold catalyzed *O*-migration reaction with MeOH as nucleophile, by using **1i** (42 mg, 0.11 mmol, 76% (E)-isomer) as starting material. After the purification done by silica gel column chromatography with EtOAc / petroleum ether = 1/3 (R_f = 0.33) as eluents, the desired product was obtained in 70% yield (32 mg, 0.07 mmol) as a yellow oil. **^{1}H NMR** (500 MHz, $CDCl_3$) δ 7.62 (d, *J* = 7.9 Hz, 1H), 7.40 – 7.19 (m, 6H), 7.17 (d, *J* = 7.9 Hz, 1H), 6.92 (s, 1H), 5.01 (s, 1H), 4.73 (d, *J* = 9.5 Hz, 1H), 4.68 – 4.57 (m, 1H), 3.33 (s, 4H), 3.14 (s, 3H), 2.45 (s, 1H), 1.27 (d, *J* = 5.7 Hz, 3H). **^{13}C NMR** (126 MHz, $CDCl_3$) δ 173.70, 168.08, 141.97, 141.14, 129.28, 127.35, 127.31, 124.51, 123.79, 121.98, 119.68, 110.79, 100.67, 77.24, 73.78, 57.02, 53.10, 51.76, 26.23, 16.85. **HRMS** (ESI): Calcd for $(M+H)^+$ $[C_{22}H_{23}O_3NBr]^+$: 428.0856, found: 428.0866.

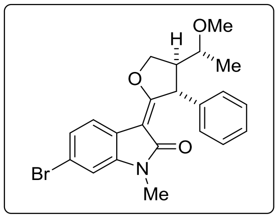

(E)-3-((3R,4S)-4-((R)-1-methoxyethyl)-3-phenyldihydrofuran-2(3H)-ylidene)-1-methyl-5-(trifluoromethoxy)indolin-2-one (**4k**) was prepared according to the general procedure for the gold catalyzed *O*-migration reaction with MeOH as nucleophile, by using **1j** (35 mg, 0.09 mmol, 80% (E)-isomer) as starting material. After the purification done by silica gel column chromatography with EtOAc / petroleum ether = 1/4 (R_f = 0.11) as eluents, the desired product was obtained in 71% yield (27 mg, 0.06 mmol) as a yellow oil. **^{1}H NMR** (500 MHz, $CDCl_3$) δ 7.65 (s, 1H), 7.35 – 7.29 (m, 2H), 7.28 – 7.15 (m, 3H), 7.05 (d, *J* = 8.4 Hz, 1H), 6.73 (d, *J* = 8.4 Hz, 1H), 5.04 (s, 1H), 4.77 (dd, *J* = 9.6, 1.6 Hz, 1H), 4.65 (dd, *J* = 9.6, 6.5 Hz, 1H), 3.34 (s, 4H), 3.16 (s, 3H), 2.46 (t, *J* = 6.5 Hz, 1H), 1.27 (d, *J* = 6.1 Hz, 3H). **^{13}C NMR** (126 MHz, $CDCl_3$) δ 174.27, 167.88, 144.29, 140.82, 139.03, 129.05, 127.06, 123.76, 120.91 (d, *J* = 255.9 Hz), 118.92, 116.15, 107.17, 100.61, 76.97, 73.80, 56.77, 52.80, 51.60, 32.77, 26.04, 16.57. **HRMS** (ESI): Calcd for $(M+H)^+$ $[C_{23}H_{23}O_4NF_3]^+$: 434.1574, found: 434.1573.

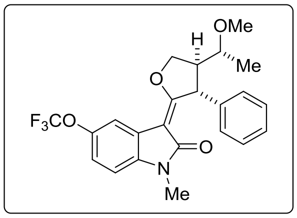

(E)-1-benzyl-3-((3R,4S)-4-((R)-1-methoxyethyl)-3-phenyldihydrofuran-2(3H)-ylidene)indolin-2-one (**4l**) was prepared according to the general procedure for the gold catalyzed *O*-migration reaction with MeOH as nucleophile, by using **1k** (39 mg, 0.10 mmol, 76% (E)-isomer) as starting material. After the purification done by silica gel column chromatography with EtOAc / petroleum ether = 1/4 (R_f = 0.29) as eluents, the desired product was obtained in 69% yield (29 mg, 0.07 mmol) as a yellow oil. **^{1}H NMR** (500 MHz, $CDCl_3$) δ 7.81 (d, *J* = 6.4 Hz, 1H), 7.37 – 7.13 (m, 11H), 7.12 – 7.05 (m, 1H), 7.05 – 7.00 (m, 1H), 6.68 (d, *J* = 7.4 Hz, 1H), 5.10 (s, 1H), 5.01 (d, *J* = 15.8 Hz, 1H), 4.81 – 4.74 (m, 2H), 4.63 (dd, *J* = 9.5, 6.6 Hz, 1H), 3.36 (s, 4H), 2.47 (t, *J* = 6.6 Hz, 1H), 1.31 (d, *J* = 6.1 Hz, 3H). **^{13}C NMR** (126 MHz, $CDCl_3$) δ 172.95, 167.98, 141.13, 139.75, 137.01, 128.95, 128.65, 127.33, 127.27, 127.10, 126.92, 126.09, 122.96, 122.60, 121.62, 108.20, 101.07, 76.91, 73.35, 56.74, 53.03, 51.30, 43.43, 16.72. **HRMS** (ESI): Calcd for $(M+H)^+$ $[C_{26}H_{28}O_3N]^+$: 426.2064, found: 426.2076.

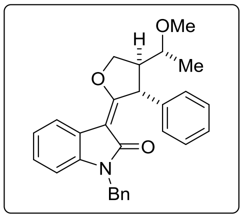

(E)-1-(4-methoxybenzyl)-3-((3R,4S)-4-((R)-1-methoxyethyl)-3-phenyldihydrofuran-2(3H)-ylidene)indolin-2-one (**4m**) was prepared from according to the general procedure for the gold catalyzed *O*-migration reaction with MeOH as nucleophile, by using **1l** (25 mg, 0.06 mmol, 73% (E)-isomer) as starting material. After the purification done by silica gel column chromatography with EtOAc / petroleum ether = 1/3 (R_f = 0.28) as eluents, the desired product was obtained in 60% yield (16 mg, 0.04 mmol) as a brown oil. **^{1}H NMR** (500 MHz, $CDCl_3$) δ 7.79 (d, *J* = 7.5 Hz, 1H), 7.36 – 7.25 (m, 4H), 7.25 – 7.20 (m, 1H), 7.14 (d, *J* = 8.6 Hz, 2H), 7.07 (td, *J* = 7.5, 1.2 Hz, 1H), 7.02 (t, *J* = 7.5 Hz, 1H), 6.77 (d, *J* = 8.6 Hz, 2H), 6.69 (d, *J* = 7.6 Hz, 1H), 5.09 (s, 1H), 4.94 (d, *J* = 15.6 Hz, 1H), 4.75 (dd, *J* = 9.6, 1.5 Hz, 1H), 4.70 (d, *J* = 15.6 Hz, 1H), 4.61 (dd, *J* = 9.6, 6.6 Hz, 1H), 3.74 (s, 3H), 3.36 (s, 4H), 2.46 (t, *J* = 6.6 Hz, 1H), 1.31 (d, *J* = 6.1 Hz, 3H). **^{13}C NMR** (126 MHz, $CDCl_3$) δ 173.09, 168.20, 159.15, 141.40, 140.04, 129.42, 129.21, 128.95, 127.37, 127.17, 126.33, 123.23, 122.85, 121.82, 114.34, 108.46, 101.40, 77.16, 73.57, 57.00, 55.62, 53.30, 51.52, 43.14, 16.99. **HRMS** (ESI): Calcd for $(M+H)^+$ $[C_{29}H_{30}O_4N]^+$: 456.2169, found: 456.2179.

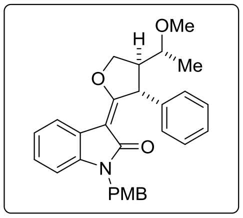

(E)-3-(-4-(-1-ethoxyethyl)-3-phenyldihydrofuran-2(3H)-ylidene)-1-methylindolin-2-one (**4n**)

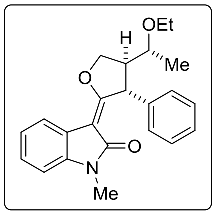

was prepared from according to the general procedure for the gold catalyzed *O*-migration reaction with MeOH as nucleophile, by using **1l** (30 mg, 0.09 mmol, 82% (E)-isomer) as starting material. After the purification done by silica gel column chromatography with Et_2O / petroleum ether = 1/2 (R_f = 0.25) as eluents, the desired product was obtained in 76% yield (26 mg, 0.07 mmol) as a yellow oil. **^{1}H NMR** (500 MHz, $CDCl_3$) δ 7.79 (d, *J* = 7.5 Hz, 1H), 7.29 (t, *J* = 7.5 Hz, 2H), 7.27 – 7.22 (m, 2H), 7.21 (d, *J* = 7.5 Hz, 1H), 7.18 (d, *J* = 7.5 Hz, 1H), 7.06 (t, *J* = 7.5 Hz, 1H), 6.79 (d, *J* = 7.5 Hz, 1H), 5.06 (s, 1H), 4.74 (d, *J* = 9.5 Hz, 1H), 4.59 (dd, *J* = 9.4, 6.5 Hz, 1H), 3.66 – 3.57 (m, 1H), 3.43 – 3.32 (m, 2H), 3.17 (s, 3H), 2.43 (t, *J* = 6.5 Hz, 1H), 1.29 (d, *J* = 6.1 Hz, 3H), 1.15 (t, *J* = 7.0 Hz, 3H). **^{13}C NMR** (126 MHz, $CDCl_3$) δ 172.70, 168.07, 141.01, 140.54, 128.95, 127.06, 126.91, 126.12, 122.78, 122.48, 121.55, 107.17, 101.16, 75.23, 73.31, 64.51, 52.98, 51.23, 25.89, 17.47, 15.55. **HRMS** (ESI): Calcd for $(M + H)^+$ $[C23H26O3N]^+$:364.1907, found:364.1902.

Gold catalyzed *O*-migration reaction

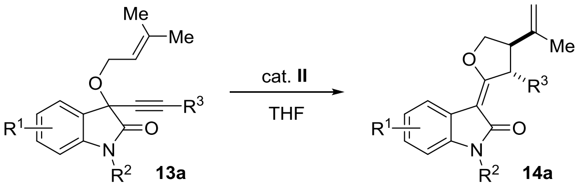

To a THF (0.6 ml) solution of the 1,6-enyne (0.1 mmol) was added a solution of cat. **II** (3.9 mg, 5 μmol) in THF (0.4 ml). After warming to room temperature, the reaction mixture was stirred overnight and TLC showed full conversion of the starting material. The reaction mixture was passed through a short pad of silica gel (Et_2O as the eluent). The resulting solution was concentrated under reduced pressure, followed by silica gel column chromatography (EtOAc / petroleum ether as the eluent) to obtain the desired product.

(E)-1-methyl-3-((3S,4S)-3-phenyl-4-(prop-1-en-2-yl)dihydrofuran-2(3H)-ylidene)indolin-2-one (**14a**) was prepared according to the general procedure for the gold catalyzed O-migration reaction, by using **13a** (49 mg, 0.15 mmol) as starting material. After the purification done by silica gel column chromatography with EtOAc / petroleum ether = 1/7 (R_f = 0.26) as eluents, the desired product was obtained in 95% yield (47 mg, 0.14 mmol) as a brown oil. The recrystallization was performed from DCM and petroleum ether.

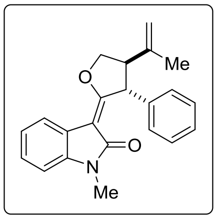

^{1}H NMR (500 MHz, $cdcl_3$) δ 7.79 (d, *J* = 7.4 Hz, 1H), 7.36 – 7.13 (m, 6H), 7.06 (t, *J* = 7.6 Hz, 1H), 6.78 (d, *J* = 7.8 Hz, 1H), 5.15 (s, 1H), 4.83 (s, 1H), 4.81 (s, 1H), 4.69 (dd, *J* = 9.4, 6.1 Hz, 1H), 4.56 (d, *J* = 9.4 Hz, 1H), 3.16 (s, 3H), 2.97 (d, *J* = 6.1 Hz, 1H), 1.80 (s, 3H). **^{13}C NMR** (126 MHz, $cdcl_3$) δ 171.96, 168.05, 144.82, 140.85, 140.70, 129.00, 127.05, 127.03, 126.30, 122.69, 122.56, 121.59, 111.65, 107.23, 101.76, 75.39, 53.84, 53.46, 25.89, 20.77. **HRMS** (ESI): Calcd for $(M + H)^+$ $[C_{22}H_{22}O_2N]^+$: 332.1645, found: 332.1660.

(E)-1-methyl-3-((3S,4S)-4-(prop-1-en-2-yl)-3-(p-tolyl)dihydrofuran-2(3H)-ylidene)indolin-2-one (**14b**) was prepared according to the general procedure for the gold catalyzed O-migration reaction, by using **13b** (37 mg, 0.11 mmol) as starting material. After the purification done by silica gel column chromatography with EtOAc / petroleum ether = 1/7 (R_f = 0.25) as eluents, the desired product was obtained in 83% yield (31 mg, 0.09 mmol) as ayellow oil. **^{1}H NMR** (500 MHz, cdcl$_3$) δ 7.79 (d, *J* = 7.6 Hz, 1H), 7.19 (td, *J* = 7.6, 1.2 Hz, 1H), 7.15 (d, *J* = 8.1 Hz, 2H), 7.11 (d, *J* = 8.1 Hz, 2H), 7.06 (td, *J* = 7.6, 1.2 Hz, 1H), 6.80 (d, *J* = 7.6 Hz, 1H), 5.10 (s, 1H), 4.81 (s, 1H), 4.80 (s, 1H), 4.69 (dd, *J* = 9.4, 6.0 Hz, 1H), 4.55 (dd, *J* = 9.4, 1.1 Hz, 1H), 3.17 (s, 3H), 2.96 (d, *J* = 6.0 Hz, 1H), 2.29 (s, 3H), 1.79 (s, 3H). **^{13}C NMR** (126 MHz, cdcl$_3$) δ 172.26, 168.06, 144.90, 140.67, 137.85, 136.62, 129.69, 126.89, 126.22, 122.74, 122.52, 121.55, 111.54, 107.20, 101.62, 75.43, 53.54, 53.52, 25.89, 21.18, 20.77. **HRMS** (ESI): Calcd for $(M + H)^+$ $[C_{23}H_{24}O_2N]^+$: 346.1802, found: 346.1804.

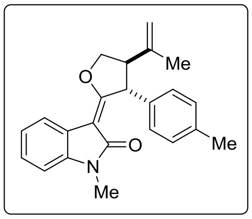

(E)-1-methyl-3-((3S,4S)-4-(prop-1-en-2-yl)-3-(m-tolyl)dihydrofuran-2(3H)-ylidene)indolin-2-one (**14c**) was prepared according to the general procedure for the gold catalyzed O-migration reaction, by using **13c** (18 mg, 0.05 mmol) as starting material. After the purification done by silica gel column chromatography with EtOAc / petroleum ether = 1/7 (R_f = 0.25) as eluents, the desired product was obtained in 77% yield (14 mg, 0.04 mmol) as a yellow oil. **^{1}H NMR** (500 MHz, CDCl$_3$) δ 7.80 (d, *J* = 7.5 Hz, 1H), 7.24 – 7.15 (m, 2H), 7.11 – 7.00 (m, 4H), 6.80 (d, *J* = 7.7 Hz, 1H), 5.12 (s, 1H), 4.83 (s, 1H), 4.81 (s, *J* = 1.2 Hz, 1H), 4.69 (dd, *J* = 9.4, 6.0 Hz, 1H), 4.55 (dd, *J* = 9.4, 0.8 Hz, 1H), 3.18 (s, 3H), 2.96 (d, *J* = 6.0 Hz, 1H), 2.32 (s, 3H), 1.80 (s, 3H). **^{13}C NMR** (126 MHz, cdcl$_3$) δ 172.10, 168.11, 144.91, 140.75, 140.71, 138.62, 128.85, 127.93, 127.67, 126.26, 124.04, 122.77, 122.57, 121.57, 111.58, 107.23, 101.75, 75.35, 53.80, 53.56, 25.92, 21.70, 20.82. **HRMS** (ESI): Calcd for $(M + H)^+$ $[C_{23}H_{24}O_2N]^+$: 346.1802, found: 346.1815.

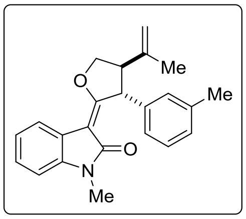

(E)-3-((3S,4S)-3-(3,4-dimethoxyphenyl)-4-(prop-1-en-2-yl)dihydrofuran-2(3H)-ylidene)-1-methylindolin-2-one (**14d**)

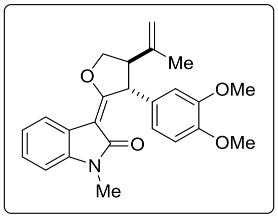

was prepared according to the general procedure for the gold catalyzed O-migration reaction, by using **13d** (41 mg, 0.10 mmol) as starting material. After the purification done by silica gel column chromatography with EtOAc / petroleum ether = 1/1 (R_f = 0.55) as eluents, the desired product was obtained in 56% yield (23 mg, 0.06 mmol) as a brown oil. **^{1}H NMR** (500 MHz, cdcl$_3$) δ 7.79 (d, *J* = 7.6 Hz, 1H), 7.20 (t, *J* = 7.6 Hz, 1H), 7.07 (t, *J* = 7.6 Hz, 1H), 6.83 (s, 1H), 6.81 (d, *J* = 7.6 Hz, 1H), 6.79 – 6.71 (m, 2H), 5.11 (s, 1H), 4.83 (s, 1H), 4.81 (s, 1H), 4.70 (dd, *J* = 9.4, 6.0 Hz, 1H), 4.55 (d, *J* = 9.4 Hz, 1H), 3.87 (s, 3H), 3.82 (s, 3H), 3.19 (s, 3H), 2.97 (d, *J* = 6.0 Hz, 1H), 1.80 (s, 3H). **^{13}C NMR** (126 MHz, cdcl$_3$) δ 172.07, 168.10, 149.38, 148.20, 144.83, 140.67, 133.41, 126.28, 122.71, 122.56, 121.58, 118.65, 111.63, 111.48, 110.83, 107.24, 101.72, 75.48, 56.09, 55.98, 53.56, 53.37, 25.91, 20.80. **HRMS** (ESI): Calcd for $(M + H)^+$ $[C_{24}H_{26}O_4N]^+$: 392.1856, found: 392.1846.

(E)-3-((3S,4S)-3-(2-methoxyphenyl)-4-(prop-1-en-2-yl)dihydrofuran-2(3H)-ylidene)-1-methylindolin-2-one (**14e**)

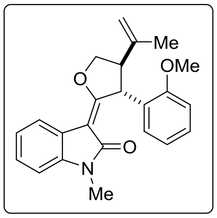

was prepared according to the general procedure for the gold catalyzed O-migration reaction, by using **13e** (60 mg, 0.17 mmol) as starting material. After the purification done by silica gel column chromatography with EtOAc / petroleum ether = 1/4 (R_f = 0.31) as eluents, the desired product was obtained in 46% yield (28 mg, 0.08 mmol) as a brown oil. **^{1}H NMR** (500 MHz, CDCl$_3$) δ 7.82 (d, *J* = 7.5 Hz, 1H), 7.25 – 7.17 (m, 2H), 7.08 (td, *J* = 7.5, 0.9 Hz, 1H), 6.96 – 6.90 (m, 2H), 6.80 (d, *J* = 7.5 Hz, 2H), 5.36 (s, 1H), 4.83 (s, 1H), 4.80 (s, 1H), 4.62 – 4.54 (m, 2H), 3.93 (s, 3H), 3.17 (s, 3H), 2.92 – 2.83 (m, 1H), 1.87 (s, 3H). **^{13}C NMR** (126 MHz, cdcl$_3$) δ 172.63, 168.06, 157.02, 145.33, 140.64, 128.82, 128.36, 126.56, 126.11, 122.82, 122.46, 121.52, 120.50, 110.96, 110.83, 107.15, 101.90, 75.44, 55.57, 51.73, 49.24, 25.86, 21.24. **HRMS** (ESI): Calcd for $(M + H)^+$ $[C_{23}H_{24}O_3N]^+$: 362.1751, found: 362.1757.

(E)-3-((3S,4S)-3-(4-fluorophenyl)-4-(prop-1-en-2-yl)dihydrofuran-2(3H)-ylidene)-1-methylindolin-2-one (**14f**) was prepared according to the general procedure for the gold catalyzed O-migration reaction, by using **13f** (31 mg, 0.09 mmol) as starting material. After the purification done by silica gel column chromatography with EtOAc / petroleum ether = 1/7 (R_f = 0.33) as eluents, the desired product was obtained in 78% yield (24 mg, 0.07 mmol) as a yellow oil. **^{1}H NMR** (500 MHz, cdcl$_3$) δ 7.79 (d, *J* = 7.6 Hz, 1H), 7.25 – 7.18 (m, 3H), 7.07 (t, *J* = 7.6 Hz, 1H), 7.00 (d, *J* = 8.6 Hz, 1H), 6.98 (d, *J* = 8.6 Hz, 1H), 6.81 (d, *J* = 7.6 Hz, 1H), 5.12 (s, 1H), 4.86 – 4.80 (m, 2H), 4.68 (dd, *J* = 9.5, 6.0 Hz, 1H), 4.57 (dd, *J* = 9.5, 1.0 Hz, 1H), 3.18 (s, 3H), 2.96 (d, *J* = 6.0 Hz, 1H), 1.80 (s, 3H). **^{13}C NMR** (126 MHz, cdcl$_3$) δ 171.61, 168.02, 161.86 (d, *J* = 245.4 Hz), 144.58, 140.72, 136.63 (d, *J* = 3.4 Hz), 128.60 (d, *J* = 8.0 Hz), 126.45, 122.62, 122.55, 121.67, 115.85 (d, *J* = 21.5 Hz), 111.84, 107.32, 101.83, 75.36, 53.48, 53.07, 25.91, 20.72. **HRMS** (ESI): Calcd for $(M + H)^+$ $[C_{22}H_{21}O_2NF]^+$: 350.1551, found: 350.1554.

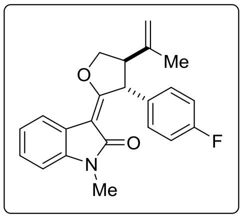

(E)-1-methyl-3-((3R,4R)-4-(prop-1-en-2-yl)-3-(thiophen-3-yl)dihydrofuran-2(3H)-ylidene)indolin-2-one (**14g**) was prepared according to the general procedure for the gold catalyzed O-migration reaction, by using **13g** (35 mg, 0.10 mmol) as starting material. After the purification done by silica gel column chromatography with EtOAc / petroleum ether = 1/6 (R_f = 0.39) as eluents, the desired product was obtained in 67% yield (23 mg, 0.07 mmol) as a yellow oil. **^{1}H NMR** (400 MHz, CDCl$_3$) δ 7.77 (d, *J* = 7.6 Hz, 1H), 7.28 (dd, *J* = 4.9, 3.0 Hz, 1H), 7.22 (t, *J* = 7.6 Hz, 1H), 7.15 – 7.04 (m, 3H), 6.85 (d, *J* = 7.6 Hz, 1H), 5.25 (s, 1H), 4.83 (s, 2H), 4.75 (dd, *J* = 9.4, 6.0 Hz, 1H), 4.61 (d, *J* = 9.4 Hz, 1H), 3.24 (s, 3H), 3.06 (d, *J* = 6.0 Hz, 1H), 1.80 (s, 3H). **^{13}C NMR** (101 MHz, CDCl$_3$) δ 173.24, 168.74, 144.33, 140.06, 139.64, 127.04, 126.41, 122.64, 122.21, 121.20, 111.86, 107.72, 101.41, 77.36, 76.15, 52.14, 49.22, 26.28, 20.89. **HRMS** (ESI): Calcd for $(M +H)^+$ [C20H20O2NS]$^+$: 338.1209, found: 338.1219.

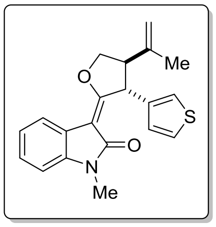

(E)-1-methyl-3-((3R,4S)-4-(prop-1-en-2-yl)-3-propyldihydrofuran-2(3H)-ylidene)indolin-2-

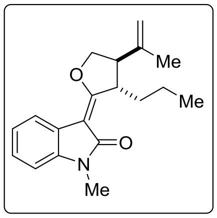

one (**14h**) was prepared according to the general procedure for the gold catalyzed O-migration reaction with 10 mol% catalyst loading, by using **13h** (18 mg, 0.06 mmol) as starting material. After the purification done by silica gel column chromatography with EtOAc / petroleum ether = 1/7 (R_f = 0.29) as eluents, the desired product was obtained in 50% yield (9 mg, 0.03 mmol) as a yellow oil. **^{1}H NMR** (500 MHz, cdcl$_3$) δ 7.68 (d, *J* = 7.6 Hz, 1H), 7.17 (td, *J* = 7.6, 0.9 Hz, 1H), 7.03 (t, *J* = 7.6 Hz, 1H), 6.82 (d, *J* = 7.6 Hz, 1H), 4.75 (s, 1H), 4.72 (s, 1H), 4.63 (dd, *J* = 9.4, 6.2 Hz, 1H), 4.52 (d, *J* = 9.4 Hz, 1H), 3.85 (dd, *J* = 10.3, 3.2 Hz, 1H), 3.28 (s, 3H), 2.88 (d, *J* = 6.2 Hz, 1H), 1.90 – 1.80 (m, 1H), 1.70 (s, 2H), 1.61 – 1.55 (m, 1H), 1.55 – 1.45 (m, 1H), 1.01 (t, *J* = 7.3 Hz, 3H). **^{13}C NMR** (126 MHz, cdcl$_3$) δ 176.05, 168.58, 145.38, 140.26, 125.83, 123.03, 122.27, 121.57, 110.98, 107.17, 100.16, 75.90, 48.47, 48.15, 34.61, 25.92, 21.45, 20.60, 13.95. **HRMS** (ESI): Calcd for $(M + H)^+$ [C19H24O2N]$^+$: 298.1802, found: 298.1805.

(E)-3-((3R,4S)-3-isopentyl-4-(prop-1-en-2-yl)dihydrofuran-2(3H)-ylidene)-1-methylindolin-

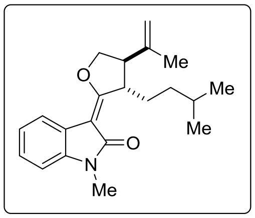

2-one (**14i**) was prepared according to the general procedure for the gold catalyzed O-migration reaction, by using **13i** (35 mg, 0.11 mmol) as starting material. After the purification done by silica gel column chromatography with EtOAc / petroleum ether = 1/7 (R_f = 0.31) as eluents, the desired product was obtained in 36% yield (12 mg, 0.04 mmol) as a yellow oil. **^{1}H NMR** (500 MHz, cdcl$_3$) δ 7.45 (dd, *J* = 7.6, 0.7 Hz, 1H), 7.31 (td, *J* = 7.6, 1.2 Hz, 1H), 7.09 (td, *J* = 7.6, 0.7 Hz, 1H), 6.79 (d, *J* = 7.8 Hz, 1H), 5.34 (t, *J* = 7.2 Hz, 1H), 4.31 (dd, *J* = 10.5, 7.2 Hz, 1H), 4.24 (dd, *J* = 10.5, 7.2 Hz, 1H), 3.18 (s, 3H), 2.24 (td, *J* = 7.4, 1.6 Hz, 2H), 1.69 (s, 3H), 1.67 – 1.61 (m, 2H), 1.41 (td, *J* = 7.4 Hz, 2H), 0.85 (dd, *J* = 6.6, 2.3 Hz, 7H).**^{13}C NMR** (126 MHz, cdcl$_3$) δ 172.42, 143.29, 137.98, 130.23, 128.68, 124.84, 123.38, 120.62, 108.58, 89.47, 74.84, 73.90, 62.15, 37.33, 27.44, 26.45, 25.91, 22.22, 18.13, 17.12. **HRMS** (ESI): Calcd for $(M + Na)^+$ $[C_{21}H_{27}O_2NNa]^+$: 348.1934, found: 348.1945.

(E)-1,5-dimethyl-3-((3S,4S)-3-phenyl-4-(prop-1-en-2-yl)dihydrofuran-2(3H)-

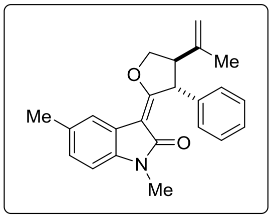

ylidene)indolin-2-one (**14j**) was prepared according to the general procedure for the gold catalyzed O-migration reaction, by using **13j** (24 mg, 0.07 mmol) as starting material. After the purification done by silica gel column chromatography with EtOAc / petroleum ether = 1/5 (R_f = 0.27) as eluents, the desired product was obtained in 76% yield (18 mg, 0.05 mmol) as a brown oil. **^{1}H NMR** (400 MHz, $CDCl_3$) δ 7.66 (s, 1H), 7.35 – 7.23 (m, 5H), 7.03 (dd, *J* = 7.8, 0.8 Hz, 1H), 6.72 (d, *J* = 7.9 Hz, 1H), 5.18 (s, 1H), 4.86 (s, 1H), 4.84 (s, 1H), 4.72 (dd, *J* = 9.6, 6.0 Hz, 1H), 4.60 (dd, *J* = 9.5, 1.0 Hz, 1H), 3.18 (s, 3H), 3.00 (d, *J* = 6.0 Hz, 1H), 2.43 (s, 3H), 1.84 (s, 3H). **^{13}C NMR** (101 MHz, $CDCl_3$) δ 171.64, 168.10, 144.86, 140.89, 138.54, 130.97, 128.99, 127.02, 126.65, 123.33, 122.68, 111.63, 106.97, 101.86, 75.29, 53.76, 53.43, 25.93, 21.42, 20.79. **HRMS** (ESI): Calcd for $(M + H)^+$ $[C_{23}H_{24}O_2N]^+$: 346.1802, found: 346.1812.

(E)-5-methoxy-1-methyl-3-((3S,4S)-3-phenyl-4-(prop-1-en-2-yl)dihydrofuran-2(3H)-

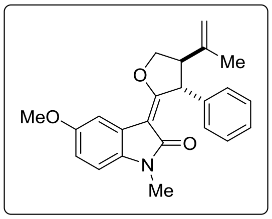

ylidene)indolin-2-one (**14k**) was prepared according to the general procedure for the gold catalyzed O-migration reaction, by using **13k** (27 mg, 0.07 mmol) as starting material. After the purification done by silica gel column chromatography with EtOAc / petroleum ether = 1/4 (R_f = 0.27) as eluents, the desired product was obtained in 66% yield (18 mg, 0.05 mmol) as a yellow oil. **^{1}H NMR** (300 MHz, $CDCl_3$) δ 7.44 (d, *J* = 2.5 Hz, 1H), 7.37 – 7.16 (m, 5H), 6.76 (dd, *J* = 8.4, 2.5 Hz, 1H), 6.68 (d, *J* = 8.4 Hz, 1H), 5.15 (s, 1H), 4.83 (s, 1H), 4.81 (s, 1H), 4.70 (dd, *J* = 9.5, 6.0 Hz, 1H), 4.57 (d, *J* = 9.5 Hz, 1H), 3.86 (s, 3H), 3.14 (s, 3H), 2.98 (d, *J* = 6.0 Hz, 1H), 1.80 (s, 3H). **^{13}C NMR** (75 MHz, $CDCl_3$) δ 172.10, 167.96, 155.49, 144.80, 140.82, 134.85, 129.01, 127.06, 127.02, 123.59, 111.67, 111.35, 109.61, 107.39, 102.12, 75.44, 56.17, 53.81, 53.39, 25.97, 20.77. **HRMS** (ESI): Calcd for $(M +Na)^+$ [C23H23O3NNa]$^+$: 384.1570, found: 384.1579.

(E)-6-bromo-1-methyl-3-((3S,4S)-3-phenyl-4-(prop-1-en-2-yl)dihydrofuran-2(3H)-

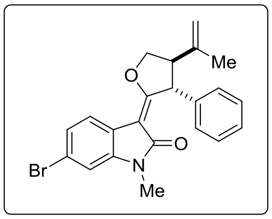

ylidene)indolin-2-one (**14l**) was prepared according to the general procedure for the gold catalyzed O-migration reaction, by using **13l** (27 mg, 0.07 mmol) as starting material. After the purification done by silica gel column chromatography with EtOAc / petroleum ether = 1/5 (R_f = 0.54) as eluents, the desired product was obtained in 69% yield (19 mg, 0.05 mmol) as a brown oil. **^{1}H NMR** (300 MHz, $CDCl_3$) δ 7.59 (d, *J* = 8.0 Hz, 1H), 7.40 – 7.17 (m, 6H), 6.90 (d, *J* = 1.7 Hz, 1H), 5.08 (s, 1H), 4.79 (s, 2H), 4.68 (dd, *J* = 9.5, 6.0 Hz, 1H), 4.55 (dd, *J* = 9.5, 1.1 Hz, 1H), 3.11 (s, 3H), 2.95 (d, *J* = 6.0 Hz, 1H), 1.77 (s, 3H). **^{13}C NMR** (75 MHz, $CDCl_3$) δ 172.76, 167.84, 144.64, 141.78, 140.50, 129.07, 127.19, 126.99, 124.31, 123.56, 121.55, 119.61, 111.77, 110.62, 101.02, 75.69, 54.00, 53.34, 25.99, 20.76. **HRMS** (ESI): Calcd for $(M + H)^+$ $[C22H21O2NBr]^+$: 410.0750, found: 410.1757.

(E)-5-fluoro-1-methyl-3-((3S,4S)-3-phenyl-4-(prop-1-en-2-yl)dihydrofuran-2(3H)-

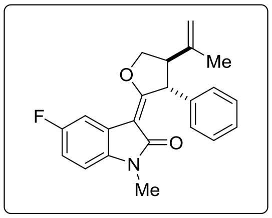

ylidene)indolin-2-one (**14m**) was prepared according to the general procedure for the gold catalyzed O-migration reaction, by using **13m** (31 mg, 0.09 mmol) as starting material. After the purification done by silica gel column chromatography with EtOAc / petroleum ether = 1/4 (R_f = 0.33) as eluents, the desired product was obtained in 62% yield (19 mg, 0.05 mmol) as a yellow oil. **^{1}H NMR** (400 MHz, $CDCl_3$) δ 7.53 (dd, *J* = 8.9, 2.6 Hz, 1H), 7.39 – 7.28 (m, 2H), 7.28 – 7.19 (m, 3H), 6.89 (td, *J* = 8.9, 2.6 Hz, 1H), 6.68 (dd, *J* = 8.9, 4.2 Hz, 1H), 5.14 (s, 1H), 4.83 (s, 2H), 4.72 (dd, *J* = 9.5, 6.1 Hz, 1H), 4.60 (dd, *J* = 9.5, 1.0 Hz, 1H), 3.16 (s, 3H), 2.99 (d, *J* = 6.1 Hz, 1H), 1.81 (s, 3H). **^{13}C NMR** (101 MHz, $CDCl_3$) δ 173.01, 167.86, 158.99 (d, *J* = 236.7 Hz), 144.62, 140.51, 136.69, 129.07, 127.17, 126.98, 123.64 (d, *J* = 10.0 Hz), 112.22 (d, *J* = 24.0 Hz), 111.75, 101.68, 110.13 (d, *J* = 26.0 Hz), 107.31 (d, *J* = 8.5 Hz), 75.73, 53.94, 53.33, 26.03, 20.76. **HRMS** (ESI): Calcd for $(M + H)^+$ $[C_{22}H_{21}O_2NF]^+$: 350.1551, found: 350.1558.

(E)-1-methyl-3-((3S,4S)-3-phenyl-4-(prop-1-en-2-yl)dihydrofuran-2(3H)-ylidene)-5-

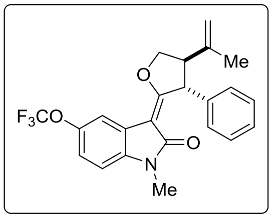

(trifluoromethoxy)indolin-2-one (**14n**) was prepared according to the general procedure for the gold catalyzed O-migration reaction, by using **13n** (28 mg, 0.07 mmol) as starting material. After the purification done by silica gel column chromatography with EtOAc / petroleum ether = 1/4 (R_f = 0.38) as eluents, the desired product was obtained in 74% yield (21 mg, 0.05 mmol) as a brown oil. **^{1}H NMR** (300 MHz, $CDCl_3$) δ 7.66 (d, *J* = 1.5 Hz, 1H), 7.39 – 7.28 (m, 2H), 7.28 – 7.17 (m, 3H), 7.06 (dd, *J* = 8.4, 1.3 Hz, 1H), 6.74 (d, *J* = 8.4 Hz, 1H), 5.14 (s, 1H), 4.84 (s, 2H), 4.75 (dd, *J* = 9.6, 6.1 Hz, 1H), 4.62 (dd, *J* = 9.6, 1.1 Hz, 1H), 3.17 (s, 3H), 3.00 (d, *J* = 5.9 Hz, 1H), 1.81 (s, 3H). **^{13}C NMR** (75 MHz, $CDCl_3$) δ 173.57, 167.89, 144.55, 144.27, 140.42, 139.12, 129.10, 127.23, 127.00, 124.68 (d, *J* = 317.8 Hz), 123.58, 119.13, 116.20, 111.83, 107.28, 101.21, 75.94, 54.07, 53.30, 26.04, 20.77. **HRMS** (ESI): Calcd for $(M + H)^+$ $[C_{23}H_{21}O_3NF_3]^+$: 416.1468, found: 416.1460.

(E)-1-methyl-5-nitro-3-((3S,4S)-3-phenyl-4-(prop-1-en-2-yl)dihydrofuran-2(3H)-

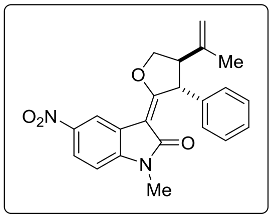

ylidene)indolin-2-one (**14o**) was prepared according to the general procedure for the gold catalyzed O-migration reaction, by using **13o** (28 mg, 0.07 mmol) as starting material. After the purification done by silica gel column chromatography with EtOAc / petroleum ether = 1/4 (R_f = 0.18) as eluents, the desired product was obtained in 64% yield (18 mg, 0.05 mmol) as a yellow oil. **^{1}H NMR** (400 MHz, $CDCl_3$) δ 8.66 (d, *J* = 2.3 Hz, 1H), 8.20 (dd, *J* = 8.3, 2.3 Hz, 1H), 7.43 – 7.31 (m, 2H), 7.31 - 7.18 (m, 3H), 6.86 (d, *J* = 8.6 Hz, 1H), 5.15 (s, 1H), 4.98 – 4.78 (m, 3H), 4.74 (dd, *J* = 9.6, 1.0 Hz, 1H), 3.26 (s, 3H), 3.06 (d, *J* = 5.8 Hz, 1H), 1.84 (s, 3H). **^{13}C NMR** (101 MHz, $CDCl_3$) δ 175.30, 168.02, 145.37, 144.31, 142.88, 139.93, 129.19, 127.42, 126.96, 123.20, 122.86, 117.89, 112.00, 106.54, 100.04, 76.56, 54.48, 53.16, 26.32, 20.73. **HRMS** (ESI): Calcd for $(M + H)^+$ $[C_{22}H_{20}O_4N_2]^+$: 377.1496, found: 377.1503.

(E)-5-chloro-1-methyl-3-((3S,4S)-3-phenyl-4-(prop-1-en-2-yl)dihydrofuran-2(3H)-ylidene)indolin-2-one (**14p**) was prepared according to the general procedure for the gold catalyzed O-migration reaction, by using **13p** (31 mg, 0.08 mmol) as starting material. After the purification done by silica gel column chromatography with EtOAc / petroleum ether = 1/5 (R_f = 0.29) as eluents, the desired product was obtained in 65% yield (20 mg, 0.05 mmol) as a yellow oil.

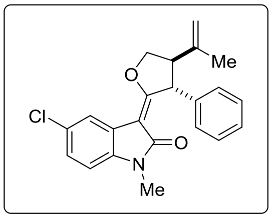

^{1}H NMR (400 MHz, $CDCl_3$) δ 7.78 (d, *J* = 2.1 Hz, 1H), 7.38 – 7.29 (m, 2H), 7.28 – 7.21 (m, 3H), 7.17 (dd, *J* = 8.3, 2.1 Hz, 1H), 6.71 (d, *J* = 8.3 Hz, 1H), 5.14 (s, 1H), 4.84 (s, 2H), 4.74 (dd, *J* = 9.5, 6.0 Hz, 1H), 4.62 (dd, *J* = 9.5, 1.1 Hz, 1H), 3.16 (s, 3H), 3.00 (d, *J* = 6.0 Hz, 1H), 1.82 (s, 3H). **^{13}C NMR** (101 MHz, $CDCl_3$) δ 173.24, 167.67, 144.59, 140.44, 139.07, 129.08, 127.20, 126.98, 126.92, 125.84, 123.99, 122.56, 111.79, 107.99, 101.05, 75.80, 54.02, 53.31, 26.01, 20.76. **HRMS** (ESI): Calcd for $(M + H)^+$ $[C_{22}H_{21}O_2NCl]^+$: 366.1255, found: 366.1267.

(E)-1-benzyl-3-((3S,4S)-3-phenyl-4-(prop-1-en-2-yl)dihydrofuran-2(3H)-ylidene)indolin-2-one (**14q**) was prepared according to the general procedure for the gold catalyzed O-migration reaction, by using **13q** (25 mg, 0.06 mmol) as starting material. After the purification done by silica gel column chromatography with EtOAc / petroleum ether = 1/4 (R_f = 0.61) as eluents, the desired product was obtained in 96% yield (24 mg, 0.06 mmol) as a yellow oil.

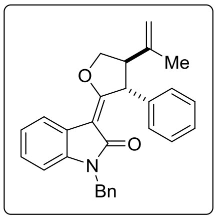

^{1}H NMR (500 MHz, $CDCl_3$) δ 7.83 (dd, *J* = 7.2, 1.1 Hz, 1H), 7.37 – 7.16 (m, 10H), 7.14 – 7.00 (m, 2H), 6.71 (d, *J* = 7.2 Hz, 1H), 5.20 (s, 1H), 5.00 (d, *J* = 15.9 Hz, 1H), 4.87 (s, 1H), 4.86 (s, 1H), 4.80 (d, *J* = 15.9 Hz, 1H), 4.73 (dd, *J* = 9.5, 6.0 Hz, 1H), 4.61 (dd, *J* = 9.5, 1.2 Hz, 1H), 3.02 (d, *J* = 6.0 Hz, 1H), 1.85 (s, 3H). **^{13}C NMR** (126 MHz, $CDCl_3$) δ 172.87, 168.26, 144.68, 140.73, 139.67, 136.75, 129.02, 128.70, 128.41, 127.36, 127.31, 127.11, 127.05, 126.28, 122.84, 122.69, 121.88, 111.79, 108.44, 101.64, 75.62, 54.09, 53.55, 43.59, 20.84. **HRMS** (ESI): Calcd for $(M + H)^+$ $[C_{28}H_{26}O_2N]^+$: 408.1958, found: 408.1958.

(E)-1-(4-methoxybenzyl)-3-((3S,4S)-3-phenyl-4-(prop-1-en-2-yl)dihydrofuran-2(3H)-

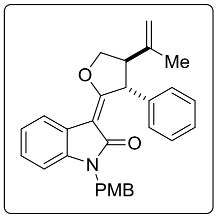

ylidene)indolin-2-one (**14r**) was prepared according to the general procedure for the gold catalyzed O-migration reaction, by using **13r** (33 mg, 0.08 mmol) as starting material. After the purification done by silica gel column chromatography with EtOAc / petroleum ether = 1/4 (R_f = 0.30) as eluents, the desired product was obtained in 83% yield (27 mg, 0.06 mmol) as a brown oil. **^{1}H NMR** (400 MHz, $CDCl_3$) δ 7.82 (dd, *J* = 7.5, 0.9 Hz, 1H), 7.43 – 7.20 (m, 5H), 7.16 (d, *J* = 8.6 Hz, 2H), 7.13 – 6.98 (m, 2H), 6.79 (d, *J* = 8.6 Hz, 2H), 6.71 (d, *J* = 7.5 Hz, 1H), 5.20 (s, 1H), 4.94 (d, *J* = 15.6 Hz, 1H), 4.87 (s, 1H), 4.85 (s, 1H), 4.77 – 4.67 (m, 2H), 4.59 (dd, *J* = 9.5, 1.0 Hz, 1H), 3.75 (s, 3H), 3.01 (d, *J* = 5.8 Hz, 1H), 1.84 (s, 3H). **^{13}C NMR** (101 MHz, $CDCl_3$) δ 172.24, 167.95, 158.84, 144.73, 140.83, 139.82, 129.03, 128.99, 128.68, 127.03, 127.01, 126.20, 122.84, 122.61, 121.59, 114.04, 111.72, 108.25, 101.63, 75.42, 55.33, 53.91, 53.51, 42.89, 20.85. **HRMS** (ESI): Calcd for $(M + H)^+$ $[C_{29}H_{28}O_3N]^+$: 438.2064, found: 438.2065.

(E)-1-(methoxymethyl)-3-((3S,4S)-3-phenyl-4-(prop-1-en-2-yl)dihydrofuran-2(3H)-

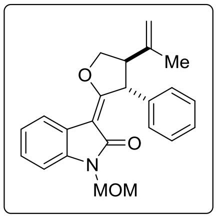

ylidene)indolin-2-one (**14s**) was prepared according to the general procedure for the gold catalyzed O-migration reaction, by using **13s** (39 mg, 0.11 mmol) as starting material. After the purification done by silica gel column chromatography with EtOAc / petroleum ether = 1/7 (R_f = 0.37) as eluents, the desired product was obtained in 97% yield (38 mg, 0.10 mmol) as a brown oil. **^{1}H NMR** (500 MHz, $cdcl_3$) δ 7.83 (d, *J* = 6.9 Hz, 1H), 7.35 – 7.17 (m, 6H), 7.11 (td, *J* = 7.7, 0.9 Hz, 1H), 7.01 (d, *J* = 7.7 Hz, 1H), 5.15 (d, *J* = 10.9 Hz, 1H), 5.14 (s, 1H), 5.04 (d, *J* = 10.9 Hz, 1H), 4.85 (s, 1H), 4.84 (s, 1H), 4.71 (dd, *J* = 9.5, 5.7 Hz, 1H), 4.59 (dd, *J* = 9.5, 1.2 Hz, 1H), 3.25 (s, 3H), 3.00 (d, *J* = 5.7 Hz, 1H), 1.82 (s, 3H). **^{13}C NMR** (126 MHz, $cdcl_3$) δ 172.76, 168.38, 144.69, 140.66, 139.08, 129.00, 128.38, 127.08, 126.99, 126.47, 122.72, 122.25, 111.75, 108.64, 101.50, 75.58, 71.13, 56.16, 54.04, 53.51, 20.78. **HRMS** (ESI): Calcd for $(M + Na)^+$ $[C_{23}H_{23}O_3NNa]^+$: 384.1570, found: 384.1584.

(E)-3-((3S,4S)-3-phenyl-4-(prop-1-en-2-yl)dihydrofuran-2(3H)-ylidene)-1-((2-(trimethylsilyl)ethoxy)methyl)indolin-2-one (**14t**) was prepared according to the general procedure for the gold catalyzed O-migration reaction, by using **13t** (22 mg, 0.05 mmol) as starting material. After the purification done by silica gel column chromatography with EtOAc / petroleum ether = 1/10 (R_f = 0.42) as eluents, the desired product was obtained in 51% yield (11 mg, 0.03 mmol) as a yellow oil. **^{1}H NMR** (500 MHz, cdcl$_3$) δ 7.89 (d, *J* = 7.6 Hz, 1H), 7.43 – 7.35 (m, 2H), 7.33 (d, *J* = 8.9 Hz, 2H), 7.29 (d, *J* = 5.9 Hz, 1H), 7.27 (d, *J* = 7.6 Hz, 1H), 7.17 (t, *J* = 7.6 Hz, 1H), 7.10 (d, *J* = 7.6 Hz, 1H), 5.24 (d, *J* = 11.0 Hz, 1H), 5.23 (s, 1H), 5.16 (d, *J* = 11.0 Hz, 1H), 4.92 (s, 1H), 4.91 (d, *J* = 1.1 Hz, 1H), 4.78 (dd, *J* = 9.4, 6.2 Hz, 1H), 4.66 (d, *J* = 9.4 Hz, 1H), 3.60 – 3.48 (m, 2H), 3.08 (d, *J* = 6.2 Hz, 1H), 1.89 (s, 3H), 0.92 (dd, *J* = 8.2, 8.0 Hz, 2H), -0.03 (s, 9H). **^{13}C NMR** (126 MHz, cdcl$_3$) δ 172.57, 168.23, 144.76, 140.68, 139.27, 128.99, 127.07, 127.02, 126.43, 122.73, 122.65, 122.11, 111.73, 108.81, 101.57, 75.54, 69.14, 65.74, 53.93, 53.48, 20.76, 17.93, -1.35. **HRMS** (ESI): Calcd for $(M + Na)^+$ $[C_{27}H_{33}O_3NSiNa]^+$: 470.2122, found: 470.2137.

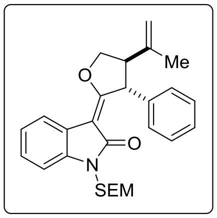

(E)-3-((3S,4S)-3-phenyl-4-(prop-1-en-2-yl)dihydrofuran-2(3H)-ylidene)indolin-2-one (**14u**) was prepared according to the general procedure for the gold catalyzed O-migration reaction, by using **13u** (33 mg, 0.01 mmol) as starting material. After the purification done by silica gel column chromatography with EtOAc / petroleum ether = 1/2 (R_f = 0.23) as eluents, the desired product was obtained in 95% yield (31 mg, 0.10 mmol) as a yellow oil. **^{1}H NMR** (500 MHz, CDCl$_3$) δ 7.89 (s, 1H), 7.77 (d, *J* = 7.6 Hz, 1H), 7.35 – 7.27 (m, 2H), 7.28 – 7.18 (m, 3H), 7.12 (td, *J* = 7.6, 1.0 Hz, 1H), 7.04 (td, *J* = 7.6, 0.6 Hz, 1H), 6.77 (d, *J* = 7.6 Hz, 1H), 5.12 (s, 1H), 4.84 (s, 1H), 4.83 (s, 1H), 4.71 (dd, *J* = 9.5, 6.0 Hz, 1H), 4.58 (dd, *J* = 9.5, 1.0 Hz, 1H), 3.00 (d, *J* = 6.0 Hz, 1H), 1.81 (s, 3H). **^{13}C NMR** (126 MHz, CDCl$_3$) δ 172.78, 169.50, 144.73, 140.69, 137.85, 128.93, 127.13, 127.09, 126.33, 123.59, 122.87, 121.65, 111.80, 108.96, 101.88, 75.65, 53.87, 53.44, 20.71. **HRMS** (ESI): Calcd for $(M +Na)^+$ [C21H19O2NNa]$^+$: 340.1308, found: 340.1312.

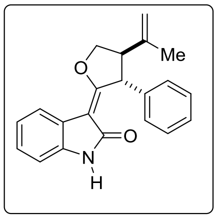

Gold catalyzed *O*-migration reaction with deuterated CD_3OD as nucleophile

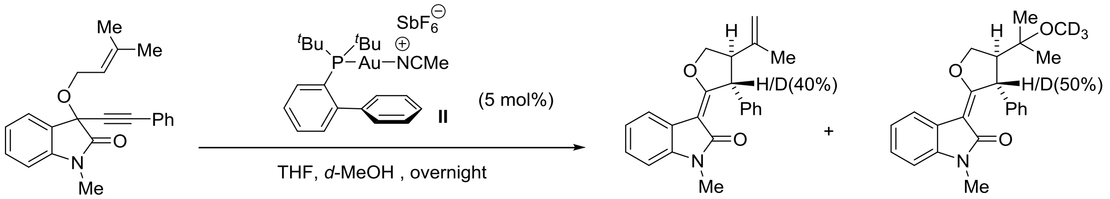

13 **14** 12% **21** 47%

At 0 °C, to a THF (0.4 ml) solution of **13** (0.08 mmol) and *d*-MeOH (53 µL, 0.75 mmol) was added a solution of cat. **II** (3 mg, 4 µmol) in THF (0.4 mL). The reaction mixture was stirred at room temperature7 overnight. Afterwards, the reaction mixture was passed through a short pad of silica gel (Et_2O as the eluent). The resulting solution was concentrated under reduced pressure, followed by silica gel column chromatography (EA/PE=1:4) to obtain compounds **14** in 12% yield **(**3 mg, 0.01 mmol, *D*-40%**)** as a yellow oil and **21** in 47% yield (13 mg, 0.04 mmol, *D*-50%**)** as a yellow oil.

(E)-1-methyl-3-((3S,4S)-3-phenyl-4-(prop-1-en-2-yl)dihydrofuran-2(3H)-ylidene)indolin-2-one (**14**) **^{1}H NMR** (300 MHz, cdcl$_3$) δ 7.79 (d, *J* = 7.4 Hz, 1H), 7.36 – 7.13 (m, 6H), 7.06 (t, *J* = 7.6 Hz, 1H), 6.80 (d, *J* = 7.8 Hz, 1H), 5.15 (s, 0:6H), 4.83 (s, 1H), 4.81 (s, 1H), 4.69 (dd, *J* = 9.4, 6.1 Hz, 1H), 4.56 (d, *J* = 9.4 Hz, 1H), 3.17 (s, 3H), 2.98 (d, *J* = 6.1 Hz, 1H), 1.80 (s, 3H).

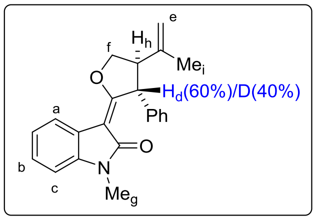

(E)-3-((3R,4S)-4-(2-(methoxy-d3)propan-2-yl)-3-phenyldihydrofuran-2(3H)-ylidene)-1-methylindolin-2-one (**20**) ^{1}H NMR (300 MHz, $CDCl_3$) δ 7.75 (d, *J* = 7.5 Hz, 1H), 7.34 – 7.12 (m, 6H), 7.04 (t, *J* = 7.5 Hz, 1H), 6.77 (d, *J* = 7.5 Hz, 1H), 5.10 (s, 0.5H), 4.73 (dd, *J* = 9.9, 1.7 Hz, 1H), 4.64 (dd, *J* = 9.9, 6.8 Hz, 1H), 3.15 (s, 3H), 2.51 (d, *J* = 6.8 Hz, 1H), 1.26 (s, 3H), 1.09 (s, 3H).

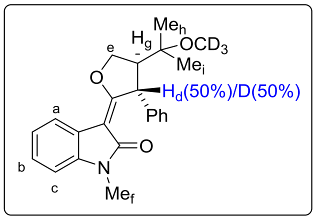

For NMR spectra of compounds in this experiment, see Supplementary Figures 130.

Compounds isolated from the condition screening

(E)-1-methyl-3-(2-oxo-2-phenylethylidene)indolin-2-one (MS) ^{1}**H NMR** (500 MHz, $CDCl_3$) δ 8.32 (d, *J* = 7.7 Hz, 1H), 8.11 (d, *J* = 7.5 Hz, 2H), 7.89 (s, 1H), 7.63 (t, *J* = 7.5 Hz, 1H), 7.53 (t, *J* = 7.5 Hz, 2H), 7.37 (td, *J* = 7.7, 0.9 Hz, 1H), 7.03 (t, *J* = 7.7 Hz, 1H), 6.81 (d, *J* = 7.7 Hz, 1H), 3.27 (s, 3H).[11]

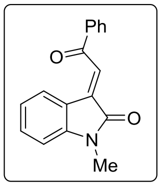

(E)-3-(-4-(-1-hydroxyethyl)-3-phenyldihydrofuran-2(3H)-ylidene)-1-methylindolin-2-one (4OH) ^{1}**H NMR** (500 MHz, CDCl3) δ 7.78 (d, J = 7.5 Hz, 1H), 7.34 – 7.23 (m, 4H), 7.23 – 7.14 (m, 2H), 7.06 (t, *J* = 7.5 Hz, 1H), 6.76 (d, *J* = 7.5 Hz, 1H), 5.07 (s, 1H), 4.81 (d, *J* = 9.6 Hz, 1H), 4.62 (dd, *J* = 9.6, 6.3 Hz, 1H), 3.89 (qd, *J* = 6.3 Hz, 6.3 Hz, 1H), 3.12 (s, 3H), 2.40 (dd, *J* = 6.3 Hz, 1H), 1.35 (d, *J* = 6.3 Hz, 3H), 1.25 (s, 1H). ^{13}**C NMR** (126 MHz, $CDCl_3$) δ 172.52, 168.14, 140.97, 140.49, 129.00, 127.04, 127.00, 126.20, 122.70, 122.53, 121.66, 107.26, 101.20, 72.92, 68.34, 54.15, 51.51, 25.88, 21.70. **HRMS** (ESI): Calcd for $(M + H)^+$ $[C21H22O3N]^+$: 336.1594, found: 336.1589.

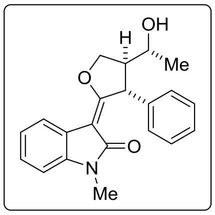

1-(-5-((E)-1-methyl-2-oxoindolin-3-ylidene)-4-phenyltetrahydrofuran-3-yl)ethyl acetate (4OAc) Here we obtained a pair of HPLC inseparable epimeric mixtures at the carbon next to acetate in the ratio 4/3 (**4OAc**/**epi-4OAc**). ^{1}H NMR (500 MHz, $CDCl_3$) δ 7.79 (d, *J* = 7.3 Hz, 1H, 4OAc), 7.77 (d, *J* = 7.2 Hz, 1H, epi-4OAc), 7.35 – 7.18 (m, 6H, 4OAc and epi-4OAc), 7.10 - 7.05 (m, 1H, 4OAc and epi-4OAc), 6.82 (d, *J* = 7.7 Hz, 1H, 4OAc), 6.79 (d, *J* = 7.7 Hz, 1H, epi-4OAc), 5.19 - 5.18 (m, 1H, 4OAc and epi-4OAc), 5.04 (qd, *J* = 6.3, 6.3 Hz, 1H, 4OAc), 4.76 (dd, *J* = 8.7, 8.7 Hz, 1H, epi-4OAc), 4.69 - 4.55 (m, 2H, 4OAc and epi-4OAc), 3.20 (s, 3H, 4OAc), 3.18 (s, 3H, epi-4OAc), 3.00 – 2.93 (m, 1H, epi-4OAc), 2.67 (t, *J* = 6.3 Hz, 1H, 4OAc), 2.04 (s, 3H, 4OAc), 1.91 (s, 3H, epi-4OAc), 1.34 - 1.27 (m, 3H, 4OAc and epi-4OAc). **HRMS** (ESI): Calcd for $(M + H)^+$ $[C23H24O4N]^+$: 378.1700, found: 378.1700.

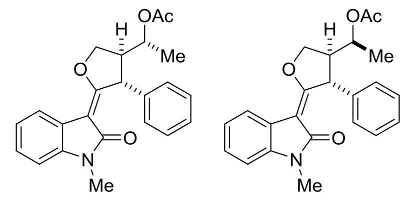

4OAc **epi-4OAc**

1,3-dimethyl-1a-phenyl-1a,3,9,9a-tetrahydrocyclopropa[4,5]pyrano[3,2-c]quinolin-

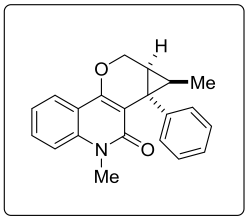

2(1H)-one (**epi-3a**) **^{1}H NMR** (500 MHz, $CDCl_3$) δ 7.91 (dd, *J* = 8.0, 1.4 Hz, 1H), 7.51 – 7.45 (m, 1H), 7.33 (d, *J* = 7.0 Hz, 2H), 7.23 (d, *J* = 8.4 Hz, 1H), 7.19 – 7.14 (m, 3H), 7.08 (t, *J* = 7.3 Hz, 1H), 4.87 (dd, *J* = 12.3, 8.5 Hz, 1H), 4.02 (dd, *J* = 12.3, 5.3 Hz, 1H), 3.51 (s, 3H), 2.14 (dq, *J* = 8.1, 6.4 Hz, 1H), 1.58 – 1.51 (m, 1H), 1.02 (d, *J* = 6.4 Hz, 3H). **^{13}C NMR** (126 MHz, $CDCl_3$) δ 162.26, 158.39, 144.56, 139.18, 130.65, 128.92, 128.09, 126.20, 123.64, 121.60, 116.10, 113.99, 109.62, 65.15, 29.40, 25.93, 22.60, 21.62, 8.96. **HRMS** (ESI): Calcd for (M + H) $^{+}$ [C21H20O2N] $^{+}$: 318.1489, found: 318.1489.

For NMR spectra of compounds in this section, see Supplementary Figures 131-134.

Supplementary References

1. Amijs, C.H.M. et al. Gold(I)-Catalyzed Intermolecular Addition of Carbon Nucleophiles to 1,5- and 1,6-Enynes. *The Journal of Organic Chemistry* **73**, 7721-7730 (2008).
2. Nieto-Oberhuber, C. et al. Gold(I)-Catalyzed Cyclizations of 1,6-Enynes: Alkoxycyclizations and exo/endo Skeletal Rearrangements. *Chemistry – A European Journal* **12**, 1677-1693 (2006).
3. Mauleón, P., Zeldin, R.M., González, A.Z. & Toste, F.D. Ligand-Controlled Access to [4 + 2] and [4 + 3] Cycloadditions in Gold-Catalyzed Reactions of Allene-Dienes. *Journal of the American Chemical Society* **131**, 6348-6349 (2009).
4. Huang, J., Mao, T. & Zhu, Q. Copper-Catalyzed Intramolecular Oxidative C(sp3)–H Amidation of 2-Aminoacetophenones: Efficient Synthesis of Indoline-2,3-diones. *European Journal of Organic Chemistry* **2014**, 2878-2882 (2014).
5. Wee, X.K., Yang, T. & Go, M.L. Exploring the Anticancer Activity of Functionalized Isoindigos: Synthesis, Drug-like Potential, Mode of Action and Effect on Tumor-Induced Xenografts. *ChemMedChem* **7**, 777-791 (2012).
6. Beauchard, A. et al. Synthesis of novel 5-substituted indirubins as protein kinases inhibitors. *Bioorganic & Medicinal Chemistry* **14**, 6434-6443 (2006).
7. El Bouakher, A. et al. A General and Efficient Method to Access Tetracyclic Spirooxindole Derivatives. *European Journal of Organic Chemistry* **2015**, 556-569 (2015).
8. Itoh, T., Ishikawa, H. & Hayashi, Y. Asymmetric Aldol Reaction of Acetaldehyde and Isatin Derivatives for the Total Syntheses of ent-Convolutamydine E and CPC-1 and a Half Fragment of Madindoline A and B. *Organic Letters* **11**, 3854-3857 (2009).
9. Ellis, J.M., Overman, L.E., Tanner, H.R. & Wang, J. A Versatile Synthesis of Unsymmetrical 3,3′-Bioxindoles: Stereoselective Mukaiyama Aldol Reactions of 2-Siloxyindoles with Isatins. *The Journal of Organic Chemistry* **73**, 9151-9154 (2008).
10. Chouhan, M., Senwar, K.R., Kumar, K., Sharma, R. & Nair, V.A. Catalytic C–H Activation of Arylacetylenes: A Fast Assembly of 3-(Arylethynyl)-3-hydroxyindolin-2-ones Using CuI/DBU. *Synthesis* **46**, 195-202 (2014).
11. Faita, G., Mella, M., Righetti, P. & Tacconi, G. An easy lewis acid-mediated isomerization from (E)- to (Z)-Oxoindolin-3-ylidene ketones. *Tetrahedron* **50**, 10955-10962 (1994).
